# A DNA Barcode Library for North American Ephemeroptera: Progress and Prospects

**DOI:** 10.1371/journal.pone.0038063

**Published:** 2012-05-30

**Authors:** Jeffrey M. Webb, Luke M. Jacobus, David H. Funk, Xin Zhou, Boris Kondratieff, Christy J. Geraci, R. Edward DeWalt, Donald J. Baird, Barton Richard, Iain Phillips, Paul D. N. Hebert

**Affiliations:** 1 Biodiversity Institute of Ontario, University of Guelph, Guelph, Ontario, Canada; 2 Division of Science, Indiana University Purdue University Columbus, Columbus, Indiana, United States of America; 3 Stroud Water Research Center, Avondale, Pennsylvania, United States of America; 4 BGI, Shenzhen, Guangdong Province, China; 5 Department of Bioagricultural Sciences and Pest Management, Colorado State University, Fort Collins, Colorado, United States of America; 6 Department of Entomology, National Museum of Natural History, Smithsonian Institution, Washington, D. C., United States of America; 7 Prairie Research Institute, Illinois Natural History Survey, University of Illinois, Champaign, Illinois, United States of America; 8 Environment Canada, Canadian Rivers Institute, Department of Biology, University of New Brunswick, Fredericton, New Brunswick, Canada; 9 Laboratory of Aquatic Entomology, Florida A&M University, Tallahassee, Florida, United States of America; 10 Saskatchewan Watershed Authority, Saskatoon, Saskatchewan, Canada; University of Western Ontario, Canada

## Abstract

DNA barcoding of aquatic macroinvertebrates holds much promise as a tool for taxonomic research and for providing the reliable identifications needed for water quality assessment programs. A prerequisite for identification using barcodes is a reliable reference library. We gathered 4165 sequences from the barcode region of the mitochondrial cytochrome *c* oxidase subunit I gene representing 264 nominal and 90 provisional species of mayflies (Insecta: Ephemeroptera) from Canada, Mexico, and the United States. No species shared barcode sequences and all can be identified with barcodes with the possible exception of some *Caenis*. Minimum interspecific distances ranged from 0.3–24.7% (mean: 12.5%), while the average intraspecific divergence was 1.97%. The latter value was inflated by the presence of very high divergences in some taxa. In fact, nearly 20% of the species included two or three haplotype clusters showing greater than 5.0% sequence divergence and some values are as high as 26.7%. Many of the species with high divergences are polyphyletic and likely represent species complexes. Indeed, many of these polyphyletic species have numerous synonyms and individuals in some barcode clusters show morphological attributes characteristic of the synonymized species. In light of our findings, it is imperative that type or topotype specimens be sequenced to correctly associate barcode clusters with morphological species concepts and to determine the status of currently synonymized species.

## Introduction

DNA barcoding [1] of animals, the analysis of a standardized segment of the mitochondrial cytochrome *c* oxidase subunit 1 (COI) gene, has rapidly become an important tool for the identification, delimitation, and discovery of species [1]–[3]. DNA barcoding has the further advantage that identification success extends across all life stages, allowing the association of immatures with adults [4], [5]. Its capacity to identify all life stages is particularly important for aquatic ecology and biological monitoring (biomonitoring) of water quality because the aquatic larvae are usually the life stage studied [6]. Unfortunately, this is the life stage that is the most poorly known taxonomically because most species concepts in aquatic insects are based on the morphology of adult males. The identification of larvae is further hindered by the fact that many are rather delicate, especially mayflies (Ephemeroptera), and the structures critical for confident identifications such as gills, legs, and caudal filaments are commonly damaged or missing.

The application of DNA barcoding to freshwater biomonitoring has recently generated much interest for several reasons [7]–[12]. In addition to allowing the identification of difficult specimens, barcoding provides a level of data standardization that has been previously lacking in environmental assessments [13], aiding broader comparisons of results gathered through monitoring programs. Furthermore, barcoding reliably produces species-level (or even population-level) identifications that can improve the sensitivity of analyses, depending on the aims of the assessment program [14], [15].

An essential requirement of biomonitoring, conservation biology, and ecology is a sound taxonomy of the study organisms. Although taxa could be based purely on operational taxonomic units (OTUs) defined by sequence data, information on key functional niche traits (e.g. [16]; functional feeding groups, life history data, behavior and historical distribution) and environmental sensitivity [17] requires linking molecular OTUs with known taxa.

Mayflies are abundant in most aquatic habits, and show varied tolerance to differing disturbance regimes, making them ideal candidates for monitoring water quality. Together with the caddisflies (Trichoptera) and stoneflies (Plecoptera), they comprise one of the most commonly used biomonitoring metrics, EPT richness [18], and as such are one of the focal groups for the International Barcode of Life Freshwater Biosurveillance Working Group (www.ibol.org).

The development of a barcode reference library for North American mayflies was initiated by sequencing 150 specimens from 80 North American species [2]. Those results indicated that all species tested could be identified accurately using barcodes, but revealed several cases of deep sequence divergence within a species. Further species were added [19] and barcoding techniques used as a taxonomic tool to confirm the validity of a presumed new species and to support the synonymy of two species of *Heptagenia* (Heptageniidae). Additional barcodes for North American mayflies were generated from a regional inventory of northeastern Manitoba [20], [21], from a test of the efficacy of barcoding for biological monitoring using aquatic macroinvertebrates [12], and a general barcoding paper [1]. Additional barcode sequences for North American species of *Ephemerella* have been generated [22], but because these sequences are not publicly available, we do not consider them further.

The mayfly fauna of North America includes 651species (Mayfly Central, http://www.entm.purdue.edu/mayfly/na-species-list.php accessed 30 November 2011), but 10 of these taxa are *nomina dubia* and four are recently extinct. Ignoring the latter two groups, 637 species and 8 subspecies remain. Previous papers have assembled barcodes for 121 of these species, most from eastern North America [1], [2], [12], [19], [20]. In this paper, we broaden geographic coverage in North America (including Mexico) and raise barcode coverage to more than 350 taxa. Aside from reporting this progress, we provide guidance for further barcoding efforts on this group and highlight taxonomic problems in the North American Ephemeroptera.

## Results

**Figure 1:**
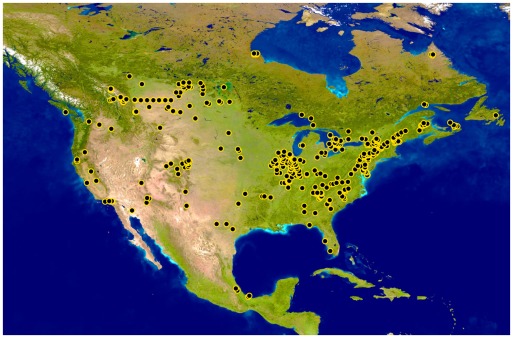
Distribution map for all barcoded specimens of Ephemeroptera with geocoordinates (n = 3902).

Although most specimens derived from east of the Rocky Mountains, collections were made across North America (Figure 1). Sequences (average length = 646 bp, ranging from 314–658 bp) were obtained from 4165 specimens; 3024 are newly analyzed and 1141 derive from prior studies. These sequences provide coverage for 71 of 106 genera and 18 of 21 families known from North America. In addition, these records provide coverage for 264 of the 647 species known from North America and for another 90 provisional species (Table 1). Because many of these provisional species were only represented by subimagos, females, or larvae that cannot be identified to species using morphological characters, many probably represent named species for which we have no sequences from adult males. As a consequence, as much as 55% of the North American fauna may have coverage, and perhaps as much as 60% of the 583 species known from Canada and the United States. Most of these taxa (284/354) were represented by more than one specimen (mean: 11.8, maximum: 236) (Table S1).

**Table 1:** Summary of current barcode coverage and divergence values for the 21 families of Ephemeroptera known from North America (NA).

Family	# known NAspecies andsubspecies	# barcodedspecies	# barcodedprovisionalspecies	# species withMXID ≥2.2%	Mean MXID	Mean MNID
Acanthametropodidae	2	0	0	0	–	–
Ameletidae	35	20	1	4	1.2	13.6
Ametropodidae	2	1	0	0	0.2	–
Baetidae	146	58	37	32	6	15.3
Baetiscidae	11	4	0	2	2.8	9.3
Behningiidae	1	1	0	1	7.9	–
Caenidae	36	12	1	6	8	11.6
Ephemerellidae	72	48	13	21	4.7	11.7
Ephemeridae	13	9	0	3	3.3	8.2
Euthyplociidae	1	0	0	–	–	–
Heptageniidae	130	56	21	19	2.8	10.2
Isonychiidae	15	7	4	2	1.5	13.3
Leptohyphidae	35	6	8	2	2.4	14.1
Leptophlebiidae	88	24	10	9	3.4	14.1
Metretopodidae	9	2	0	1	5.1	14.3
Neoephemeridae	4	1	0	0	0.8	–
Oligoneuriidae	8	0	0	–	–	–
Palingeniidae	1	1	0	0	1.6	–
Polymitarcyidae	7	2	0	1	4.8	6.3
Potamanthidae	5	1	0	0	0.2	–
Siphlonuridae	24	11	5	1	1.8	9.4

All distances are % K2P; MNID = minimum interspecific K2P distance, MXID = maximum intraspecific K2P distance.

No species shared haplotypes but previously published sequences for nine currently valid species clustered with specimens assigned to a different taxon. Whenever we were able to reexamine specimens, the original morphological identification proved incorrect. Table S2 summarizes these identification updates which have now been implemented on GenBank and BOLD (Barcode of Life Data Systems www.boldsystems.org).

The average maximum intraspecific divergence was 3.9% (max = 26.7%) and the average intraspecific divergence was 1.97%. Minimum interspecific distances ranged from 0.3–24.7% (mean: 12.5%). One hundred five species (29.7%) had maximum intraspecific divergences greater than 2.2%, a level of divergence found to delimit species across diverse groups of insects [1], [2], [20] although higher maximum intraspecific distances have been observed in Trichoptera when widely separated geographic areas are included [3]. Almost 20% of species with more than one specimen had maximum intraspecific divergences >5.0%; these species with high intraspecific divergences may represent species-complexes and when they are excluded from the analysis, the mean maximum intraspecific divergence decreased to 1.3%. In 44 species (12.5%), the maximum intraspecific distance was greater than the minimum interspecific distance. A Neighbor Joining tree of all specimens is available in Figure S1.

Increasing the geographic range between samples did not always lead to large increases in intraspecific divergence. For example, *Epeorus vitreus* (Walker) was sampled from throughout its latitudinal range but the maximum intraspecific divergence was only 2.8% and a specimen of *Ephemera simulans* Walker from Colorado differed by only a single nucleotide (0.15%) from a specimen from Churchill, Manitoba, a distance of over 2,200 km. Even in species with multiple barcode clusters, geographically distant specimens often clustered within the same group and specimens with small geographic distances sometimes occurred in different barcode clusters. The cluster of *Acentrella parvula* (McDunnough) that included a topotypical (from type locality) specimen from southern Ontario, for example, also included specimens from Saskatchewan and New Brunswick, but other specimens of *A. parvula* from New Brunswick formed a separate cluster together with specimens from New York.

### Barcode Divergences Between Species

Among the morphologically distinct species *Siphlonurus rapidus* McDunnough, *S. typicus* (Eaton), and *S*. sp.JMW1 the minimum divergence between species was 1.3–1.6%, but each species was monophyletic and so can be distinguished by barcode sequences using a tree-based criterion. Furthermore, each of the species can be identified by 2–6 fixed nucleotide differences (Table 2). Among *Caenis amica* Hagen, *C. punctata* McDunnough, and *C. youngi* Roemhild, the interspecific distances were as low as 0.3% (maximum intraspecific distances ranged from 3.7–21.9% and none of the species were monophyletic), possibly an artifact of incomplete taxonomic knowledge or historical introgression. For nearly all other species, the minimum interspecific distances were much greater (mean: 12.5%).

**Table 2:** Diagnostic nucleotides in *Siphlonurus rapidus*, *S. typicus*, and *S*. sp.JMW1.

Species	Diagnostic Nucleotides (position:nucleotide)
*Siphlonurus rapidus*	364:T, 502:C
*Siphlonurus typicus*	208:A, 232:C, 574:A
*Siphlonurus* sp.JMW1	79:A, 172:C, 397:T, 433:A, 541:A, 553:A

All but 9 of the 44 species with a maximum intraspecific divergence greater than the minimum interspecific divergence were polyphyletic or paraphyletic. *Centroptilum triangulifer* (McDunnough), *C. minor* (McDunnough), *Ephoron album* (Say), *Maccaffertium mexicanum integrum* (McDunnough), *Siphlonurus rapidus*, *Stenonema femoratum* (Say), *Eurylophella funeralis* (McDunnough), *Ephemerella dorothea infrequens* McDunnough, and *Teloganopsis deficiens* (Morgan) were all monophyletic with a maximum intraspecific divergence greater than the minimum interspecific divergence. Some of these species are clearly species complexes, with well-defined and deeply divergent clusters with associated morphological differences (i.e. *Maccaffertium mexicanum integrum*). The non-monophyletic species are also likely species complexes as preliminary examination of some, such as *Baetis tricaudatus* Dodds, show morphological variation corresponding to barcode clusters. Most of the species with very large intraspecific distances have at least one synonym. Further discussion of species with high intraspecific divergence and/or polyphyletic haplotype groups is available in Text S1.

### New Distribution and Life Stage Records

New distribution records were discovered for *Caenis anceps* McDunnough, *Maccaffertium smithae* (Traver), *Nixe joernensis* (Bengtsson), *Acentrella nadineae* McCafferty, Waltz & Webb, *Acentrella lapponica* Bengtsson, *Isonychia rufa* McDunnough and *Baetis foemina* Ide (Tables S1, S3). New life stage associations include the larvae of *Baetis persecutor* McDunnough, *Procloeon fragile* (McDunnough) and *Isonychia berneri* Kondratieff & Voshell; descriptions of these life stages are beyond the scope of this paper and will be treated elsewhere.

## Discussion

This paper presents the largest barcoding dataset of any order of aquatic insects, providing records for more than 4000 individuals from over 350 species. The analysis of both larvae and adults from several localities and some topotypes enabled identification with less ambiguity than in the past. This allowed the correction of previously misidentified species and strengthened knowledge of the levels of COI variation both within and between species. Our results confirm that levels of sequence divergence among closely allied species are generally high. For example, congeneric species of North American Ephemeroptera showed a mean barcode divergence of 13.9%, a much higher value than the 7–8% divergence reported for congeneric species of Lepidoptera in both North America [23] and Europe [24].

Our results indicate that much more taxonomic work is required on North American Ephemeroptera as many currently recognized species include several highly divergent, often polyphyletic, haplotypes, usually correlated with morphological differentiation among lineages. Most of these species have complex histories of synonymy, reflecting a 60 year trend in North American mayfly systematics towards inclusive species concepts. This trend was driven by the observation of individuals morphologically intermediate between named species, or by observations suggesting that members of one species fell within ‘the expected range of variation’ of another. Nearly all of these decisions of synonymy were based only on limited morphological studies, without consideration of biogeographic, ecological, behavioral or molecular data. While this fusion of species has simplified morphology-based identifications, it now seems likely that this trend often ignored biological reality. For example, the four species in the *Drunella lata* complex were all synonymized under *Drunella lata* (Morgan) based on morphological study [25], but morphometric, ecological, and allozyme data indicates at least three species in the northeastern United States [26]. Our results confirm this conclusion and amplify it by indicating that *D. longicornis* (Traver) likely also represents a valid species in the complex and that there is a fifth species in the southeastern United States (Figure S1).

Our results reveal that many species currently treated as synonyms may well represent valid species. A critical step in the resolution of this uncertainty lies in the acquisition of DNA barcodes from the holotype of each taxon as it represents the only unambiguously identified specimen. Because type specimens of North American Ephemeroptera have a mean age of 75 years (range 0–250 years), DNA degradation will be significant. However, prior work on Lepidoptera has shown that DNA barcode sequences can regularly be recovered from specimens that are less than a century old by assembling short amplicons [27], [28]. When sequences cannot be obtained from the holotype, topotypic specimens should be analyzed, an approach that we used for 19 of the species examined in this study. However, the analysis of type specimens is clearly preferable because many species descriptions lack detailed information on collection locality (e.g. state, province or sometimes just “North America”). When the type series has been destroyed, the designation of a barcoded neotype will provide a pathway to allow use of existing names and the description of new taxa [29]–[31]. For species with degraded types and ambiguous type locales, it may be useful to choose a specimen closely matching the morphological type concept as an informal “barcode type”.

While obtaining barcodes from type specimens will aid in the application of names to barcode clusters, further taxonomic work integrating multiple lines of evidence may be required to test current species hypotheses. For example, our results show that additional examination of the North American Baetidae and Ephemerellidae is required as they both contain large numbers of species with multiple barcode clusters, high intraspecific morphological variation, and many interim species identifications. DNA barcoding results cannot only serve as a guide of where to focus these efforts, but also be used as part of an iterative revisionary process [32] together with morphological, ecological, and behavioral characteristics to achieve stable, robust species hypotheses. Such refined species hypotheses will greatly improve our ability to determine and communicate the ecological characteristics of a species, such as phenology and tolerance to pollutants, and in turn improve our ability to monitor ecological changes. This can most effectively be achieved by involving taxonomists not only in the identification of specimens for barcoding, but also in the design of barcoding projects and the selection and collection of specimens for further analysis [33].

This study has increased barcode coverage for North American Ephemeroptera from 121 to 354 species, or over 50% of the known fauna. Because the present library includes records for nearly all common species, most Ephemeroptera taken in routine biomonitoring samples may now be rapidly and accurately identified through DNA barcoding, albeit with the proviso that further taxonomic work is needed to clarify species boundaries. Our progress in constructing a barcode library for North American Ephemeroptera further indicates the feasibility of gaining global coverage both for this order and for other key groups of aquatic insects.

## Materials and Methods

### Specimen Collection and Sequencing

Detailed collection data are included in Table S3, and are also available on BOLD (www.boldsystems.org) in the Virtual Project - Ephemeroptera (mayflies) of North America - Phase I (DATASET-EPNA1). Specimens were identified following currently accepted taxonomy at Mayfly Central (http://www.entm.purdue.edu/mayfly/na-species-list.php), except the Arthropleidae was included in the Heptageniidae because of recent molecular and integrated phylogenies [34], [35]. When discrepancies were detected between barcode results and morphological identifications, specimens were reexamined. When specimens could not be identified morphologically (e.g. certain females, some larvae), they were assigned the same name as expertly identified specimens with either the same or a closely similar (<2% divergence) COI haplotype. Specimen(s) which formed a unique barcode cluster, but which could not be identified morphologically, were assigned a provisional name structured in a consistent fashion (generic name followed by a species name composed of ‘sp.’ followed by initials of the taxonomist e.g. *Heptagenia* sp.LJ1; exceptions include previously published provisional names e.g. *Acerpenna* sp. CHU1 and those from California identified as part of an ongoing Southern California Coastal Water Research Project e.g. *Drunella* sp.CA1).

Topotypes, specimens collected from the type locality and which have a high certainty of representing the type concept of a species, were sequenced for the following species: *Acentrella parvula*, *Ameletus amador* Mayo, *A. andersoni* Zloty, *A. bellulus* Zloty, *A. pritchardi* Zloty, *Baetis adonis* Traver, *Caenis eglinensis* Pescador & Richard, *Cercobrachys cree* Sun, Webb & McCafferty, *Drunella grandis* (Eaton), *Epeorus albertae* (McDunnough), *Ephemerella dorothea infrequens*, *Eurylophella doris* (Traver), *E. oviruptis* Funk, *E. poconoensis* Funk and *Paraleptophlebia kirchneri* Kondratieff & Durfee. Individuals from near the type locality were sequenced for *Baetodes tritus* Cohen & Allen, *Camelobaetidius trivialis* Allen & Chao [currently considered a synonym of *C. warreni* (Traver & Edmunds)], *Heptagenia julia* Traver, and *Susperatus prudens* (McDunnough).

Each barcode sequence (>400 bp) for North American Ephemeroptera from previous publications (150 - [2], 13 - [19], 1 -[1], 414 - [12], 564 - [20]) were assembled and their source specimen was reexamined, whenever possible, to confirm its identification. In addition, barcodes were generated for another 3024 specimens at the Canadian Centre for DNA Barcoding using standard protocols for DNA extraction, polymerase chain reaction (PCR) and sequencing [36], [37]. PCR was performed using the standard LCO1490/HCO2198 and/or LepF1/LepR1 primers with M13 tails. When these protocols failed to recover a sequence, the primer set LCO1490 and MEPTR1-t1 was used to recover a 325 bp segment from the 5′ end of the barcode region [20].

Sequences were obtained from the following Barcode of Life Data systems (BOLD systems: http://www.boldsystems.org) project codes: ELPYO, BBEPT, CPMAY, ECEPH, FAMAY, SMMAY, INHSE, LJMAY, LJGSM, BKMAY, ABMAY, MBMAY, NYMAY, ONMAY, SKMAY, USMAY, PUMAY, CFWIA, CFWIB, CFWIC, CFWID, CFWIE, CFWIF, CFWIG, CFWIH, CFWII, SWRCE, SWRCD, PRESV, GSEPT, SWAMI, WEAI, SBEP, NBMAY, HIEPT, HIMXD.

COI sequences were aligned in MEGA 5 [38] using the integrated ClustalX method with default parameters. All sequences were examined for the presence of stop codons and indels. Kimura-2-Parameter (K2P) distances were calculated in MEGA5 using the pairwise deletion option and a Neighbor Joining tree was generated.

## Supporting Information

Figure S1 Neighbour Joining tree using Kimura-2-Parameter distance for COI DNA sequences from 4065 individuals of North American Ephemeroptera. Specimens and species with topotypes are indicated with ‘*’; specimens and species from the same general area as the type locality are indicated with ‘#’.
(PDF)

Table S1 Species-level summary of K2P distance, sample distribution, and sample size for North American Ephemeroptera. All distributional records use standard 2-letter (or 3-letter, for Mexico) postal abbreviations. MNID = minimum interspecific K2P distance, MXID = maximum intraspecific K2P distance, species with maximum intraspecific sequence divergence ≥5.0% are indicated with ‘*’.
(DOC)

Table S2 Updated identifications for Ephemeroptera specimens with published barcode records.
(DOC)

Table S3 Collection data for all barcoded specimens of Ephemeroptera.
(XLS)

Text S1 Taxonomic notes on selected North American Ephemeroptera species with barcode records.
(DOC)
