# Supplementary material for: A DNA Barcode Library for North American Ephemeroptera: Progress and Prospects
Source: PLoS One. 2012 May 30;7(5):e38063. doi: 10.1371/journal.pone.0038063 (PMC3364165; doi:10.1371/journal.pone.0038063)
Supplement: Figure S1 — Neighbour Joining tree using Kimura-2-Parameter distance for COI DNA sequences from 4065 individuals of North American Ephemeroptera. Specimens and species with topotypes are indicated with ‘*’; specimens and species from the same general area as the type locality are indicated with ‘#’. (PDF) [file pone.0038063.s001.pdf]

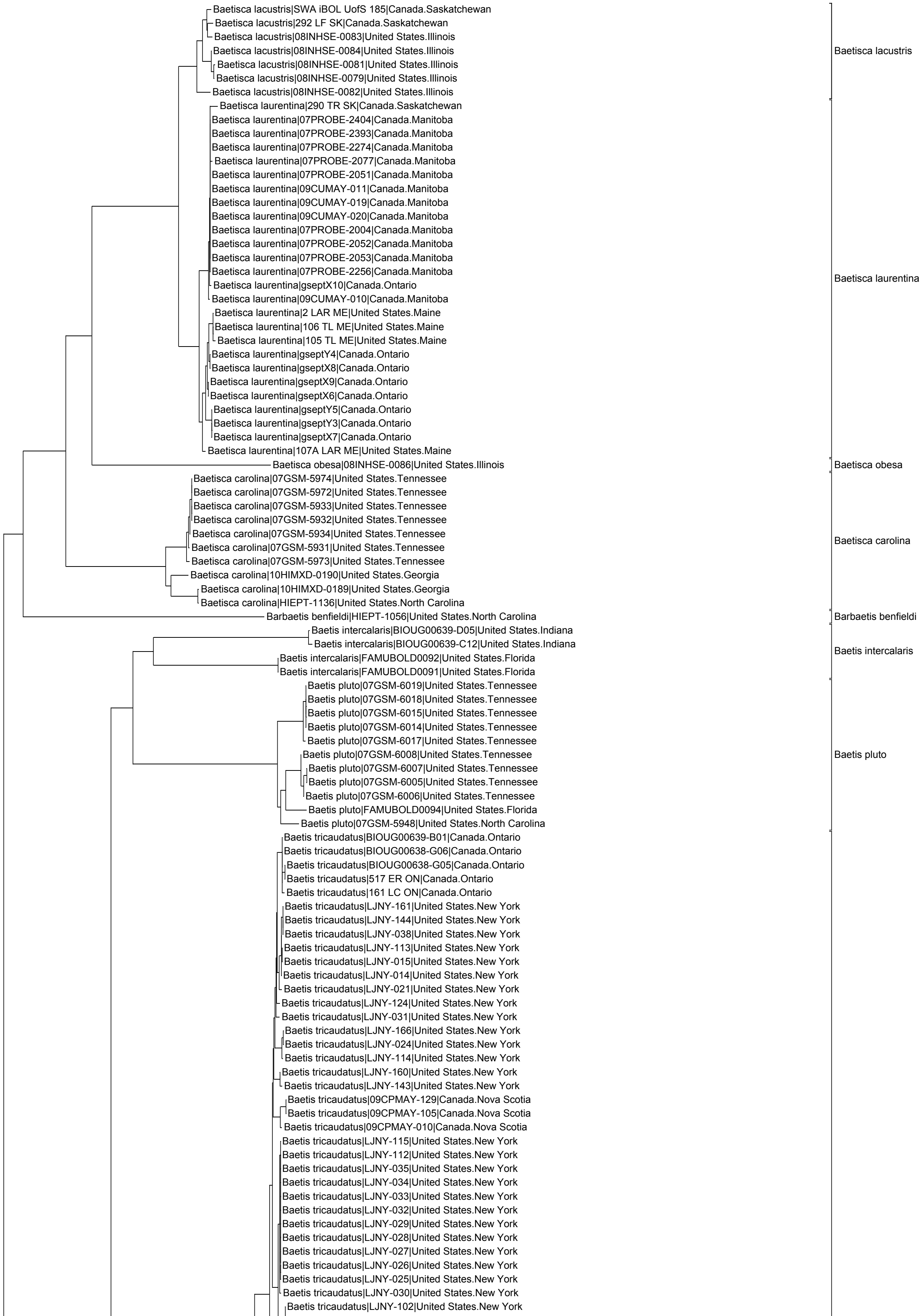

Baetisca lacustris|SWA iBOL UofS 185|Canada.Saskatchewan
Baetisca lacustris|292 LF SK|Canada.Saskatchewan
Baetisca lacustris|08INHSE-0083|United States.Illinois
Baetisca lacustris|08INHSE-0084|United States.Illinois
Baetisca lacustris|08INHSE-0081|United States.Illinois
Baetisca lacustris|08INHSE-0079|United States.Illinois
Baetisca lacustris|08INHSE-0082|United States.Illinois
Baetisca laurentina|290 TR SK|Canada.Saskatchewan
Baetisca laurentina|07PROBE-2404|Canada.Manitoba
Baetisca laurentina|07PROBE-2393|Canada.Manitoba
Baetisca laurentina|07PROBE-2274|Canada.Manitoba
Baetisca laurentina|07PROBE-2077|Canada.Manitoba
Baetisca laurentina|07PROBE-2051|Canada.Manitoba
Baetisca laurentina|09CUMAY-011|Canada.Manitoba
Baetisca laurentina|09CUMAY-019|Canada.Manitoba
Baetisca laurentina|09CUMAY-020|Canada.Manitoba
Baetisca laurentina|07PROBE-2004|Canada.Manitoba
Baetisca laurentina|07PROBE-2052|Canada.Manitoba
Baetisca laurentina|07PROBE-2053|Canada.Manitoba
Baetisca laurentina|07PROBE-2256|Canada.Manitoba
Baetisca laurentina|gseptX10|Canada.Ontario
Baetisca laurentina|09CUMAY-010|Canada.Manitoba
Baetisca laurentina|2 LAR ME|United States.Maine
Baetisca laurentina|106 TL ME|United States.Maine
Baetisca laurentina|105 TL ME|United States.Maine
Baetisca laurentina|gseptY4|Canada.Ontario
Baetisca laurentina|gseptX8|Canada.Ontario
Baetisca laurentina|gseptX9|Canada.Ontario
Baetisca laurentina|gseptX6|Canada.Ontario
Baetisca laurentina|gseptY5|Canada.Ontario
Baetisca laurentina|gseptY3|Canada.Ontario
Baetisca laurentina|gseptX7|Canada.Ontario
Baetisca laurentina|107A LAR ME|United States.Maine
Baetisca obesa|08INHSE-0086|United States.Illinois
Baetisca carolina|07GSM-5974|United States.Tennessee
Baetisca carolina|07GSM-5972|United States.Tennessee
Baetisca carolina|07GSM-5933|United States.Tennessee
Baetisca carolina|07GSM-5932|United States.Tennessee
Baetisca carolina|07GSM-5934|United States.Tennessee
Baetisca carolina|07GSM-5931|United States.Tennessee
Baetisca carolina|07GSM-5973|United States.Tennessee
Baetisca carolina|10HIMXD-0190|United States.Georgia
Baetisca carolina|10HIMXD-0189|United States.Georgia
Baetisca carolina|HIEPT-1136|United States.North Carolina
Barbaetis benfieldi|HIEPT-1056|United States.North Carolina
Baetis intercalaris|BIOUG00639-D05|United States.Indiana
Baetis intercalaris|BIOUG00639-C12|United States.Indiana
Baetis intercalaris|FAMUBOLD0092|United States.Florida
Baetis intercalaris|FAMUBOLD0091|United States.Florida
Baetis pluto|07GSM-6019|United States.Tennessee
Baetis pluto|07GSM-6018|United States.Tennessee
Baetis pluto|07GSM-6015|United States.Tennessee
Baetis pluto|07GSM-6014|United States.Tennessee
Baetis pluto|07GSM-6017|United States.Tennessee
Baetis pluto|07GSM-6008|United States.Tennessee
Baetis pluto|07GSM-6007|United States.Tennessee
Baetis pluto|07GSM-6005|United States.Tennessee
Baetis pluto|07GSM-6006|United States.Tennessee
Baetis pluto|FAMUBOLD0094|United States.Florida
Baetis pluto|07GSM-5948|United States.North Carolina
Baetis tricaudatus|BIOUG00639-B01|Canada.Ontario
Baetis tricaudatus|BIOUG00638-G06|Canada.Ontario
Baetis tricaudatus|BIOUG00638-G05|Canada.Ontario
Baetis tricaudatus|517 ER ON|Canada.Ontario
Baetis tricaudatus|161 LC ON|Canada.Ontario
Baetis tricaudatus|LJNY-161|United States.New York
Baetis tricaudatus|LJNY-144|United States.New York
Baetis tricaudatus|LJNY-038|United States.New York
Baetis tricaudatus|LJNY-113|United States.New York
Baetis tricaudatus|LJNY-015|United States.New York
Baetis tricaudatus|LJNY-014|United States.New York
Baetis tricaudatus|LJNY-021|United States.New York
Baetis tricaudatus|LJNY-124|United States.New York
Baetis tricaudatus|LJNY-031|United States.New York
Baetis tricaudatus|LJNY-166|United States.New York
Baetis tricaudatus|LJNY-024|United States.New York
Baetis tricaudatus|LJNY-114|United States.New York
Baetis tricaudatus|LJNY-160|United States.New York
Baetis tricaudatus|LJNY-143|United States.New York
Baetis tricaudatus|09CPMAY-129|Canada.Nova Scotia
Baetis tricaudatus|09CPMAY-105|Canada.Nova Scotia
Baetis tricaudatus|09CPMAY-010|Canada.Nova Scotia
Baetis tricaudatus|LJNY-115|United States.New York
Baetis tricaudatus|LJNY-112|United States.New York
Baetis tricaudatus|LJNY-035|United States.New York
Baetis tricaudatus|LJNY-034|United States.New York
Baetis tricaudatus|LJNY-033|United States.New York
Baetis tricaudatus|LJNY-032|United States.New York
Baetis tricaudatus|LJNY-029|United States.New York
Baetis tricaudatus|LJNY-028|United States.New York
Baetis tricaudatus|LJNY-027|United States.New York
Baetis tricaudatus|LJNY-026|United States.New York
Baetis tricaudatus|LJNY-025|United States.New York
Baetis tricaudatus|LJNY-030|United States.New York
Baetis tricaudatus|LJNY-102|United States.New York
Baetisca lacustris
Baetisca laurentina
Baetisca obesa
Baetisca carolina
Barbaetis benfieldi
Baetis intercalaris
Baetis pluto

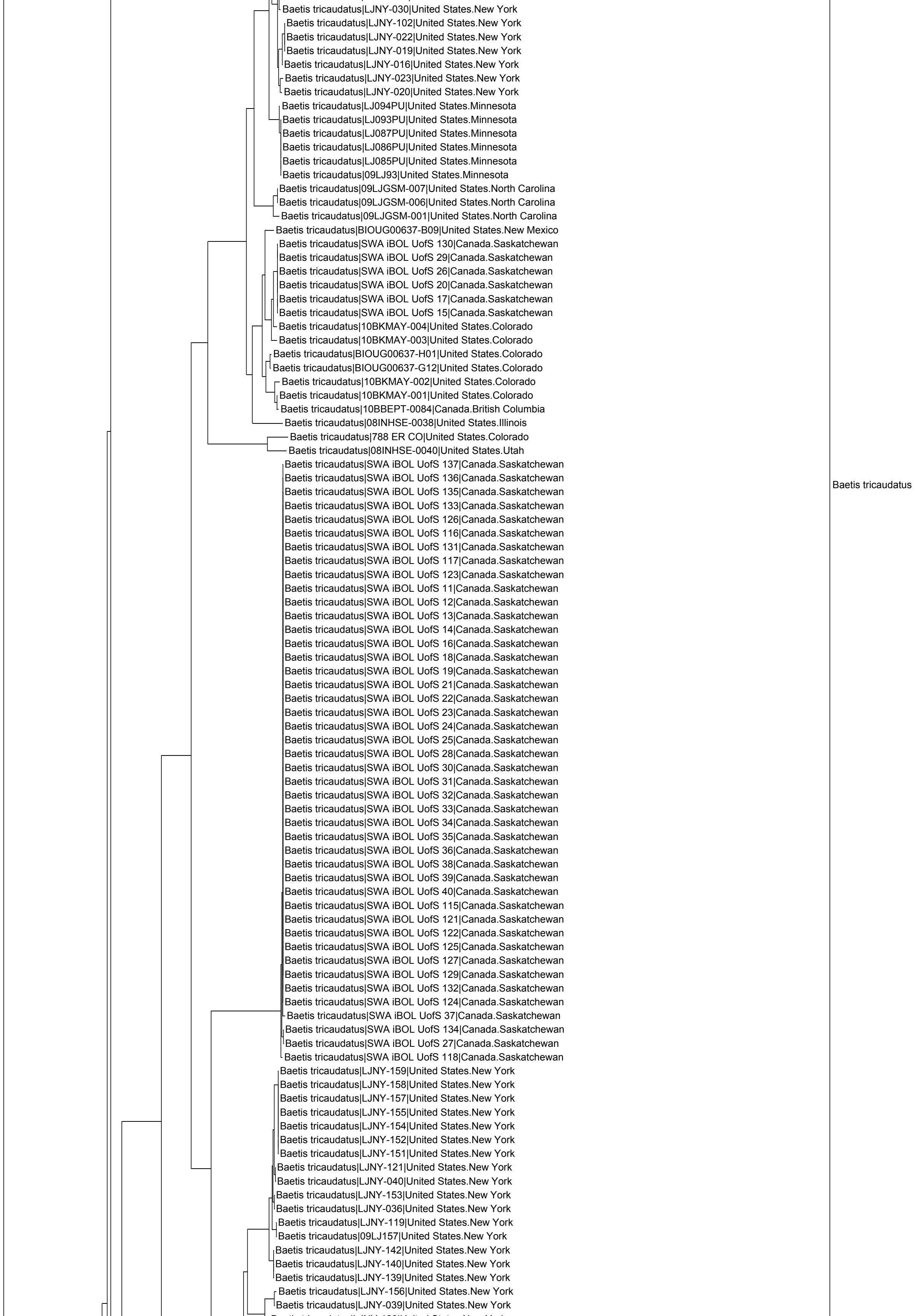
Baetis tricaudatus|LJNY-030|United States.New York
Baetis tricaudatus|LJNY-102|United States.New York
Baetis tricaudatus|LJNY-022|United States.New York
Baetis tricaudatus|LJNY-019|United States.New York
Baetis tricaudatus|LJNY-016|United States.New York
Baetis tricaudatus|LJNY-023|United States.New York
Baetis tricaudatus|LJNY-020|United States.New York
Baetis tricaudatus|LJ094PU|United States.Minnesota
Baetis tricaudatus|LJ093PU|United States.Minnesota
Baetis tricaudatus|LJ087PU|United States.Minnesota
Baetis tricaudatus|LJ086PU|United States.Minnesota
Baetis tricaudatus|LJ085PU|United States.Minnesota
Baetis tricaudatus|09LJ93|United States.Minnesota
Baetis tricaudatus|09LJGSM-007|United States.North Carolina
Baetis tricaudatus|09LJGSM-006|United States.North Carolina
Baetis tricaudatus|09LJGSM-001|United States.North Carolina
Baetis tricaudatus|BIOUG00637-B09|United States.New Mexico
Baetis tricaudatus|SWA iBOL UofS 130|Canada.Saskatchewan
Baetis tricaudatus|SWA iBOL UofS 29|Canada.Saskatchewan
Baetis tricaudatus|SWA iBOL UofS 26|Canada.Saskatchewan
Baetis tricaudatus|SWA iBOL UofS 20|Canada.Saskatchewan
Baetis tricaudatus|SWA iBOL UofS 17|Canada.Saskatchewan
Baetis tricaudatus|SWA iBOL UofS 15|Canada.Saskatchewan
Baetis tricaudatus|10BKMAY-004|United States.Colorado
Baetis tricaudatus|10BKMAY-003|United States.Colorado
Baetis tricaudatus|BIOUG00637-H01|United States.Colorado
Baetis tricaudatus|BIOUG00637-G12|United States.Colorado
Baetis tricaudatus|10BKMAY-002|United States.Colorado
Baetis tricaudatus|10BKMAY-001|United States.Colorado
Baetis tricaudatus|10BBEPT-0084|Canada.British Columbia
Baetis tricaudatus|08INHSE-0038|United States.Illinois
Baetis tricaudatus|788 ER CO|United States.Colorado
Baetis tricaudatus|08INHSE-0040|United States.Utah
Baetis tricaudatus|SWA iBOL UofS 137|Canada.Saskatchewan
Baetis tricaudatus|SWA iBOL UofS 136|Canada.Saskatchewan
Baetis tricaudatus|SWA iBOL UofS 135|Canada.Saskatchewan
Baetis tricaudatus|SWA iBOL UofS 133|Canada.Saskatchewan
Baetis tricaudatus|SWA iBOL UofS 126|Canada.Saskatchewan
Baetis tricaudatus|SWA iBOL UofS 116|Canada.Saskatchewan
Baetis tricaudatus|SWA iBOL UofS 131|Canada.Saskatchewan
Baetis tricaudatus|SWA iBOL UofS 117|Canada.Saskatchewan
Baetis tricaudatus|SWA iBOL UofS 123|Canada.Saskatchewan
Baetis tricaudatus|SWA iBOL UofS 11|Canada.Saskatchewan
Baetis tricaudatus|SWA iBOL UofS 12|Canada.Saskatchewan
Baetis tricaudatus|SWA iBOL UofS 13|Canada.Saskatchewan
Baetis tricaudatus|SWA iBOL UofS 14|Canada.Saskatchewan
Baetis tricaudatus|SWA iBOL UofS 16|Canada.Saskatchewan
Baetis tricaudatus|SWA iBOL UofS 18|Canada.Saskatchewan
Baetis tricaudatus|SWA iBOL UofS 19|Canada.Saskatchewan
Baetis tricaudatus|SWA iBOL UofS 21|Canada.Saskatchewan
Baetis tricaudatus|SWA iBOL UofS 22|Canada.Saskatchewan
Baetis tricaudatus|SWA iBOL UofS 23|Canada.Saskatchewan
Baetis tricaudatus|SWA iBOL UofS 24|Canada.Saskatchewan
Baetis tricaudatus|SWA iBOL UofS 25|Canada.Saskatchewan
Baetis tricaudatus|SWA iBOL UofS 28|Canada.Saskatchewan
Baetis tricaudatus|SWA iBOL UofS 30|Canada.Saskatchewan
Baetis tricaudatus|SWA iBOL UofS 31|Canada.Saskatchewan
Baetis tricaudatus|SWA iBOL UofS 32|Canada.Saskatchewan
Baetis tricaudatus|SWA iBOL UofS 33|Canada.Saskatchewan
Baetis tricaudatus|SWA iBOL UofS 34|Canada.Saskatchewan
Baetis tricaudatus|SWA iBOL UofS 35|Canada.Saskatchewan
Baetis tricaudatus|SWA iBOL UofS 36|Canada.Saskatchewan
Baetis tricaudatus|SWA iBOL UofS 38|Canada.Saskatchewan
Baetis tricaudatus|SWA iBOL UofS 39|Canada.Saskatchewan
Baetis tricaudatus|SWA iBOL UofS 40|Canada.Saskatchewan
Baetis tricaudatus|SWA iBOL UofS 115|Canada.Saskatchewan
Baetis tricaudatus|SWA iBOL UofS 121|Canada.Saskatchewan
Baetis tricaudatus|SWA iBOL UofS 122|Canada.Saskatchewan
Baetis tricaudatus|SWA iBOL UofS 125|Canada.Saskatchewan
Baetis tricaudatus|SWA iBOL UofS 127|Canada.Saskatchewan
Baetis tricaudatus|SWA iBOL UofS 129|Canada.Saskatchewan
Baetis tricaudatus|SWA iBOL UofS 132|Canada.Saskatchewan
Baetis tricaudatus|SWA iBOL UofS 124|Canada.Saskatchewan
Baetis tricaudatus|SWA iBOL UofS 37|Canada.Saskatchewan
Baetis tricaudatus|SWA iBOL UofS 134|Canada.Saskatchewan
Baetis tricaudatus|SWA iBOL UofS 27|Canada.Saskatchewan
Baetis tricaudatus|SWA iBOL UofS 118|Canada.Saskatchewan
Baetis tricaudatus|LJNY-159|United States.New York
Baetis tricaudatus|LJNY-158|United States.New York
Baetis tricaudatus|LJNY-157|United States.New York
Baetis tricaudatus|LJNY-155|United States.New York
Baetis tricaudatus|LJNY-154|United States.New York
Baetis tricaudatus|LJNY-152|United States.New York
Baetis tricaudatus|LJNY-151|United States.New York
Baetis tricaudatus|LJNY-121|United States.New York
Baetis tricaudatus|LJNY-040|United States.New York
Baetis tricaudatus|LJNY-153|United States.New York
Baetis tricaudatus|LJNY-036|United States.New York
Baetis tricaudatus|LJNY-119|United States.New York
Baetis tricaudatus|09LJ157|United States.New York
Baetis tricaudatus|LJNY-142|United States.New York
Baetis tricaudatus|LJNY-140|United States.New York
Baetis tricaudatus|LJNY-139|United States.New York
Baetis tricaudatus|LJNY-156|United States.New York
Baetis tricaudatus|LJNY-039|United States.New York
Baetis tricaudatus

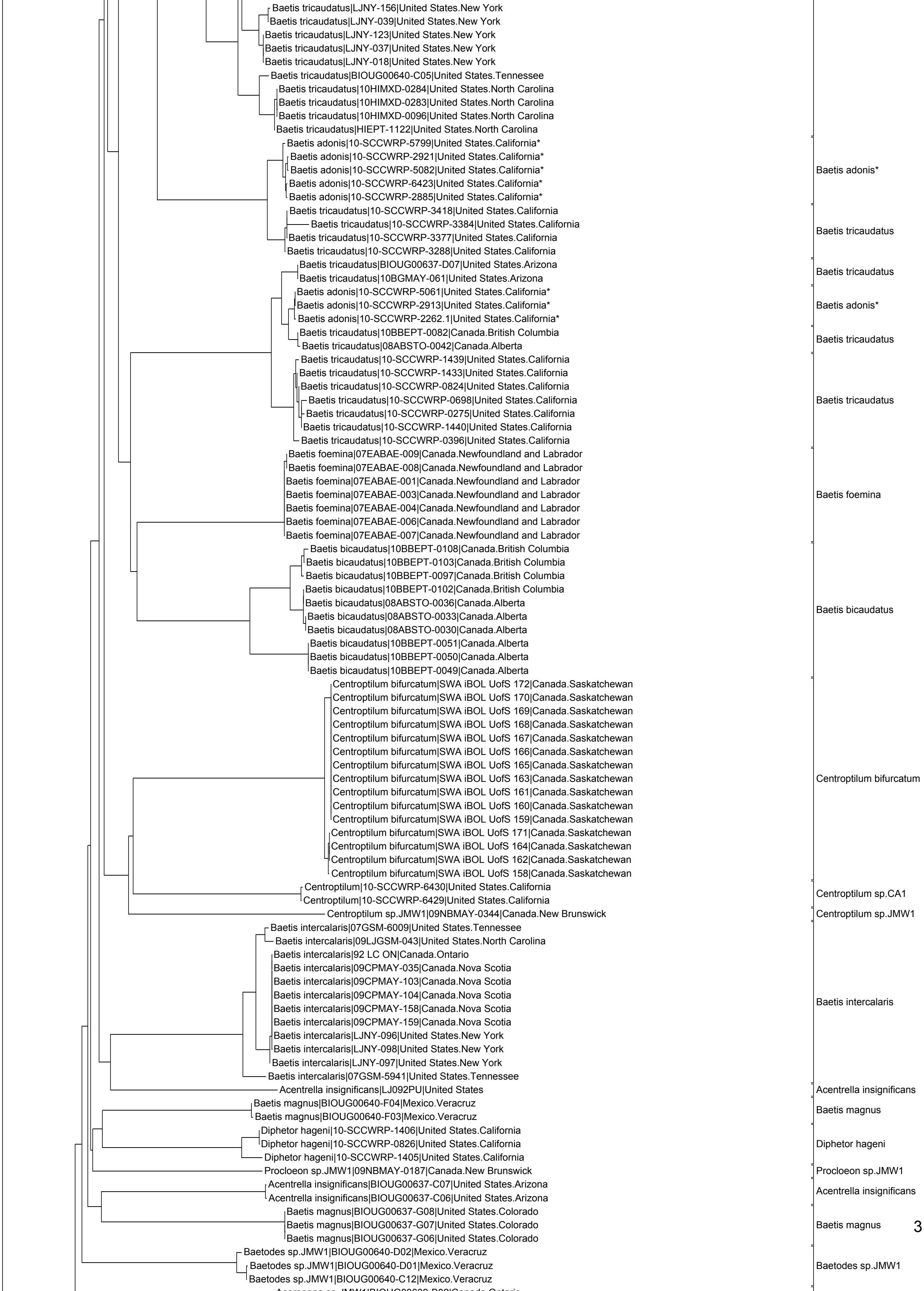
Baetis tricaudatus|LJNY-156|United States.New York
Baetis tricaudatus|LJNY-039|United States.New York
Baetis tricaudatus|LJNY-123|United States.New York
Baetis tricaudatus|LJNY-037|United States.New York
Baetis tricaudatus|LJNY-018|United States.New York
Baetis tricaudatus|BIOUG00640-C05|United States.Tennessee
Baetis tricaudatus|10HIMXD-0284|United States.North Carolina
Baetis tricaudatus|10HIMXD-0283|United States.North Carolina
Baetis tricaudatus|10HIMXD-0096|United States.North Carolina
Baetis tricaudatus|HIEPT-1122|United States.North Carolina
Baetis adonis|10-SCCWRP-5799|United States.California*
Baetis adonis|10-SCCWRP-2921|United States.California*
Baetis adonis|10-SCCWRP-5082|United States.California*
Baetis adonis|10-SCCWRP-6423|United States.California*
Baetis adonis|10-SCCWRP-2885|United States.California*
Baetis adonis*
Baetis tricaudatus|10-SCCWRP-3418|United States.California
Baetis tricaudatus|10-SCCWRP-3384|United States.California
Baetis tricaudatus|10-SCCWRP-3377|United States.California
Baetis tricaudatus|10-SCCWRP-3288|United States.California
Baetis tricaudatus
Baetis tricaudatus|BIOUG00637-D07|United States.Arizona
Baetis tricaudatus|10BGMAY-061|United States.Arizona
Baetis tricaudatus
Baetis adonis|10-SCCWRP-5061|United States.California*
Baetis adonis|10-SCCWRP-2913|United States.California*
Baetis adonis|10-SCCWRP-2262.1|United States.California*
Baetis adonis*
Baetis tricaudatus|10BBEPT-0082|Canada.British Columbia
Baetis tricaudatus|08ABSTO-0042|Canada.Alberta
Baetis tricaudatus
Baetis tricaudatus|10-SCCWRP-1439|United States.California
Baetis tricaudatus|10-SCCWRP-1433|United States.California
Baetis tricaudatus|10-SCCWRP-0824|United States.California
Baetis tricaudatus|10-SCCWRP-0698|United States.California
Baetis tricaudatus|10-SCCWRP-0275|United States.California
Baetis tricaudatus|10-SCCWRP-1440|United States.California
Baetis tricaudatus|10-SCCWRP-0396|United States.California
Baetis tricaudatus
Baetis foemina|07EABAE-009|Canada.Newfoundland and Labrador
Baetis foemina|07EABAE-008|Canada.Newfoundland and Labrador
Baetis foemina|07EABAE-001|Canada.Newfoundland and Labrador
Baetis foemina|07EABAE-003|Canada.Newfoundland and Labrador
Baetis foemina|07EABAE-004|Canada.Newfoundland and Labrador
Baetis foemina|07EABAE-006|Canada.Newfoundland and Labrador
Baetis foemina|07EABAE-007|Canada.Newfoundland and Labrador
Baetis foemina
Baetis bicaudatus|10BBEPT-0108|Canada.British Columbia
Baetis bicaudatus|10BBEPT-0103|Canada.British Columbia
Baetis bicaudatus|10BBEPT-0097|Canada.British Columbia
Baetis bicaudatus|10BBEPT-0102|Canada.British Columbia
Baetis bicaudatus|08ABSTO-0036|Canada.Alberta
Baetis bicaudatus|08ABSTO-0033|Canada.Alberta
Baetis bicaudatus|08ABSTO-0030|Canada.Alberta
Baetis bicaudatus|10BBEPT-0051|Canada.Alberta
Baetis bicaudatus|10BBEPT-0050|Canada.Alberta
Baetis bicaudatus|10BBEPT-0049|Canada.Alberta
Baetis bicaudatus
Centroptilum bifurcatum|SWA iBOL UofS 172|Canada.Saskatchewan
Centroptilum bifurcatum|SWA iBOL UofS 170|Canada.Saskatchewan
Centroptilum bifurcatum|SWA iBOL UofS 169|Canada.Saskatchewan
Centroptilum bifurcatum|SWA iBOL UofS 168|Canada.Saskatchewan
Centroptilum bifurcatum|SWA iBOL UofS 167|Canada.Saskatchewan
Centroptilum bifurcatum|SWA iBOL UofS 166|Canada.Saskatchewan
Centroptilum bifurcatum|SWA iBOL UofS 165|Canada.Saskatchewan
Centroptilum bifurcatum|SWA iBOL UofS 163|Canada.Saskatchewan
Centroptilum bifurcatum|SWA iBOL UofS 161|Canada.Saskatchewan
Centroptilum bifurcatum|SWA iBOL UofS 160|Canada.Saskatchewan
Centroptilum bifurcatum|SWA iBOL UofS 159|Canada.Saskatchewan
Centroptilum bifurcatum|SWA iBOL UofS 171|Canada.Saskatchewan
Centroptilum bifurcatum|SWA iBOL UofS 164|Canada.Saskatchewan
Centroptilum bifurcatum|SWA iBOL UofS 162|Canada.Saskatchewan
Centroptilum bifurcatum|SWA iBOL UofS 158|Canada.Saskatchewan
Centroptilum bifurcatum
Centroptilum|10-SCCWRP-6430|United States.California
Centroptilum|10-SCCWRP-6429|United States.California
Centroptilum sp.CA1
Centroptilum sp.JMW1|09NBMAY-0344|Canada.New Brunswick
Centroptilum sp.JMW1
Baetis intercalaris|07GSM-6009|United States.Tennessee
Baetis intercalaris|09LJGSM-043|United States.North Carolina
Baetis intercalaris|92 LC ON|Canada.Ontario
Baetis intercalaris|09CPMAY-035|Canada.Nova Scotia
Baetis intercalaris|09CPMAY-103|Canada.Nova Scotia
Baetis intercalaris|09CPMAY-104|Canada.Nova Scotia
Baetis intercalaris|09CPMAY-158|Canada.Nova Scotia
Baetis intercalaris|09CPMAY-159|Canada.Nova Scotia
Baetis intercalaris|LJNY-096|United States.New York
Baetis intercalaris|LJNY-098|United States.New York
Baetis intercalaris|LJNY-097|United States.New York
Baetis intercalaris|07GSM-5941|United States.Tennessee
Baetis intercalaris
Acentrella insignificans|LJ092PU|United States
Acentrella insignificans
Baetis magnus|BIOUG00640-F04|Mexico.Veracruz
Baetis magnus|BIOUG00640-F03|Mexico.Veracruz
Baetis magnus
Diphetor hageni|10-SCCWRP-1406|United States.California
Diphetor hageni|10-SCCWRP-0826|United States.California
Diphetor hageni|10-SCCWRP-1405|United States.California
Diphetor hageni
Procloeon sp.JMW1|09NBMAY-0187|Canada.New Brunswick
Procloeon sp.JMW1
Acentrella insignificans|BIOUG00637-C07|United States.Arizona
Acentrella insignificans|BIOUG00637-C06|United States.Arizona
Acentrella insignificans
Baetis magnus|BIOUG00637-G08|United States.Colorado
Baetis magnus|BIOUG00637-G07|United States.Colorado
Baetis magnus|BIOUG00637-G06|United States.Colorado
Baetis magnus
Baetodes sp.JMW1|BIOUG00640-D02|Mexico.Veracruz
Baetodes sp.JMW1|BIOUG00640-D01|Mexico.Veracruz
Baetodes sp.JMW1|BIOUG00640-C12|Mexico.Veracruz
Baetodes sp.JMW1

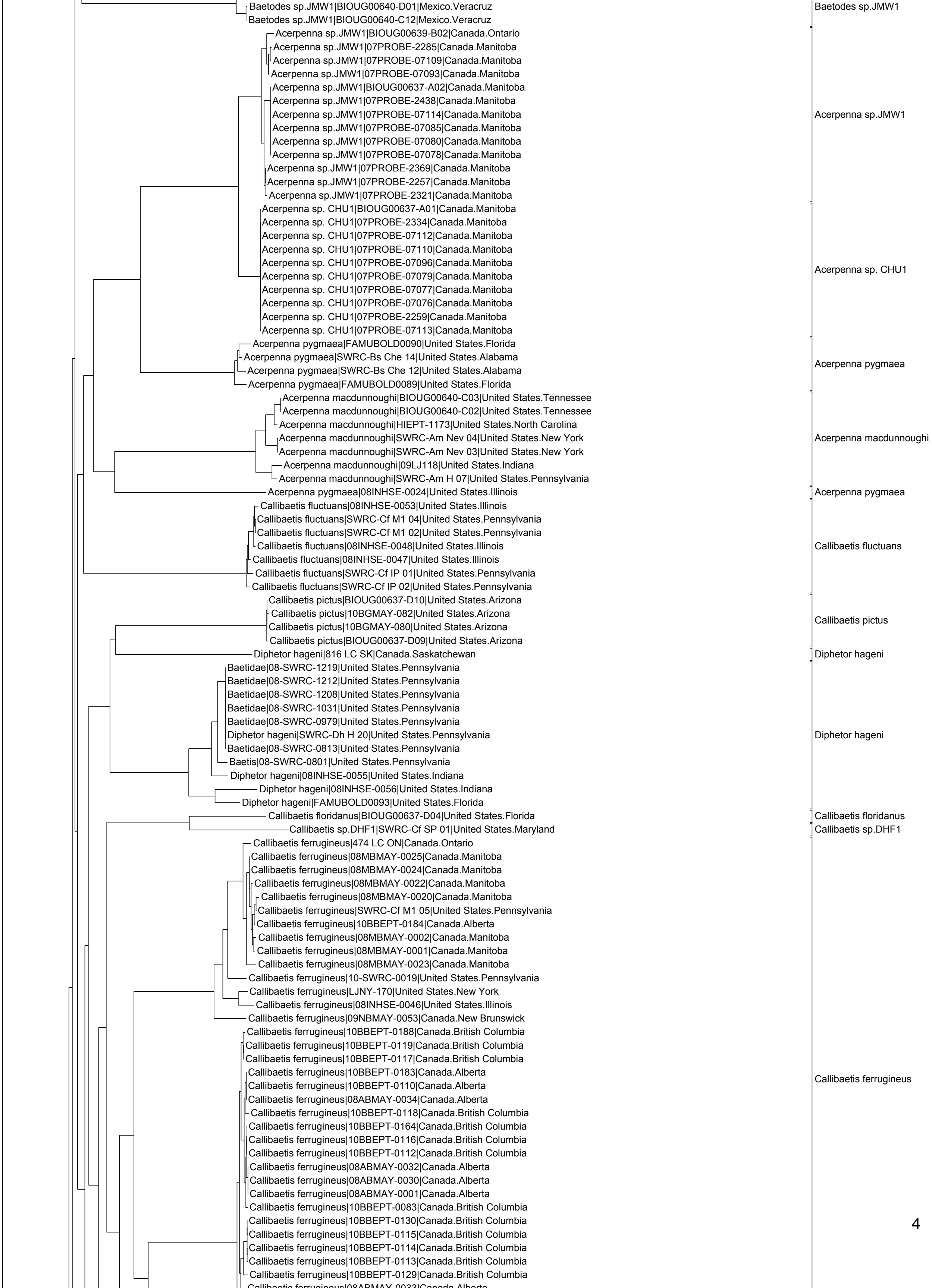
Baetodes sp.JMW1|BIOUG00640-D01|Mexico.Veracruz
Baetodes sp.JMW1|BIOUG00640-C12|Mexico.Veracruz
Acerpenna sp.JMW1|BIOUG00639-B02|Canada.Ontario
Acerpenna sp.JMW1|07PROBE-2285|Canada.Manitoba
Acerpenna sp.JMW1|07PROBE-07109|Canada.Manitoba
Acerpenna sp.JMW1|07PROBE-07093|Canada.Manitoba
Acerpenna sp.JMW1|BIOUG00637-A02|Canada.Manitoba
Acerpenna sp.JMW1|07PROBE-2438|Canada.Manitoba
Acerpenna sp.JMW1|07PROBE-07114|Canada.Manitoba
Acerpenna sp.JMW1|07PROBE-07085|Canada.Manitoba
Acerpenna sp.JMW1|07PROBE-07080|Canada.Manitoba
Acerpenna sp.JMW1|07PROBE-07078|Canada.Manitoba
Acerpenna sp.JMW1|07PROBE-2369|Canada.Manitoba
Acerpenna sp.JMW1|07PROBE-2257|Canada.Manitoba
Acerpenna sp.JMW1|07PROBE-2321|Canada.Manitoba
Acerpenna sp. CHU1|BIOUG00637-A01|Canada.Manitoba
Acerpenna sp. CHU1|07PROBE-2334|Canada.Manitoba
Acerpenna sp. CHU1|07PROBE-07112|Canada.Manitoba
Acerpenna sp. CHU1|07PROBE-07110|Canada.Manitoba
Acerpenna sp. CHU1|07PROBE-07096|Canada.Manitoba
Acerpenna sp. CHU1|07PROBE-07079|Canada.Manitoba
Acerpenna sp. CHU1|07PROBE-07077|Canada.Manitoba
Acerpenna sp. CHU1|07PROBE-07076|Canada.Manitoba
Acerpenna sp. CHU1|07PROBE-2259|Canada.Manitoba
Acerpenna sp. CHU1|07PROBE-07113|Canada.Manitoba
Acerpenna pygmaea|FAMUBOLD0090|United States.Florida
Acerpenna pygmaea|SWRC-Bs Che 14|United States.Alabama
Acerpenna pygmaea|SWRC-Bs Che 12|United States.Alabama
Acerpenna pygmaea|FAMUBOLD0089|United States.Florida
Acerpenna macdunnoughi|BIOUG00640-C03|United States.Tennessee
Acerpenna macdunnoughi|BIOUG00640-C02|United States.Tennessee
Acerpenna macdunnoughi|HIEPT-1173|United States.North Carolina
Acerpenna macdunnoughi|SWRC-Am Nev 04|United States.New York
Acerpenna macdunnoughi|SWRC-Am Nev 03|United States.New York
Acerpenna macdunnoughi|09LJ118|United States.Indiana
Acerpenna macdunnoughi|SWRC-Am H 07|United States.Pennsylvania
Acerpenna pygmaea|08INHSE-0024|United States.Illinois
Callibaetis fluctuans|08INHSE-0053|United States.Illinois
Callibaetis fluctuans|SWRC-Cf M1 04|United States.Pennsylvania
Callibaetis fluctuans|SWRC-Cf M1 02|United States.Pennsylvania
Callibaetis fluctuans|08INHSE-0048|United States.Illinois
Callibaetis fluctuans|08INHSE-0047|United States.Illinois
Callibaetis fluctuans|SWRC-Cf IP 01|United States.Pennsylvania
Callibaetis fluctuans|SWRC-Cf IP 02|United States.Pennsylvania
Callibaetis pictus|BIOUG00637-D10|United States.Arizona
Callibaetis pictus|10BGMAY-082|United States.Arizona
Callibaetis pictus|10BGMAY-080|United States.Arizona
Callibaetis pictus|BIOUG00637-D09|United States.Arizona
Diphetor hageni|816 LC SK|Canada.Saskatchewan
Baetidae|08-SWRC-1219|United States.Pennsylvania
Baetidae|08-SWRC-1212|United States.Pennsylvania
Baetidae|08-SWRC-1208|United States.Pennsylvania
Baetidae|08-SWRC-1031|United States.Pennsylvania
Baetidae|08-SWRC-0979|United States.Pennsylvania
Diphetor hageni|SWRC-Dh H 20|United States.Pennsylvania
Baetidae|08-SWRC-0813|United States.Pennsylvania
Baetis|08-SWRC-0801|United States.Pennsylvania
Diphetor hageni|08INHSE-0055|United States.Indiana
Diphetor hageni|08INHSE-0056|United States.Indiana
Diphetor hageni|FAMUBOLD0093|United States.Florida
Callibaetis floridanus|BIOUG00637-D04|United States.Florida
Callibaetis sp.DHF1|SWRC-Cf SP 01|United States.Maryland
Callibaetis ferrugineus|474 LC ON|Canada.Ontario
Callibaetis ferrugineus|08MBMAY-0025|Canada.Manitoba
Callibaetis ferrugineus|08MBMAY-0024|Canada.Manitoba
Callibaetis ferrugineus|08MBMAY-0022|Canada.Manitoba
Callibaetis ferrugineus|08MBMAY-0020|Canada.Manitoba
Callibaetis ferrugineus|SWRC-Cf M1 05|United States.Pennsylvania
Callibaetis ferrugineus|10BBEPT-0184|Canada.Alberta
Callibaetis ferrugineus|08MBMAY-0002|Canada.Manitoba
Callibaetis ferrugineus|08MBMAY-0001|Canada.Manitoba
Callibaetis ferrugineus|08MBMAY-0023|Canada.Manitoba
Callibaetis ferrugineus|10-SWRC-0019|United States.Pennsylvania
Callibaetis ferrugineus|LJNY-170|United States.New York
Callibaetis ferrugineus|08INHSE-0046|United States.Illinois
Callibaetis ferrugineus|09NBMAY-0053|Canada.New Brunswick
Callibaetis ferrugineus|10BBEPT-0188|Canada.British Columbia
Callibaetis ferrugineus|10BBEPT-0119|Canada.British Columbia
Callibaetis ferrugineus|10BBEPT-0117|Canada.British Columbia
Callibaetis ferrugineus|10BBEPT-0183|Canada.Alberta
Callibaetis ferrugineus|10BBEPT-0110|Canada.Alberta
Callibaetis ferrugineus|08ABMAY-0034|Canada.Alberta
Callibaetis ferrugineus|10BBEPT-0118|Canada.British Columbia
Callibaetis ferrugineus|10BBEPT-0164|Canada.British Columbia
Callibaetis ferrugineus|10BBEPT-0116|Canada.British Columbia
Callibaetis ferrugineus|10BBEPT-0112|Canada.British Columbia
Callibaetis ferrugineus|08ABMAY-0032|Canada.Alberta
Callibaetis ferrugineus|08ABMAY-0030|Canada.Alberta
Callibaetis ferrugineus|08ABMAY-0001|Canada.Alberta
Callibaetis ferrugineus|10BBEPT-0083|Canada.British Columbia
Callibaetis ferrugineus|10BBEPT-0130|Canada.British Columbia
Callibaetis ferrugineus|10BBEPT-0115|Canada.British Columbia
Callibaetis ferrugineus|10BBEPT-0114|Canada.British Columbia
Callibaetis ferrugineus|10BBEPT-0113|Canada.British Columbia
Callibaetis ferrugineus|10BBEPT-0129|Canada.British Columbia
Baetodes sp.JMW1
Acerpenna sp.JMW1
Acerpenna sp. CHU1
Acerpenna pygmaea
Acerpenna macdunnoughi
Acerpenna pygmaea
Callibaetis fluctuans
Callibaetis pictus
Diphetor hageni
Diphetor hageni
Callibaetis floridanus
Callibaetis sp.DHF1
Callibaetis ferrugineus

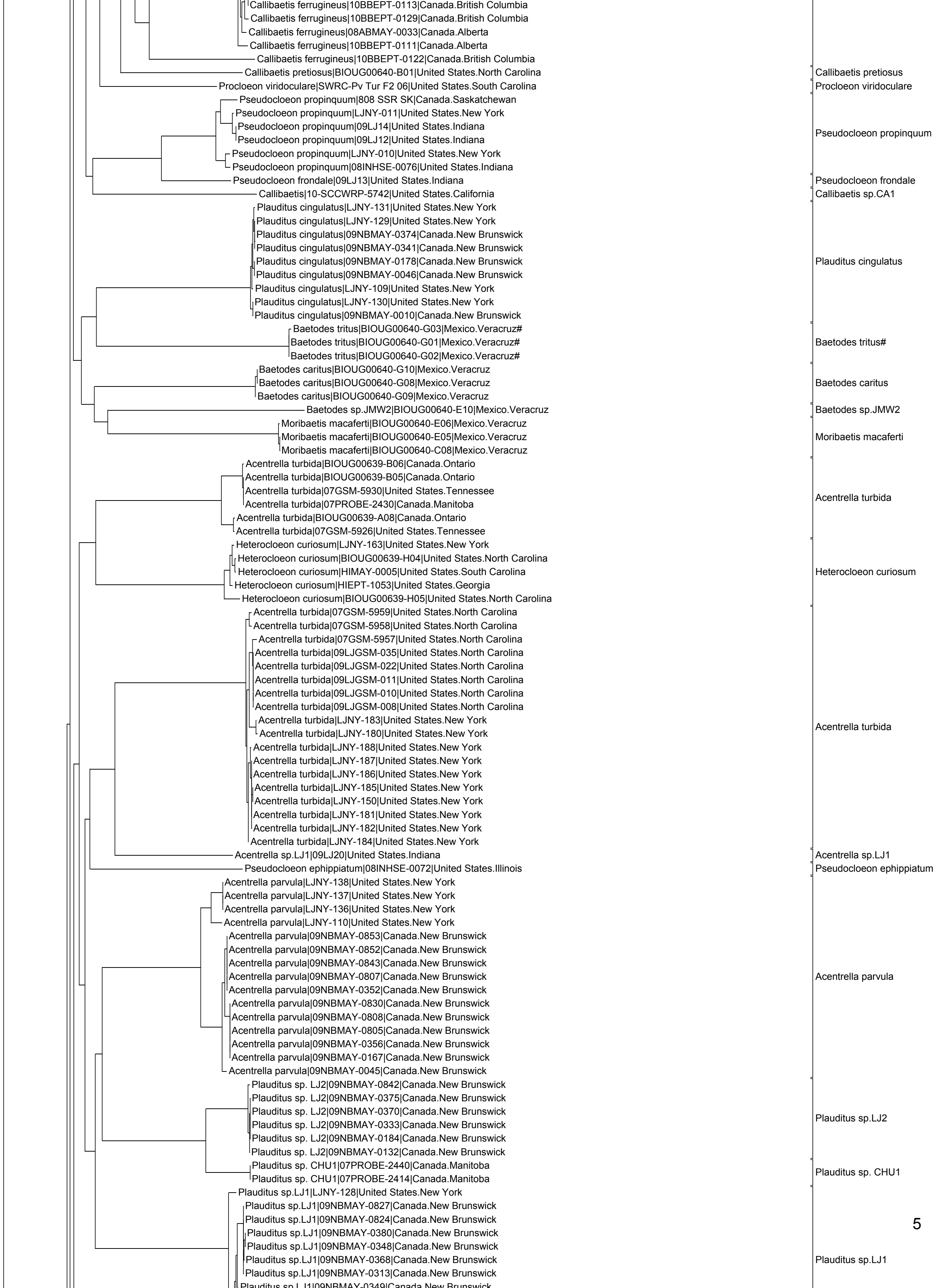

Callibaetis ferrugineus|10BBEPT-0113|Canada.British Columbia
Callibaetis ferrugineus|10BBEPT-0129|Canada.British Columbia
Callibaetis ferrugineus|08ABMAY-0033|Canada.Alberta
Callibaetis ferrugineus|10BBEPT-0111|Canada.Alberta
Callibaetis ferrugineus|10BBEPT-0122|Canada.British Columbia
Callibaetis pretiosus|BIOUG00640-B01|United States.North Carolina
Procloeon viridoculare|SWRC-Pv Tur F2 06|United States.South Carolina
Pseudocloeon propinquum|808 SSR SK|Canada.Saskatchewan
Pseudocloeon propinquum|LJNY-011|United States.New York
Pseudocloeon propinquum|09LJ14|United States.Indiana
Pseudocloeon propinquum|09LJ12|United States.Indiana
Pseudocloeon propinquum|LJNY-010|United States.New York
Pseudocloeon propinquum|08INHSE-0076|United States.Indiana
Pseudocloeon frondale|09LJ13|United States.Indiana
Callibaetis|10-SCCWRP-5742|United States.California
Plauditus cingulatus|LJNY-131|United States.New York
Plauditus cingulatus|LJNY-129|United States.New York
Plauditus cingulatus|09NBMAY-0374|Canada.New Brunswick
Plauditus cingulatus|09NBMAY-0341|Canada.New Brunswick
Plauditus cingulatus|09NBMAY-0178|Canada.New Brunswick
Plauditus cingulatus|09NBMAY-0046|Canada.New Brunswick
Plauditus cingulatus|LJNY-109|United States.New York
Plauditus cingulatus|LJNY-130|United States.New York
Plauditus cingulatus|09NBMAY-0010|Canada.New Brunswick
Baetodes tritus|BIOUG00640-G03|Mexico.Veracruz#
Baetodes tritus|BIOUG00640-G01|Mexico.Veracruz#
Baetodes tritus|BIOUG00640-G02|Mexico.Veracruz#
Baetodes caritus|BIOUG00640-G10|Mexico.Veracruz
Baetodes caritus|BIOUG00640-G08|Mexico.Veracruz
Baetodes caritus|BIOUG00640-G09|Mexico.Veracruz
Baetodes sp.JMW2|BIOUG00640-E10|Mexico.Veracruz
Moribaetis macaferti|BIOUG00640-E06|Mexico.Veracruz
Moribaetis macaferti|BIOUG00640-E05|Mexico.Veracruz
Moribaetis macaferti|BIOUG00640-C08|Mexico.Veracruz
Acentrella turbida|BIOUG00639-B06|Canada.Ontario
Acentrella turbida|BIOUG00639-B05|Canada.Ontario
Acentrella turbida|07GSM-5930|United States.Tennessee
Acentrella turbida|07PROBE-2430|Canada.Manitoba
Acentrella turbida|BIOUG00639-A08|Canada.Ontario
Acentrella turbida|07GSM-5926|United States.Tennessee
Heterocloeon curiosum|LJNY-163|United States.New York
Heterocloeon curiosum|BIOUG00639-H04|United States.North Carolina
Heterocloeon curiosum|HIMAY-0005|United States.South Carolina
Heterocloeon curiosum|HIEPT-1053|United States.Georgia
Heterocloeon curiosum|BIOUG00639-H05|United States.North Carolina
Acentrella turbida|07GSM-5959|United States.North Carolina
Acentrella turbida|07GSM-5958|United States.North Carolina
Acentrella turbida|07GSM-5957|United States.North Carolina
Acentrella turbida|09LJGSM-035|United States.North Carolina
Acentrella turbida|09LJGSM-022|United States.North Carolina
Acentrella turbida|09LJGSM-011|United States.North Carolina
Acentrella turbida|09LJGSM-010|United States.North Carolina
Acentrella turbida|09LJGSM-008|United States.North Carolina
Acentrella turbida|LJNY-183|United States.New York
Acentrella turbida|LJNY-180|United States.New York
Acentrella turbida|LJNY-188|United States.New York
Acentrella turbida|LJNY-187|United States.New York
Acentrella turbida|LJNY-186|United States.New York
Acentrella turbida|LJNY-185|United States.New York
Acentrella turbida|LJNY-150|United States.New York
Acentrella turbida|LJNY-181|United States.New York
Acentrella turbida|LJNY-182|United States.New York
Acentrella turbida|LJNY-184|United States.New York
Acentrella sp.LJ1|09LJ20|United States.Indiana
Pseudocloeon ephippiatum|08INHSE-0072|United States.Illinois
Acentrella parvula|LJNY-138|United States.New York
Acentrella parvula|LJNY-137|United States.New York
Acentrella parvula|LJNY-136|United States.New York
Acentrella parvula|LJNY-110|United States.New York
Acentrella parvula|09NBMAY-0853|Canada.New Brunswick
Acentrella parvula|09NBMAY-0852|Canada.New Brunswick
Acentrella parvula|09NBMAY-0843|Canada.New Brunswick
Acentrella parvula|09NBMAY-0807|Canada.New Brunswick
Acentrella parvula|09NBMAY-0352|Canada.New Brunswick
Acentrella parvula|09NBMAY-0830|Canada.New Brunswick
Acentrella parvula|09NBMAY-0808|Canada.New Brunswick
Acentrella parvula|09NBMAY-0805|Canada.New Brunswick
Acentrella parvula|09NBMAY-0356|Canada.New Brunswick
Acentrella parvula|09NBMAY-0167|Canada.New Brunswick
Acentrella parvula|09NBMAY-0045|Canada.New Brunswick
Plauditus sp. LJ2|09NBMAY-0842|Canada.New Brunswick
Plauditus sp. LJ2|09NBMAY-0375|Canada.New Brunswick
Plauditus sp. LJ2|09NBMAY-0370|Canada.New Brunswick
Plauditus sp. LJ2|09NBMAY-0333|Canada.New Brunswick
Plauditus sp. LJ2|09NBMAY-0184|Canada.New Brunswick
Plauditus sp. LJ2|09NBMAY-0132|Canada.New Brunswick
Plauditus sp. CHU1|07PROBE-2440|Canada.Manitoba
Plauditus sp. CHU1|07PROBE-2414|Canada.Manitoba
Plauditus sp.LJ1|LJNY-128|United States.New York
Plauditus sp.LJ1|09NBMAY-0827|Canada.New Brunswick
Plauditus sp.LJ1|09NBMAY-0824|Canada.New Brunswick
Plauditus sp.LJ1|09NBMAY-0380|Canada.New Brunswick
Plauditus sp.LJ1|09NBMAY-0348|Canada.New Brunswick
Plauditus sp.LJ1|09NBMAY-0368|Canada.New Brunswick
Plauditus sp.LJ1|09NBMAY-0313|Canada.New Brunswick
Plauditus sp.LJ1|09NBMAY-0349|Canada.New Brunswick
Callibaetis pretiosus
Procloeon viridoculare
Pseudocloeon propinquum
Pseudocloeon frondale
Callibaetis sp.CA1
Plauditus cingulatus
Baetodes tritus#
Baetodes caritus
Baetodes sp.JMW2
Moribaetis macaferti
Acentrella turbida
Heterocloeon curiosum
Acentrella turbida
Acentrella sp.LJ1
Pseudocloeon ephippiatum
Acentrella parvula
Plauditus sp.LJ2
Plauditus sp. CHU1
Plauditus sp.LJ1

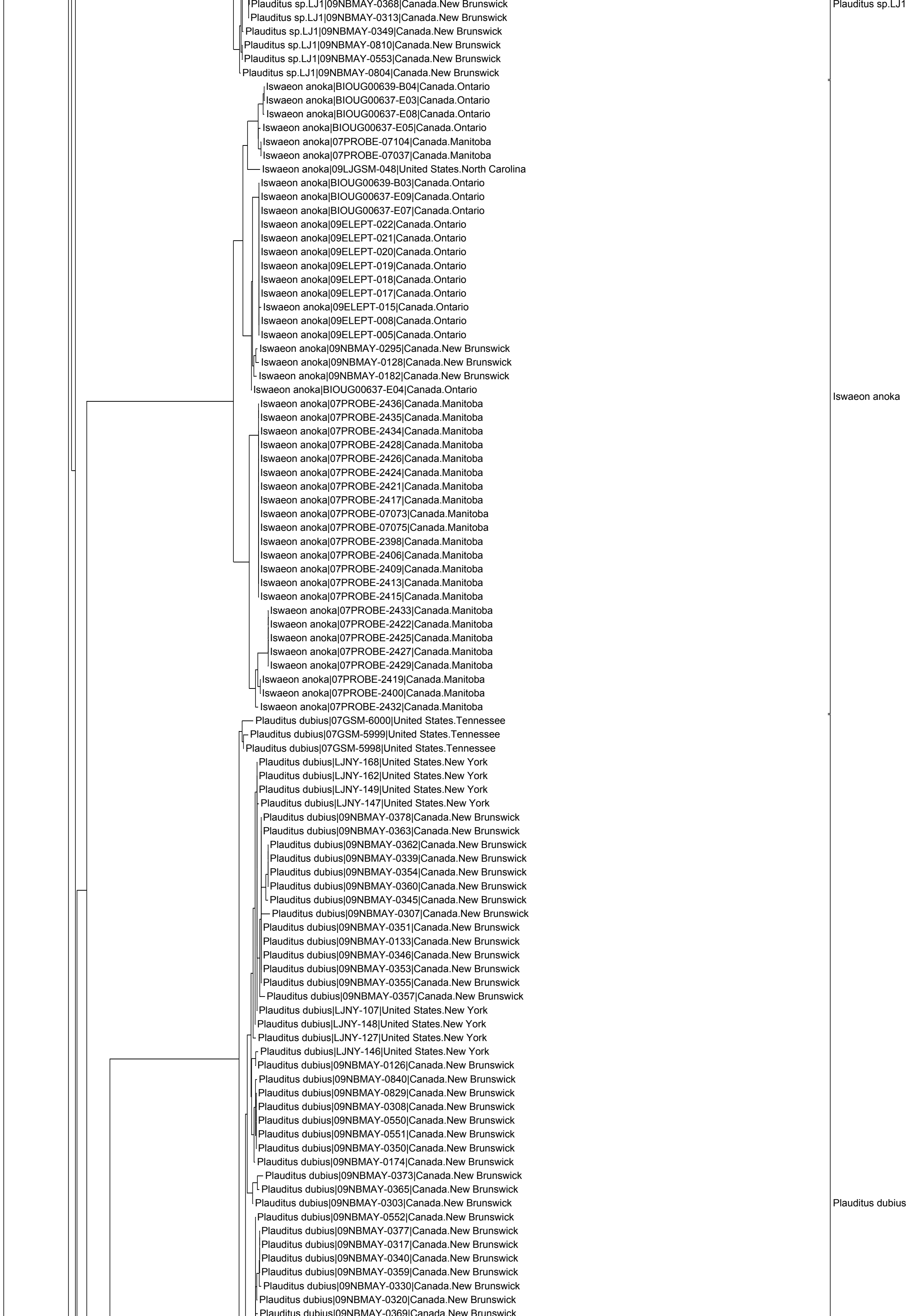
Plauditus sp.LJ1|09NBMAY-0368|Canada.New Brunswick
Plauditus sp.LJ1|09NBMAY-0313|Canada.New Brunswick
Plauditus sp.LJ1|09NBMAY-0349|Canada.New Brunswick
Plauditus sp.LJ1|09NBMAY-0810|Canada.New Brunswick
Plauditus sp.LJ1|09NBMAY-0553|Canada.New Brunswick
Plauditus sp.LJ1|09NBMAY-0804|Canada.New Brunswick
Iswaeon anoka|BIOUG00639-B04|Canada.Ontario
Iswaeon anoka|BIOUG00637-E03|Canada.Ontario
Iswaeon anoka|BIOUG00637-E08|Canada.Ontario
Iswaeon anoka|BIOUG00637-E05|Canada.Ontario
Iswaeon anoka|07PROBE-07104|Canada.Manitoba
Iswaeon anoka|07PROBE-07037|Canada.Manitoba
Iswaeon anoka|09LJGSM-048|United States.North Carolina
Iswaeon anoka|BIOUG00639-B03|Canada.Ontario
Iswaeon anoka|BIOUG00637-E09|Canada.Ontario
Iswaeon anoka|BIOUG00637-E07|Canada.Ontario
Iswaeon anoka|09ELEPT-022|Canada.Ontario
Iswaeon anoka|09ELEPT-021|Canada.Ontario
Iswaeon anoka|09ELEPT-020|Canada.Ontario
Iswaeon anoka|09ELEPT-019|Canada.Ontario
Iswaeon anoka|09ELEPT-018|Canada.Ontario
Iswaeon anoka|09ELEPT-017|Canada.Ontario
Iswaeon anoka|09ELEPT-015|Canada.Ontario
Iswaeon anoka|09ELEPT-008|Canada.Ontario
Iswaeon anoka|09ELEPT-005|Canada.Ontario
Iswaeon anoka|09NBMAY-0295|Canada.New Brunswick
Iswaeon anoka|09NBMAY-0128|Canada.New Brunswick
Iswaeon anoka|09NBMAY-0182|Canada.New Brunswick
Iswaeon anoka|BIOUG00637-E04|Canada.Ontario
Iswaeon anoka|07PROBE-2436|Canada.Manitoba
Iswaeon anoka|07PROBE-2435|Canada.Manitoba
Iswaeon anoka|07PROBE-2434|Canada.Manitoba
Iswaeon anoka|07PROBE-2428|Canada.Manitoba
Iswaeon anoka|07PROBE-2426|Canada.Manitoba
Iswaeon anoka|07PROBE-2424|Canada.Manitoba
Iswaeon anoka|07PROBE-2421|Canada.Manitoba
Iswaeon anoka|07PROBE-2417|Canada.Manitoba
Iswaeon anoka|07PROBE-07073|Canada.Manitoba
Iswaeon anoka|07PROBE-07075|Canada.Manitoba
Iswaeon anoka|07PROBE-2398|Canada.Manitoba
Iswaeon anoka|07PROBE-2406|Canada.Manitoba
Iswaeon anoka|07PROBE-2409|Canada.Manitoba
Iswaeon anoka|07PROBE-2413|Canada.Manitoba
Iswaeon anoka|07PROBE-2415|Canada.Manitoba
Iswaeon anoka|07PROBE-2433|Canada.Manitoba
Iswaeon anoka|07PROBE-2422|Canada.Manitoba
Iswaeon anoka|07PROBE-2425|Canada.Manitoba
Iswaeon anoka|07PROBE-2427|Canada.Manitoba
Iswaeon anoka|07PROBE-2429|Canada.Manitoba
Iswaeon anoka|07PROBE-2419|Canada.Manitoba
Iswaeon anoka|07PROBE-2400|Canada.Manitoba
Iswaeon anoka|07PROBE-2432|Canada.Manitoba
Plauditus dubius|07GSM-6000|United States.Tennessee
Plauditus dubius|07GSM-5999|United States.Tennessee
Plauditus dubius|07GSM-5998|United States.Tennessee
Plauditus dubius|LJNY-168|United States.New York
Plauditus dubius|LJNY-162|United States.New York
Plauditus dubius|LJNY-149|United States.New York
Plauditus dubius|LJNY-147|United States.New York
Plauditus dubius|09NBMAY-0378|Canada.New Brunswick
Plauditus dubius|09NBMAY-0363|Canada.New Brunswick
Plauditus dubius|09NBMAY-0362|Canada.New Brunswick
Plauditus dubius|09NBMAY-0339|Canada.New Brunswick
Plauditus dubius|09NBMAY-0354|Canada.New Brunswick
Plauditus dubius|09NBMAY-0360|Canada.New Brunswick
Plauditus dubius|09NBMAY-0345|Canada.New Brunswick
Plauditus dubius|09NBMAY-0307|Canada.New Brunswick
Plauditus dubius|09NBMAY-0351|Canada.New Brunswick
Plauditus dubius|09NBMAY-0133|Canada.New Brunswick
Plauditus dubius|09NBMAY-0346|Canada.New Brunswick
Plauditus dubius|09NBMAY-0353|Canada.New Brunswick
Plauditus dubius|09NBMAY-0355|Canada.New Brunswick
Plauditus dubius|09NBMAY-0357|Canada.New Brunswick
Plauditus dubius|LJNY-107|United States.New York
Plauditus dubius|LJNY-148|United States.New York
Plauditus dubius|LJNY-127|United States.New York
Plauditus dubius|LJNY-146|United States.New York
Plauditus dubius|09NBMAY-0126|Canada.New Brunswick
Plauditus dubius|09NBMAY-0840|Canada.New Brunswick
Plauditus dubius|09NBMAY-0829|Canada.New Brunswick
Plauditus dubius|09NBMAY-0308|Canada.New Brunswick
Plauditus dubius|09NBMAY-0550|Canada.New Brunswick
Plauditus dubius|09NBMAY-0551|Canada.New Brunswick
Plauditus dubius|09NBMAY-0350|Canada.New Brunswick
Plauditus dubius|09NBMAY-0174|Canada.New Brunswick
Plauditus dubius|09NBMAY-0373|Canada.New Brunswick
Plauditus dubius|09NBMAY-0365|Canada.New Brunswick
Plauditus dubius|09NBMAY-0303|Canada.New Brunswick
Plauditus dubius|09NBMAY-0552|Canada.New Brunswick
Plauditus dubius|09NBMAY-0377|Canada.New Brunswick
Plauditus dubius|09NBMAY-0317|Canada.New Brunswick
Plauditus dubius|09NBMAY-0340|Canada.New Brunswick
Plauditus dubius|09NBMAY-0359|Canada.New Brunswick
Plauditus dubius|09NBMAY-0330|Canada.New Brunswick
Plauditus dubius|09NBMAY-0320|Canada.New Brunswick
Plauditus dubius|09NBMAY-0369|Canada.New Brunswick
Plauditus sp.LJ1
Iswaeon anoka
Plauditus dubius

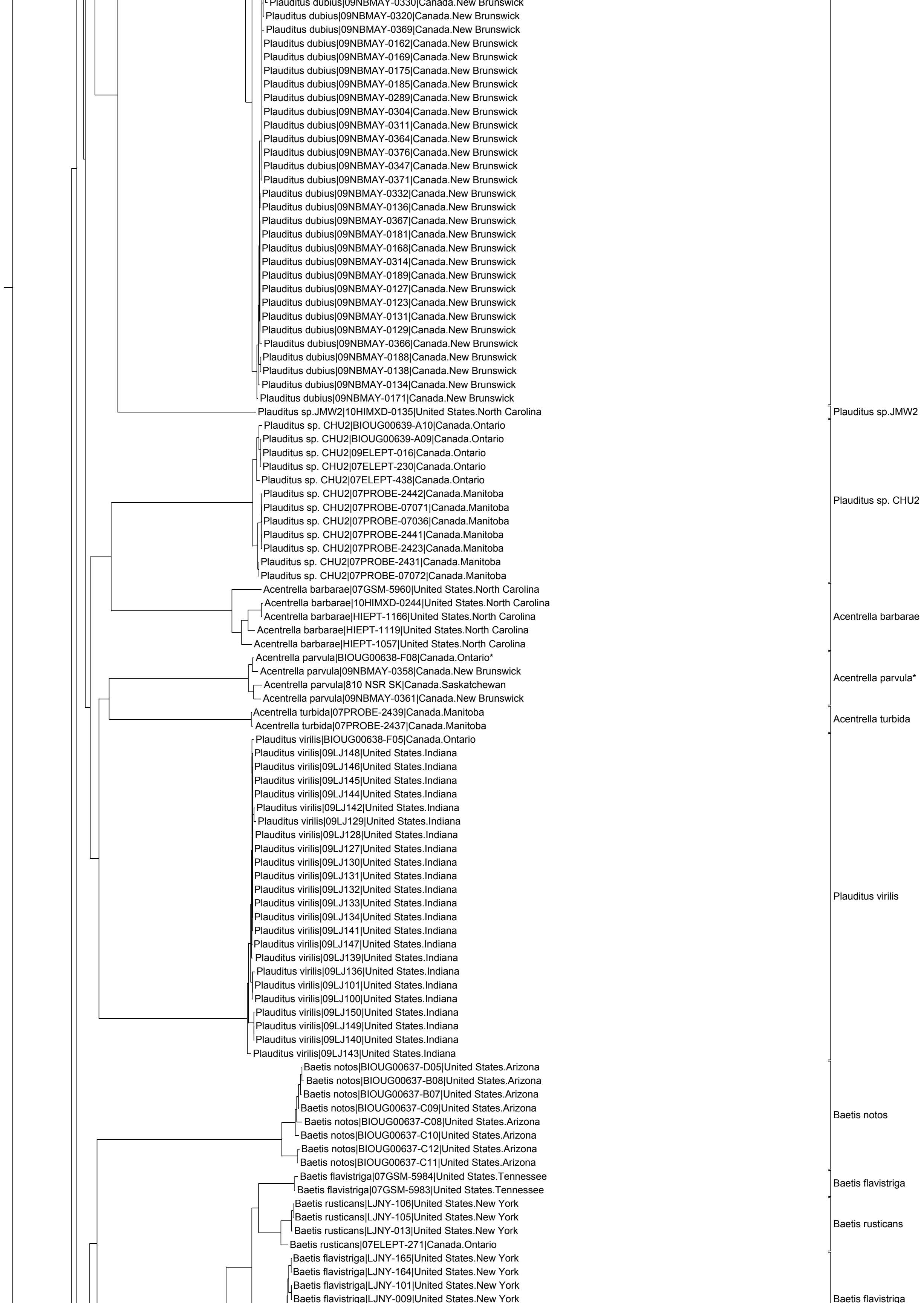
Plauditus dubius|09NBMAY-0330|Canada.New Brunswick
Plauditus dubius|09NBMAY-0320|Canada.New Brunswick
Plauditus dubius|09NBMAY-0369|Canada.New Brunswick
Plauditus dubius|09NBMAY-0162|Canada.New Brunswick
Plauditus dubius|09NBMAY-0169|Canada.New Brunswick
Plauditus dubius|09NBMAY-0175|Canada.New Brunswick
Plauditus dubius|09NBMAY-0185|Canada.New Brunswick
Plauditus dubius|09NBMAY-0289|Canada.New Brunswick
Plauditus dubius|09NBMAY-0304|Canada.New Brunswick
Plauditus dubius|09NBMAY-0311|Canada.New Brunswick
Plauditus dubius|09NBMAY-0364|Canada.New Brunswick
Plauditus dubius|09NBMAY-0376|Canada.New Brunswick
Plauditus dubius|09NBMAY-0347|Canada.New Brunswick
Plauditus dubius|09NBMAY-0371|Canada.New Brunswick
Plauditus dubius|09NBMAY-0332|Canada.New Brunswick
Plauditus dubius|09NBMAY-0136|Canada.New Brunswick
Plauditus dubius|09NBMAY-0367|Canada.New Brunswick
Plauditus dubius|09NBMAY-0181|Canada.New Brunswick
Plauditus dubius|09NBMAY-0168|Canada.New Brunswick
Plauditus dubius|09NBMAY-0314|Canada.New Brunswick
Plauditus dubius|09NBMAY-0189|Canada.New Brunswick
Plauditus dubius|09NBMAY-0127|Canada.New Brunswick
Plauditus dubius|09NBMAY-0123|Canada.New Brunswick
Plauditus dubius|09NBMAY-0131|Canada.New Brunswick
Plauditus dubius|09NBMAY-0129|Canada.New Brunswick
Plauditus dubius|09NBMAY-0366|Canada.New Brunswick
Plauditus dubius|09NBMAY-0188|Canada.New Brunswick
Plauditus dubius|09NBMAY-0138|Canada.New Brunswick
Plauditus dubius|09NBMAY-0134|Canada.New Brunswick
Plauditus dubius|09NBMAY-0171|Canada.New Brunswick
Plauditus sp.JMW2|10HIMXD-0135|United States.North Carolina
Plauditus sp.JMW2
Plauditus sp. CHU2|BIOUG00639-A10|Canada.Ontario
Plauditus sp. CHU2|BIOUG00639-A09|Canada.Ontario
Plauditus sp. CHU2|09ELEPT-016|Canada.Ontario
Plauditus sp. CHU2|07ELEPT-230|Canada.Ontario
Plauditus sp. CHU2|07ELEPT-438|Canada.Ontario
Plauditus sp. CHU2|07PROBE-2442|Canada.Manitoba
Plauditus sp. CHU2|07PROBE-07071|Canada.Manitoba
Plauditus sp. CHU2|07PROBE-07036|Canada.Manitoba
Plauditus sp. CHU2|07PROBE-2441|Canada.Manitoba
Plauditus sp. CHU2|07PROBE-2423|Canada.Manitoba
Plauditus sp. CHU2|07PROBE-2431|Canada.Manitoba
Plauditus sp. CHU2|07PROBE-07072|Canada.Manitoba
Plauditus sp. CHU2
Acentrella barbarae|07GSM-5960|United States.North Carolina
Acentrella barbarae|10HIMXD-0244|United States.North Carolina
Acentrella barbarae|HIEPT-1166|United States.North Carolina
Acentrella barbarae|HIEPT-1119|United States.North Carolina
Acentrella barbarae|HIEPT-1057|United States.North Carolina
Acentrella barbarae
Acentrella parvula|BIOUG00638-F08|Canada.Ontario*
Acentrella parvula|09NBMAY-0358|Canada.New Brunswick
Acentrella parvula|810 NSR SK|Canada.Saskatchewan
Acentrella parvula|09NBMAY-0361|Canada.New Brunswick
Acentrella parvula*
Acentrella turbida|07PROBE-2439|Canada.Manitoba
Acentrella turbida|07PROBE-2437|Canada.Manitoba
Acentrella turbida
Plauditus virilis|BIOUG00638-F05|Canada.Ontario
Plauditus virilis|09LJ148|United States.Indiana
Plauditus virilis|09LJ146|United States.Indiana
Plauditus virilis|09LJ145|United States.Indiana
Plauditus virilis|09LJ144|United States.Indiana
Plauditus virilis|09LJ142|United States.Indiana
Plauditus virilis|09LJ129|United States.Indiana
Plauditus virilis|09LJ128|United States.Indiana
Plauditus virilis|09LJ127|United States.Indiana
Plauditus virilis|09LJ130|United States.Indiana
Plauditus virilis|09LJ131|United States.Indiana
Plauditus virilis|09LJ132|United States.Indiana
Plauditus virilis|09LJ133|United States.Indiana
Plauditus virilis|09LJ134|United States.Indiana
Plauditus virilis|09LJ141|United States.Indiana
Plauditus virilis|09LJ147|United States.Indiana
Plauditus virilis|09LJ139|United States.Indiana
Plauditus virilis|09LJ136|United States.Indiana
Plauditus virilis|09LJ101|United States.Indiana
Plauditus virilis|09LJ100|United States.Indiana
Plauditus virilis|09LJ150|United States.Indiana
Plauditus virilis|09LJ149|United States.Indiana
Plauditus virilis|09LJ140|United States.Indiana
Plauditus virilis|09LJ143|United States.Indiana
Plauditus virilis
Baetis notos|BIOUG00637-D05|United States.Arizona
Baetis notos|BIOUG00637-B08|United States.Arizona
Baetis notos|BIOUG00637-B07|United States.Arizona
Baetis notos|BIOUG00637-C09|United States.Arizona
Baetis notos|BIOUG00637-C08|United States.Arizona
Baetis notos|BIOUG00637-C10|United States.Arizona
Baetis notos|BIOUG00637-C12|United States.Arizona
Baetis notos|BIOUG00637-C11|United States.Arizona
Baetis notos
Baetis flavistriga|07GSM-5984|United States.Tennessee
Baetis flavistriga|07GSM-5983|United States.Tennessee
Baetis flavistriga
Baetis rusticans|LJNY-106|United States.New York
Baetis rusticans|LJNY-105|United States.New York
Baetis rusticans|LJNY-013|United States.New York
Baetis rusticans|07ELEPT-271|Canada.Ontario
Baetis rusticans
Baetis flavistriga|LJNY-165|United States.New York
Baetis flavistriga|LJNY-164|United States.New York
Baetis flavistriga|LJNY-101|United States.New York
Baetis flavistriga|LJNY-009|United States.New York
Baetis flavistriga

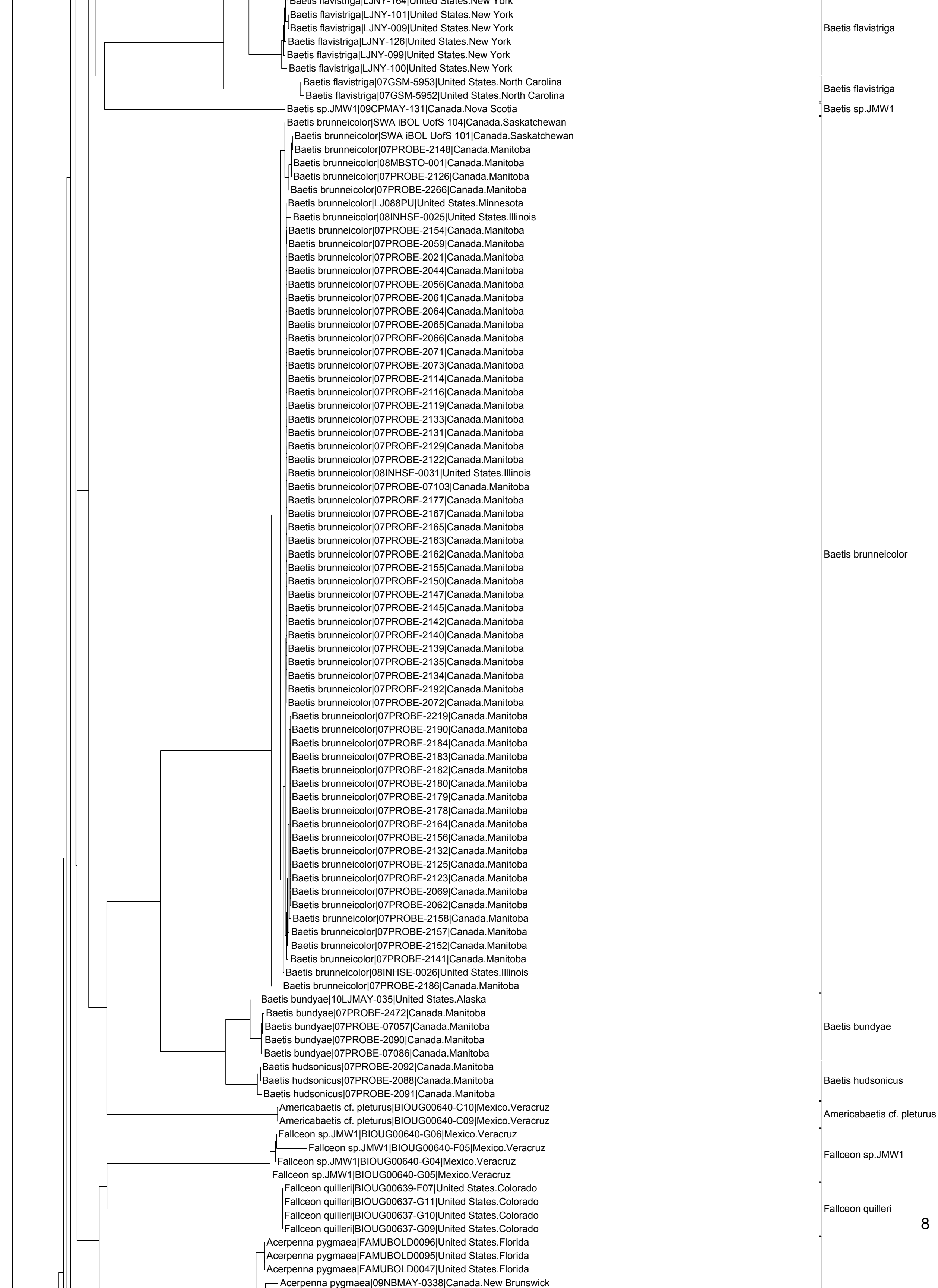
Baetis flavistriga|LJNY-164|United States.New York
Baetis flavistriga|LJNY-101|United States.New York
Baetis flavistriga|LJNY-009|United States.New York
Baetis flavistriga|LJNY-126|United States.New York
Baetis flavistriga|LJNY-099|United States.New York
Baetis flavistriga|LJNY-100|United States.New York
Baetis flavistriga
Baetis flavistriga|07GSM-5953|United States.North Carolina
Baetis flavistriga|07GSM-5952|United States.North Carolina
Baetis flavistriga
Baetis sp.JMW1|09CPMAY-131|Canada.Nova Scotia
Baetis sp.JMW1
Baetis brunneicolor|SWA iBOL UofS 104|Canada.Saskatchewan
Baetis brunneicolor|SWA iBOL UofS 101|Canada.Saskatchewan
Baetis brunneicolor|07PROBE-2148|Canada.Manitoba
Baetis brunneicolor|08MBSTO-001|Canada.Manitoba
Baetis brunneicolor|07PROBE-2126|Canada.Manitoba
Baetis brunneicolor|07PROBE-2266|Canada.Manitoba
Baetis brunneicolor|LJ088PU|United States.Minnesota
Baetis brunneicolor|08INHSE-0025|United States.Illinois
Baetis brunneicolor|07PROBE-2154|Canada.Manitoba
Baetis brunneicolor|07PROBE-2059|Canada.Manitoba
Baetis brunneicolor|07PROBE-2021|Canada.Manitoba
Baetis brunneicolor|07PROBE-2044|Canada.Manitoba
Baetis brunneicolor|07PROBE-2056|Canada.Manitoba
Baetis brunneicolor|07PROBE-2061|Canada.Manitoba
Baetis brunneicolor|07PROBE-2064|Canada.Manitoba
Baetis brunneicolor|07PROBE-2065|Canada.Manitoba
Baetis brunneicolor|07PROBE-2066|Canada.Manitoba
Baetis brunneicolor|07PROBE-2071|Canada.Manitoba
Baetis brunneicolor|07PROBE-2073|Canada.Manitoba
Baetis brunneicolor|07PROBE-2114|Canada.Manitoba
Baetis brunneicolor|07PROBE-2116|Canada.Manitoba
Baetis brunneicolor|07PROBE-2119|Canada.Manitoba
Baetis brunneicolor|07PROBE-2133|Canada.Manitoba
Baetis brunneicolor|07PROBE-2131|Canada.Manitoba
Baetis brunneicolor|07PROBE-2129|Canada.Manitoba
Baetis brunneicolor|07PROBE-2122|Canada.Manitoba
Baetis brunneicolor|08INHSE-0031|United States.Illinois
Baetis brunneicolor|07PROBE-07103|Canada.Manitoba
Baetis brunneicolor|07PROBE-2177|Canada.Manitoba
Baetis brunneicolor|07PROBE-2167|Canada.Manitoba
Baetis brunneicolor|07PROBE-2165|Canada.Manitoba
Baetis brunneicolor|07PROBE-2163|Canada.Manitoba
Baetis brunneicolor|07PROBE-2162|Canada.Manitoba
Baetis brunneicolor
Baetis brunneicolor|07PROBE-2155|Canada.Manitoba
Baetis brunneicolor|07PROBE-2150|Canada.Manitoba
Baetis brunneicolor|07PROBE-2147|Canada.Manitoba
Baetis brunneicolor|07PROBE-2145|Canada.Manitoba
Baetis brunneicolor|07PROBE-2142|Canada.Manitoba
Baetis brunneicolor|07PROBE-2140|Canada.Manitoba
Baetis brunneicolor|07PROBE-2139|Canada.Manitoba
Baetis brunneicolor|07PROBE-2135|Canada.Manitoba
Baetis brunneicolor|07PROBE-2134|Canada.Manitoba
Baetis brunneicolor|07PROBE-2192|Canada.Manitoba
Baetis brunneicolor|07PROBE-2072|Canada.Manitoba
Baetis brunneicolor|07PROBE-2219|Canada.Manitoba
Baetis brunneicolor|07PROBE-2190|Canada.Manitoba
Baetis brunneicolor|07PROBE-2184|Canada.Manitoba
Baetis brunneicolor|07PROBE-2183|Canada.Manitoba
Baetis brunneicolor|07PROBE-2182|Canada.Manitoba
Baetis brunneicolor|07PROBE-2180|Canada.Manitoba
Baetis brunneicolor|07PROBE-2179|Canada.Manitoba
Baetis brunneicolor|07PROBE-2178|Canada.Manitoba
Baetis brunneicolor|07PROBE-2164|Canada.Manitoba
Baetis brunneicolor|07PROBE-2156|Canada.Manitoba
Baetis brunneicolor|07PROBE-2132|Canada.Manitoba
Baetis brunneicolor|07PROBE-2125|Canada.Manitoba
Baetis brunneicolor|07PROBE-2123|Canada.Manitoba
Baetis brunneicolor|07PROBE-2069|Canada.Manitoba
Baetis brunneicolor|07PROBE-2062|Canada.Manitoba
Baetis brunneicolor|07PROBE-2158|Canada.Manitoba
Baetis brunneicolor|07PROBE-2157|Canada.Manitoba
Baetis brunneicolor|07PROBE-2152|Canada.Manitoba
Baetis brunneicolor|07PROBE-2141|Canada.Manitoba
Baetis brunneicolor|08INHSE-0026|United States.Illinois
Baetis brunneicolor|07PROBE-2186|Canada.Manitoba
Baetis bundyae|10LJMAY-035|United States.Alaska
Baetis bundyae|07PROBE-2472|Canada.Manitoba
Baetis bundyae|07PROBE-07057|Canada.Manitoba
Baetis bundyae
Baetis bundyae|07PROBE-2090|Canada.Manitoba
Baetis bundyae|07PROBE-07086|Canada.Manitoba
Baetis hudsonicus|07PROBE-2092|Canada.Manitoba
Baetis hudsonicus|07PROBE-2088|Canada.Manitoba
Baetis hudsonicus
Baetis hudsonicus|07PROBE-2091|Canada.Manitoba
Americabaetis cf. pleturus|BIOUG00640-C10|Mexico.Veracruz
Americabaetis cf. pleturus
Americabaetis cf. pleturus|BIOUG00640-C09|Mexico.Veracruz
Fallceon sp.JMW1|BIOUG00640-G06|Mexico.Veracruz
Fallceon sp.JMW1|BIOUG00640-F05|Mexico.Veracruz
Fallceon sp.JMW1
Fallceon sp.JMW1|BIOUG00640-G04|Mexico.Veracruz
Fallceon sp.JMW1|BIOUG00640-G05|Mexico.Veracruz
Fallceon quilleri|BIOUG00639-F07|United States.Colorado
Fallceon quilleri|BIOUG00637-G11|United States.Colorado
Fallceon quilleri|BIOUG00637-G10|United States.Colorado
Fallceon quilleri
Fallceon quilleri|BIOUG00637-G09|United States.Colorado
Acerpenna pygmaea|FAMUBOLD0096|United States.Florida
Acerpenna pygmaea|FAMUBOLD0095|United States.Florida
Acerpenna pygmaea|FAMUBOLD0047|United States.Florida
Acerpenna pygmaea|09NBMAY-0338|Canada.New Brunswick

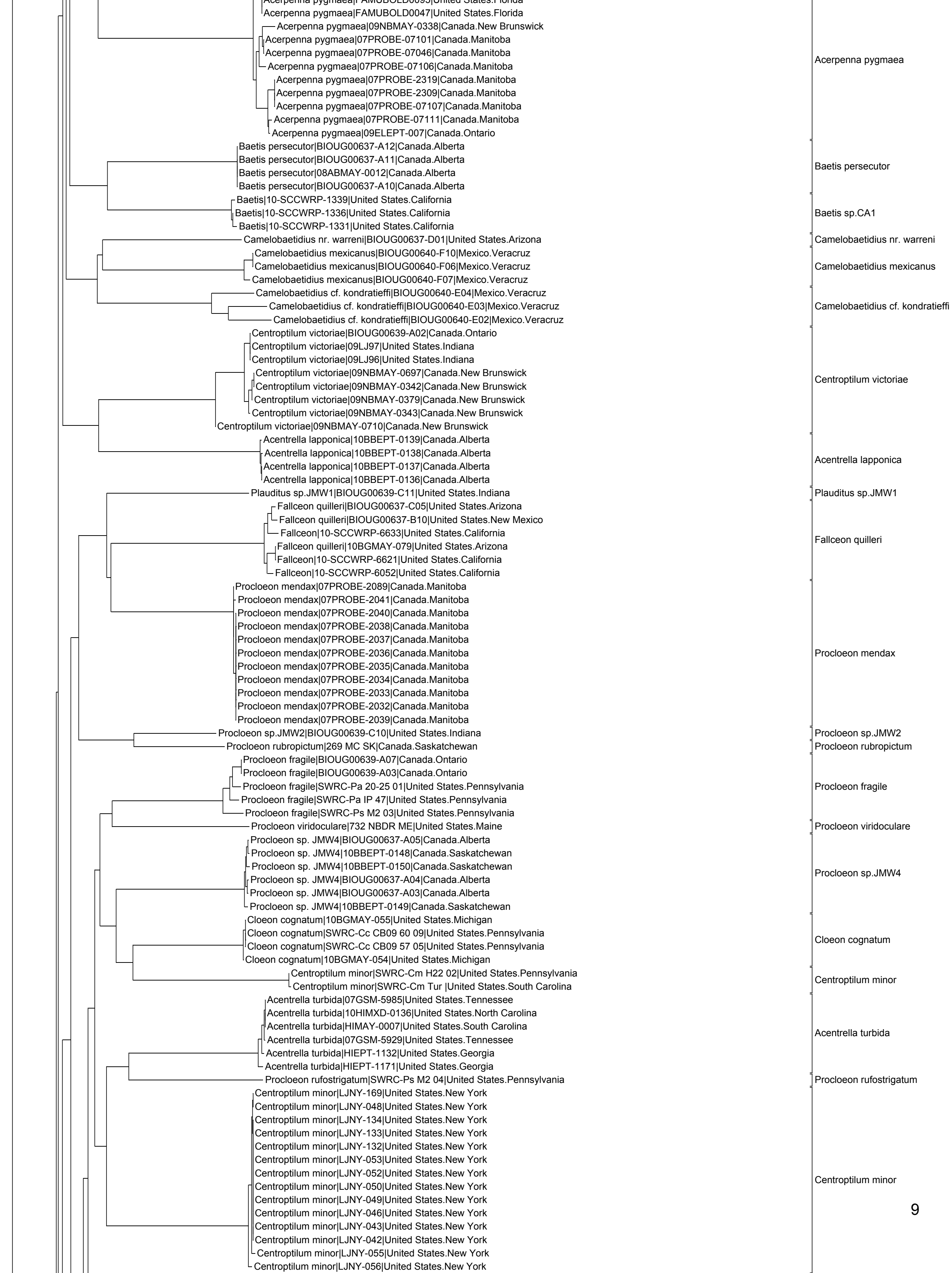

Acerpenna pygmaea|FAMUBOLD0095|United States.Florida
Acerpenna pygmaea|FAMUBOLD0047|United States.Florida
Acerpenna pygmaea|09NBMAY-0338|Canada.New Brunswick
Acerpenna pygmaea|07PROBE-07101|Canada.Manitoba
Acerpenna pygmaea|07PROBE-07046|Canada.Manitoba
Acerpenna pygmaea|07PROBE-07106|Canada.Manitoba
Acerpenna pygmaea|07PROBE-2319|Canada.Manitoba
Acerpenna pygmaea|07PROBE-2309|Canada.Manitoba
Acerpenna pygmaea|07PROBE-07107|Canada.Manitoba
Acerpenna pygmaea|07PROBE-07111|Canada.Manitoba
Acerpenna pygmaea|09ELEPT-007|Canada.Ontario
Acerpenna pygmaea
Baetis persecutor|BIOUG00637-A12|Canada.Alberta
Baetis persecutor|BIOUG00637-A11|Canada.Alberta
Baetis persecutor|08ABMAY-0012|Canada.Alberta
Baetis persecutor|BIOUG00637-A10|Canada.Alberta
Baetis persecutor
Baetis|10-SCCWRP-1339|United States.California
Baetis|10-SCCWRP-1336|United States.California
Baetis|10-SCCWRP-1331|United States.California
Baetis sp.CA1
Camelobaetidius nr. warreni|BIOUG00637-D01|United States.Arizona
Camelobaetidius nr. warreni
Camelobaetidius mexicanus|BIOUG00640-F10|Mexico.Veracruz
Camelobaetidius mexicanus|BIOUG00640-F06|Mexico.Veracruz
Camelobaetidius mexicanus|BIOUG00640-F07|Mexico.Veracruz
Camelobaetidius mexicanus
Camelobaetidius cf. kondratieffi|BIOUG00640-E04|Mexico.Veracruz
Camelobaetidius cf. kondratieffi|BIOUG00640-E03|Mexico.Veracruz
Camelobaetidius cf. kondratieffi|BIOUG00640-E02|Mexico.Veracruz
Camelobaetidius cf. kondratieffi
Centroptilum victoriae|BIOUG00639-A02|Canada.Ontario
Centroptilum victoriae|09LJ97|United States.Indiana
Centroptilum victoriae|09LJ96|United States.Indiana
Centroptilum victoriae|09NBMAY-0697|Canada.New Brunswick
Centroptilum victoriae|09NBMAY-0342|Canada.New Brunswick
Centroptilum victoriae|09NBMAY-0379|Canada.New Brunswick
Centroptilum victoriae|09NBMAY-0343|Canada.New Brunswick
Centroptilum victoriae|09NBMAY-0710|Canada.New Brunswick
Centroptilum victoriae
Acentrella lapponica|10BBEPT-0139|Canada.Alberta
Acentrella lapponica|10BBEPT-0138|Canada.Alberta
Acentrella lapponica|10BBEPT-0137|Canada.Alberta
Acentrella lapponica|10BBEPT-0136|Canada.Alberta
Acentrella lapponica
Plauditus sp.JMW1|BIOUG00639-C11|United States.Indiana
Plauditus sp.JMW1
Fallceon quilleri|BIOUG00637-C05|United States.Arizona
Fallceon quilleri|BIOUG00637-B10|United States.New Mexico
Fallceon|10-SCCWRP-6633|United States.California
Fallceon quilleri|10BGMAY-079|United States.Arizona
Fallceon|10-SCCWRP-6621|United States.California
Fallceon|10-SCCWRP-6052|United States.California
Fallceon quilleri
Procloeon mendax|07PROBE-2089|Canada.Manitoba
Procloeon mendax|07PROBE-2041|Canada.Manitoba
Procloeon mendax|07PROBE-2040|Canada.Manitoba
Procloeon mendax|07PROBE-2038|Canada.Manitoba
Procloeon mendax|07PROBE-2037|Canada.Manitoba
Procloeon mendax|07PROBE-2036|Canada.Manitoba
Procloeon mendax|07PROBE-2035|Canada.Manitoba
Procloeon mendax|07PROBE-2034|Canada.Manitoba
Procloeon mendax|07PROBE-2033|Canada.Manitoba
Procloeon mendax|07PROBE-2032|Canada.Manitoba
Procloeon mendax|07PROBE-2039|Canada.Manitoba
Procloeon mendax
Procloeon sp.JMW2|BIOUG00639-C10|United States.Indiana
Procloeon sp.JMW2
Procloeon rubropictum|269 MC SK|Canada.Saskatchewan
Procloeon rubropictum
Procloeon fragile|BIOUG00639-A07|Canada.Ontario
Procloeon fragile|BIOUG00639-A03|Canada.Ontario
Procloeon fragile|SWRC-Pa 20-25 01|United States.Pennsylvania
Procloeon fragile|SWRC-Pa IP 47|United States.Pennsylvania
Procloeon fragile|SWRC-Ps M2 03|United States.Pennsylvania
Procloeon fragile
Procloeon viridoculare|732 NBDR ME|United States.Maine
Procloeon viridoculare
Procloeon sp. JMW4|BIOUG00637-A05|Canada.Alberta
Procloeon sp. JMW4|10BBEPT-0148|Canada.Saskatchewan
Procloeon sp. JMW4|10BBEPT-0150|Canada.Saskatchewan
Procloeon sp. JMW4|BIOUG00637-A04|Canada.Alberta
Procloeon sp. JMW4|BIOUG00637-A03|Canada.Alberta
Procloeon sp. JMW4|10BBEPT-0149|Canada.Saskatchewan
Procloeon sp.JMW4
Cloeon cognatum|10BGMAY-055|United States.Michigan
Cloeon cognatum|SWRC-Cc CB09 60 09|United States.Pennsylvania
Cloeon cognatum|SWRC-Cc CB09 57 05|United States.Pennsylvania
Cloeon cognatum|10BGMAY-054|United States.Michigan
Cloeon cognatum
Centroptilum minor|SWRC-Cm H22 02|United States.Pennsylvania
Centroptilum minor|SWRC-Cm Tur |United States.South Carolina
Centroptilum minor
Acentrella turbida|07GSM-5985|United States.Tennessee
Acentrella turbida|10HIMXD-0136|United States.North Carolina
Acentrella turbida|HIMAY-0007|United States.South Carolina
Acentrella turbida|07GSM-5929|United States.Tennessee
Acentrella turbida|HIEPT-1132|United States.Georgia
Acentrella turbida|HIEPT-1171|United States.Georgia
Acentrella turbida
Procloeon rufostrigatum|SWRC-Ps M2 04|United States.Pennsylvania
Procloeon rufostrigatum
Centroptilum minor|LJNY-169|United States.New York
Centroptilum minor|LJNY-048|United States.New York
Centroptilum minor|LJNY-134|United States.New York
Centroptilum minor|LJNY-133|United States.New York
Centroptilum minor|LJNY-132|United States.New York
Centroptilum minor|LJNY-053|United States.New York
Centroptilum minor|LJNY-052|United States.New York
Centroptilum minor|LJNY-050|United States.New York
Centroptilum minor|LJNY-049|United States.New York
Centroptilum minor|LJNY-046|United States.New York
Centroptilum minor|LJNY-043|United States.New York
Centroptilum minor|LJNY-042|United States.New York
Centroptilum minor|LJNY-055|United States.New York
Centroptilum minor|LJNY-056|United States.New York
Centroptilum minor

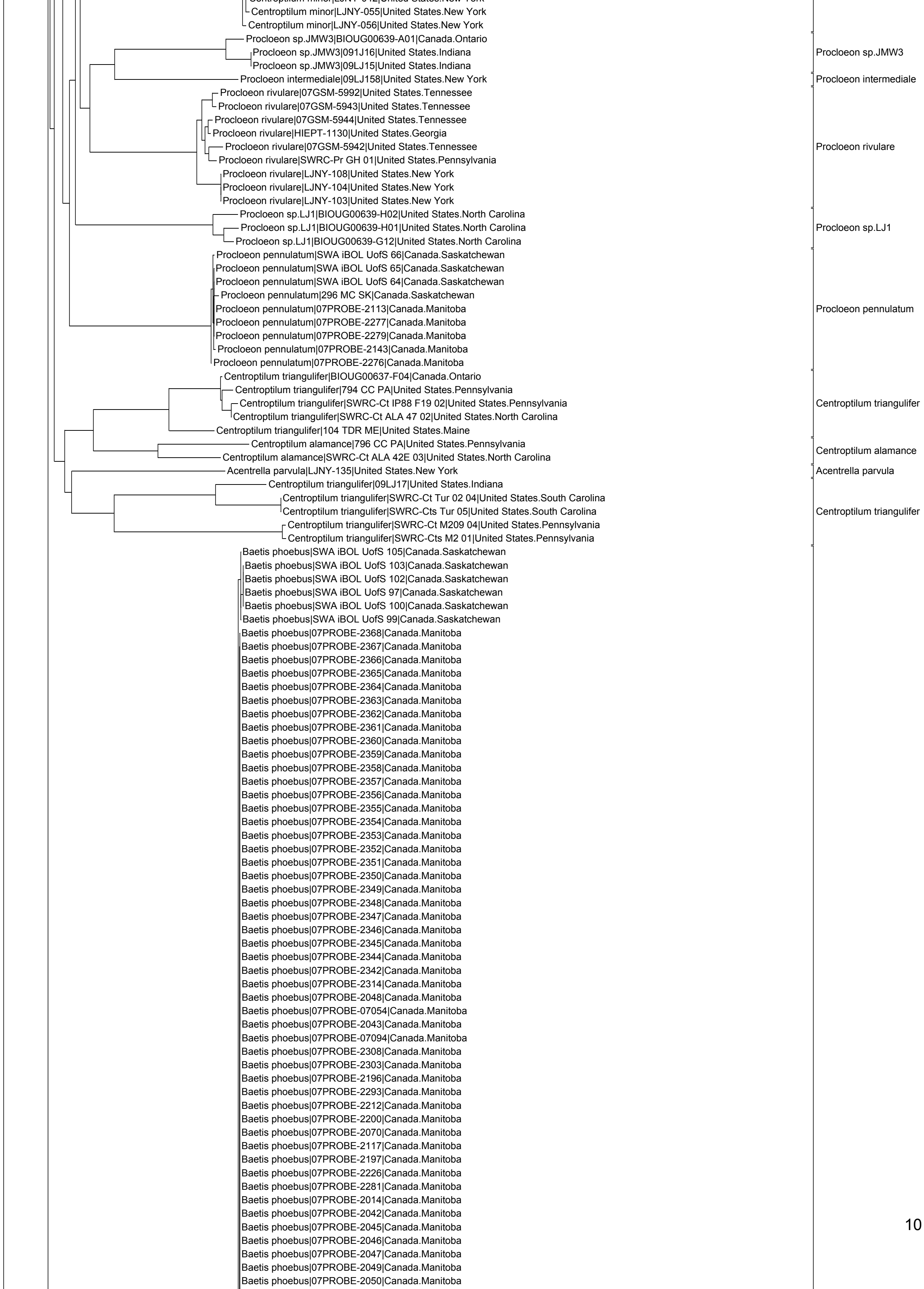
Centroptilum minor|LJNY-055|United States.New York
Centroptilum minor|LJNY-056|United States.New York
Procloeon sp.JMW3|BIOUG00639-A01|Canada.Ontario
Procloeon sp.JMW3|091J16|United States.Indiana
Procloeon sp.JMW3|09LJ15|United States.Indiana
Procloeon intermediale|09LJ158|United States.New York
Procloeon rivulare|07GSM-5992|United States.Tennessee
Procloeon rivulare|07GSM-5943|United States.Tennessee
Procloeon rivulare|07GSM-5944|United States.Tennessee
Procloeon rivulare|HIEPT-1130|United States.Georgia
Procloeon rivulare|07GSM-5942|United States.Tennessee
Procloeon rivulare|SWRC-Pr GH 01|United States.Pennsylvania
Procloeon rivulare|LJNY-108|United States.New York
Procloeon rivulare|LJNY-104|United States.New York
Procloeon rivulare|LJNY-103|United States.New York
Procloeon sp.LJ1|BIOUG00639-H02|United States.North Carolina
Procloeon sp.LJ1|BIOUG00639-H01|United States.North Carolina
Procloeon sp.LJ1|BIOUG00639-G12|United States.North Carolina
Procloeon pennulatum|SWA iBOL UofS 66|Canada.Saskatchewan
Procloeon pennulatum|SWA iBOL UofS 65|Canada.Saskatchewan
Procloeon pennulatum|SWA iBOL UofS 64|Canada.Saskatchewan
Procloeon pennulatum|296 MC SK|Canada.Saskatchewan
Procloeon pennulatum|07PROBE-2113|Canada.Manitoba
Procloeon pennulatum|07PROBE-2277|Canada.Manitoba
Procloeon pennulatum|07PROBE-2279|Canada.Manitoba
Procloeon pennulatum|07PROBE-2143|Canada.Manitoba
Procloeon pennulatum|07PROBE-2276|Canada.Manitoba
Centroptilum triangulifer|BIOUG00637-F04|Canada.Ontario
Centroptilum triangulifer|794 CC PA|United States.Pennsylvania
Centroptilum triangulifer|SWRC-Ct IP88 F19 02|United States.Pennsylvania
Centroptilum triangulifer|SWRC-Ct ALA 47 02|United States.North Carolina
Centroptilum triangulifer|104 TDR ME|United States.Maine
Centroptilum alamance|796 CC PA|United States.Pennsylvania
Centroptilum alamance|SWRC-Ct ALA 42E 03|United States.North Carolina
Acentrella parvula|LJNY-135|United States.New York
Centroptilum triangulifer|09LJ17|United States.Indiana
Centroptilum triangulifer|SWRC-Ct Tur 02 04|United States.South Carolina
Centroptilum triangulifer|SWRC-Cts Tur 05|United States.South Carolina
Centroptilum triangulifer|SWRC-Ct M209 04|United States.Pennsylvania
Centroptilum triangulifer|SWRC-Cts M2 01|United States.Pennsylvania
Baetis phoebus|SWA iBOL UofS 105|Canada.Saskatchewan
Baetis phoebus|SWA iBOL UofS 103|Canada.Saskatchewan
Baetis phoebus|SWA iBOL UofS 102|Canada.Saskatchewan
Baetis phoebus|SWA iBOL UofS 97|Canada.Saskatchewan
Baetis phoebus|SWA iBOL UofS 100|Canada.Saskatchewan
Baetis phoebus|SWA iBOL UofS 99|Canada.Saskatchewan
Baetis phoebus|07PROBE-2368|Canada.Manitoba
Baetis phoebus|07PROBE-2367|Canada.Manitoba
Baetis phoebus|07PROBE-2366|Canada.Manitoba
Baetis phoebus|07PROBE-2365|Canada.Manitoba
Baetis phoebus|07PROBE-2364|Canada.Manitoba
Baetis phoebus|07PROBE-2363|Canada.Manitoba
Baetis phoebus|07PROBE-2362|Canada.Manitoba
Baetis phoebus|07PROBE-2361|Canada.Manitoba
Baetis phoebus|07PROBE-2360|Canada.Manitoba
Baetis phoebus|07PROBE-2359|Canada.Manitoba
Baetis phoebus|07PROBE-2358|Canada.Manitoba
Baetis phoebus|07PROBE-2357|Canada.Manitoba
Baetis phoebus|07PROBE-2356|Canada.Manitoba
Baetis phoebus|07PROBE-2355|Canada.Manitoba
Baetis phoebus|07PROBE-2354|Canada.Manitoba
Baetis phoebus|07PROBE-2353|Canada.Manitoba
Baetis phoebus|07PROBE-2352|Canada.Manitoba
Baetis phoebus|07PROBE-2351|Canada.Manitoba
Baetis phoebus|07PROBE-2350|Canada.Manitoba
Baetis phoebus|07PROBE-2349|Canada.Manitoba
Baetis phoebus|07PROBE-2348|Canada.Manitoba
Baetis phoebus|07PROBE-2347|Canada.Manitoba
Baetis phoebus|07PROBE-2346|Canada.Manitoba
Baetis phoebus|07PROBE-2345|Canada.Manitoba
Baetis phoebus|07PROBE-2344|Canada.Manitoba
Baetis phoebus|07PROBE-2342|Canada.Manitoba
Baetis phoebus|07PROBE-2314|Canada.Manitoba
Baetis phoebus|07PROBE-2048|Canada.Manitoba
Baetis phoebus|07PROBE-07054|Canada.Manitoba
Baetis phoebus|07PROBE-2043|Canada.Manitoba
Baetis phoebus|07PROBE-07094|Canada.Manitoba
Baetis phoebus|07PROBE-2308|Canada.Manitoba
Baetis phoebus|07PROBE-2303|Canada.Manitoba
Baetis phoebus|07PROBE-2196|Canada.Manitoba
Baetis phoebus|07PROBE-2293|Canada.Manitoba
Baetis phoebus|07PROBE-2212|Canada.Manitoba
Baetis phoebus|07PROBE-2200|Canada.Manitoba
Baetis phoebus|07PROBE-2070|Canada.Manitoba
Baetis phoebus|07PROBE-2117|Canada.Manitoba
Baetis phoebus|07PROBE-2197|Canada.Manitoba
Baetis phoebus|07PROBE-2226|Canada.Manitoba
Baetis phoebus|07PROBE-2281|Canada.Manitoba
Baetis phoebus|07PROBE-2014|Canada.Manitoba
Baetis phoebus|07PROBE-2042|Canada.Manitoba
Baetis phoebus|07PROBE-2045|Canada.Manitoba
Baetis phoebus|07PROBE-2046|Canada.Manitoba
Baetis phoebus|07PROBE-2047|Canada.Manitoba
Baetis phoebus|07PROBE-2049|Canada.Manitoba
Baetis phoebus|07PROBE-2050|Canada.Manitoba
Procloeon sp.JMW3
Procloeon intermediale
Procloeon rivulare
Procloeon sp.LJ1
Procloeon pennulatum
Centroptilum triangulifer
Centroptilum alamance
Acentrella parvula
Centroptilum triangulifer

[illegible]
Baetis phoebus|07PROBE-2049|Canada.Manitoba
Baetis phoebus|07PROBE-2050|Canada.Manitoba
Baetis phoebus|07PROBE-2063|Canada.Manitoba
Baetis phoebus|07PROBE-2067|Canada.Manitoba
Baetis phoebus|07PROBE-2068|Canada.Manitoba
Baetis phoebus|07PROBE-2074|Canada.Manitoba
Baetis phoebus|07PROBE-2115|Canada.Manitoba
Baetis phoebus|07PROBE-2118|Canada.Manitoba
Baetis phoebus|07PROBE-2120|Canada.Manitoba
Baetis phoebus|07PROBE-2124|Canada.Manitoba
Baetis phoebus|07PROBE-2127|Canada.Manitoba
Baetis phoebus|07PROBE-2128|Canada.Manitoba
Baetis phoebus|07PROBE-2136|Canada.Manitoba
Baetis phoebus|07PROBE-2137|Canada.Manitoba
Baetis phoebus|07PROBE-2138|Canada.Manitoba
Baetis phoebus|07PROBE-2144|Canada.Manitoba
Baetis phoebus|07PROBE-2146|Canada.Manitoba
Baetis phoebus|07PROBE-2149|Canada.Manitoba
Baetis phoebus|07PROBE-2151|Canada.Manitoba
Baetis phoebus|07PROBE-2153|Canada.Manitoba
Baetis phoebus|07PROBE-2159|Canada.Manitoba
Baetis phoebus|07PROBE-2160|Canada.Manitoba
Baetis phoebus|07PROBE-2161|Canada.Manitoba
Baetis phoebus|07PROBE-2172|Canada.Manitoba
Baetis phoebus|07PROBE-2185|Canada.Manitoba
Baetis phoebus|07PROBE-2187|Canada.Manitoba
Baetis phoebus|07PROBE-2188|Canada.Manitoba
Baetis phoebus|07PROBE-2189|Canada.Manitoba
Baetis phoebus|07PROBE-2191|Canada.Manitoba
Baetis phoebus|07PROBE-2193|Canada.Manitoba
Baetis phoebus|07PROBE-2198|Canada.Manitoba
Baetis phoebus|07PROBE-2199|Canada.Manitoba
Baetis phoebus|07PROBE-2201|Canada.Manitoba
Baetis phoebus|07PROBE-2202|Canada.Manitoba
Baetis phoebus|07PROBE-2203|Canada.Manitoba
Baetis phoebus|07PROBE-2204|Canada.Manitoba
Baetis phoebus|07PROBE-2205|Canada.Manitoba
Baetis phoebus|07PROBE-2206|Canada.Manitoba
Baetis phoebus|07PROBE-2207|Canada.Manitoba
Baetis phoebus|07PROBE-2208|Canada.Manitoba
Baetis phoebus|07PROBE-2209|Canada.Manitoba
Baetis phoebus|07PROBE-2210|Canada.Manitoba
Baetis phoebus|07PROBE-2211|Canada.Manitoba
Baetis phoebus|07PROBE-2213|Canada.Manitoba
Baetis phoebus|07PROBE-2218|Canada.Manitoba
Baetis phoebus|07PROBE-2221|Canada.Manitoba
Baetis phoebus|07PROBE-2223|Canada.Manitoba
Baetis phoebus|07PROBE-2224|Canada.Manitoba
Baetis phoebus|07PROBE-2225|Canada.Manitoba
Baetis phoebus|07PROBE-2227|Canada.Manitoba
Baetis phoebus|07PROBE-2228|Canada.Manitoba
Baetis phoebus|07PROBE-2229|Canada.Manitoba
Baetis phoebus|07PROBE-2230|Canada.Manitoba
Baetis phoebus|07PROBE-2232|Canada.Manitoba
Baetis phoebus|07PROBE-2233|Canada.Manitoba
Baetis phoebus|07PROBE-2234|Canada.Manitoba
Baetis phoebus
Baetis phoebus|07PROBE-2235|Canada.Manitoba
Baetis phoebus|07PROBE-2237|Canada.Manitoba
Baetis phoebus|07PROBE-2238|Canada.Manitoba
Baetis phoebus|07PROBE-2258|Canada.Manitoba
Baetis phoebus|07PROBE-2261|Canada.Manitoba
Baetis phoebus|07PROBE-2262|Canada.Manitoba
Baetis phoebus|07PROBE-2264|Canada.Manitoba
Baetis phoebus|07PROBE-2265|Canada.Manitoba
Baetis phoebus|07PROBE-2269|Canada.Manitoba
Baetis phoebus|07PROBE-2270|Canada.Manitoba
Baetis phoebus|07PROBE-2271|Canada.Manitoba
Baetis phoebus|07PROBE-2272|Canada.Manitoba
Baetis phoebus|07PROBE-2273|Canada.Manitoba
Baetis phoebus|07PROBE-2275|Canada.Manitoba
Baetis phoebus|07PROBE-07022|Canada.Manitoba
Baetis phoebus|07PROBE-07045|Canada.Manitoba
Baetis phoebus|07PROBE-07052|Canada.Manitoba
Baetis phoebus|07PROBE-07053|Canada.Manitoba
Baetis phoebus|07PROBE-07055|Canada.Manitoba
Baetis phoebus|07PROBE-07056|Canada.Manitoba
Baetis phoebus|07PROBE-07074|Canada.Manitoba
Baetis phoebus|07PROBE-07081|Canada.Manitoba
Baetis phoebus|07PROBE-07087|Canada.Manitoba
Baetis phoebus|07PROBE-07088|Canada.Manitoba
Baetis phoebus|07PROBE-07090|Canada.Manitoba
Baetis phoebus|07PROBE-07095|Canada.Manitoba
Baetis phoebus|07PROBE-07099|Canada.Manitoba
Baetis phoebus|07PROBE-07100|Canada.Manitoba
Baetis phoebus|07PROBE-07105|Canada.Manitoba
Baetis phoebus|07PROBE-07108|Canada.Manitoba
Baetis phoebus|07PROBE-2287|Canada.Manitoba
Baetis phoebus|07PROBE-2289|Canada.Manitoba
Baetis phoebus|07PROBE-2291|Canada.Manitoba
Baetis phoebus|07PROBE-2292|Canada.Manitoba
Baetis phoebus|07PROBE-2294|Canada.Manitoba
Baetis phoebus|07PROBE-2295|Canada.Manitoba
Baetis phoebus|07PROBE-2296|Canada.Manitoba
Baetis phoebus|07PROBE-2297|Canada.Manitoba
Baetis phoebus|07PROBE-2298|Canada.Manitoba

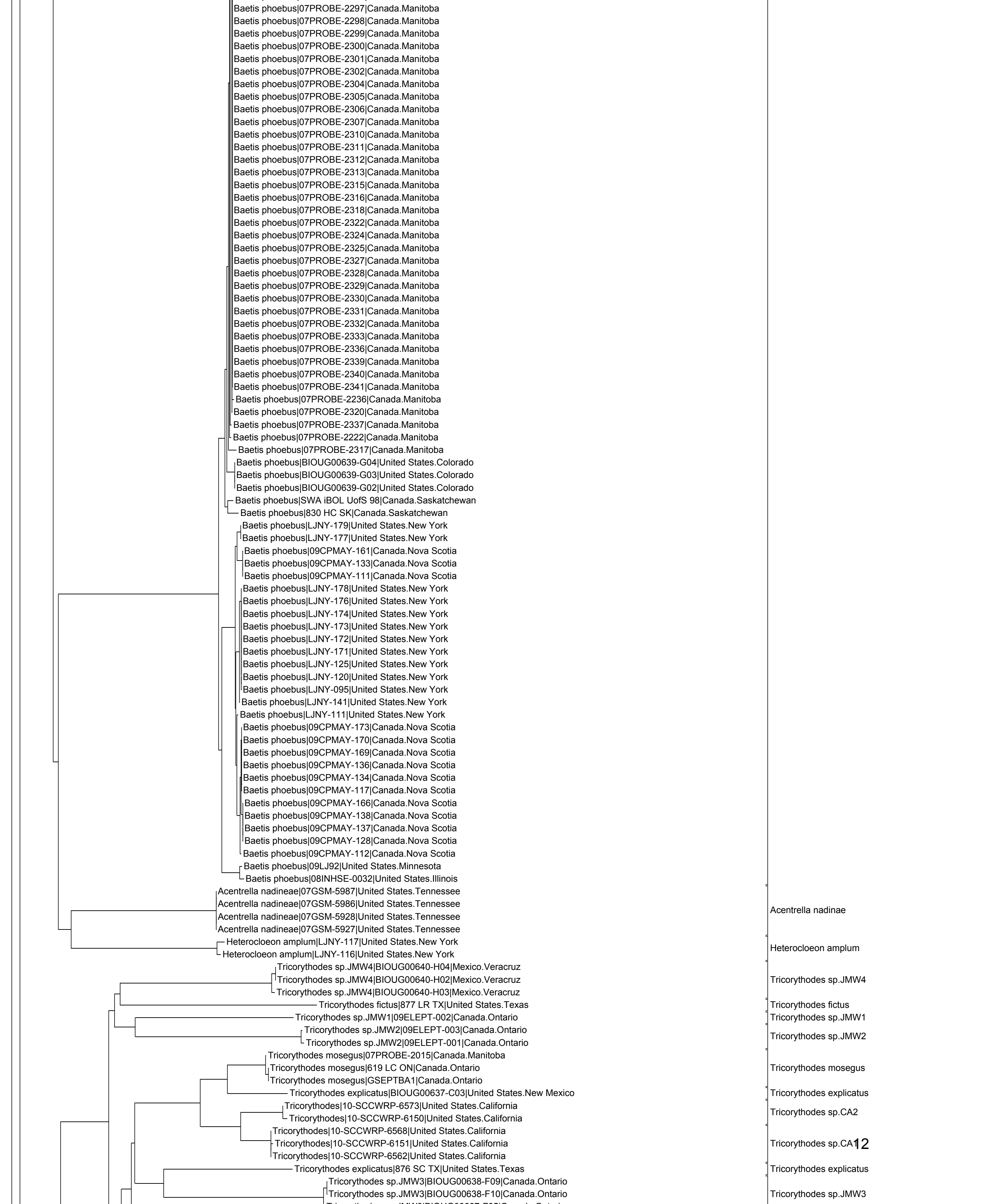
Baetis phoebus|07PROBE-2297|Canada.Manitoba
Baetis phoebus|07PROBE-2298|Canada.Manitoba
Baetis phoebus|07PROBE-2299|Canada.Manitoba
Baetis phoebus|07PROBE-2300|Canada.Manitoba
Baetis phoebus|07PROBE-2301|Canada.Manitoba
Baetis phoebus|07PROBE-2302|Canada.Manitoba
Baetis phoebus|07PROBE-2304|Canada.Manitoba
Baetis phoebus|07PROBE-2305|Canada.Manitoba
Baetis phoebus|07PROBE-2306|Canada.Manitoba
Baetis phoebus|07PROBE-2307|Canada.Manitoba
Baetis phoebus|07PROBE-2310|Canada.Manitoba
Baetis phoebus|07PROBE-2311|Canada.Manitoba
Baetis phoebus|07PROBE-2312|Canada.Manitoba
Baetis phoebus|07PROBE-2313|Canada.Manitoba
Baetis phoebus|07PROBE-2315|Canada.Manitoba
Baetis phoebus|07PROBE-2316|Canada.Manitoba
Baetis phoebus|07PROBE-2318|Canada.Manitoba
Baetis phoebus|07PROBE-2322|Canada.Manitoba
Baetis phoebus|07PROBE-2324|Canada.Manitoba
Baetis phoebus|07PROBE-2325|Canada.Manitoba
Baetis phoebus|07PROBE-2327|Canada.Manitoba
Baetis phoebus|07PROBE-2328|Canada.Manitoba
Baetis phoebus|07PROBE-2329|Canada.Manitoba
Baetis phoebus|07PROBE-2330|Canada.Manitoba
Baetis phoebus|07PROBE-2331|Canada.Manitoba
Baetis phoebus|07PROBE-2332|Canada.Manitoba
Baetis phoebus|07PROBE-2333|Canada.Manitoba
Baetis phoebus|07PROBE-2336|Canada.Manitoba
Baetis phoebus|07PROBE-2339|Canada.Manitoba
Baetis phoebus|07PROBE-2340|Canada.Manitoba
Baetis phoebus|07PROBE-2341|Canada.Manitoba
Baetis phoebus|07PROBE-2236|Canada.Manitoba
Baetis phoebus|07PROBE-2320|Canada.Manitoba
Baetis phoebus|07PROBE-2337|Canada.Manitoba
Baetis phoebus|07PROBE-2222|Canada.Manitoba
Baetis phoebus|07PROBE-2317|Canada.Manitoba
Baetis phoebus|BIOUG00639-G04|United States.Colorado
Baetis phoebus|BIOUG00639-G03|United States.Colorado
Baetis phoebus|BIOUG00639-G02|United States.Colorado
Baetis phoebus|SWA iBOL UofS 98|Canada.Saskatchewan
Baetis phoebus|830 HC SK|Canada.Saskatchewan
Baetis phoebus|LJNY-179|United States.New York
Baetis phoebus|LJNY-177|United States.New York
Baetis phoebus|09CPMAY-161|Canada.Nova Scotia
Baetis phoebus|09CPMAY-133|Canada.Nova Scotia
Baetis phoebus|09CPMAY-111|Canada.Nova Scotia
Baetis phoebus|LJNY-178|United States.New York
Baetis phoebus|LJNY-176|United States.New York
Baetis phoebus|LJNY-174|United States.New York
Baetis phoebus|LJNY-173|United States.New York
Baetis phoebus|LJNY-172|United States.New York
Baetis phoebus|LJNY-171|United States.New York
Baetis phoebus|LJNY-125|United States.New York
Baetis phoebus|LJNY-120|United States.New York
Baetis phoebus|LJNY-095|United States.New York
Baetis phoebus|LJNY-141|United States.New York
Baetis phoebus|LJNY-111|United States.New York
Baetis phoebus|09CPMAY-173|Canada.Nova Scotia
Baetis phoebus|09CPMAY-170|Canada.Nova Scotia
Baetis phoebus|09CPMAY-169|Canada.Nova Scotia
Baetis phoebus|09CPMAY-136|Canada.Nova Scotia
Baetis phoebus|09CPMAY-134|Canada.Nova Scotia
Baetis phoebus|09CPMAY-117|Canada.Nova Scotia
Baetis phoebus|09CPMAY-166|Canada.Nova Scotia
Baetis phoebus|09CPMAY-138|Canada.Nova Scotia
Baetis phoebus|09CPMAY-137|Canada.Nova Scotia
Baetis phoebus|09CPMAY-128|Canada.Nova Scotia
Baetis phoebus|09CPMAY-112|Canada.Nova Scotia
Baetis phoebus|09LJ92|United States.Minnesota
Baetis phoebus|08INHSE-0032|United States.Illinois
Acentrella nadineae|07GSM-5987|United States.Tennessee
Acentrella nadineae|07GSM-5986|United States.Tennessee
Acentrella nadineae|07GSM-5928|United States.Tennessee
Acentrella nadineae|07GSM-5927|United States.Tennessee
Acentrella nadinae
Heterocloeon amplum|LJNY-117|United States.New York
Heterocloeon amplum|LJNY-116|United States.New York
Heterocloeon amplum
Tricorythodes sp.JMW4|BIOUG00640-H04|Mexico.Veracruz
Tricorythodes sp.JMW4|BIOUG00640-H02|Mexico.Veracruz
Tricorythodes sp.JMW4|BIOUG00640-H03|Mexico.Veracruz
Tricorythodes sp.JMW4
Tricorythodes fictus|877 LR TX|United States.Texas
Tricorythodes fictus
Tricorythodes sp.JMW1|09ELEPT-002|Canada.Ontario
Tricorythodes sp.JMW1
Tricorythodes sp.JMW2|09ELEPT-003|Canada.Ontario
Tricorythodes sp.JMW2|09ELEPT-001|Canada.Ontario
Tricorythodes sp.JMW2
Tricorythodes mosegus|07PROBE-2015|Canada.Manitoba
Tricorythodes mosegus|619 LC ON|Canada.Ontario
Tricorythodes mosegus|GSEPTBA1|Canada.Ontario
Tricorythodes mosegus
Tricorythodes explicatus|BIOUG00637-C03|United States.New Mexico
Tricorythodes explicatus
Tricorythodes|10-SCCWRP-6573|United States.California
Tricorythodes|10-SCCWRP-6150|United States.California
Tricorythodes sp.CA2
Tricorythodes|10-SCCWRP-6568|United States.California
Tricorythodes|10-SCCWRP-6151|United States.California
Tricorythodes|10-SCCWRP-6562|United States.California
Tricorythodes sp.CA1
Tricorythodes explicatus|876 SC TX|United States.Texas
Tricorythodes explicatus
Tricorythodes sp.JMW3|BIOUG00638-F09|Canada.Ontario
Tricorythodes sp.JMW3|BIOUG00638-F10|Canada.Ontario
Tricorythodes sp.JMW3

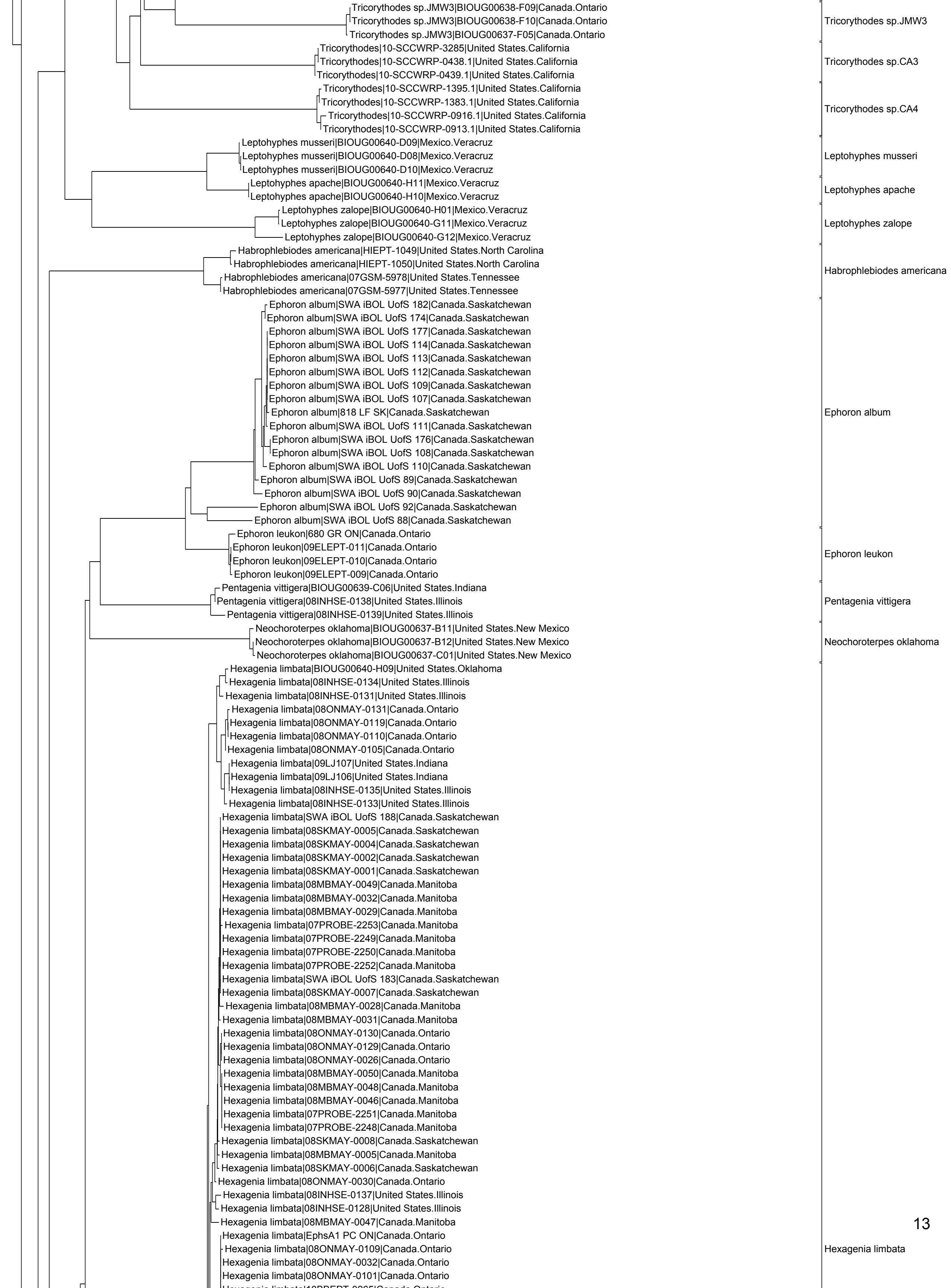

Tricorythodes sp.JMW3|BIOUG00638-F09|Canada.Ontario
Tricorythodes sp.JMW3|BIOUG00638-F10|Canada.Ontario
Tricorythodes sp.JMW3|BIOUG00637-F05|Canada.Ontario
Tricorythodes|10-SCCWRP-3285|United States.California
Tricorythodes|10-SCCWRP-0438.1|United States.California
Tricorythodes|10-SCCWRP-0439.1|United States.California
Tricorythodes|10-SCCWRP-1395.1|United States.California
Tricorythodes|10-SCCWRP-1383.1|United States.California
Tricorythodes|10-SCCWRP-0916.1|United States.California
Tricorythodes|10-SCCWRP-0913.1|United States.California
Leptohyphes musseri|BIOUG00640-D09|Mexico.Veracruz
Leptohyphes musseri|BIOUG00640-D08|Mexico.Veracruz
Leptohyphes musseri|BIOUG00640-D10|Mexico.Veracruz
Leptohyphes apache|BIOUG00640-H11|Mexico.Veracruz
Leptohyphes apache|BIOUG00640-H10|Mexico.Veracruz
Leptohyphes zalope|BIOUG00640-H01|Mexico.Veracruz
Leptohyphes zalope|BIOUG00640-G11|Mexico.Veracruz
Leptohyphes zalope|BIOUG00640-G12|Mexico.Veracruz
Habrophlebiodes americana|HIEPT-1049|United States.North Carolina
Habrophlebiodes americana|HIEPT-1050|United States.North Carolina
Habrophlebiodes americana|07GSM-5978|United States.Tennessee
Habrophlebiodes americana|07GSM-5977|United States.Tennessee
Ephoron album|SWA iBOL UofS 182|Canada.Saskatchewan
Ephoron album|SWA iBOL UofS 174|Canada.Saskatchewan
Ephoron album|SWA iBOL UofS 177|Canada.Saskatchewan
Ephoron album|SWA iBOL UofS 114|Canada.Saskatchewan
Ephoron album|SWA iBOL UofS 113|Canada.Saskatchewan
Ephoron album|SWA iBOL UofS 112|Canada.Saskatchewan
Ephoron album|SWA iBOL UofS 109|Canada.Saskatchewan
Ephoron album|SWA iBOL UofS 107|Canada.Saskatchewan
Ephoron album|818 LF SK|Canada.Saskatchewan
Ephoron album|SWA iBOL UofS 111|Canada.Saskatchewan
Ephoron album|SWA iBOL UofS 176|Canada.Saskatchewan
Ephoron album|SWA iBOL UofS 108|Canada.Saskatchewan
Ephoron album|SWA iBOL UofS 110|Canada.Saskatchewan
Ephoron album|SWA iBOL UofS 89|Canada.Saskatchewan
Ephoron album|SWA iBOL UofS 90|Canada.Saskatchewan
Ephoron album|SWA iBOL UofS 92|Canada.Saskatchewan
Ephoron album|SWA iBOL UofS 88|Canada.Saskatchewan
Ephoron leukon|680 GR ON|Canada.Ontario
Ephoron leukon|09ELEPT-011|Canada.Ontario
Ephoron leukon|09ELEPT-010|Canada.Ontario
Ephoron leukon|09ELEPT-009|Canada.Ontario
Pentagenia vittigera|BIOUG00639-C06|United States.Indiana
Pentagenia vittigera|08INHSE-0138|United States.Illinois
Pentagenia vittigera|08INHSE-0139|United States.Illinois
Neochoroterpes oklahoma|BIOUG00637-B11|United States.New Mexico
Neochoroterpes oklahoma|BIOUG00637-B12|United States.New Mexico
Neochoroterpes oklahoma|BIOUG00637-C01|United States.New Mexico
Hexagenia limbata|BIOUG00640-H09|United States.Oklahoma
Hexagenia limbata|08INHSE-0134|United States.Illinois
Hexagenia limbata|08INHSE-0131|United States.Illinois
Hexagenia limbata|08ONMAY-0131|Canada.Ontario
Hexagenia limbata|08ONMAY-0119|Canada.Ontario
Hexagenia limbata|08ONMAY-0110|Canada.Ontario
Hexagenia limbata|08ONMAY-0105|Canada.Ontario
Hexagenia limbata|09LJ107|United States.Indiana
Hexagenia limbata|09LJ106|United States.Indiana
Hexagenia limbata|08INHSE-0135|United States.Illinois
Hexagenia limbata|08INHSE-0133|United States.Illinois
Hexagenia limbata|SWA iBOL UofS 188|Canada.Saskatchewan
Hexagenia limbata|08SKMAY-0005|Canada.Saskatchewan
Hexagenia limbata|08SKMAY-0004|Canada.Saskatchewan
Hexagenia limbata|08SKMAY-0002|Canada.Saskatchewan
Hexagenia limbata|08SKMAY-0001|Canada.Saskatchewan
Hexagenia limbata|08MBMAY-0049|Canada.Manitoba
Hexagenia limbata|08MBMAY-0032|Canada.Manitoba
Hexagenia limbata|08MBMAY-0029|Canada.Manitoba
Hexagenia limbata|07PROBE-2253|Canada.Manitoba
Hexagenia limbata|07PROBE-2249|Canada.Manitoba
Hexagenia limbata|07PROBE-2250|Canada.Manitoba
Hexagenia limbata|07PROBE-2252|Canada.Manitoba
Hexagenia limbata|SWA iBOL UofS 183|Canada.Saskatchewan
Hexagenia limbata|08SKMAY-0007|Canada.Saskatchewan
Hexagenia limbata|08MBMAY-0028|Canada.Manitoba
Hexagenia limbata|08MBMAY-0031|Canada.Manitoba
Hexagenia limbata|08ONMAY-0130|Canada.Ontario
Hexagenia limbata|08ONMAY-0129|Canada.Ontario
Hexagenia limbata|08ONMAY-0026|Canada.Ontario
Hexagenia limbata|08MBMAY-0050|Canada.Manitoba
Hexagenia limbata|08MBMAY-0048|Canada.Manitoba
Hexagenia limbata|08MBMAY-0046|Canada.Manitoba
Hexagenia limbata|07PROBE-2251|Canada.Manitoba
Hexagenia limbata|07PROBE-2248|Canada.Manitoba
Hexagenia limbata|08SKMAY-0008|Canada.Saskatchewan
Hexagenia limbata|08MBMAY-0005|Canada.Manitoba
Hexagenia limbata|08SKMAY-0006|Canada.Saskatchewan
Hexagenia limbata|08ONMAY-0030|Canada.Ontario
Hexagenia limbata|08INHSE-0137|United States.Illinois
Hexagenia limbata|08INHSE-0128|United States.Illinois
Hexagenia limbata|08MBMAY-0047|Canada.Manitoba
Hexagenia limbata|EphsA1 PC ON|Canada.Ontario
Hexagenia limbata|08ONMAY-0109|Canada.Ontario
Hexagenia limbata|08ONMAY-0032|Canada.Ontario
Hexagenia limbata|08ONMAY-0101|Canada.Ontario
Tricorythodes sp.JMW3
Tricorythodes sp.CA3
Tricorythodes sp.CA4
Leptohyphes musseri
Leptohyphes apache
Leptohyphes zalope
Habrophlebiodes americana
Ephoron album
Ephoron leukon
Pentagenia vittigera
Neochoroterpes oklahoma
Hexagenia limbata

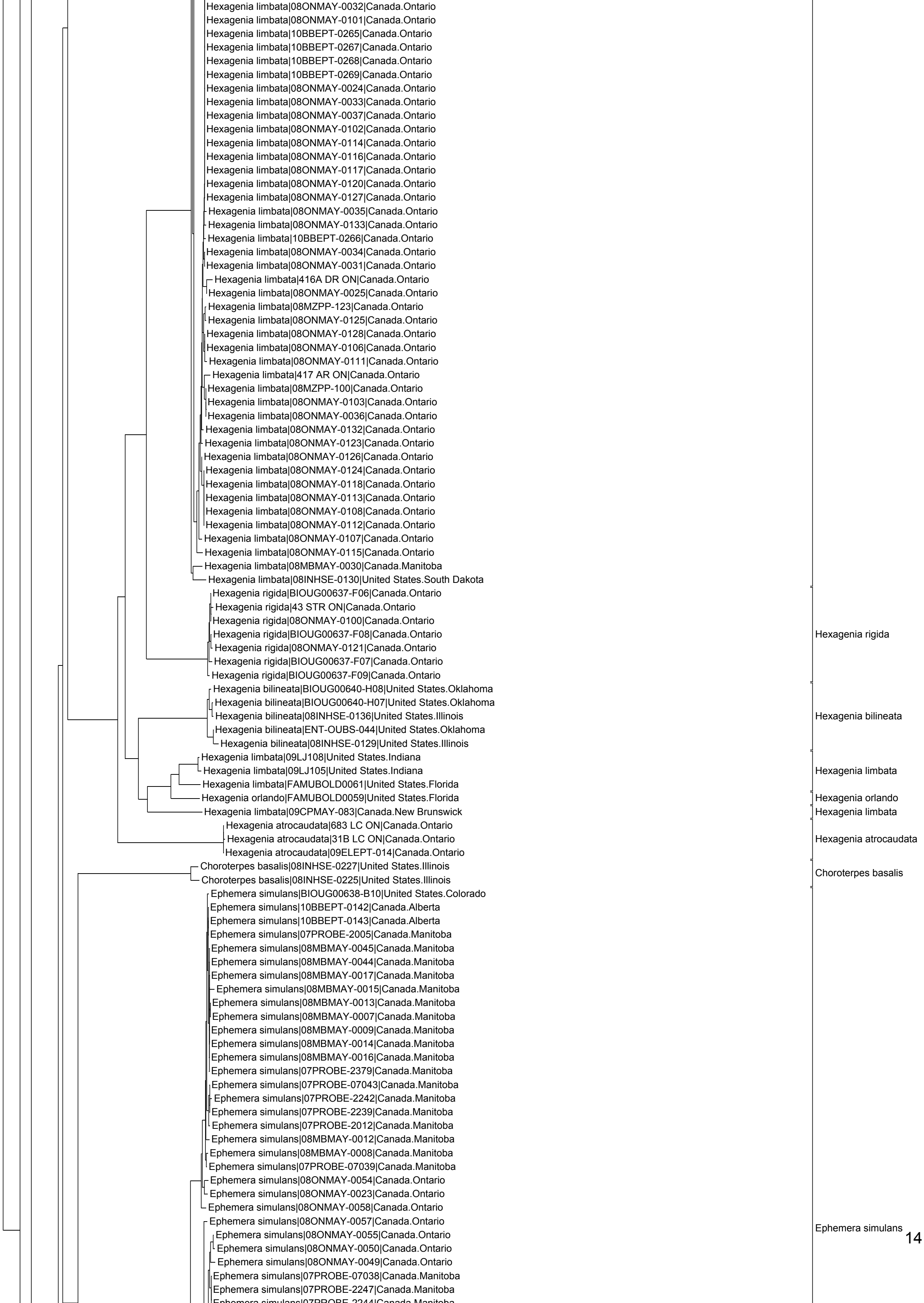
Hexagenia limbata|08ONMAY-0032|Canada.Ontario
Hexagenia limbata|08ONMAY-0101|Canada.Ontario
Hexagenia limbata|10BBEPT-0265|Canada.Ontario
Hexagenia limbata|10BBEPT-0267|Canada.Ontario
Hexagenia limbata|10BBEPT-0268|Canada.Ontario
Hexagenia limbata|10BBEPT-0269|Canada.Ontario
Hexagenia limbata|08ONMAY-0024|Canada.Ontario
Hexagenia limbata|08ONMAY-0033|Canada.Ontario
Hexagenia limbata|08ONMAY-0037|Canada.Ontario
Hexagenia limbata|08ONMAY-0102|Canada.Ontario
Hexagenia limbata|08ONMAY-0114|Canada.Ontario
Hexagenia limbata|08ONMAY-0116|Canada.Ontario
Hexagenia limbata|08ONMAY-0117|Canada.Ontario
Hexagenia limbata|08ONMAY-0120|Canada.Ontario
Hexagenia limbata|08ONMAY-0127|Canada.Ontario
Hexagenia limbata|08ONMAY-0035|Canada.Ontario
Hexagenia limbata|08ONMAY-0133|Canada.Ontario
Hexagenia limbata|10BBEPT-0266|Canada.Ontario
Hexagenia limbata|08ONMAY-0034|Canada.Ontario
Hexagenia limbata|08ONMAY-0031|Canada.Ontario
Hexagenia limbata|416A DR ON|Canada.Ontario
Hexagenia limbata|08ONMAY-0025|Canada.Ontario
Hexagenia limbata|08MZPP-123|Canada.Ontario
Hexagenia limbata|08ONMAY-0125|Canada.Ontario
Hexagenia limbata|08ONMAY-0128|Canada.Ontario
Hexagenia limbata|08ONMAY-0106|Canada.Ontario
Hexagenia limbata|08ONMAY-0111|Canada.Ontario
Hexagenia limbata|417 AR ON|Canada.Ontario
Hexagenia limbata|08MZPP-100|Canada.Ontario
Hexagenia limbata|08ONMAY-0103|Canada.Ontario
Hexagenia limbata|08ONMAY-0036|Canada.Ontario
Hexagenia limbata|08ONMAY-0132|Canada.Ontario
Hexagenia limbata|08ONMAY-0123|Canada.Ontario
Hexagenia limbata|08ONMAY-0126|Canada.Ontario
Hexagenia limbata|08ONMAY-0124|Canada.Ontario
Hexagenia limbata|08ONMAY-0118|Canada.Ontario
Hexagenia limbata|08ONMAY-0113|Canada.Ontario
Hexagenia limbata|08ONMAY-0108|Canada.Ontario
Hexagenia limbata|08ONMAY-0112|Canada.Ontario
Hexagenia limbata|08ONMAY-0107|Canada.Ontario
Hexagenia limbata|08ONMAY-0115|Canada.Ontario
Hexagenia limbata|08MBMAY-0030|Canada.Manitoba
Hexagenia limbata|08INHSE-0130|United States.South Dakota
Hexagenia rigida|BIOUG00637-F06|Canada.Ontario
Hexagenia rigida|43 STR ON|Canada.Ontario
Hexagenia rigida|08ONMAY-0100|Canada.Ontario
Hexagenia rigida|BIOUG00637-F08|Canada.Ontario
Hexagenia rigida|08ONMAY-0121|Canada.Ontario
Hexagenia rigida|BIOUG00637-F07|Canada.Ontario
Hexagenia rigida|BIOUG00637-F09|Canada.Ontario
Hexagenia bilineata|BIOUG00640-H08|United States.Oklahoma
Hexagenia bilineata|BIOUG00640-H07|United States.Oklahoma
Hexagenia bilineata|08INHSE-0136|United States.Illinois
Hexagenia bilineata|ENT-OUBS-044|United States.Oklahoma
Hexagenia bilineata|08INHSE-0129|United States.Illinois
Hexagenia limbata|09LJ108|United States.Indiana
Hexagenia limbata|09LJ105|United States.Indiana
Hexagenia limbata|FAMUBOLD0061|United States.Florida
Hexagenia orlando|FAMUBOLD0059|United States.Florida
Hexagenia limbata|09CPMAY-083|Canada.New Brunswick
Hexagenia atrocaudata|683 LC ON|Canada.Ontario
Hexagenia atrocaudata|31B LC ON|Canada.Ontario
Hexagenia atrocaudata|09ELEPT-014|Canada.Ontario
Choroterpes basalis|08INHSE-0227|United States.Illinois
Choroterpes basalis|08INHSE-0225|United States.Illinois
Ephemera simulans|BIOUG00638-B10|United States.Colorado
Ephemera simulans|10BBEPT-0142|Canada.Alberta
Ephemera simulans|10BBEPT-0143|Canada.Alberta
Ephemera simulans|07PROBE-2005|Canada.Manitoba
Ephemera simulans|08MBMAY-0045|Canada.Manitoba
Ephemera simulans|08MBMAY-0044|Canada.Manitoba
Ephemera simulans|08MBMAY-0017|Canada.Manitoba
Ephemera simulans|08MBMAY-0015|Canada.Manitoba
Ephemera simulans|08MBMAY-0013|Canada.Manitoba
Ephemera simulans|08MBMAY-0007|Canada.Manitoba
Ephemera simulans|08MBMAY-0009|Canada.Manitoba
Ephemera simulans|08MBMAY-0014|Canada.Manitoba
Ephemera simulans|08MBMAY-0016|Canada.Manitoba
Ephemera simulans|07PROBE-2379|Canada.Manitoba
Ephemera simulans|07PROBE-07043|Canada.Manitoba
Ephemera simulans|07PROBE-2242|Canada.Manitoba
Ephemera simulans|07PROBE-2239|Canada.Manitoba
Ephemera simulans|07PROBE-2012|Canada.Manitoba
Ephemera simulans|08MBMAY-0012|Canada.Manitoba
Ephemera simulans|08MBMAY-0008|Canada.Manitoba
Ephemera simulans|07PROBE-07039|Canada.Manitoba
Ephemera simulans|08ONMAY-0054|Canada.Ontario
Ephemera simulans|08ONMAY-0023|Canada.Ontario
Ephemera simulans|08ONMAY-0058|Canada.Ontario
Ephemera simulans|08ONMAY-0057|Canada.Ontario
Ephemera simulans|08ONMAY-0055|Canada.Ontario
Ephemera simulans|08ONMAY-0050|Canada.Ontario
Ephemera simulans|08ONMAY-0049|Canada.Ontario
Ephemera simulans|07PROBE-07038|Canada.Manitoba
Ephemera simulans|07PROBE-2247|Canada.Manitoba
Hexagenia rigida
Hexagenia bilineata
Hexagenia limbata
Hexagenia orlando
Hexagenia limbata
Hexagenia atrocaudata
Choroterpes basalis
Ephemera simulans

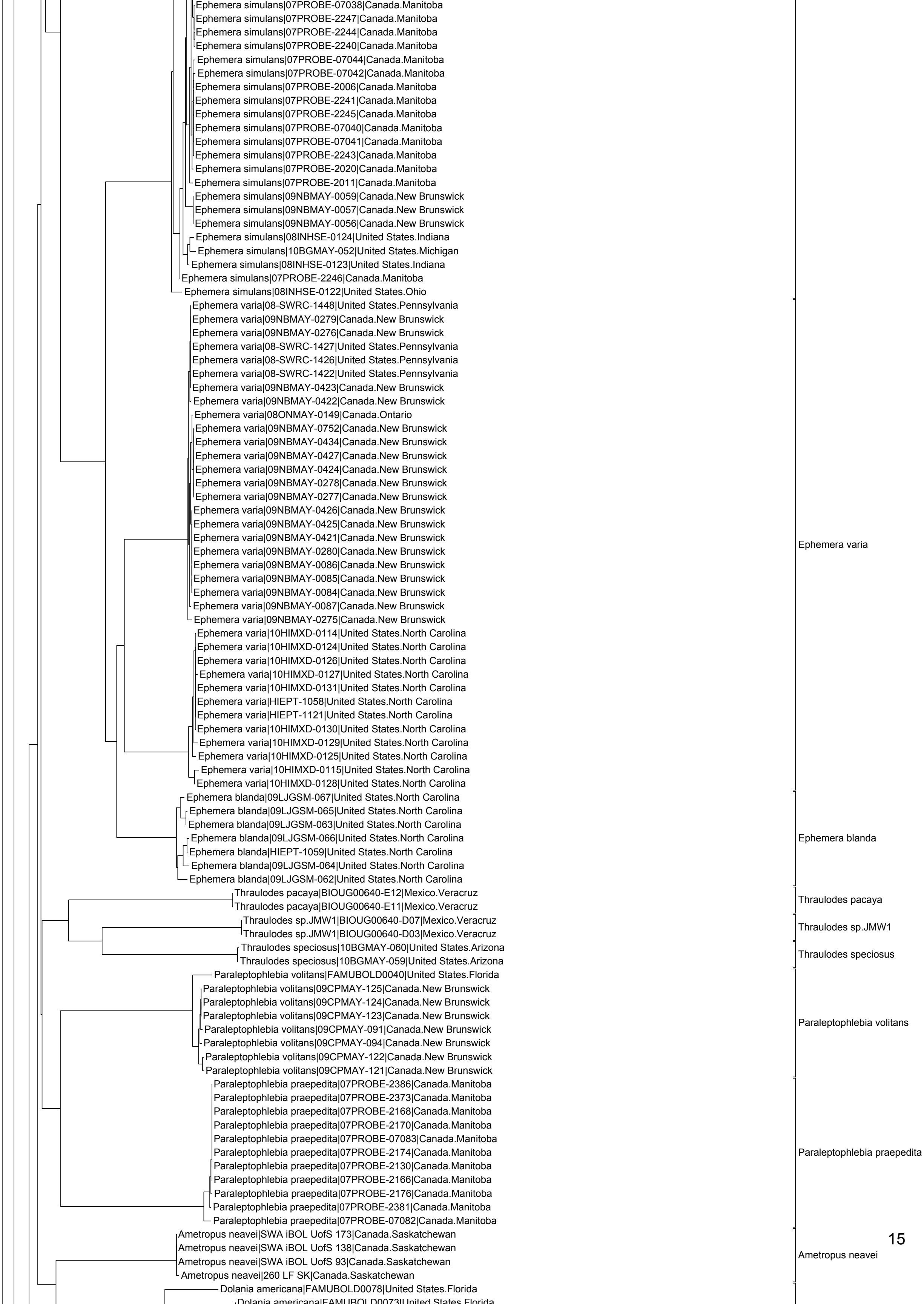
Ephemera simulans|07PROBE-07038|Canada.Manitoba
Ephemera simulans|07PROBE-2247|Canada.Manitoba
Ephemera simulans|07PROBE-2244|Canada.Manitoba
Ephemera simulans|07PROBE-2240|Canada.Manitoba
Ephemera simulans|07PROBE-07044|Canada.Manitoba
Ephemera simulans|07PROBE-07042|Canada.Manitoba
Ephemera simulans|07PROBE-2006|Canada.Manitoba
Ephemera simulans|07PROBE-2241|Canada.Manitoba
Ephemera simulans|07PROBE-2245|Canada.Manitoba
Ephemera simulans|07PROBE-07040|Canada.Manitoba
Ephemera simulans|07PROBE-07041|Canada.Manitoba
Ephemera simulans|07PROBE-2243|Canada.Manitoba
Ephemera simulans|07PROBE-2020|Canada.Manitoba
Ephemera simulans|07PROBE-2011|Canada.Manitoba
Ephemera simulans|09NBMAY-0059|Canada.New Brunswick
Ephemera simulans|09NBMAY-0057|Canada.New Brunswick
Ephemera simulans|09NBMAY-0056|Canada.New Brunswick
Ephemera simulans|08INHSE-0124|United States.Indiana
Ephemera simulans|10BGMAY-052|United States.Michigan
Ephemera simulans|08INHSE-0123|United States.Indiana
Ephemera simulans|07PROBE-2246|Canada.Manitoba
Ephemera simulans|08INHSE-0122|United States.Ohio
Ephemera varia|08-SWRC-1448|United States.Pennsylvania
Ephemera varia|09NBMAY-0279|Canada.New Brunswick
Ephemera varia|09NBMAY-0276|Canada.New Brunswick
Ephemera varia|08-SWRC-1427|United States.Pennsylvania
Ephemera varia|08-SWRC-1426|United States.Pennsylvania
Ephemera varia|08-SWRC-1422|United States.Pennsylvania
Ephemera varia|09NBMAY-0423|Canada.New Brunswick
Ephemera varia|09NBMAY-0422|Canada.New Brunswick
Ephemera varia|08ONMAY-0149|Canada.Ontario
Ephemera varia|09NBMAY-0752|Canada.New Brunswick
Ephemera varia|09NBMAY-0434|Canada.New Brunswick
Ephemera varia|09NBMAY-0427|Canada.New Brunswick
Ephemera varia|09NBMAY-0424|Canada.New Brunswick
Ephemera varia|09NBMAY-0278|Canada.New Brunswick
Ephemera varia|09NBMAY-0277|Canada.New Brunswick
Ephemera varia|09NBMAY-0426|Canada.New Brunswick
Ephemera varia|09NBMAY-0425|Canada.New Brunswick
Ephemera varia|09NBMAY-0421|Canada.New Brunswick
Ephemera varia|09NBMAY-0280|Canada.New Brunswick
Ephemera varia|09NBMAY-0086|Canada.New Brunswick
Ephemera varia|09NBMAY-0085|Canada.New Brunswick
Ephemera varia|09NBMAY-0084|Canada.New Brunswick
Ephemera varia|09NBMAY-0087|Canada.New Brunswick
Ephemera varia|09NBMAY-0275|Canada.New Brunswick
Ephemera varia|10HIMXD-0114|United States.North Carolina
Ephemera varia|10HIMXD-0124|United States.North Carolina
Ephemera varia|10HIMXD-0126|United States.North Carolina
Ephemera varia|10HIMXD-0127|United States.North Carolina
Ephemera varia|10HIMXD-0131|United States.North Carolina
Ephemera varia|HIEPT-1058|United States.North Carolina
Ephemera varia|HIEPT-1121|United States.North Carolina
Ephemera varia|10HIMXD-0130|United States.North Carolina
Ephemera varia|10HIMXD-0129|United States.North Carolina
Ephemera varia|10HIMXD-0125|United States.North Carolina
Ephemera varia|10HIMXD-0115|United States.North Carolina
Ephemera varia|10HIMXD-0128|United States.North Carolina
Ephemera blanda|09LJGSM-067|United States.North Carolina
Ephemera blanda|09LJGSM-065|United States.North Carolina
Ephemera blanda|09LJGSM-063|United States.North Carolina
Ephemera blanda|09LJGSM-066|United States.North Carolina
Ephemera blanda|HIEPT-1059|United States.North Carolina
Ephemera blanda|09LJGSM-064|United States.North Carolina
Ephemera blanda|09LJGSM-062|United States.North Carolina
Thraulodes pacaya|BIOUG00640-E12|Mexico.Veracruz
Thraulodes pacaya|BIOUG00640-E11|Mexico.Veracruz
Thraulodes sp.JMW1|BIOUG00640-D07|Mexico.Veracruz
Thraulodes sp.JMW1|BIOUG00640-D03|Mexico.Veracruz
Thraulodes speciosus|10BGMAY-060|United States.Arizona
Thraulodes speciosus|10BGMAY-059|United States.Arizona
Paraleptophlebia volitans|FAMUBOLD0040|United States.Florida
Paraleptophlebia volitans|09CPMAY-125|Canada.New Brunswick
Paraleptophlebia volitans|09CPMAY-124|Canada.New Brunswick
Paraleptophlebia volitans|09CPMAY-123|Canada.New Brunswick
Paraleptophlebia volitans|09CPMAY-091|Canada.New Brunswick
Paraleptophlebia volitans|09CPMAY-094|Canada.New Brunswick
Paraleptophlebia volitans|09CPMAY-122|Canada.New Brunswick
Paraleptophlebia volitans|09CPMAY-121|Canada.New Brunswick
Paraleptophlebia praepedita|07PROBE-2386|Canada.Manitoba
Paraleptophlebia praepedita|07PROBE-2373|Canada.Manitoba
Paraleptophlebia praepedita|07PROBE-2168|Canada.Manitoba
Paraleptophlebia praepedita|07PROBE-2170|Canada.Manitoba
Paraleptophlebia praepedita|07PROBE-07083|Canada.Manitoba
Paraleptophlebia praepedita|07PROBE-2174|Canada.Manitoba
Paraleptophlebia praepedita|07PROBE-2130|Canada.Manitoba
Paraleptophlebia praepedita|07PROBE-2166|Canada.Manitoba
Paraleptophlebia praepedita|07PROBE-2176|Canada.Manitoba
Paraleptophlebia praepedita|07PROBE-2381|Canada.Manitoba
Paraleptophlebia praepedita|07PROBE-07082|Canada.Manitoba
Ametropus neavei|SWA iBOL UofS 173|Canada.Saskatchewan
Ametropus neavei|SWA iBOL UofS 138|Canada.Saskatchewan
Ametropus neavei|SWA iBOL UofS 93|Canada.Saskatchewan
Ametropus neavei|260 LF SK|Canada.Saskatchewan
Dolania americana|FAMUBOLD0078|United States.Florida
Ephemera varia
Ephemera blanda
Thraulodes pacaya
Thraulodes sp.JMW1
Thraulodes speciosus
Paraleptophlebia volitans
Paraleptophlebia praepedita
Ametropus neavei

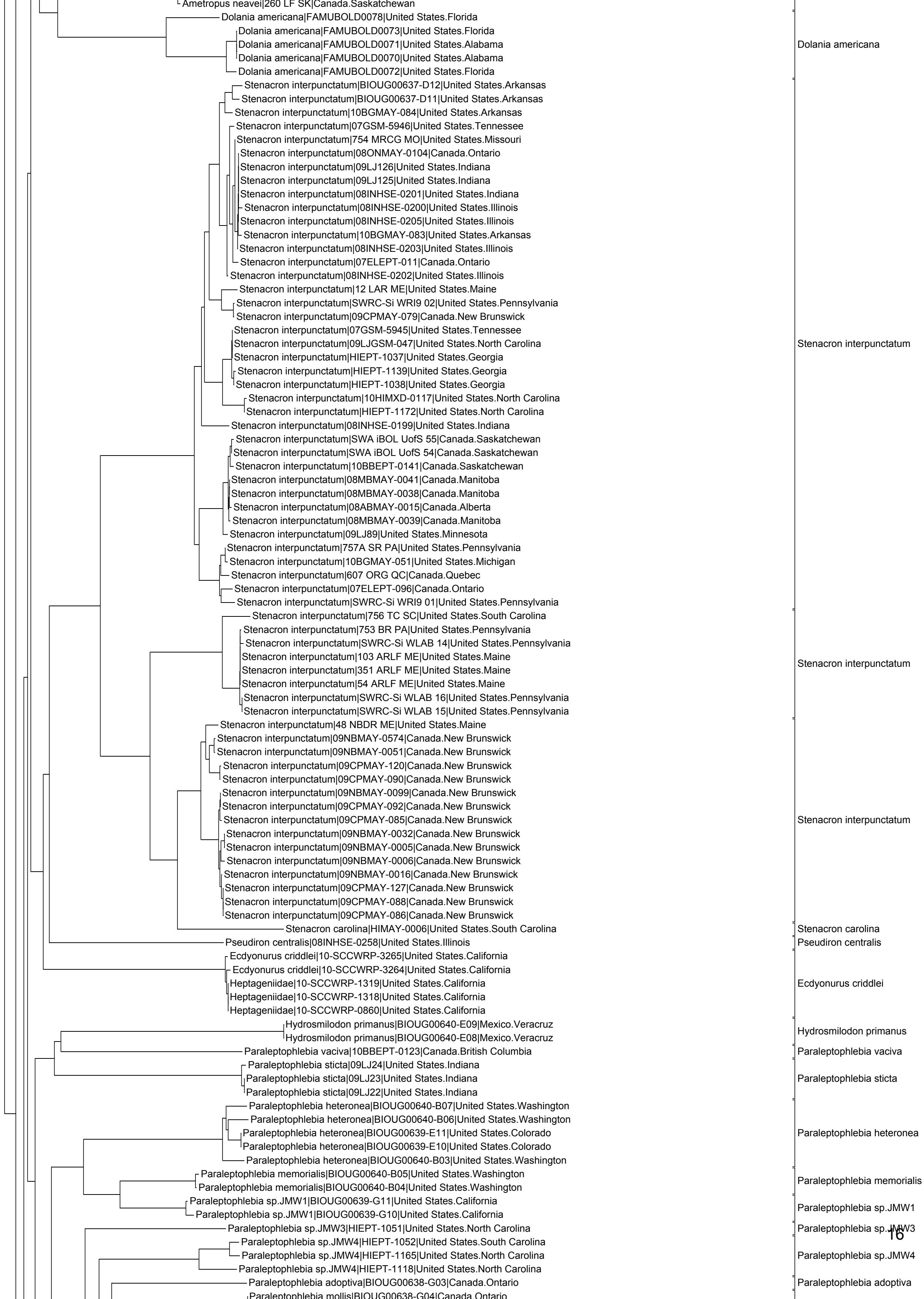
Ametropus neavei|260 LF SK|Canada.Saskatchewan
Dolania americana|FAMUBOLD0078|United States.Florida
Dolania americana|FAMUBOLD0073|United States.Florida
Dolania americana|FAMUBOLD0071|United States.Alabama
Dolania americana|FAMUBOLD0070|United States.Alabama
Dolania americana|FAMUBOLD0072|United States.Florida
Dolania americana
Stenacron interpunctatum|BIOUG00637-D12|United States.Arkansas
Stenacron interpunctatum|BIOUG00637-D11|United States.Arkansas
Stenacron interpunctatum|10BGMAY-084|United States.Arkansas
Stenacron interpunctatum|07GSM-5946|United States.Tennessee
Stenacron interpunctatum|754 MRCG MO|United States.Missouri
Stenacron interpunctatum|08ONMAY-0104|Canada.Ontario
Stenacron interpunctatum|09LJ126|United States.Indiana
Stenacron interpunctatum|09LJ125|United States.Indiana
Stenacron interpunctatum|08INHSE-0201|United States.Indiana
Stenacron interpunctatum|08INHSE-0200|United States.Illinois
Stenacron interpunctatum|08INHSE-0205|United States.Illinois
Stenacron interpunctatum|10BGMAY-083|United States.Arkansas
Stenacron interpunctatum|08INHSE-0203|United States.Illinois
Stenacron interpunctatum|07ELEPT-011|Canada.Ontario
Stenacron interpunctatum|08INHSE-0202|United States.Illinois
Stenacron interpunctatum|12 LAR ME|United States.Maine
Stenacron interpunctatum|SWRC-Si WRI9 02|United States.Pennsylvania
Stenacron interpunctatum|09CPMAY-079|Canada.New Brunswick
Stenacron interpunctatum|07GSM-5945|United States.Tennessee
Stenacron interpunctatum|09LJGSM-047|United States.North Carolina
Stenacron interpunctatum|HIEPT-1037|United States.Georgia
Stenacron interpunctatum|HIEPT-1139|United States.Georgia
Stenacron interpunctatum|HIEPT-1038|United States.Georgia
Stenacron interpunctatum|10HIMXD-0117|United States.North Carolina
Stenacron interpunctatum|HIEPT-1172|United States.North Carolina
Stenacron interpunctatum|08INHSE-0199|United States.Indiana
Stenacron interpunctatum|SWA iBOL UofS 55|Canada.Saskatchewan
Stenacron interpunctatum|SWA iBOL UofS 54|Canada.Saskatchewan
Stenacron interpunctatum|10BBEPT-0141|Canada.Saskatchewan
Stenacron interpunctatum|08MBMAY-0041|Canada.Manitoba
Stenacron interpunctatum|08MBMAY-0038|Canada.Manitoba
Stenacron interpunctatum|08ABMAY-0015|Canada.Alberta
Stenacron interpunctatum|08MBMAY-0039|Canada.Manitoba
Stenacron interpunctatum|09LJ89|United States.Minnesota
Stenacron interpunctatum|757A SR PA|United States.Pennsylvania
Stenacron interpunctatum|10BGMAY-051|United States.Michigan
Stenacron interpunctatum|607 ORG QC|Canada.Quebec
Stenacron interpunctatum|07ELEPT-096|Canada.Ontario
Stenacron interpunctatum|SWRC-Si WRI9 01|United States.Pennsylvania
Stenacron interpunctatum
Stenacron interpunctatum|756 TC SC|United States.South Carolina
Stenacron interpunctatum|753 BR PA|United States.Pennsylvania
Stenacron interpunctatum|SWRC-Si WLAB 14|United States.Pennsylvania
Stenacron interpunctatum|103 ARLF ME|United States.Maine
Stenacron interpunctatum|351 ARLF ME|United States.Maine
Stenacron interpunctatum|54 ARLF ME|United States.Maine
Stenacron interpunctatum|SWRC-Si WLAB 16|United States.Pennsylvania
Stenacron interpunctatum|SWRC-Si WLAB 15|United States.Pennsylvania
Stenacron interpunctatum
Stenacron interpunctatum|48 NBDR ME|United States.Maine
Stenacron interpunctatum|09NBMAY-0574|Canada.New Brunswick
Stenacron interpunctatum|09NBMAY-0051|Canada.New Brunswick
Stenacron interpunctatum|09CPMAY-120|Canada.New Brunswick
Stenacron interpunctatum|09CPMAY-090|Canada.New Brunswick
Stenacron interpunctatum|09NBMAY-0099|Canada.New Brunswick
Stenacron interpunctatum|09CPMAY-092|Canada.New Brunswick
Stenacron interpunctatum|09CPMAY-085|Canada.New Brunswick
Stenacron interpunctatum|09NBMAY-0032|Canada.New Brunswick
Stenacron interpunctatum|09NBMAY-0005|Canada.New Brunswick
Stenacron interpunctatum|09NBMAY-0006|Canada.New Brunswick
Stenacron interpunctatum|09NBMAY-0016|Canada.New Brunswick
Stenacron interpunctatum|09CPMAY-127|Canada.New Brunswick
Stenacron interpunctatum|09CPMAY-088|Canada.New Brunswick
Stenacron interpunctatum|09CPMAY-086|Canada.New Brunswick
Stenacron interpunctatum
Stenacron carolina|HIMAY-0006|United States.South Carolina
Stenacron carolina
Pseudiron centralis|08INHSE-0258|United States.Illinois
Pseudiron centralis
Ecdyonurus criddlei|10-SCCWRP-3265|United States.California
Ecdyonurus criddlei|10-SCCWRP-3264|United States.California
Heptageniidae|10-SCCWRP-1319|United States.California
Heptageniidae|10-SCCWRP-1318|United States.California
Heptageniidae|10-SCCWRP-0860|United States.California
Ecdyonurus criddlei
Hydrosmilodon primanus|BIOUG00640-E09|Mexico.Veracruz
Hydrosmilodon primanus|BIOUG00640-E08|Mexico.Veracruz
Hydrosmilodon primanus
Paraleptophlebia vaciva|10BBEPT-0123|Canada.British Columbia
Paraleptophlebia vaciva
Paraleptophlebia sticta|09LJ24|United States.Indiana
Paraleptophlebia sticta|09LJ23|United States.Indiana
Paraleptophlebia sticta|09LJ22|United States.Indiana
Paraleptophlebia sticta
Paraleptophlebia heteronea|BIOUG00640-B07|United States.Washington
Paraleptophlebia heteronea|BIOUG00640-B06|United States.Washington
Paraleptophlebia heteronea|BIOUG00639-E11|United States.Colorado
Paraleptophlebia heteronea|BIOUG00639-E10|United States.Colorado
Paraleptophlebia heteronea|BIOUG00640-B03|United States.Washington
Paraleptophlebia heteronea
Paraleptophlebia memorialis|BIOUG00640-B05|United States.Washington
Paraleptophlebia memorialis|BIOUG00640-B04|United States.Washington
Paraleptophlebia memorialis
Paraleptophlebia sp.JMW1|BIOUG00639-G11|United States.California
Paraleptophlebia sp.JMW1|BIOUG00639-G10|United States.California
Paraleptophlebia sp.JMW1
Paraleptophlebia sp.JMW3|HIEPT-1051|United States.North Carolina
Paraleptophlebia sp.JMW3
Paraleptophlebia sp.JMW4|HIEPT-1052|United States.South Carolina
Paraleptophlebia sp.JMW4|HIEPT-1165|United States.North Carolina
Paraleptophlebia sp.JMW4|HIEPT-1118|United States.North Carolina
Paraleptophlebia sp.JMW4
Paraleptophlebia adoptiva|BIOUG00638-G03|Canada.Ontario
Paraleptophlebia adoptiva
Paraleptophlebia mollis|BIOUG00638-G04|Canada.Ontario

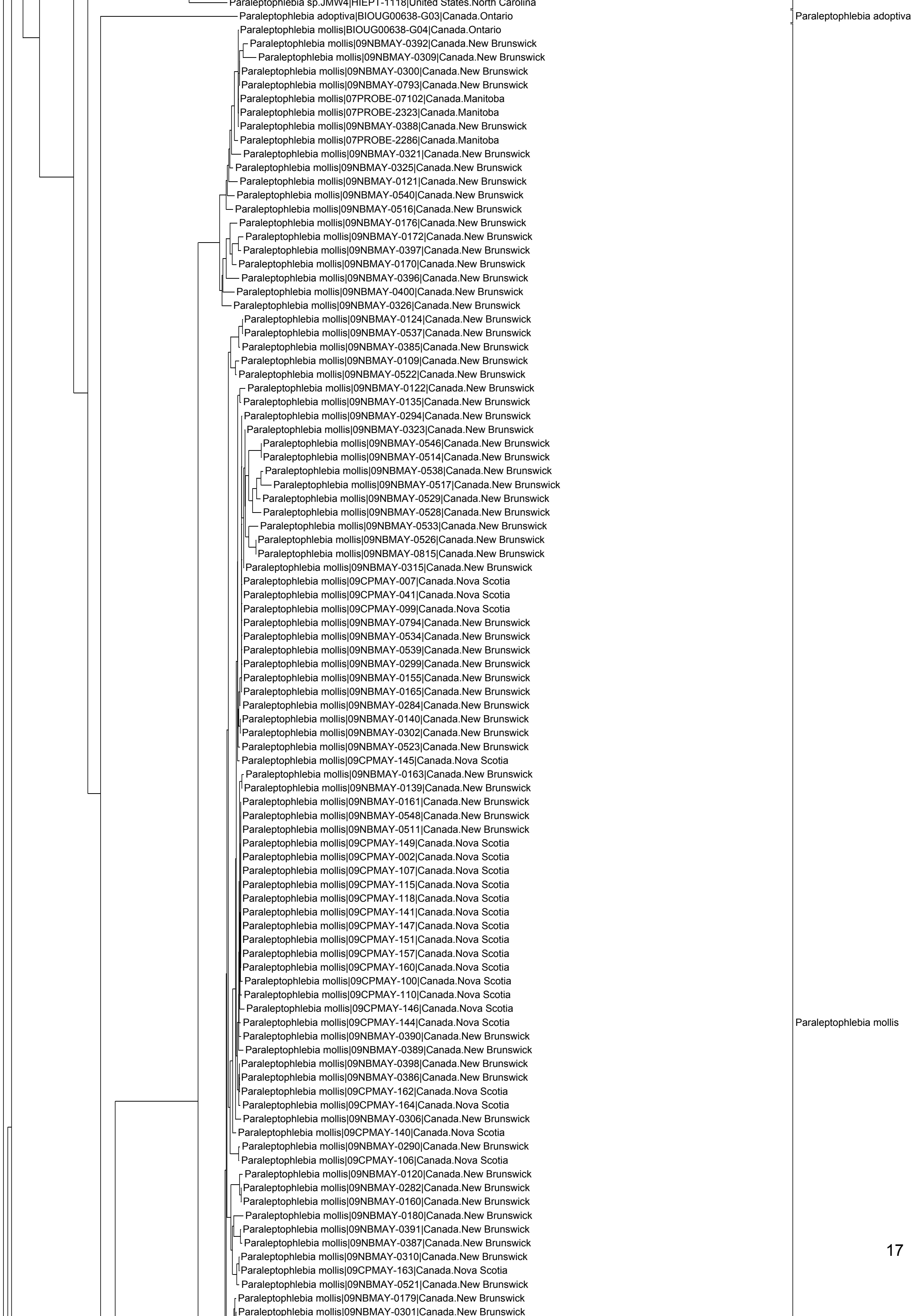

Paraleptophlebia sp.JMW4|HIEPT-1118|United States.North Carolina
Paraleptophlebia adoptiva|BIOUG00638-G03|Canada.Ontario
Paraleptophlebia mollis|BIOUG00638-G04|Canada.Ontario
Paraleptophlebia mollis|09NBMAY-0392|Canada.New Brunswick
Paraleptophlebia mollis|09NBMAY-0309|Canada.New Brunswick
Paraleptophlebia mollis|09NBMAY-0300|Canada.New Brunswick
Paraleptophlebia mollis|09NBMAY-0793|Canada.New Brunswick
Paraleptophlebia mollis|07PROBE-07102|Canada.Manitoba
Paraleptophlebia mollis|07PROBE-2323|Canada.Manitoba
Paraleptophlebia mollis|09NBMAY-0388|Canada.New Brunswick
Paraleptophlebia mollis|07PROBE-2286|Canada.Manitoba
Paraleptophlebia mollis|09NBMAY-0321|Canada.New Brunswick
Paraleptophlebia mollis|09NBMAY-0325|Canada.New Brunswick
Paraleptophlebia mollis|09NBMAY-0121|Canada.New Brunswick
Paraleptophlebia mollis|09NBMAY-0540|Canada.New Brunswick
Paraleptophlebia mollis|09NBMAY-0516|Canada.New Brunswick
Paraleptophlebia mollis|09NBMAY-0176|Canada.New Brunswick
Paraleptophlebia mollis|09NBMAY-0172|Canada.New Brunswick
Paraleptophlebia mollis|09NBMAY-0397|Canada.New Brunswick
Paraleptophlebia mollis|09NBMAY-0170|Canada.New Brunswick
Paraleptophlebia mollis|09NBMAY-0396|Canada.New Brunswick
Paraleptophlebia mollis|09NBMAY-0400|Canada.New Brunswick
Paraleptophlebia mollis|09NBMAY-0326|Canada.New Brunswick
Paraleptophlebia mollis|09NBMAY-0124|Canada.New Brunswick
Paraleptophlebia mollis|09NBMAY-0537|Canada.New Brunswick
Paraleptophlebia mollis|09NBMAY-0385|Canada.New Brunswick
Paraleptophlebia mollis|09NBMAY-0109|Canada.New Brunswick
Paraleptophlebia mollis|09NBMAY-0522|Canada.New Brunswick
Paraleptophlebia mollis|09NBMAY-0122|Canada.New Brunswick
Paraleptophlebia mollis|09NBMAY-0135|Canada.New Brunswick
Paraleptophlebia mollis|09NBMAY-0294|Canada.New Brunswick
Paraleptophlebia mollis|09NBMAY-0323|Canada.New Brunswick
Paraleptophlebia mollis|09NBMAY-0546|Canada.New Brunswick
Paraleptophlebia mollis|09NBMAY-0514|Canada.New Brunswick
Paraleptophlebia mollis|09NBMAY-0538|Canada.New Brunswick
Paraleptophlebia mollis|09NBMAY-0517|Canada.New Brunswick
Paraleptophlebia mollis|09NBMAY-0529|Canada.New Brunswick
Paraleptophlebia mollis|09NBMAY-0528|Canada.New Brunswick
Paraleptophlebia mollis|09NBMAY-0533|Canada.New Brunswick
Paraleptophlebia mollis|09NBMAY-0526|Canada.New Brunswick
Paraleptophlebia mollis|09NBMAY-0815|Canada.New Brunswick
Paraleptophlebia mollis|09NBMAY-0315|Canada.New Brunswick
Paraleptophlebia mollis|09CPMAY-007|Canada.Nova Scotia
Paraleptophlebia mollis|09CPMAY-041|Canada.Nova Scotia
Paraleptophlebia mollis|09CPMAY-099|Canada.Nova Scotia
Paraleptophlebia mollis|09NBMAY-0794|Canada.New Brunswick
Paraleptophlebia mollis|09NBMAY-0534|Canada.New Brunswick
Paraleptophlebia mollis|09NBMAY-0539|Canada.New Brunswick
Paraleptophlebia mollis|09NBMAY-0299|Canada.New Brunswick
Paraleptophlebia mollis|09NBMAY-0155|Canada.New Brunswick
Paraleptophlebia mollis|09NBMAY-0165|Canada.New Brunswick
Paraleptophlebia mollis|09NBMAY-0284|Canada.New Brunswick
Paraleptophlebia mollis|09NBMAY-0140|Canada.New Brunswick
Paraleptophlebia mollis|09NBMAY-0302|Canada.New Brunswick
Paraleptophlebia mollis|09NBMAY-0523|Canada.New Brunswick
Paraleptophlebia mollis|09CPMAY-145|Canada.Nova Scotia
Paraleptophlebia mollis|09NBMAY-0163|Canada.New Brunswick
Paraleptophlebia mollis|09NBMAY-0139|Canada.New Brunswick
Paraleptophlebia mollis|09NBMAY-0161|Canada.New Brunswick
Paraleptophlebia mollis|09NBMAY-0548|Canada.New Brunswick
Paraleptophlebia mollis|09NBMAY-0511|Canada.New Brunswick
Paraleptophlebia mollis|09CPMAY-149|Canada.Nova Scotia
Paraleptophlebia mollis|09CPMAY-002|Canada.Nova Scotia
Paraleptophlebia mollis|09CPMAY-107|Canada.Nova Scotia
Paraleptophlebia mollis|09CPMAY-115|Canada.Nova Scotia
Paraleptophlebia mollis|09CPMAY-118|Canada.Nova Scotia
Paraleptophlebia mollis|09CPMAY-141|Canada.Nova Scotia
Paraleptophlebia mollis|09CPMAY-147|Canada.Nova Scotia
Paraleptophlebia mollis|09CPMAY-151|Canada.Nova Scotia
Paraleptophlebia mollis|09CPMAY-157|Canada.Nova Scotia
Paraleptophlebia mollis|09CPMAY-160|Canada.Nova Scotia
Paraleptophlebia mollis|09CPMAY-100|Canada.Nova Scotia
Paraleptophlebia mollis|09CPMAY-110|Canada.Nova Scotia
Paraleptophlebia mollis|09CPMAY-146|Canada.Nova Scotia
Paraleptophlebia mollis|09CPMAY-144|Canada.Nova Scotia
Paraleptophlebia mollis|09NBMAY-0390|Canada.New Brunswick
Paraleptophlebia mollis|09NBMAY-0389|Canada.New Brunswick
Paraleptophlebia mollis|09NBMAY-0398|Canada.New Brunswick
Paraleptophlebia mollis|09NBMAY-0386|Canada.New Brunswick
Paraleptophlebia mollis|09CPMAY-162|Canada.Nova Scotia
Paraleptophlebia mollis|09CPMAY-164|Canada.Nova Scotia
Paraleptophlebia mollis|09NBMAY-0306|Canada.New Brunswick
Paraleptophlebia mollis|09CPMAY-140|Canada.Nova Scotia
Paraleptophlebia mollis|09NBMAY-0290|Canada.New Brunswick
Paraleptophlebia mollis|09CPMAY-106|Canada.Nova Scotia
Paraleptophlebia mollis|09NBMAY-0120|Canada.New Brunswick
Paraleptophlebia mollis|09NBMAY-0282|Canada.New Brunswick
Paraleptophlebia mollis|09NBMAY-0160|Canada.New Brunswick
Paraleptophlebia mollis|09NBMAY-0180|Canada.New Brunswick
Paraleptophlebia mollis|09NBMAY-0391|Canada.New Brunswick
Paraleptophlebia mollis|09NBMAY-0387|Canada.New Brunswick
Paraleptophlebia mollis|09NBMAY-0310|Canada.New Brunswick
Paraleptophlebia mollis|09CPMAY-163|Canada.Nova Scotia
Paraleptophlebia mollis|09NBMAY-0521|Canada.New Brunswick
Paraleptophlebia mollis|09NBMAY-0179|Canada.New Brunswick
Paraleptophlebia mollis|09NBMAY-0301|Canada.New Brunswick
Paraleptophlebia adoptiva
Paraleptophlebia mollis

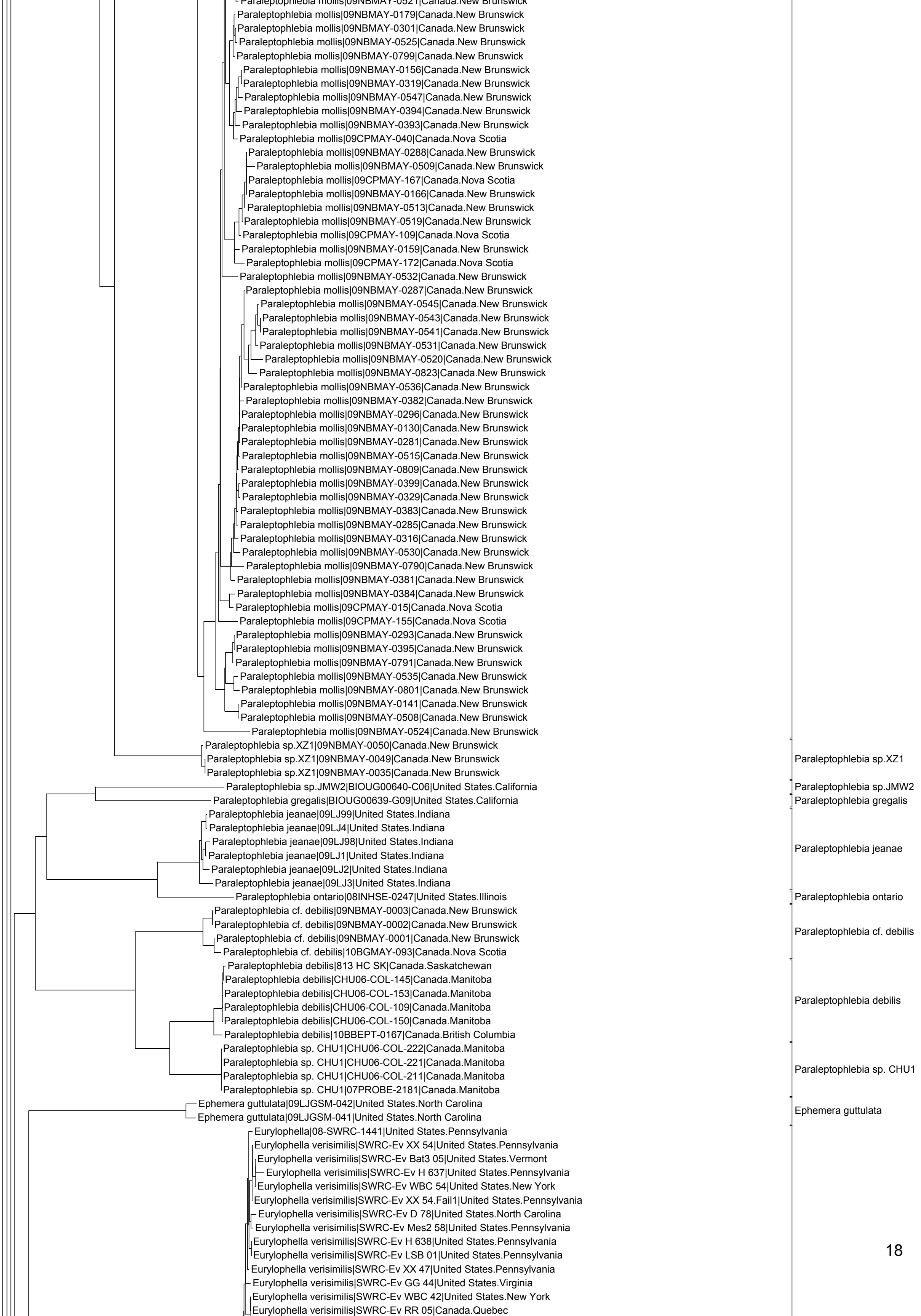

Paraleptophlebia mollis|09NBMAY-0521|Canada.New Brunswick
Paraleptophlebia mollis|09NBMAY-0179|Canada.New Brunswick
Paraleptophlebia mollis|09NBMAY-0301|Canada.New Brunswick
Paraleptophlebia mollis|09NBMAY-0525|Canada.New Brunswick
Paraleptophlebia mollis|09NBMAY-0799|Canada.New Brunswick
Paraleptophlebia mollis|09NBMAY-0156|Canada.New Brunswick
Paraleptophlebia mollis|09NBMAY-0319|Canada.New Brunswick
Paraleptophlebia mollis|09NBMAY-0547|Canada.New Brunswick
Paraleptophlebia mollis|09NBMAY-0394|Canada.New Brunswick
Paraleptophlebia mollis|09NBMAY-0393|Canada.New Brunswick
Paraleptophlebia mollis|09CPMAY-040|Canada.Nova Scotia
Paraleptophlebia mollis|09NBMAY-0288|Canada.New Brunswick
Paraleptophlebia mollis|09NBMAY-0509|Canada.New Brunswick
Paraleptophlebia mollis|09CPMAY-167|Canada.Nova Scotia
Paraleptophlebia mollis|09NBMAY-0166|Canada.New Brunswick
Paraleptophlebia mollis|09NBMAY-0513|Canada.New Brunswick
Paraleptophlebia mollis|09NBMAY-0519|Canada.New Brunswick
Paraleptophlebia mollis|09CPMAY-109|Canada.Nova Scotia
Paraleptophlebia mollis|09NBMAY-0159|Canada.New Brunswick
Paraleptophlebia mollis|09CPMAY-172|Canada.Nova Scotia
Paraleptophlebia mollis|09NBMAY-0532|Canada.New Brunswick
Paraleptophlebia mollis|09NBMAY-0287|Canada.New Brunswick
Paraleptophlebia mollis|09NBMAY-0545|Canada.New Brunswick
Paraleptophlebia mollis|09NBMAY-0543|Canada.New Brunswick
Paraleptophlebia mollis|09NBMAY-0541|Canada.New Brunswick
Paraleptophlebia mollis|09NBMAY-0531|Canada.New Brunswick
Paraleptophlebia mollis|09NBMAY-0520|Canada.New Brunswick
Paraleptophlebia mollis|09NBMAY-0823|Canada.New Brunswick
Paraleptophlebia mollis|09NBMAY-0536|Canada.New Brunswick
Paraleptophlebia mollis|09NBMAY-0382|Canada.New Brunswick
Paraleptophlebia mollis|09NBMAY-0296|Canada.New Brunswick
Paraleptophlebia mollis|09NBMAY-0130|Canada.New Brunswick
Paraleptophlebia mollis|09NBMAY-0281|Canada.New Brunswick
Paraleptophlebia mollis|09NBMAY-0515|Canada.New Brunswick
Paraleptophlebia mollis|09NBMAY-0809|Canada.New Brunswick
Paraleptophlebia mollis|09NBMAY-0399|Canada.New Brunswick
Paraleptophlebia mollis|09NBMAY-0329|Canada.New Brunswick
Paraleptophlebia mollis|09NBMAY-0383|Canada.New Brunswick
Paraleptophlebia mollis|09NBMAY-0285|Canada.New Brunswick
Paraleptophlebia mollis|09NBMAY-0316|Canada.New Brunswick
Paraleptophlebia mollis|09NBMAY-0530|Canada.New Brunswick
Paraleptophlebia mollis|09NBMAY-0790|Canada.New Brunswick
Paraleptophlebia mollis|09NBMAY-0381|Canada.New Brunswick
Paraleptophlebia mollis|09NBMAY-0384|Canada.New Brunswick
Paraleptophlebia mollis|09CPMAY-015|Canada.Nova Scotia
Paraleptophlebia mollis|09CPMAY-155|Canada.Nova Scotia
Paraleptophlebia mollis|09NBMAY-0293|Canada.New Brunswick
Paraleptophlebia mollis|09NBMAY-0395|Canada.New Brunswick
Paraleptophlebia mollis|09NBMAY-0791|Canada.New Brunswick
Paraleptophlebia mollis|09NBMAY-0535|Canada.New Brunswick
Paraleptophlebia mollis|09NBMAY-0801|Canada.New Brunswick
Paraleptophlebia mollis|09NBMAY-0141|Canada.New Brunswick
Paraleptophlebia mollis|09NBMAY-0508|Canada.New Brunswick
Paraleptophlebia mollis|09NBMAY-0524|Canada.New Brunswick
Paraleptophlebia sp.XZ1|09NBMAY-0050|Canada.New Brunswick
Paraleptophlebia sp.XZ1|09NBMAY-0049|Canada.New Brunswick
Paraleptophlebia sp.XZ1|09NBMAY-0035|Canada.New Brunswick
Paraleptophlebia sp.XZ1
Paraleptophlebia sp.JMW2|BIOUG00640-C06|United States.California
Paraleptophlebia sp.JMW2
Paraleptophlebia gregalis|BIOUG00639-G09|United States.California
Paraleptophlebia gregalis
Paraleptophlebia jeanae|09LJ99|United States.Indiana
Paraleptophlebia jeanae|09LJ4|United States.Indiana
Paraleptophlebia jeanae|09LJ98|United States.Indiana
Paraleptophlebia jeanae|09LJ1|United States.Indiana
Paraleptophlebia jeanae|09LJ2|United States.Indiana
Paraleptophlebia jeanae|09LJ3|United States.Indiana
Paraleptophlebia jeanae
Paraleptophlebia ontario|08INHSE-0247|United States.Illinois
Paraleptophlebia ontario
Paraleptophlebia cf. debilis|09NBMAY-0003|Canada.New Brunswick
Paraleptophlebia cf. debilis|09NBMAY-0002|Canada.New Brunswick
Paraleptophlebia cf. debilis|09NBMAY-0001|Canada.New Brunswick
Paraleptophlebia cf. debilis|10BGMAY-093|Canada.Nova Scotia
Paraleptophlebia cf. debilis
Paraleptophlebia debilis|813 HC SK|Canada.Saskatchewan
Paraleptophlebia debilis|CHU06-COL-145|Canada.Manitoba
Paraleptophlebia debilis|CHU06-COL-153|Canada.Manitoba
Paraleptophlebia debilis|CHU06-COL-109|Canada.Manitoba
Paraleptophlebia debilis|CHU06-COL-150|Canada.Manitoba
Paraleptophlebia debilis|10BBEPT-0167|Canada.British Columbia
Paraleptophlebia debilis
Paraleptophlebia sp. CHU1|CHU06-COL-222|Canada.Manitoba
Paraleptophlebia sp. CHU1|CHU06-COL-221|Canada.Manitoba
Paraleptophlebia sp. CHU1|CHU06-COL-211|Canada.Manitoba
Paraleptophlebia sp. CHU1|07PROBE-2181|Canada.Manitoba
Paraleptophlebia sp. CHU1
Ephemera guttulata|09LJGSM-042|United States.North Carolina
Ephemera guttulata|09LJGSM-041|United States.North Carolina
Ephemera guttulata
Eurylophella|08-SWRC-1441|United States.Pennsylvania
Eurylophella verisimilis|SWRC-Ev XX 54|United States.Pennsylvania
Eurylophella verisimilis|SWRC-Ev Bat3 05|United States.Vermont
Eurylophella verisimilis|SWRC-Ev H 637|United States.Pennsylvania
Eurylophella verisimilis|SWRC-Ev WBC 54|United States.New York
Eurylophella verisimilis|SWRC-Ev XX 54.Fail1|United States.Pennsylvania
Eurylophella verisimilis|SWRC-Ev D 78|United States.North Carolina
Eurylophella verisimilis|SWRC-Ev Mes2 58|United States.Pennsylvania
Eurylophella verisimilis|SWRC-Ev H 638|United States.Pennsylvania
Eurylophella verisimilis|SWRC-Ev LSB 01|United States.Pennsylvania
Eurylophella verisimilis|SWRC-Ev XX 47|United States.Pennsylvania
Eurylophella verisimilis|SWRC-Ev GG 44|United States.Virginia
Eurylophella verisimilis|SWRC-Ev WBC 42|United States.New York
Eurylophella verisimilis|SWRC-Ev RR 05|Canada.Quebec

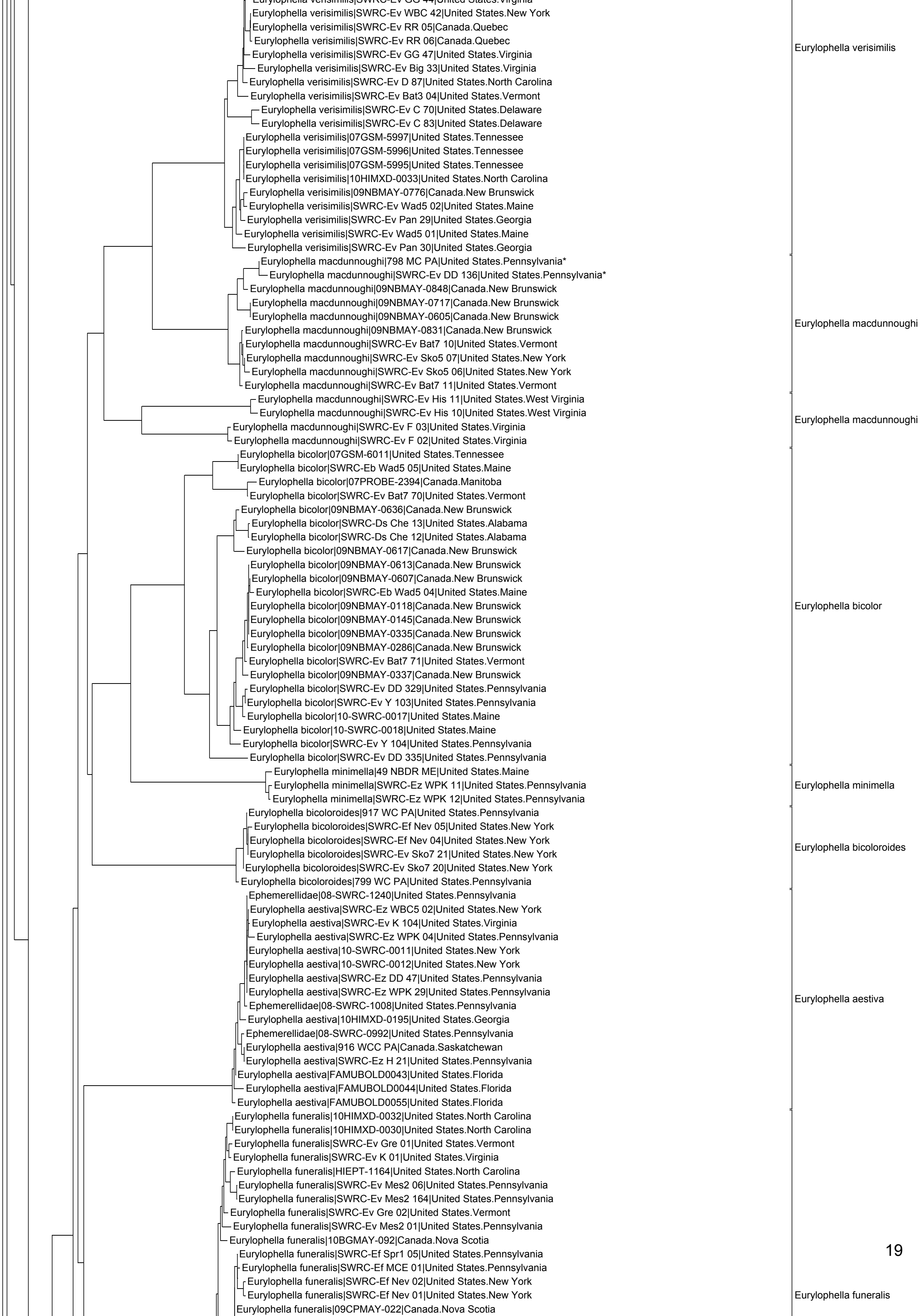

Eurylophella verisimilis|SWRC-Ev GG 44|United States.Virginia
Eurylophella verisimilis|SWRC-Ev WBC 42|United States.New York
Eurylophella verisimilis|SWRC-Ev RR 05|Canada.Quebec
Eurylophella verisimilis|SWRC-Ev RR 06|Canada.Quebec
Eurylophella verisimilis|SWRC-Ev GG 47|United States.Virginia
Eurylophella verisimilis|SWRC-Ev Big 33|United States.Virginia
Eurylophella verisimilis|SWRC-Ev D 87|United States.North Carolina
Eurylophella verisimilis|SWRC-Ev Bat3 04|United States.Vermont
Eurylophella verisimilis|SWRC-Ev C 70|United States.Delaware
Eurylophella verisimilis|SWRC-Ev C 83|United States.Delaware
Eurylophella verisimilis|07GSM-5997|United States.Tennessee
Eurylophella verisimilis|07GSM-5996|United States.Tennessee
Eurylophella verisimilis|07GSM-5995|United States.Tennessee
Eurylophella verisimilis|10HIMXD-0033|United States.North Carolina
Eurylophella verisimilis|09NBMAY-0776|Canada.New Brunswick
Eurylophella verisimilis|SWRC-Ev Wad5 02|United States.Maine
Eurylophella verisimilis|SWRC-Ev Pan 29|United States.Georgia
Eurylophella verisimilis|SWRC-Ev Wad5 01|United States.Maine
Eurylophella verisimilis|SWRC-Ev Pan 30|United States.Georgia
Eurylophella verisimilis
Eurylophella macdunnoughi|798 MC PA|United States.Pennsylvania*
Eurylophella macdunnoughi|SWRC-Ev DD 136|United States.Pennsylvania*
Eurylophella macdunnoughi|09NBMAY-0848|Canada.New Brunswick
Eurylophella macdunnoughi|09NBMAY-0717|Canada.New Brunswick
Eurylophella macdunnoughi|09NBMAY-0605|Canada.New Brunswick
Eurylophella macdunnoughi|09NBMAY-0831|Canada.New Brunswick
Eurylophella macdunnoughi|SWRC-Ev Bat7 10|United States.Vermont
Eurylophella macdunnoughi|SWRC-Ev Sko5 07|United States.New York
Eurylophella macdunnoughi|SWRC-Ev Sko5 06|United States.New York
Eurylophella macdunnoughi|SWRC-Ev Bat7 11|United States.Vermont
Eurylophella macdunnoughi
Eurylophella macdunnoughi|SWRC-Ev His 11|United States.West Virginia
Eurylophella macdunnoughi|SWRC-Ev His 10|United States.West Virginia
Eurylophella macdunnoughi|SWRC-Ev F 03|United States.Virginia
Eurylophella macdunnoughi|SWRC-Ev F 02|United States.Virginia
Eurylophella macdunnoughi
Eurylophella bicolor|07GSM-6011|United States.Tennessee
Eurylophella bicolor|SWRC-Eb Wad5 05|United States.Maine
Eurylophella bicolor|07PROBE-2394|Canada.Manitoba
Eurylophella bicolor|SWRC-Ev Bat7 70|United States.Vermont
Eurylophella bicolor|09NBMAY-0636|Canada.New Brunswick
Eurylophella bicolor|SWRC-Ds Che 13|United States.Alabama
Eurylophella bicolor|SWRC-Ds Che 12|United States.Alabama
Eurylophella bicolor|09NBMAY-0617|Canada.New Brunswick
Eurylophella bicolor|09NBMAY-0613|Canada.New Brunswick
Eurylophella bicolor|09NBMAY-0607|Canada.New Brunswick
Eurylophella bicolor|SWRC-Eb Wad5 04|United States.Maine
Eurylophella bicolor|09NBMAY-0118|Canada.New Brunswick
Eurylophella bicolor|09NBMAY-0145|Canada.New Brunswick
Eurylophella bicolor|09NBMAY-0335|Canada.New Brunswick
Eurylophella bicolor|09NBMAY-0286|Canada.New Brunswick
Eurylophella bicolor|SWRC-Ev Bat7 71|United States.Vermont
Eurylophella bicolor|09NBMAY-0337|Canada.New Brunswick
Eurylophella bicolor|SWRC-Ev DD 329|United States.Pennsylvania
Eurylophella bicolor|SWRC-Ev Y 103|United States.Pennsylvania
Eurylophella bicolor|10-SWRC-0017|United States.Maine
Eurylophella bicolor|10-SWRC-0018|United States.Maine
Eurylophella bicolor|SWRC-Ev Y 104|United States.Pennsylvania
Eurylophella bicolor|SWRC-Ev DD 335|United States.Pennsylvania
Eurylophella bicolor
Eurylophella minimella|49 NBDR ME|United States.Maine
Eurylophella minimella|SWRC-Ez WPK 11|United States.Pennsylvania
Eurylophella minimella|SWRC-Ez WPK 12|United States.Pennsylvania
Eurylophella minimella
Eurylophella bicoloroides|917 WC PA|United States.Pennsylvania
Eurylophella bicoloroides|SWRC-Ef Nev 05|United States.New York
Eurylophella bicoloroides|SWRC-Ef Nev 04|United States.New York
Eurylophella bicoloroides|SWRC-Ev Sko7 21|United States.New York
Eurylophella bicoloroides|SWRC-Ev Sko7 20|United States.New York
Eurylophella bicoloroides|799 WC PA|United States.Pennsylvania
Eurylophella bicoloroides
Ephemerellidae|08-SWRC-1240|United States.Pennsylvania
Eurylophella aestiva|SWRC-Ez WBC5 02|United States.New York
Eurylophella aestiva|SWRC-Ev K 104|United States.Virginia
Eurylophella aestiva|SWRC-Ez WPK 04|United States.Pennsylvania
Eurylophella aestiva|10-SWRC-0011|United States.New York
Eurylophella aestiva|10-SWRC-0012|United States.New York
Eurylophella aestiva|SWRC-Ez DD 47|United States.Pennsylvania
Eurylophella aestiva|SWRC-Ez WPK 29|United States.Pennsylvania
Ephemerellidae|08-SWRC-1008|United States.Pennsylvania
Eurylophella aestiva|10HIMXD-0195|United States.Georgia
Ephemerellidae|08-SWRC-0992|United States.Pennsylvania
Eurylophella aestiva|916 WCC PA|Canada.Saskatchewan
Eurylophella aestiva|SWRC-Ez H 21|United States.Pennsylvania
Eurylophella aestiva|FAMUBOLD0043|United States.Florida
Eurylophella aestiva|FAMUBOLD0044|United States.Florida
Eurylophella aestiva|FAMUBOLD0055|United States.Florida
Eurylophella aestiva
Eurylophella funeralis|10HIMXD-0032|United States.North Carolina
Eurylophella funeralis|10HIMXD-0030|United States.North Carolina
Eurylophella funeralis|SWRC-Ev Gre 01|United States.Vermont
Eurylophella funeralis|SWRC-Ev K 01|United States.Virginia
Eurylophella funeralis|HIEPT-1164|United States.North Carolina
Eurylophella funeralis|SWRC-Ev Mes2 06|United States.Pennsylvania
Eurylophella funeralis|SWRC-Ev Mes2 164|United States.Pennsylvania
Eurylophella funeralis|SWRC-Ev Gre 02|United States.Vermont
Eurylophella funeralis|SWRC-Ev Mes2 01|United States.Pennsylvania
Eurylophella funeralis|10BGMAY-092|Canada.Nova Scotia
Eurylophella funeralis|SWRC-Ef Spr1 05|United States.Pennsylvania
Eurylophella funeralis|SWRC-Ef MCE 01|United States.Pennsylvania
Eurylophella funeralis|SWRC-Ef Nev 02|United States.New York
Eurylophella funeralis|SWRC-Ef Nev 01|United States.New York
Eurylophella funeralis|09CPMAY-022|Canada.Nova Scotia
Eurylophella funeralis

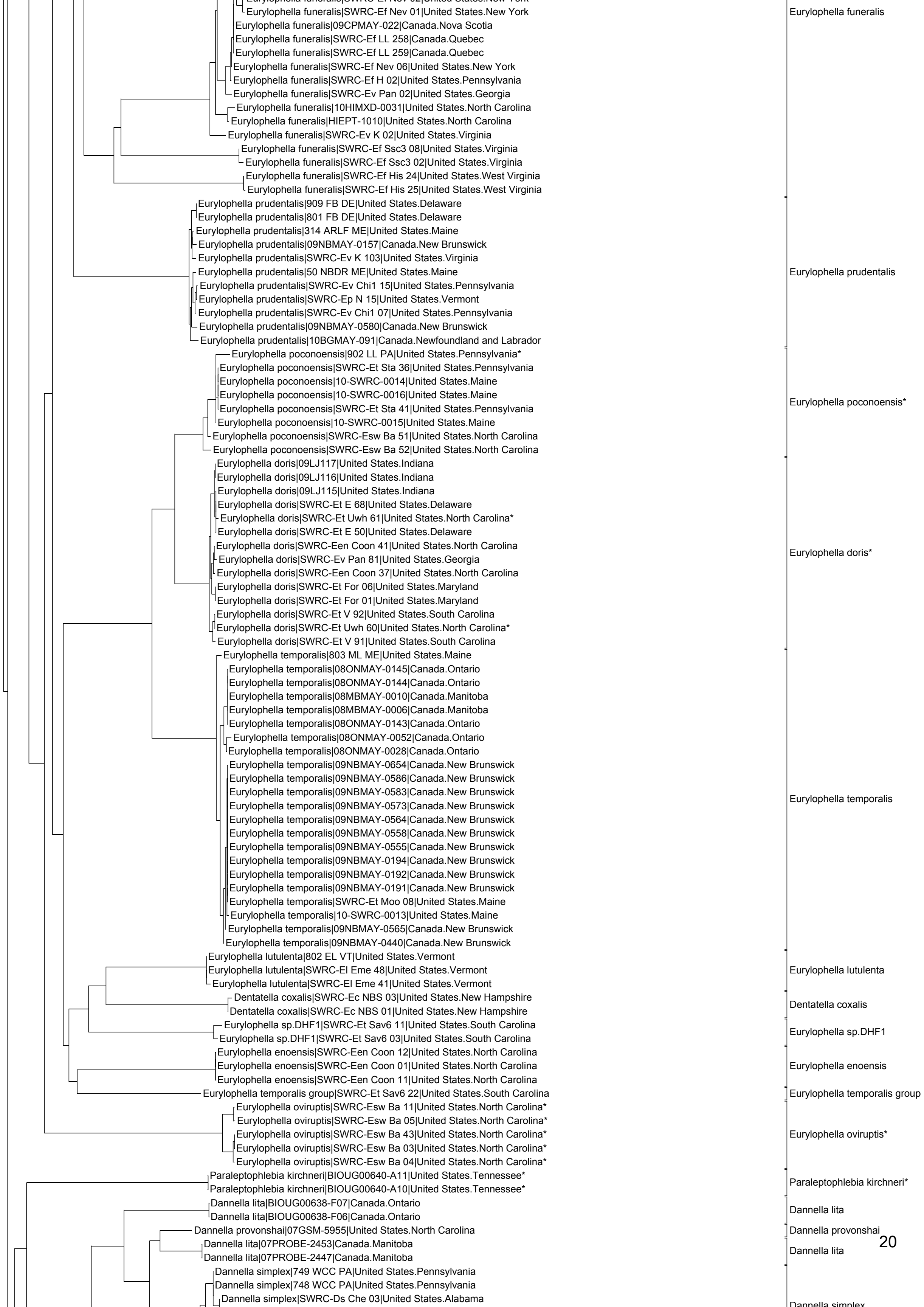
Eurylophella funeralis|SWRC-Ef Nev 01|United States.New York
Eurylophella funeralis|09CPMAY-022|Canada.Nova Scotia
Eurylophella funeralis|SWRC-Ef LL 258|Canada.Quebec
Eurylophella funeralis|SWRC-Ef LL 259|Canada.Quebec
Eurylophella funeralis|SWRC-Ef Nev 06|United States.New York
Eurylophella funeralis|SWRC-Ef H 02|United States.Pennsylvania
Eurylophella funeralis|SWRC-Ev Pan 02|United States.Georgia
Eurylophella funeralis|10HIMXD-0031|United States.North Carolina
Eurylophella funeralis|HIEPT-1010|United States.North Carolina
Eurylophella funeralis|SWRC-Ev K 02|United States.Virginia
Eurylophella funeralis|SWRC-Ef Ssc3 08|United States.Virginia
Eurylophella funeralis|SWRC-Ef Ssc3 02|United States.Virginia
Eurylophella funeralis|SWRC-Ef His 24|United States.West Virginia
Eurylophella funeralis|SWRC-Ef His 25|United States.West Virginia
Eurylophella funeralis
Eurylophella prudentalis|909 FB DE|United States.Delaware
Eurylophella prudentalis|801 FB DE|United States.Delaware
Eurylophella prudentalis|314 ARLF ME|United States.Maine
Eurylophella prudentalis|09NBMAY-0157|Canada.New Brunswick
Eurylophella prudentalis|SWRC-Ev K 103|United States.Virginia
Eurylophella prudentalis|50 NBDR ME|United States.Maine
Eurylophella prudentalis|SWRC-Ev Chi1 15|United States.Pennsylvania
Eurylophella prudentalis|SWRC-Ep N 15|United States.Vermont
Eurylophella prudentalis|SWRC-Ev Chi1 07|United States.Pennsylvania
Eurylophella prudentalis|09NBMAY-0580|Canada.New Brunswick
Eurylophella prudentalis|10BGMAY-091|Canada.Newfoundland and Labrador
Eurylophella prudentalis
Eurylophella poconoensis|902 LL PA|United States.Pennsylvania*
Eurylophella poconoensis|SWRC-Et Sta 36|United States.Pennsylvania
Eurylophella poconoensis|10-SWRC-0014|United States.Maine
Eurylophella poconoensis|10-SWRC-0016|United States.Maine
Eurylophella poconoensis|SWRC-Et Sta 41|United States.Pennsylvania
Eurylophella poconoensis|10-SWRC-0015|United States.Maine
Eurylophella poconoensis|SWRC-Esw Ba 51|United States.North Carolina
Eurylophella poconoensis|SWRC-Esw Ba 52|United States.North Carolina
Eurylophella poconoensis*
Eurylophella doris|09LJ117|United States.Indiana
Eurylophella doris|09LJ116|United States.Indiana
Eurylophella doris|09LJ115|United States.Indiana
Eurylophella doris|SWRC-Et E 68|United States.Delaware
Eurylophella doris|SWRC-Et Uwh 61|United States.North Carolina*
Eurylophella doris|SWRC-Et E 50|United States.Delaware
Eurylophella doris|SWRC-Een Coon 41|United States.North Carolina
Eurylophella doris|SWRC-Ev Pan 81|United States.Georgia
Eurylophella doris|SWRC-Een Coon 37|United States.North Carolina
Eurylophella doris|SWRC-Et For 06|United States.Maryland
Eurylophella doris|SWRC-Et For 01|United States.Maryland
Eurylophella doris|SWRC-Et V 92|United States.South Carolina
Eurylophella doris|SWRC-Et Uwh 60|United States.North Carolina*
Eurylophella doris|SWRC-Et V 91|United States.South Carolina
Eurylophella doris*
Eurylophella temporalis|803 ML ME|United States.Maine
Eurylophella temporalis|08ONMAY-0145|Canada.Ontario
Eurylophella temporalis|08ONMAY-0144|Canada.Ontario
Eurylophella temporalis|08MBMAY-0010|Canada.Manitoba
Eurylophella temporalis|08MBMAY-0006|Canada.Manitoba
Eurylophella temporalis|08ONMAY-0143|Canada.Ontario
Eurylophella temporalis|08ONMAY-0052|Canada.Ontario
Eurylophella temporalis|08ONMAY-0028|Canada.Ontario
Eurylophella temporalis|09NBMAY-0654|Canada.New Brunswick
Eurylophella temporalis|09NBMAY-0586|Canada.New Brunswick
Eurylophella temporalis|09NBMAY-0583|Canada.New Brunswick
Eurylophella temporalis|09NBMAY-0573|Canada.New Brunswick
Eurylophella temporalis|09NBMAY-0564|Canada.New Brunswick
Eurylophella temporalis|09NBMAY-0558|Canada.New Brunswick
Eurylophella temporalis|09NBMAY-0555|Canada.New Brunswick
Eurylophella temporalis|09NBMAY-0194|Canada.New Brunswick
Eurylophella temporalis|09NBMAY-0192|Canada.New Brunswick
Eurylophella temporalis|09NBMAY-0191|Canada.New Brunswick
Eurylophella temporalis|SWRC-Et Moo 08|United States.Maine
Eurylophella temporalis|10-SWRC-0013|United States.Maine
Eurylophella temporalis|09NBMAY-0565|Canada.New Brunswick
Eurylophella temporalis|09NBMAY-0440|Canada.New Brunswick
Eurylophella temporalis
Eurylophella lutulenta|802 EL VT|United States.Vermont
Eurylophella lutulenta|SWRC-El Eme 48|United States.Vermont
Eurylophella lutulenta|SWRC-El Eme 41|United States.Vermont
Eurylophella lutulenta
Dentatella coxalis|SWRC-Ec NBS 03|United States.New Hampshire
Dentatella coxalis|SWRC-Ec NBS 01|United States.New Hampshire
Dentatella coxalis
Eurylophella sp.DHF1|SWRC-Et Sav6 11|United States.South Carolina
Eurylophella sp.DHF1|SWRC-Et Sav6 03|United States.South Carolina
Eurylophella sp.DHF1
Eurylophella enoensis|SWRC-Een Coon 12|United States.North Carolina
Eurylophella enoensis|SWRC-Een Coon 01|United States.North Carolina
Eurylophella enoensis|SWRC-Een Coon 11|United States.North Carolina
Eurylophella enoensis
Eurylophella temporalis group|SWRC-Et Sav6 22|United States.South Carolina
Eurylophella temporalis group
Eurylophella oviruptis|SWRC-Esw Ba 11|United States.North Carolina*
Eurylophella oviruptis|SWRC-Esw Ba 05|United States.North Carolina*
Eurylophella oviruptis|SWRC-Esw Ba 43|United States.North Carolina*
Eurylophella oviruptis|SWRC-Esw Ba 03|United States.North Carolina*
Eurylophella oviruptis|SWRC-Esw Ba 04|United States.North Carolina*
Eurylophella oviruptis*
Paraleptophlebia kirchneri|BIOUG00640-A11|United States.Tennessee*
Paraleptophlebia kirchneri|BIOUG00640-A10|United States.Tennessee*
Paraleptophlebia kirchneri*
Dannella lita|BIOUG00638-F07|Canada.Ontario
Dannella lita|BIOUG00638-F06|Canada.Ontario
Dannella lita
Dannella provonshai|07GSM-5955|United States.North Carolina
Dannella provonshai
Dannella lita|07PROBE-2453|Canada.Manitoba
Dannella lita|07PROBE-2447|Canada.Manitoba
Dannella lita
Dannella simplex|749 WCC PA|United States.Pennsylvania
Dannella simplex|748 WCC PA|United States.Pennsylvania
Dannella simplex|SWRC-Ds Che 03|United States.Alabama

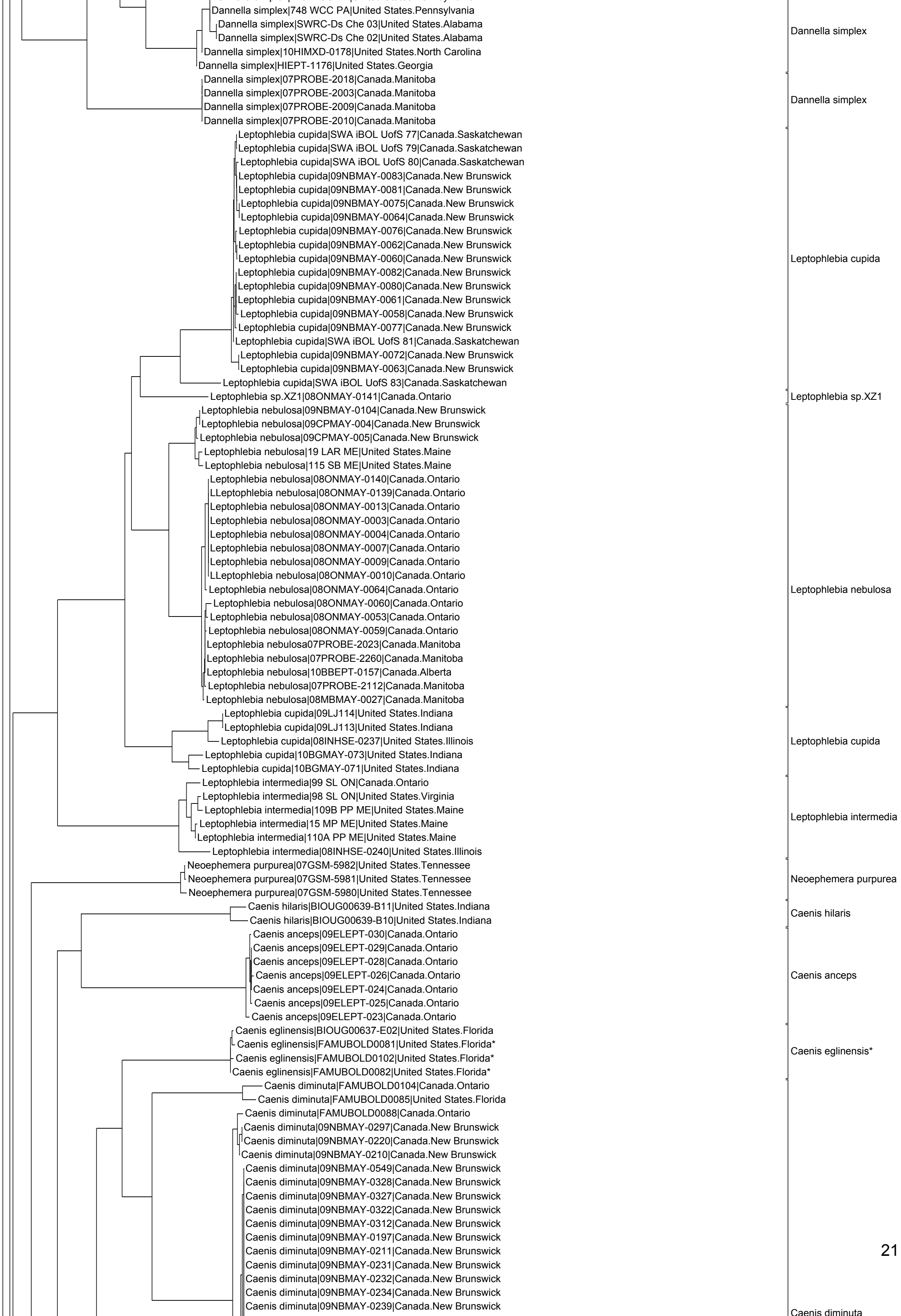

Dannella simplex|748 WCC PA|United States.Pennsylvania
Dannella simplex|SWRC-Ds Che 03|United States.Alabama
Dannella simplex|SWRC-Ds Che 02|United States.Alabama
Dannella simplex|10HIMXD-0178|United States.North Carolina
Dannella simplex|HIEPT-1176|United States.Georgia
Dannella simplex|07PROBE-2018|Canada.Manitoba
Dannella simplex|07PROBE-2003|Canada.Manitoba
Dannella simplex|07PROBE-2009|Canada.Manitoba
Dannella simplex|07PROBE-2010|Canada.Manitoba
Leptophlebia cupida|SWA iBOL UofS 77|Canada.Saskatchewan
Leptophlebia cupida|SWA iBOL UofS 79|Canada.Saskatchewan
Leptophlebia cupida|SWA iBOL UofS 80|Canada.Saskatchewan
Leptophlebia cupida|09NBMAY-0083|Canada.New Brunswick
Leptophlebia cupida|09NBMAY-0081|Canada.New Brunswick
Leptophlebia cupida|09NBMAY-0075|Canada.New Brunswick
Leptophlebia cupida|09NBMAY-0064|Canada.New Brunswick
Leptophlebia cupida|09NBMAY-0076|Canada.New Brunswick
Leptophlebia cupida|09NBMAY-0062|Canada.New Brunswick
Leptophlebia cupida|09NBMAY-0060|Canada.New Brunswick
Leptophlebia cupida|09NBMAY-0082|Canada.New Brunswick
Leptophlebia cupida|09NBMAY-0080|Canada.New Brunswick
Leptophlebia cupida|09NBMAY-0061|Canada.New Brunswick
Leptophlebia cupida|09NBMAY-0058|Canada.New Brunswick
Leptophlebia cupida|09NBMAY-0077|Canada.New Brunswick
Leptophlebia cupida|SWA iBOL UofS 81|Canada.Saskatchewan
Leptophlebia cupida|09NBMAY-0072|Canada.New Brunswick
Leptophlebia cupida|09NBMAY-0063|Canada.New Brunswick
Leptophlebia cupida|SWA iBOL UofS 83|Canada.Saskatchewan
Leptophlebia sp.XZ1|08ONMAY-0141|Canada.Ontario
Leptophlebia nebulosa|09NBMAY-0104|Canada.New Brunswick
Leptophlebia nebulosa|09CPMAY-004|Canada.New Brunswick
Leptophlebia nebulosa|09CPMAY-005|Canada.New Brunswick
Leptophlebia nebulosa|19 LAR ME|United States.Maine
Leptophlebia nebulosa|115 SB ME|United States.Maine
Leptophlebia nebulosa|08ONMAY-0140|Canada.Ontario
LLeptophlebia nebulosa|08ONMAY-0139|Canada.Ontario
Leptophlebia nebulosa|08ONMAY-0013|Canada.Ontario
Leptophlebia nebulosa|08ONMAY-0003|Canada.Ontario
Leptophlebia nebulosa|08ONMAY-0004|Canada.Ontario
Leptophlebia nebulosa|08ONMAY-0007|Canada.Ontario
Leptophlebia nebulosa|08ONMAY-0009|Canada.Ontario
LLeptophlebia nebulosa|08ONMAY-0010|Canada.Ontario
Leptophlebia nebulosa|08ONMAY-0064|Canada.Ontario
Leptophlebia nebulosa|08ONMAY-0060|Canada.Ontario
Leptophlebia nebulosa|08ONMAY-0053|Canada.Ontario
Leptophlebia nebulosa|08ONMAY-0059|Canada.Ontario
Leptophlebia nebulosa07PROBE-2023|Canada.Manitoba
Leptophlebia nebulosa|07PROBE-2260|Canada.Manitoba
Leptophlebia nebulosa|10BBEPT-0157|Canada.Alberta
Leptophlebia nebulosa|07PROBE-2112|Canada.Manitoba
Leptophlebia nebulosa|08MBMAY-0027|Canada.Manitoba
Leptophlebia cupida|09LJ114|United States.Indiana
Leptophlebia cupida|09LJ113|United States.Indiana
Leptophlebia cupida|08INHSE-0237|United States.Illinois
Leptophlebia cupida|10BGMAY-073|United States.Indiana
Leptophlebia cupida|10BGMAY-071|United States.Indiana
Leptophlebia intermedia|99 SL ON|Canada.Ontario
Leptophlebia intermedia|98 SL ON|United States.Virginia
Leptophlebia intermedia|109B PP ME|United States.Maine
Leptophlebia intermedia|15 MP ME|United States.Maine
Leptophlebia intermedia|110A PP ME|United States.Maine
Leptophlebia intermedia|08INHSE-0240|United States.Illinois
Neoephemera purpurea|07GSM-5982|United States.Tennessee
Neoephemera purpurea|07GSM-5981|United States.Tennessee
Neoephemera purpurea|07GSM-5980|United States.Tennessee
Caenis hilaris|BIOUG00639-B11|United States.Indiana
Caenis hilaris|BIOUG00639-B10|United States.Indiana
Caenis anceps|09ELEPT-030|Canada.Ontario
Caenis anceps|09ELEPT-029|Canada.Ontario
Caenis anceps|09ELEPT-028|Canada.Ontario
Caenis anceps|09ELEPT-026|Canada.Ontario
Caenis anceps|09ELEPT-024|Canada.Ontario
Caenis anceps|09ELEPT-025|Canada.Ontario
Caenis anceps|09ELEPT-023|Canada.Ontario
Caenis eglinensis|BIOUG00637-E02|United States.Florida
Caenis eglinensis|FAMUBOLD0081|United States.Florida*
Caenis eglinensis|FAMUBOLD0102|United States.Florida*
Caenis eglinensis|FAMUBOLD0082|United States.Florida*
Caenis diminuta|FAMUBOLD0104|Canada.Ontario
Caenis diminuta|FAMUBOLD0085|United States.Florida
Caenis diminuta|FAMUBOLD0088|Canada.Ontario
Caenis diminuta|09NBMAY-0297|Canada.New Brunswick
Caenis diminuta|09NBMAY-0220|Canada.New Brunswick
Caenis diminuta|09NBMAY-0210|Canada.New Brunswick
Caenis diminuta|09NBMAY-0549|Canada.New Brunswick
Caenis diminuta|09NBMAY-0328|Canada.New Brunswick
Caenis diminuta|09NBMAY-0327|Canada.New Brunswick
Caenis diminuta|09NBMAY-0322|Canada.New Brunswick
Caenis diminuta|09NBMAY-0312|Canada.New Brunswick
Caenis diminuta|09NBMAY-0197|Canada.New Brunswick
Caenis diminuta|09NBMAY-0211|Canada.New Brunswick
Caenis diminuta|09NBMAY-0231|Canada.New Brunswick
Caenis diminuta|09NBMAY-0232|Canada.New Brunswick
Caenis diminuta|09NBMAY-0234|Canada.New Brunswick
Caenis diminuta|09NBMAY-0239|Canada.New Brunswick
Dannella simplex
Dannella simplex
Leptophlebia cupida
Leptophlebia sp.XZ1
Leptophlebia nebulosa
Leptophlebia cupida
Leptophlebia intermedia
Neoephemera purpurea
Caenis hilaris
Caenis anceps
Caenis eglinensis*
Caenis diminuta

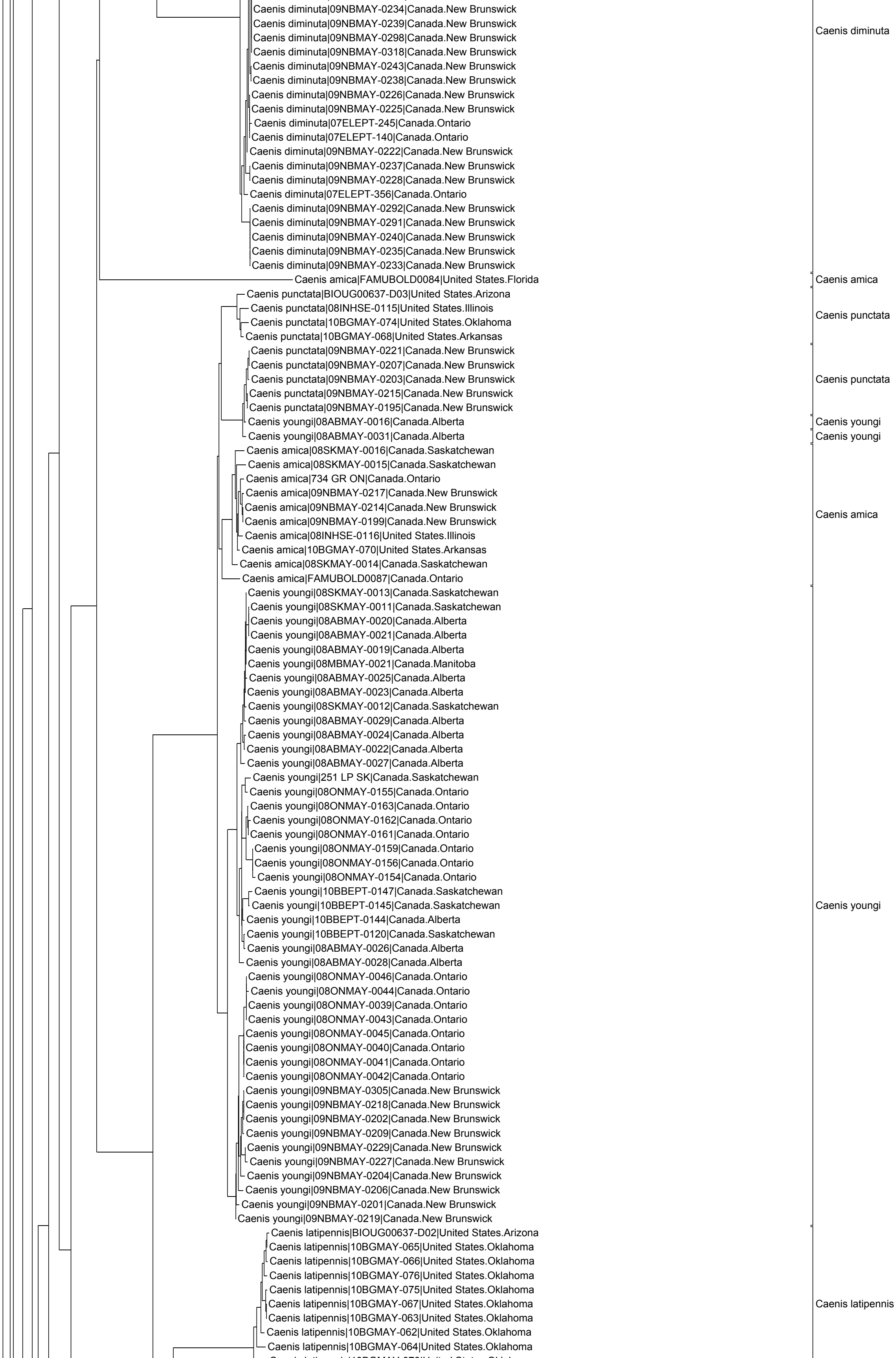

Caenis diminuta|09NBMAY-0234|Canada.New Brunswick
Caenis diminuta|09NBMAY-0239|Canada.New Brunswick
Caenis diminuta|09NBMAY-0298|Canada.New Brunswick
Caenis diminuta|09NBMAY-0318|Canada.New Brunswick
Caenis diminuta|09NBMAY-0243|Canada.New Brunswick
Caenis diminuta|09NBMAY-0238|Canada.New Brunswick
Caenis diminuta|09NBMAY-0226|Canada.New Brunswick
Caenis diminuta|09NBMAY-0225|Canada.New Brunswick
Caenis diminuta|07ELEPT-245|Canada.Ontario
Caenis diminuta|07ELEPT-140|Canada.Ontario
Caenis diminuta|09NBMAY-0222|Canada.New Brunswick
Caenis diminuta|09NBMAY-0237|Canada.New Brunswick
Caenis diminuta|09NBMAY-0228|Canada.New Brunswick
Caenis diminuta|07ELEPT-356|Canada.Ontario
Caenis diminuta|09NBMAY-0292|Canada.New Brunswick
Caenis diminuta|09NBMAY-0291|Canada.New Brunswick
Caenis diminuta|09NBMAY-0240|Canada.New Brunswick
Caenis diminuta|09NBMAY-0235|Canada.New Brunswick
Caenis diminuta|09NBMAY-0233|Canada.New Brunswick
Caenis diminuta
Caenis amica|FAMUBOLD0084|United States.Florida
Caenis amica
Caenis punctata|BIOUG00637-D03|United States.Arizona
Caenis punctata|08INHSE-0115|United States.Illinois
Caenis punctata|10BGMAY-074|United States.Oklahoma
Caenis punctata|10BGMAY-068|United States.Arkansas
Caenis punctata
Caenis punctata|09NBMAY-0221|Canada.New Brunswick
Caenis punctata|09NBMAY-0207|Canada.New Brunswick
Caenis punctata|09NBMAY-0203|Canada.New Brunswick
Caenis punctata|09NBMAY-0215|Canada.New Brunswick
Caenis punctata|09NBMAY-0195|Canada.New Brunswick
Caenis punctata
Caenis youngi|08ABMAY-0016|Canada.Alberta
Caenis youngi
Caenis youngi|08ABMAY-0031|Canada.Alberta
Caenis youngi
Caenis amica|08SKMAY-0016|Canada.Saskatchewan
Caenis amica|08SKMAY-0015|Canada.Saskatchewan
Caenis amica|734 GR ON|Canada.Ontario
Caenis amica|09NBMAY-0217|Canada.New Brunswick
Caenis amica|09NBMAY-0214|Canada.New Brunswick
Caenis amica|09NBMAY-0199|Canada.New Brunswick
Caenis amica|08INHSE-0116|United States.Illinois
Caenis amica|10BGMAY-070|United States.Arkansas
Caenis amica|08SKMAY-0014|Canada.Saskatchewan
Caenis amica|FAMUBOLD0087|Canada.Ontario
Caenis amica
Caenis youngi|08SKMAY-0013|Canada.Saskatchewan
Caenis youngi|08SKMAY-0011|Canada.Saskatchewan
Caenis youngi|08ABMAY-0020|Canada.Alberta
Caenis youngi|08ABMAY-0021|Canada.Alberta
Caenis youngi|08ABMAY-0019|Canada.Alberta
Caenis youngi|08MBMAY-0021|Canada.Manitoba
Caenis youngi|08ABMAY-0025|Canada.Alberta
Caenis youngi|08ABMAY-0023|Canada.Alberta
Caenis youngi|08SKMAY-0012|Canada.Saskatchewan
Caenis youngi|08ABMAY-0029|Canada.Alberta
Caenis youngi|08ABMAY-0024|Canada.Alberta
Caenis youngi|08ABMAY-0022|Canada.Alberta
Caenis youngi|08ABMAY-0027|Canada.Alberta
Caenis youngi|251 LP SK|Canada.Saskatchewan
Caenis youngi|08ONMAY-0155|Canada.Ontario
Caenis youngi|08ONMAY-0163|Canada.Ontario
Caenis youngi|08ONMAY-0162|Canada.Ontario
Caenis youngi|08ONMAY-0161|Canada.Ontario
Caenis youngi|08ONMAY-0159|Canada.Ontario
Caenis youngi|08ONMAY-0156|Canada.Ontario
Caenis youngi|08ONMAY-0154|Canada.Ontario
Caenis youngi|10BBEPT-0147|Canada.Saskatchewan
Caenis youngi|10BBEPT-0145|Canada.Saskatchewan
Caenis youngi
Caenis youngi|10BBEPT-0144|Canada.Alberta
Caenis youngi|10BBEPT-0120|Canada.Saskatchewan
Caenis youngi|08ABMAY-0026|Canada.Alberta
Caenis youngi|08ABMAY-0028|Canada.Alberta
Caenis youngi|08ONMAY-0046|Canada.Ontario
Caenis youngi|08ONMAY-0044|Canada.Ontario
Caenis youngi|08ONMAY-0039|Canada.Ontario
Caenis youngi|08ONMAY-0043|Canada.Ontario
Caenis youngi|08ONMAY-0045|Canada.Ontario
Caenis youngi|08ONMAY-0040|Canada.Ontario
Caenis youngi|08ONMAY-0041|Canada.Ontario
Caenis youngi|08ONMAY-0042|Canada.Ontario
Caenis youngi|09NBMAY-0305|Canada.New Brunswick
Caenis youngi|09NBMAY-0218|Canada.New Brunswick
Caenis youngi|09NBMAY-0202|Canada.New Brunswick
Caenis youngi|09NBMAY-0209|Canada.New Brunswick
Caenis youngi|09NBMAY-0229|Canada.New Brunswick
Caenis youngi|09NBMAY-0227|Canada.New Brunswick
Caenis youngi|09NBMAY-0204|Canada.New Brunswick
Caenis youngi|09NBMAY-0206|Canada.New Brunswick
Caenis youngi|09NBMAY-0201|Canada.New Brunswick
Caenis youngi|09NBMAY-0219|Canada.New Brunswick
Caenis latipennis|BIOUG00637-D02|United States.Arizona
Caenis latipennis|10BGMAY-065|United States.Oklahoma
Caenis latipennis|10BGMAY-066|United States.Oklahoma
Caenis latipennis|10BGMAY-076|United States.Oklahoma
Caenis latipennis|10BGMAY-075|United States.Oklahoma
Caenis latipennis|10BGMAY-067|United States.Oklahoma
Caenis latipennis
Caenis latipennis|10BGMAY-063|United States.Oklahoma
Caenis latipennis|10BGMAY-062|United States.Oklahoma
Caenis latipennis|10BGMAY-064|United States.Oklahoma

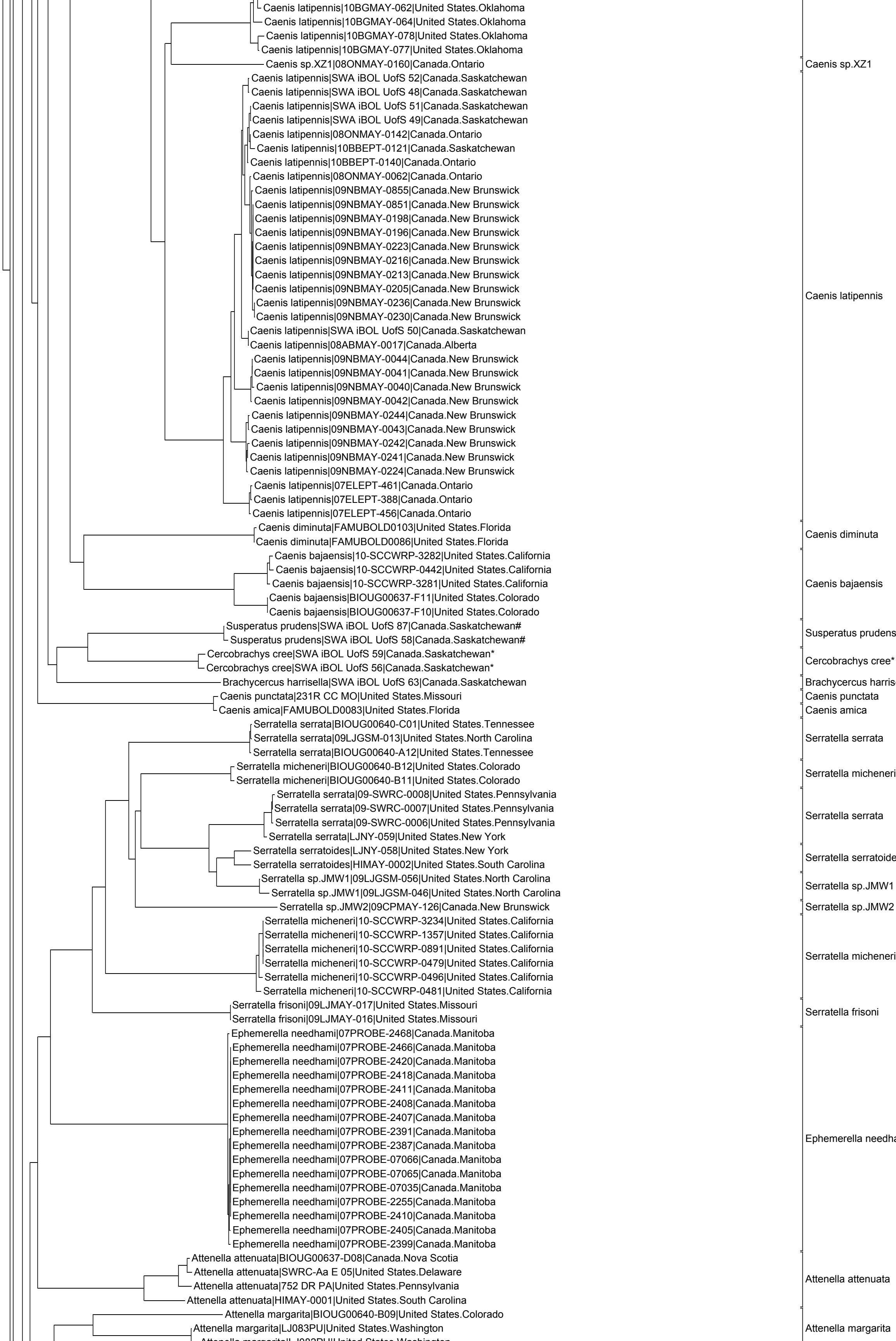

Caenis latipennis|10BGMAY-062|United States.Oklahoma
Caenis latipennis|10BGMAY-064|United States.Oklahoma
Caenis latipennis|10BGMAY-078|United States.Oklahoma
Caenis latipennis|10BGMAY-077|United States.Oklahoma
Caenis sp.XZ1|08ONMAY-0160|Canada.Ontario
Caenis latipennis|SWA iBOL UofS 52|Canada.Saskatchewan
Caenis latipennis|SWA iBOL UofS 48|Canada.Saskatchewan
Caenis latipennis|SWA iBOL UofS 51|Canada.Saskatchewan
Caenis latipennis|SWA iBOL UofS 49|Canada.Saskatchewan
Caenis latipennis|08ONMAY-0142|Canada.Ontario
Caenis latipennis|10BBEPT-0121|Canada.Saskatchewan
Caenis latipennis|10BBEPT-0140|Canada.Ontario
Caenis latipennis|08ONMAY-0062|Canada.Ontario
Caenis latipennis|09NBMAY-0855|Canada.New Brunswick
Caenis latipennis|09NBMAY-0851|Canada.New Brunswick
Caenis latipennis|09NBMAY-0198|Canada.New Brunswick
Caenis latipennis|09NBMAY-0196|Canada.New Brunswick
Caenis latipennis|09NBMAY-0223|Canada.New Brunswick
Caenis latipennis|09NBMAY-0216|Canada.New Brunswick
Caenis latipennis|09NBMAY-0213|Canada.New Brunswick
Caenis latipennis|09NBMAY-0205|Canada.New Brunswick
Caenis latipennis|09NBMAY-0236|Canada.New Brunswick
Caenis latipennis|09NBMAY-0230|Canada.New Brunswick
Caenis latipennis|SWA iBOL UofS 50|Canada.Saskatchewan
Caenis latipennis|08ABMAY-0017|Canada.Alberta
Caenis latipennis|09NBMAY-0044|Canada.New Brunswick
Caenis latipennis|09NBMAY-0041|Canada.New Brunswick
Caenis latipennis|09NBMAY-0040|Canada.New Brunswick
Caenis latipennis|09NBMAY-0042|Canada.New Brunswick
Caenis latipennis|09NBMAY-0244|Canada.New Brunswick
Caenis latipennis|09NBMAY-0043|Canada.New Brunswick
Caenis latipennis|09NBMAY-0242|Canada.New Brunswick
Caenis latipennis|09NBMAY-0241|Canada.New Brunswick
Caenis latipennis|09NBMAY-0224|Canada.New Brunswick
Caenis latipennis|07ELEPT-461|Canada.Ontario
Caenis latipennis|07ELEPT-388|Canada.Ontario
Caenis latipennis|07ELEPT-456|Canada.Ontario
Caenis diminuta|FAMUBOLD0103|United States.Florida
Caenis diminuta|FAMUBOLD0086|United States.Florida
Caenis bajaensis|10-SCCWRP-3282|United States.California
Caenis bajaensis|10-SCCWRP-0442|United States.California
Caenis bajaensis|10-SCCWRP-3281|United States.California
Caenis bajaensis|BIOUG00637-F11|United States.Colorado
Caenis bajaensis|BIOUG00637-F10|United States.Colorado
Susperatus prudens|SWA iBOL UofS 87|Canada.Saskatchewan#
Susperatus prudens|SWA iBOL UofS 58|Canada.Saskatchewan#
Cercobrachys cree|SWA iBOL UofS 59|Canada.Saskatchewan*
Cercobrachys cree|SWA iBOL UofS 56|Canada.Saskatchewan*
Brachycercus harrisella|SWA iBOL UofS 63|Canada.Saskatchewan
Caenis punctata|231R CC MO|United States.Missouri
Caenis amica|FAMUBOLD0083|United States.Florida
Serratella serrata|BIOUG00640-C01|United States.Tennessee
Serratella serrata|09LJGSM-013|United States.North Carolina
Serratella serrata|BIOUG00640-A12|United States.Tennessee
Serratella micheneri|BIOUG00640-B12|United States.Colorado
Serratella micheneri|BIOUG00640-B11|United States.Colorado
Serratella serrata|09-SWRC-0008|United States.Pennsylvania
Serratella serrata|09-SWRC-0007|United States.Pennsylvania
Serratella serrata|09-SWRC-0006|United States.Pennsylvania
Serratella serrata|LJNY-059|United States.New York
Serratella serratoides|LJNY-058|United States.New York
Serratella serratoides|HIMAY-0002|United States.South Carolina
Serratella sp.JMW1|09LJGSM-056|United States.North Carolina
Serratella sp.JMW1|09LJGSM-046|United States.North Carolina
Serratella sp.JMW2|09CPMAY-126|Canada.New Brunswick
Serratella micheneri|10-SCCWRP-3234|United States.California
Serratella micheneri|10-SCCWRP-1357|United States.California
Serratella micheneri|10-SCCWRP-0891|United States.California
Serratella micheneri|10-SCCWRP-0479|United States.California
Serratella micheneri|10-SCCWRP-0496|United States.California
Serratella micheneri|10-SCCWRP-0481|United States.California
Serratella frisoni|09LJMAY-017|United States.Missouri
Serratella frisoni|09LJMAY-016|United States.Missouri
Ephemerella needhami|07PROBE-2468|Canada.Manitoba
Ephemerella needhami|07PROBE-2466|Canada.Manitoba
Ephemerella needhami|07PROBE-2420|Canada.Manitoba
Ephemerella needhami|07PROBE-2418|Canada.Manitoba
Ephemerella needhami|07PROBE-2411|Canada.Manitoba
Ephemerella needhami|07PROBE-2408|Canada.Manitoba
Ephemerella needhami|07PROBE-2407|Canada.Manitoba
Ephemerella needhami|07PROBE-2391|Canada.Manitoba
Ephemerella needhami|07PROBE-2387|Canada.Manitoba
Ephemerella needhami|07PROBE-07066|Canada.Manitoba
Ephemerella needhami|07PROBE-07065|Canada.Manitoba
Ephemerella needhami|07PROBE-07035|Canada.Manitoba
Ephemerella needhami|07PROBE-2255|Canada.Manitoba
Ephemerella needhami|07PROBE-2410|Canada.Manitoba
Ephemerella needhami|07PROBE-2405|Canada.Manitoba
Ephemerella needhami|07PROBE-2399|Canada.Manitoba
Attenella attenuata|BIOUG00637-D08|Canada.Nova Scotia
Attenella attenuata|SWRC-Aa E 05|United States.Delaware
Attenella attenuata|752 DR PA|United States.Pennsylvania
Attenella attenuata|HIMAY-0001|United States.South Carolina
Attenella margarita|BIOUG00640-B09|United States.Colorado
Attenella margarita|LJ083PU|United States.Washington
Caenis sp.XZ1
Caenis latipennis
Caenis diminuta
Caenis bajaensis
Susperatus prudens#
Cercobrachys cree*
Brachycercus harrisella
Caenis punctata
Caenis amica
Serratella serrata
Serratella micheneri
Serratella serrata
Serratella serratoides
Serratella sp.JMW1
Serratella sp.JMW2
Serratella micheneri
Serratella frisoni
Ephemerella needhami
Attenella attenuata
Attenella margarita

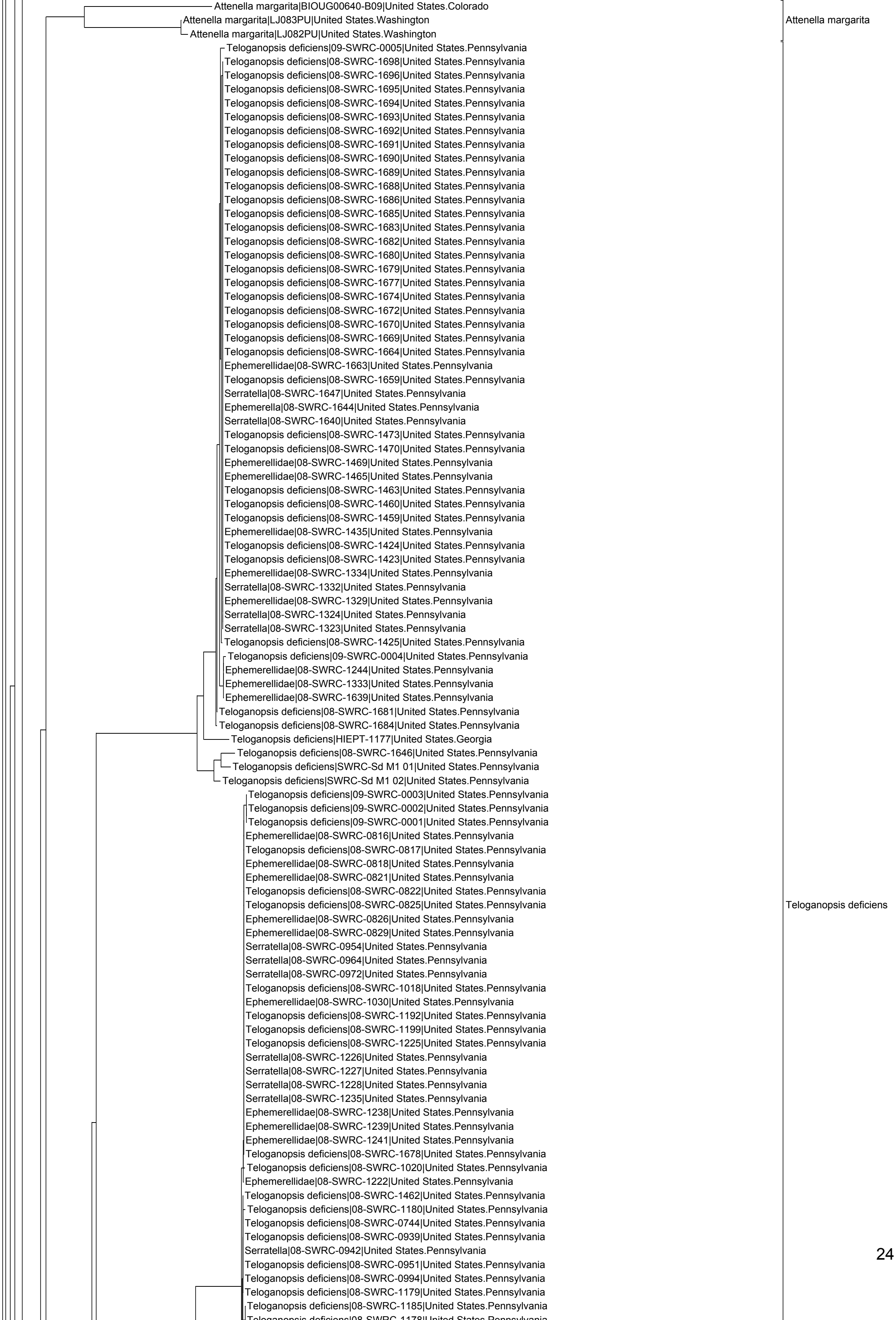
Attenella margarita|BIOUG00640-B09|United States.Colorado
Attenella margarita|LJ083PU|United States.Washington
Attenella margarita|LJ082PU|United States.Washington
Teloganopsis deficiens|09-SWRC-0005|United States.Pennsylvania
Teloganopsis deficiens|08-SWRC-1698|United States.Pennsylvania
Teloganopsis deficiens|08-SWRC-1696|United States.Pennsylvania
Teloganopsis deficiens|08-SWRC-1695|United States.Pennsylvania
Teloganopsis deficiens|08-SWRC-1694|United States.Pennsylvania
Teloganopsis deficiens|08-SWRC-1693|United States.Pennsylvania
Teloganopsis deficiens|08-SWRC-1692|United States.Pennsylvania
Teloganopsis deficiens|08-SWRC-1691|United States.Pennsylvania
Teloganopsis deficiens|08-SWRC-1690|United States.Pennsylvania
Teloganopsis deficiens|08-SWRC-1689|United States.Pennsylvania
Teloganopsis deficiens|08-SWRC-1688|United States.Pennsylvania
Teloganopsis deficiens|08-SWRC-1686|United States.Pennsylvania
Teloganopsis deficiens|08-SWRC-1685|United States.Pennsylvania
Teloganopsis deficiens|08-SWRC-1683|United States.Pennsylvania
Teloganopsis deficiens|08-SWRC-1682|United States.Pennsylvania
Teloganopsis deficiens|08-SWRC-1680|United States.Pennsylvania
Teloganopsis deficiens|08-SWRC-1679|United States.Pennsylvania
Teloganopsis deficiens|08-SWRC-1677|United States.Pennsylvania
Teloganopsis deficiens|08-SWRC-1674|United States.Pennsylvania
Teloganopsis deficiens|08-SWRC-1672|United States.Pennsylvania
Teloganopsis deficiens|08-SWRC-1670|United States.Pennsylvania
Teloganopsis deficiens|08-SWRC-1669|United States.Pennsylvania
Teloganopsis deficiens|08-SWRC-1664|United States.Pennsylvania
Ephemerellidae|08-SWRC-1663|United States.Pennsylvania
Teloganopsis deficiens|08-SWRC-1659|United States.Pennsylvania
Serratella|08-SWRC-1647|United States.Pennsylvania
Ephemerella|08-SWRC-1644|United States.Pennsylvania
Serratella|08-SWRC-1640|United States.Pennsylvania
Teloganopsis deficiens|08-SWRC-1473|United States.Pennsylvania
Teloganopsis deficiens|08-SWRC-1470|United States.Pennsylvania
Ephemerellidae|08-SWRC-1469|United States.Pennsylvania
Ephemerellidae|08-SWRC-1465|United States.Pennsylvania
Teloganopsis deficiens|08-SWRC-1463|United States.Pennsylvania
Teloganopsis deficiens|08-SWRC-1460|United States.Pennsylvania
Teloganopsis deficiens|08-SWRC-1459|United States.Pennsylvania
Ephemerellidae|08-SWRC-1435|United States.Pennsylvania
Teloganopsis deficiens|08-SWRC-1424|United States.Pennsylvania
Teloganopsis deficiens|08-SWRC-1423|United States.Pennsylvania
Ephemerellidae|08-SWRC-1334|United States.Pennsylvania
Serratella|08-SWRC-1332|United States.Pennsylvania
Ephemerellidae|08-SWRC-1329|United States.Pennsylvania
Serratella|08-SWRC-1324|United States.Pennsylvania
Serratella|08-SWRC-1323|United States.Pennsylvania
Teloganopsis deficiens|08-SWRC-1425|United States.Pennsylvania
Teloganopsis deficiens|09-SWRC-0004|United States.Pennsylvania
Ephemerellidae|08-SWRC-1244|United States.Pennsylvania
Ephemerellidae|08-SWRC-1333|United States.Pennsylvania
Ephemerellidae|08-SWRC-1639|United States.Pennsylvania
Teloganopsis deficiens|08-SWRC-1681|United States.Pennsylvania
Teloganopsis deficiens|08-SWRC-1684|United States.Pennsylvania
Teloganopsis deficiens|HIEPT-1177|United States.Georgia
Teloganopsis deficiens|08-SWRC-1646|United States.Pennsylvania
Teloganopsis deficiens|SWRC-Sd M1 01|United States.Pennsylvania
Teloganopsis deficiens|SWRC-Sd M1 02|United States.Pennsylvania
Teloganopsis deficiens|09-SWRC-0003|United States.Pennsylvania
Teloganopsis deficiens|09-SWRC-0002|United States.Pennsylvania
Teloganopsis deficiens|09-SWRC-0001|United States.Pennsylvania
Ephemerellidae|08-SWRC-0816|United States.Pennsylvania
Teloganopsis deficiens|08-SWRC-0817|United States.Pennsylvania
Ephemerellidae|08-SWRC-0818|United States.Pennsylvania
Ephemerellidae|08-SWRC-0821|United States.Pennsylvania
Teloganopsis deficiens|08-SWRC-0822|United States.Pennsylvania
Teloganopsis deficiens|08-SWRC-0825|United States.Pennsylvania
Ephemerellidae|08-SWRC-0826|United States.Pennsylvania
Ephemerellidae|08-SWRC-0829|United States.Pennsylvania
Serratella|08-SWRC-0954|United States.Pennsylvania
Serratella|08-SWRC-0964|United States.Pennsylvania
Serratella|08-SWRC-0972|United States.Pennsylvania
Teloganopsis deficiens|08-SWRC-1018|United States.Pennsylvania
Ephemerellidae|08-SWRC-1030|United States.Pennsylvania
Teloganopsis deficiens|08-SWRC-1192|United States.Pennsylvania
Teloganopsis deficiens|08-SWRC-1199|United States.Pennsylvania
Teloganopsis deficiens|08-SWRC-1225|United States.Pennsylvania
Serratella|08-SWRC-1226|United States.Pennsylvania
Serratella|08-SWRC-1227|United States.Pennsylvania
Serratella|08-SWRC-1228|United States.Pennsylvania
Serratella|08-SWRC-1235|United States.Pennsylvania
Ephemerellidae|08-SWRC-1238|United States.Pennsylvania
Ephemerellidae|08-SWRC-1239|United States.Pennsylvania
Ephemerellidae|08-SWRC-1241|United States.Pennsylvania
Teloganopsis deficiens|08-SWRC-1678|United States.Pennsylvania
Teloganopsis deficiens|08-SWRC-1020|United States.Pennsylvania
Ephemerellidae|08-SWRC-1222|United States.Pennsylvania
Teloganopsis deficiens|08-SWRC-1462|United States.Pennsylvania
Teloganopsis deficiens|08-SWRC-1180|United States.Pennsylvania
Teloganopsis deficiens|08-SWRC-0744|United States.Pennsylvania
Teloganopsis deficiens|08-SWRC-0939|United States.Pennsylvania
Serratella|08-SWRC-0942|United States.Pennsylvania
Teloganopsis deficiens|08-SWRC-0951|United States.Pennsylvania
Teloganopsis deficiens|08-SWRC-0994|United States.Pennsylvania
Teloganopsis deficiens|08-SWRC-1179|United States.Pennsylvania
Teloganopsis deficiens|08-SWRC-1185|United States.Pennsylvania
Teloganopsis deficiens|08-SWRC-1178|United States.Pennsylvania
Attenella margarita
Teloganopsis deficiens

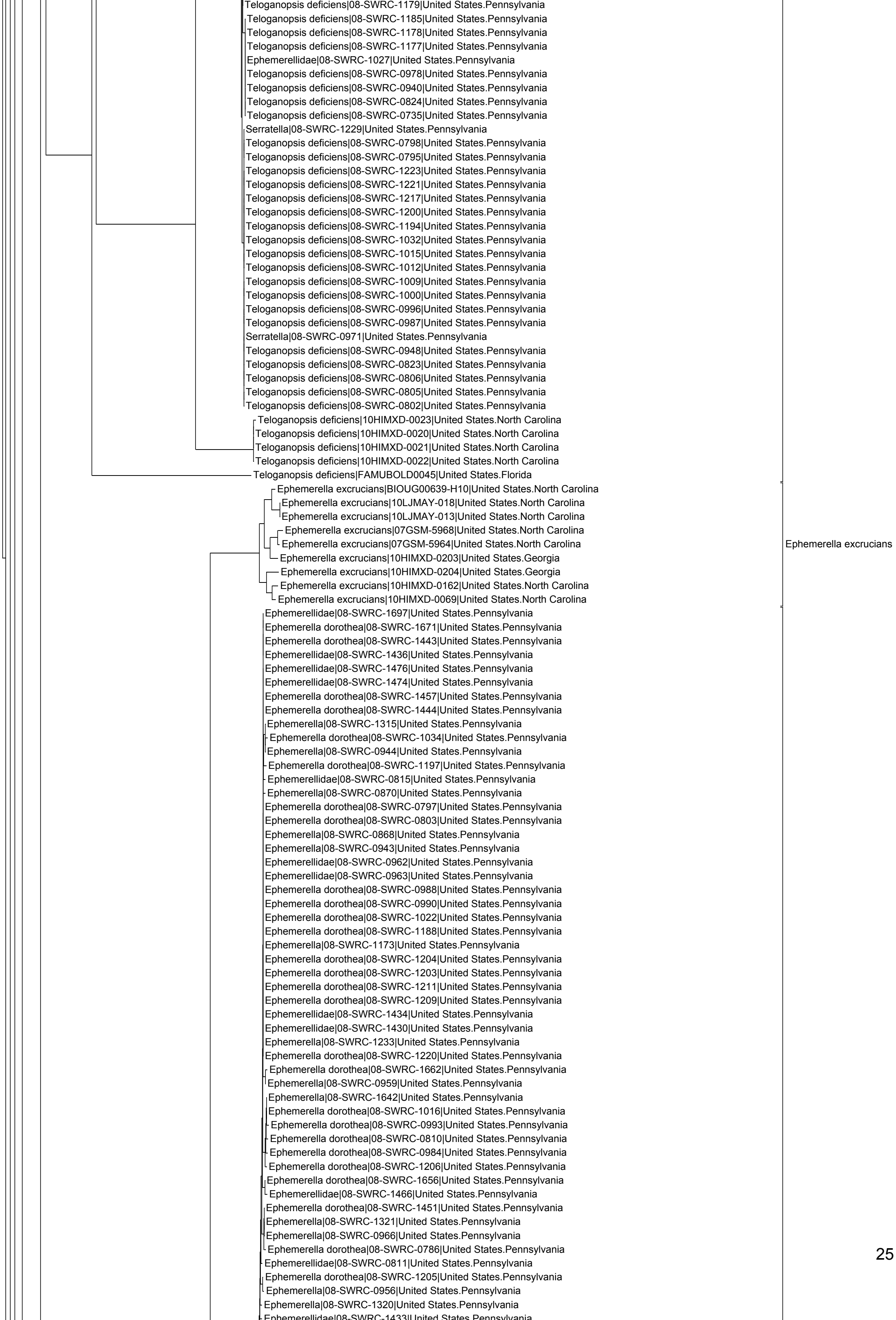
Teloganopsis deficiens|08-SWRC-1179|United States.Pennsylvania
Teloganopsis deficiens|08-SWRC-1185|United States.Pennsylvania
Teloganopsis deficiens|08-SWRC-1178|United States.Pennsylvania
Teloganopsis deficiens|08-SWRC-1177|United States.Pennsylvania
Ephemerellidae|08-SWRC-1027|United States.Pennsylvania
Teloganopsis deficiens|08-SWRC-0978|United States.Pennsylvania
Teloganopsis deficiens|08-SWRC-0940|United States.Pennsylvania
Teloganopsis deficiens|08-SWRC-0824|United States.Pennsylvania
Teloganopsis deficiens|08-SWRC-0735|United States.Pennsylvania
Serratella|08-SWRC-1229|United States.Pennsylvania
Teloganopsis deficiens|08-SWRC-0798|United States.Pennsylvania
Teloganopsis deficiens|08-SWRC-0795|United States.Pennsylvania
Teloganopsis deficiens|08-SWRC-1223|United States.Pennsylvania
Teloganopsis deficiens|08-SWRC-1221|United States.Pennsylvania
Teloganopsis deficiens|08-SWRC-1217|United States.Pennsylvania
Teloganopsis deficiens|08-SWRC-1200|United States.Pennsylvania
Teloganopsis deficiens|08-SWRC-1194|United States.Pennsylvania
Teloganopsis deficiens|08-SWRC-1032|United States.Pennsylvania
Teloganopsis deficiens|08-SWRC-1015|United States.Pennsylvania
Teloganopsis deficiens|08-SWRC-1012|United States.Pennsylvania
Teloganopsis deficiens|08-SWRC-1009|United States.Pennsylvania
Teloganopsis deficiens|08-SWRC-1000|United States.Pennsylvania
Teloganopsis deficiens|08-SWRC-0996|United States.Pennsylvania
Teloganopsis deficiens|08-SWRC-0987|United States.Pennsylvania
Serratella|08-SWRC-0971|United States.Pennsylvania
Teloganopsis deficiens|08-SWRC-0948|United States.Pennsylvania
Teloganopsis deficiens|08-SWRC-0823|United States.Pennsylvania
Teloganopsis deficiens|08-SWRC-0806|United States.Pennsylvania
Teloganopsis deficiens|08-SWRC-0805|United States.Pennsylvania
Teloganopsis deficiens|08-SWRC-0802|United States.Pennsylvania
Teloganopsis deficiens|10HIMXD-0023|United States.North Carolina
Teloganopsis deficiens|10HIMXD-0020|United States.North Carolina
Teloganopsis deficiens|10HIMXD-0021|United States.North Carolina
Teloganopsis deficiens|10HIMXD-0022|United States.North Carolina
Teloganopsis deficiens|FAMUBOLD0045|United States.Florida
Ephemerella excrucians|BIOUG00639-H10|United States.North Carolina
Ephemerella excrucians|10LJMAY-018|United States.North Carolina
Ephemerella excrucians|10LJMAY-013|United States.North Carolina
Ephemerella excrucians|07GSM-5968|United States.North Carolina
Ephemerella excrucians|07GSM-5964|United States.North Carolina
Ephemerella excrucians|10HIMXD-0203|United States.Georgia
Ephemerella excrucians|10HIMXD-0204|United States.Georgia
Ephemerella excrucians|10HIMXD-0162|United States.North Carolina
Ephemerella excrucians|10HIMXD-0069|United States.North Carolina
Ephemerella excrucians
Ephemerellidae|08-SWRC-1697|United States.Pennsylvania
Ephemerella dorothea|08-SWRC-1671|United States.Pennsylvania
Ephemerella dorothea|08-SWRC-1443|United States.Pennsylvania
Ephemerellidae|08-SWRC-1436|United States.Pennsylvania
Ephemerellidae|08-SWRC-1476|United States.Pennsylvania
Ephemerellidae|08-SWRC-1474|United States.Pennsylvania
Ephemerella dorothea|08-SWRC-1457|United States.Pennsylvania
Ephemerella dorothea|08-SWRC-1444|United States.Pennsylvania
Ephemerella|08-SWRC-1315|United States.Pennsylvania
Ephemerella dorothea|08-SWRC-1034|United States.Pennsylvania
Ephemerella|08-SWRC-0944|United States.Pennsylvania
Ephemerella dorothea|08-SWRC-1197|United States.Pennsylvania
Ephemerellidae|08-SWRC-0815|United States.Pennsylvania
Ephemerella|08-SWRC-0870|United States.Pennsylvania
Ephemerella dorothea|08-SWRC-0797|United States.Pennsylvania
Ephemerella dorothea|08-SWRC-0803|United States.Pennsylvania
Ephemerella|08-SWRC-0868|United States.Pennsylvania
Ephemerella|08-SWRC-0943|United States.Pennsylvania
Ephemerellidae|08-SWRC-0962|United States.Pennsylvania
Ephemerellidae|08-SWRC-0963|United States.Pennsylvania
Ephemerella dorothea|08-SWRC-0988|United States.Pennsylvania
Ephemerella dorothea|08-SWRC-0990|United States.Pennsylvania
Ephemerella dorothea|08-SWRC-1022|United States.Pennsylvania
Ephemerella dorothea|08-SWRC-1188|United States.Pennsylvania
Ephemerella|08-SWRC-1173|United States.Pennsylvania
Ephemerella dorothea|08-SWRC-1204|United States.Pennsylvania
Ephemerella dorothea|08-SWRC-1203|United States.Pennsylvania
Ephemerella dorothea|08-SWRC-1211|United States.Pennsylvania
Ephemerella dorothea|08-SWRC-1209|United States.Pennsylvania
Ephemerellidae|08-SWRC-1434|United States.Pennsylvania
Ephemerellidae|08-SWRC-1430|United States.Pennsylvania
Ephemerella|08-SWRC-1233|United States.Pennsylvania
Ephemerella dorothea|08-SWRC-1220|United States.Pennsylvania
Ephemerella dorothea|08-SWRC-1662|United States.Pennsylvania
Ephemerella|08-SWRC-0959|United States.Pennsylvania
Ephemerella|08-SWRC-1642|United States.Pennsylvania
Ephemerella dorothea|08-SWRC-1016|United States.Pennsylvania
Ephemerella dorothea|08-SWRC-0993|United States.Pennsylvania
Ephemerella dorothea|08-SWRC-0810|United States.Pennsylvania
Ephemerella dorothea|08-SWRC-0984|United States.Pennsylvania
Ephemerella dorothea|08-SWRC-1206|United States.Pennsylvania
Ephemerella dorothea|08-SWRC-1656|United States.Pennsylvania
Ephemerellidae|08-SWRC-1466|United States.Pennsylvania
Ephemerella dorothea|08-SWRC-1451|United States.Pennsylvania
Ephemerella|08-SWRC-1321|United States.Pennsylvania
Ephemerella|08-SWRC-0966|United States.Pennsylvania
Ephemerella dorothea|08-SWRC-0786|United States.Pennsylvania
Ephemerellidae|08-SWRC-0811|United States.Pennsylvania
Ephemerella dorothea|08-SWRC-1205|United States.Pennsylvania
Ephemerella|08-SWRC-0956|United States.Pennsylvania
Ephemerella|08-SWRC-1320|United States.Pennsylvania
Ephemerellidae|08-SWRC-1433|United States.Pennsylvania

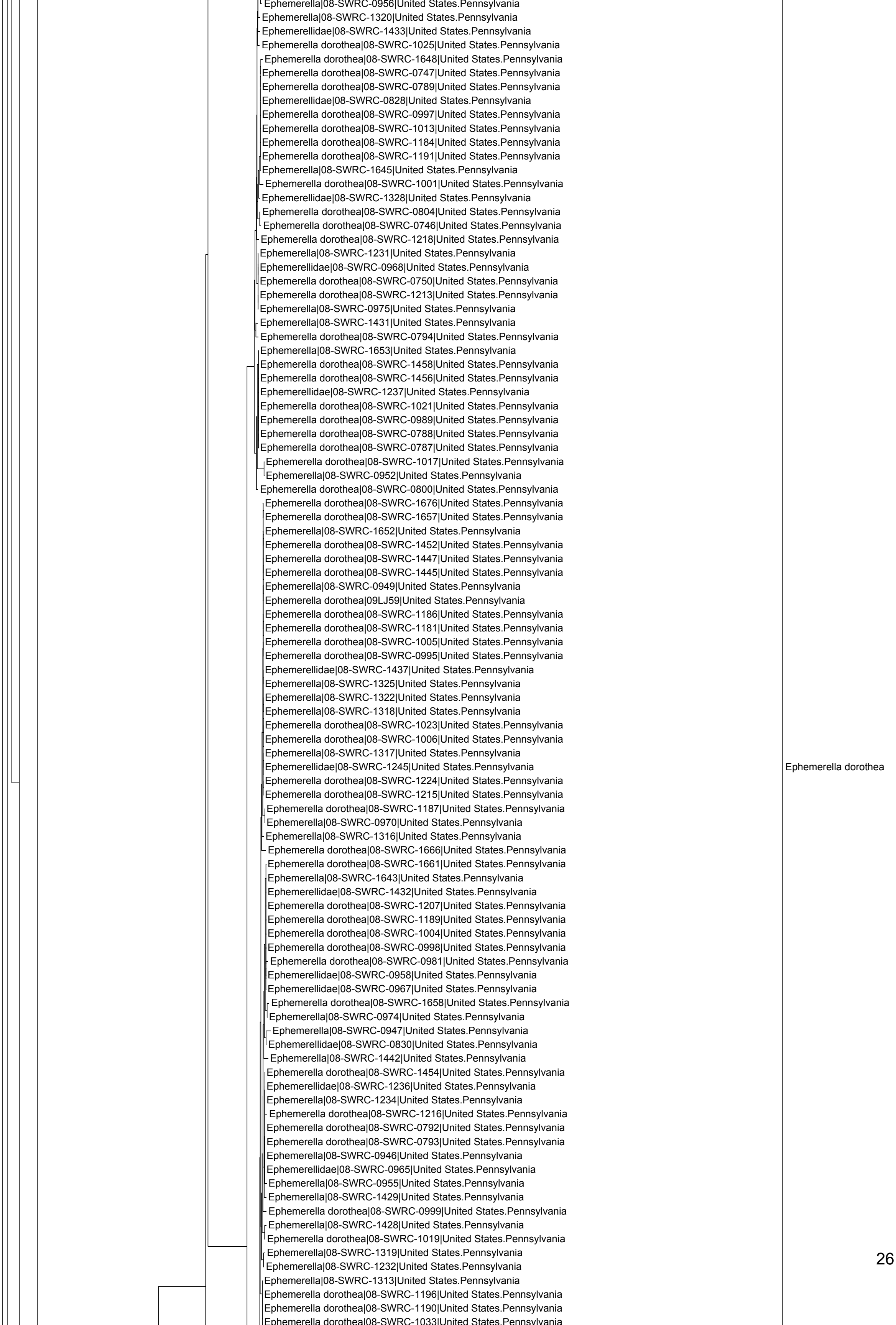

Ephemerella|08-SWRC-0956|United States.Pennsylvania
Ephemerella|08-SWRC-1320|United States.Pennsylvania
Ephemerellidae|08-SWRC-1433|United States.Pennsylvania
Ephemerella dorothea|08-SWRC-1025|United States.Pennsylvania
Ephemerella dorothea|08-SWRC-1648|United States.Pennsylvania
Ephemerella dorothea|08-SWRC-0747|United States.Pennsylvania
Ephemerella dorothea|08-SWRC-0789|United States.Pennsylvania
Ephemerellidae|08-SWRC-0828|United States.Pennsylvania
Ephemerella dorothea|08-SWRC-0997|United States.Pennsylvania
Ephemerella dorothea|08-SWRC-1013|United States.Pennsylvania
Ephemerella dorothea|08-SWRC-1184|United States.Pennsylvania
Ephemerella dorothea|08-SWRC-1191|United States.Pennsylvania
Ephemerella|08-SWRC-1645|United States.Pennsylvania
Ephemerella dorothea|08-SWRC-1001|United States.Pennsylvania
Ephemerellidae|08-SWRC-1328|United States.Pennsylvania
Ephemerella dorothea|08-SWRC-0804|United States.Pennsylvania
Ephemerella dorothea|08-SWRC-0746|United States.Pennsylvania
Ephemerella dorothea|08-SWRC-1218|United States.Pennsylvania
Ephemerella|08-SWRC-1231|United States.Pennsylvania
Ephemerellidae|08-SWRC-0968|United States.Pennsylvania
Ephemerella dorothea|08-SWRC-0750|United States.Pennsylvania
Ephemerella dorothea|08-SWRC-1213|United States.Pennsylvania
Ephemerella|08-SWRC-0975|United States.Pennsylvania
Ephemerella|08-SWRC-1431|United States.Pennsylvania
Ephemerella dorothea|08-SWRC-0794|United States.Pennsylvania
Ephemerella|08-SWRC-1653|United States.Pennsylvania
Ephemerella dorothea|08-SWRC-1458|United States.Pennsylvania
Ephemerella dorothea|08-SWRC-1456|United States.Pennsylvania
Ephemerellidae|08-SWRC-1237|United States.Pennsylvania
Ephemerella dorothea|08-SWRC-1021|United States.Pennsylvania
Ephemerella dorothea|08-SWRC-0989|United States.Pennsylvania
Ephemerella dorothea|08-SWRC-0788|United States.Pennsylvania
Ephemerella dorothea|08-SWRC-0787|United States.Pennsylvania
Ephemerella dorothea|08-SWRC-1017|United States.Pennsylvania
Ephemerella|08-SWRC-0952|United States.Pennsylvania
Ephemerella dorothea|08-SWRC-0800|United States.Pennsylvania
Ephemerella dorothea|08-SWRC-1676|United States.Pennsylvania
Ephemerella dorothea|08-SWRC-1657|United States.Pennsylvania
Ephemerella|08-SWRC-1652|United States.Pennsylvania
Ephemerella dorothea|08-SWRC-1452|United States.Pennsylvania
Ephemerella dorothea|08-SWRC-1447|United States.Pennsylvania
Ephemerella dorothea|08-SWRC-1445|United States.Pennsylvania
Ephemerella|08-SWRC-0949|United States.Pennsylvania
Ephemerella dorothea|09LJ59|United States.Pennsylvania
Ephemerella dorothea|08-SWRC-1186|United States.Pennsylvania
Ephemerella dorothea|08-SWRC-1181|United States.Pennsylvania
Ephemerella dorothea|08-SWRC-1005|United States.Pennsylvania
Ephemerella dorothea|08-SWRC-0995|United States.Pennsylvania
Ephemerellidae|08-SWRC-1437|United States.Pennsylvania
Ephemerella|08-SWRC-1325|United States.Pennsylvania
Ephemerella|08-SWRC-1322|United States.Pennsylvania
Ephemerella|08-SWRC-1318|United States.Pennsylvania
Ephemerella dorothea|08-SWRC-1023|United States.Pennsylvania
Ephemerella dorothea|08-SWRC-1006|United States.Pennsylvania
Ephemerella|08-SWRC-1317|United States.Pennsylvania
Ephemerellidae|08-SWRC-1245|United States.Pennsylvania
Ephemerella dorothea|08-SWRC-1224|United States.Pennsylvania
Ephemerella dorothea|08-SWRC-1215|United States.Pennsylvania
Ephemerella dorothea|08-SWRC-1187|United States.Pennsylvania
Ephemerella|08-SWRC-0970|United States.Pennsylvania
Ephemerella|08-SWRC-1316|United States.Pennsylvania
Ephemerella dorothea|08-SWRC-1666|United States.Pennsylvania
Ephemerella dorothea|08-SWRC-1661|United States.Pennsylvania
Ephemerella|08-SWRC-1643|United States.Pennsylvania
Ephemerellidae|08-SWRC-1432|United States.Pennsylvania
Ephemerella dorothea|08-SWRC-1207|United States.Pennsylvania
Ephemerella dorothea|08-SWRC-1189|United States.Pennsylvania
Ephemerella dorothea|08-SWRC-1004|United States.Pennsylvania
Ephemerella dorothea|08-SWRC-0998|United States.Pennsylvania
Ephemerella dorothea|08-SWRC-0981|United States.Pennsylvania
Ephemerellidae|08-SWRC-0958|United States.Pennsylvania
Ephemerellidae|08-SWRC-0967|United States.Pennsylvania
Ephemerella dorothea|08-SWRC-1658|United States.Pennsylvania
Ephemerella|08-SWRC-0974|United States.Pennsylvania
Ephemerella|08-SWRC-0947|United States.Pennsylvania
Ephemerellidae|08-SWRC-0830|United States.Pennsylvania
Ephemerella|08-SWRC-1442|United States.Pennsylvania
Ephemerella dorothea|08-SWRC-1454|United States.Pennsylvania
Ephemerellidae|08-SWRC-1236|United States.Pennsylvania
Ephemerella|08-SWRC-1234|United States.Pennsylvania
Ephemerella dorothea|08-SWRC-1216|United States.Pennsylvania
Ephemerella dorothea|08-SWRC-0792|United States.Pennsylvania
Ephemerella dorothea|08-SWRC-0793|United States.Pennsylvania
Ephemerella|08-SWRC-0946|United States.Pennsylvania
Ephemerellidae|08-SWRC-0965|United States.Pennsylvania
Ephemerella|08-SWRC-0955|United States.Pennsylvania
Ephemerella|08-SWRC-1429|United States.Pennsylvania
Ephemerella dorothea|08-SWRC-0999|United States.Pennsylvania
Ephemerella|08-SWRC-1428|United States.Pennsylvania
Ephemerella dorothea|08-SWRC-1019|United States.Pennsylvania
Ephemerella|08-SWRC-1319|United States.Pennsylvania
Ephemerella|08-SWRC-1232|United States.Pennsylvania
Ephemerella|08-SWRC-1313|United States.Pennsylvania
Ephemerella dorothea|08-SWRC-1196|United States.Pennsylvania
Ephemerella dorothea|08-SWRC-1190|United States.Pennsylvania
Ephemerella dorothea|08-SWRC-1033|United States.Pennsylvania
Ephemerella dorothea

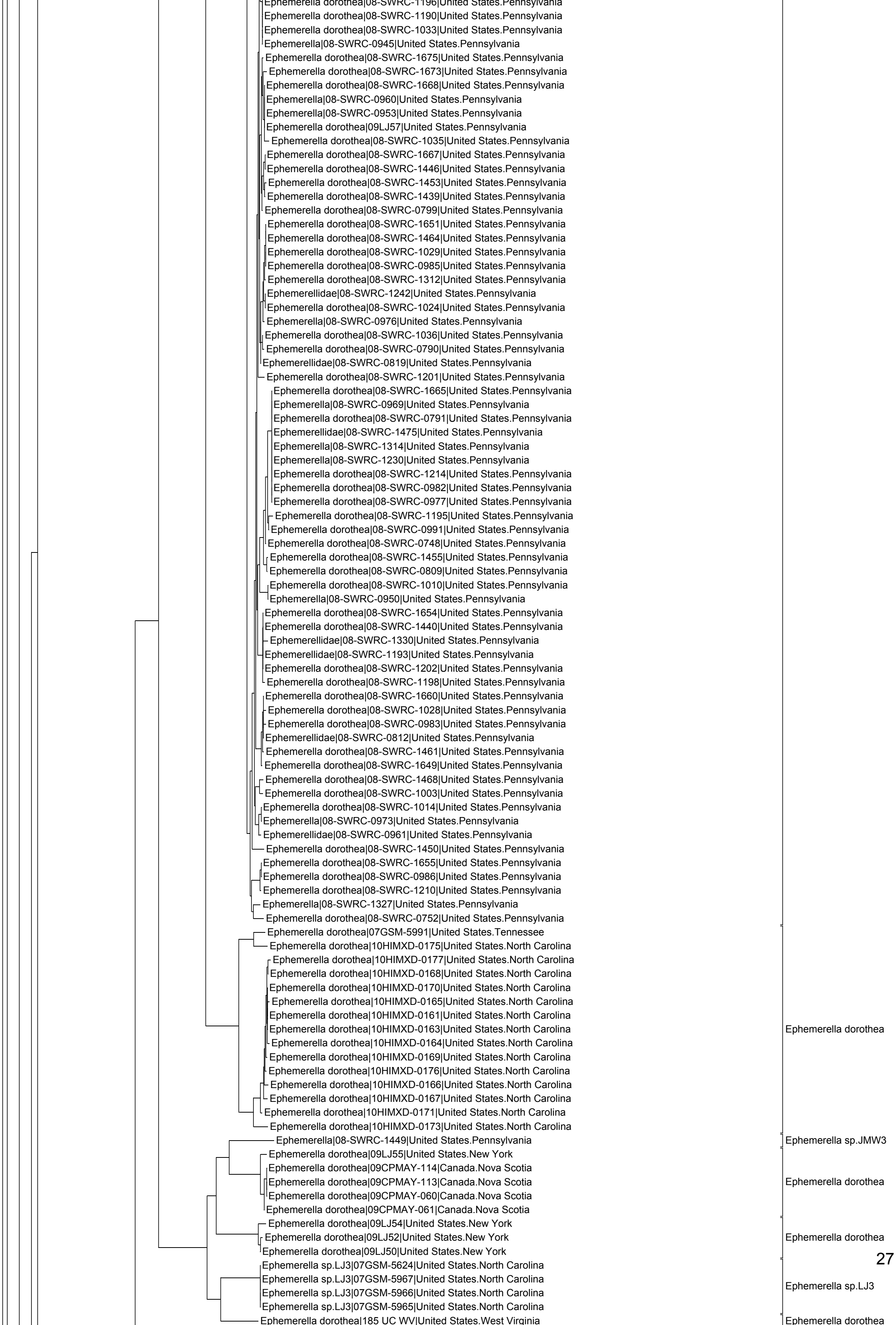

Ephemerella dorothea|08-SWRC-1196|United States.Pennsylvania
Ephemerella dorothea|08-SWRC-1190|United States.Pennsylvania
Ephemerella dorothea|08-SWRC-1033|United States.Pennsylvania
Ephemerella|08-SWRC-0945|United States.Pennsylvania
Ephemerella dorothea|08-SWRC-1675|United States.Pennsylvania
Ephemerella dorothea|08-SWRC-1673|United States.Pennsylvania
Ephemerella dorothea|08-SWRC-1668|United States.Pennsylvania
Ephemerella|08-SWRC-0960|United States.Pennsylvania
Ephemerella|08-SWRC-0953|United States.Pennsylvania
Ephemerella dorothea|09LJ57|United States.Pennsylvania
Ephemerella dorothea|08-SWRC-1035|United States.Pennsylvania
Ephemerella dorothea|08-SWRC-1667|United States.Pennsylvania
Ephemerella dorothea|08-SWRC-1446|United States.Pennsylvania
Ephemerella dorothea|08-SWRC-1453|United States.Pennsylvania
Ephemerella dorothea|08-SWRC-1439|United States.Pennsylvania
Ephemerella dorothea|08-SWRC-0799|United States.Pennsylvania
Ephemerella dorothea|08-SWRC-1651|United States.Pennsylvania
Ephemerella dorothea|08-SWRC-1464|United States.Pennsylvania
Ephemerella dorothea|08-SWRC-1029|United States.Pennsylvania
Ephemerella dorothea|08-SWRC-0985|United States.Pennsylvania
Ephemerella dorothea|08-SWRC-1312|United States.Pennsylvania
Ephemerellidae|08-SWRC-1242|United States.Pennsylvania
Ephemerella dorothea|08-SWRC-1024|United States.Pennsylvania
Ephemerella|08-SWRC-0976|United States.Pennsylvania
Ephemerella dorothea|08-SWRC-1036|United States.Pennsylvania
Ephemerella dorothea|08-SWRC-0790|United States.Pennsylvania
Ephemerellidae|08-SWRC-0819|United States.Pennsylvania
Ephemerella dorothea|08-SWRC-1201|United States.Pennsylvania
Ephemerella dorothea|08-SWRC-1665|United States.Pennsylvania
Ephemerella|08-SWRC-0969|United States.Pennsylvania
Ephemerella dorothea|08-SWRC-0791|United States.Pennsylvania
Ephemerellidae|08-SWRC-1475|United States.Pennsylvania
Ephemerella|08-SWRC-1314|United States.Pennsylvania
Ephemerella|08-SWRC-1230|United States.Pennsylvania
Ephemerella dorothea|08-SWRC-1214|United States.Pennsylvania
Ephemerella dorothea|08-SWRC-0982|United States.Pennsylvania
Ephemerella dorothea|08-SWRC-0977|United States.Pennsylvania
Ephemerella dorothea|08-SWRC-1195|United States.Pennsylvania
Ephemerella dorothea|08-SWRC-0991|United States.Pennsylvania
Ephemerella dorothea|08-SWRC-0748|United States.Pennsylvania
Ephemerella dorothea|08-SWRC-1455|United States.Pennsylvania
Ephemerella dorothea|08-SWRC-0809|United States.Pennsylvania
Ephemerella dorothea|08-SWRC-1010|United States.Pennsylvania
Ephemerella|08-SWRC-0950|United States.Pennsylvania
Ephemerella dorothea|08-SWRC-1654|United States.Pennsylvania
Ephemerella dorothea|08-SWRC-1440|United States.Pennsylvania
Ephemerellidae|08-SWRC-1330|United States.Pennsylvania
Ephemerellidae|08-SWRC-1193|United States.Pennsylvania
Ephemerella dorothea|08-SWRC-1202|United States.Pennsylvania
Ephemerella dorothea|08-SWRC-1198|United States.Pennsylvania
Ephemerella dorothea|08-SWRC-1660|United States.Pennsylvania
Ephemerella dorothea|08-SWRC-1028|United States.Pennsylvania
Ephemerella dorothea|08-SWRC-0983|United States.Pennsylvania
Ephemerellidae|08-SWRC-0812|United States.Pennsylvania
Ephemerella dorothea|08-SWRC-1461|United States.Pennsylvania
Ephemerella dorothea|08-SWRC-1649|United States.Pennsylvania
Ephemerella dorothea|08-SWRC-1468|United States.Pennsylvania
Ephemerella dorothea|08-SWRC-1003|United States.Pennsylvania
Ephemerella dorothea|08-SWRC-1014|United States.Pennsylvania
Ephemerella|08-SWRC-0973|United States.Pennsylvania
Ephemerellidae|08-SWRC-0961|United States.Pennsylvania
Ephemerella dorothea|08-SWRC-1450|United States.Pennsylvania
Ephemerella dorothea|08-SWRC-1655|United States.Pennsylvania
Ephemerella dorothea|08-SWRC-0986|United States.Pennsylvania
Ephemerella dorothea|08-SWRC-1210|United States.Pennsylvania
Ephemerella|08-SWRC-1327|United States.Pennsylvania
Ephemerella dorothea|08-SWRC-0752|United States.Pennsylvania
Ephemerella dorothea|07GSM-5991|United States.Tennessee
Ephemerella dorothea|10HIMXD-0175|United States.North Carolina
Ephemerella dorothea|10HIMXD-0177|United States.North Carolina
Ephemerella dorothea|10HIMXD-0168|United States.North Carolina
Ephemerella dorothea|10HIMXD-0170|United States.North Carolina
Ephemerella dorothea|10HIMXD-0165|United States.North Carolina
Ephemerella dorothea|10HIMXD-0161|United States.North Carolina
Ephemerella dorothea|10HIMXD-0163|United States.North Carolina
Ephemerella dorothea|10HIMXD-0164|United States.North Carolina
Ephemerella dorothea|10HIMXD-0169|United States.North Carolina
Ephemerella dorothea|10HIMXD-0176|United States.North Carolina
Ephemerella dorothea|10HIMXD-0166|United States.North Carolina
Ephemerella dorothea|10HIMXD-0167|United States.North Carolina
Ephemerella dorothea|10HIMXD-0171|United States.North Carolina
Ephemerella dorothea|10HIMXD-0173|United States.North Carolina
Ephemerella dorothea
Ephemerella|08-SWRC-1449|United States.Pennsylvania
Ephemerella sp.JMW3
Ephemerella dorothea|09LJ55|United States.New York
Ephemerella dorothea|09CPMAY-114|Canada.Nova Scotia
Ephemerella dorothea|09CPMAY-113|Canada.Nova Scotia
Ephemerella dorothea|09CPMAY-060|Canada.Nova Scotia
Ephemerella dorothea|09CPMAY-061|Canada.Nova Scotia
Ephemerella dorothea
Ephemerella dorothea|09LJ54|United States.New York
Ephemerella dorothea|09LJ52|United States.New York
Ephemerella dorothea|09LJ50|United States.New York
Ephemerella dorothea
Ephemerella sp.LJ3|07GSM-5624|United States.North Carolina
Ephemerella sp.LJ3|07GSM-5967|United States.North Carolina
Ephemerella sp.LJ3|07GSM-5966|United States.North Carolina
Ephemerella sp.LJ3|07GSM-5965|United States.North Carolina
Ephemerella sp.LJ3
Ephemerella dorothea|185 UC WV|United States.West Virginia
Ephemerella dorothea

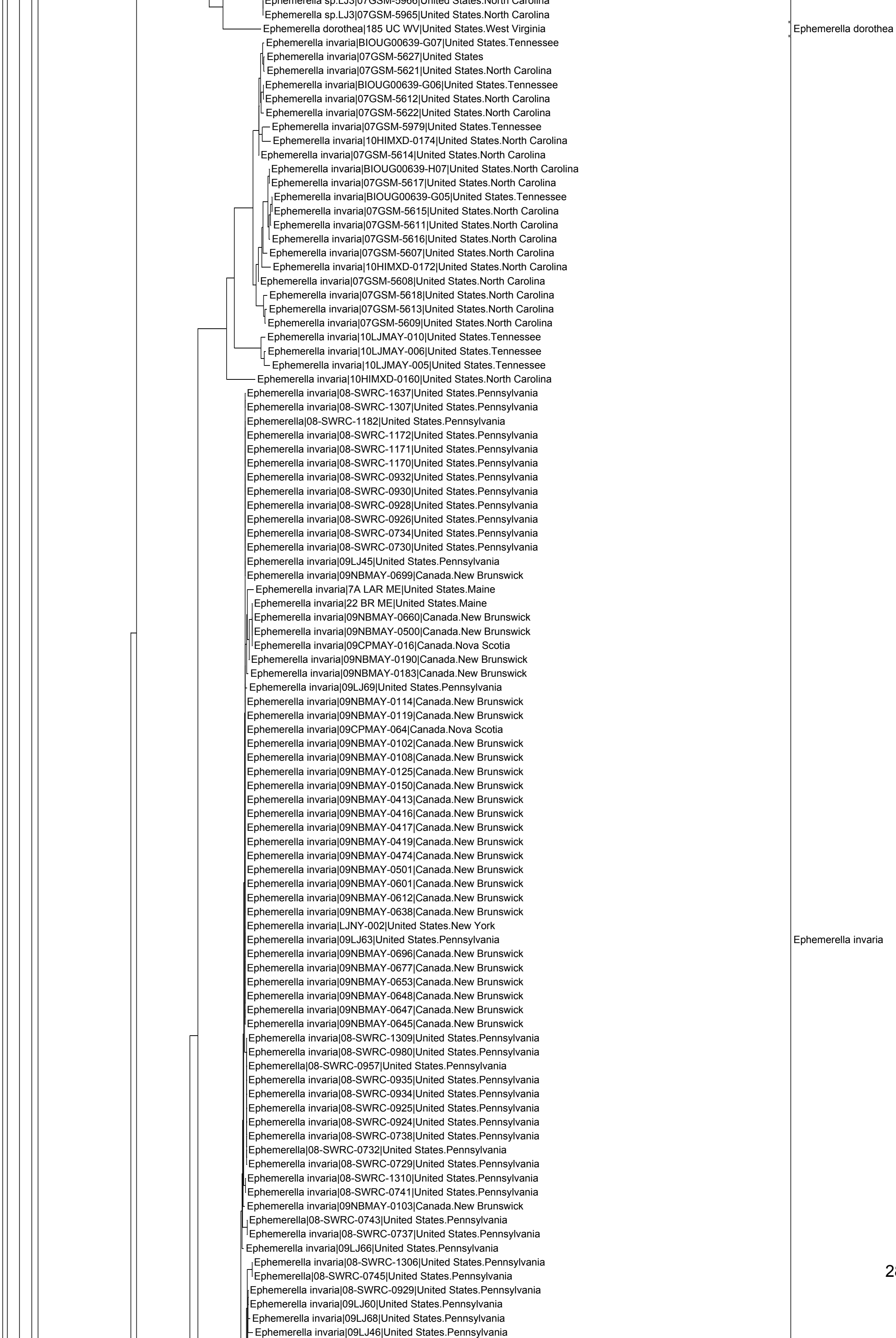
Ephemerella sp.LJ3|07GSM-5965|United States.North Carolina
Ephemerella dorothea|185 UC WV|United States.West Virginia
Ephemerella invaria|BIOUG00639-G07|United States.Tennessee
Ephemerella invaria|07GSM-5627|United States
Ephemerella invaria|07GSM-5621|United States.North Carolina
Ephemerella invaria|BIOUG00639-G06|United States.Tennessee
Ephemerella invaria|07GSM-5612|United States.North Carolina
Ephemerella invaria|07GSM-5622|United States.North Carolina
Ephemerella invaria|07GSM-5979|United States.Tennessee
Ephemerella invaria|10HIMXD-0174|United States.North Carolina
Ephemerella invaria|07GSM-5614|United States.North Carolina
Ephemerella invaria|BIOUG00639-H07|United States.North Carolina
Ephemerella invaria|07GSM-5617|United States.North Carolina
Ephemerella invaria|BIOUG00639-G05|United States.Tennessee
Ephemerella invaria|07GSM-5615|United States.North Carolina
Ephemerella invaria|07GSM-5611|United States.North Carolina
Ephemerella invaria|07GSM-5616|United States.North Carolina
Ephemerella invaria|07GSM-5607|United States.North Carolina
Ephemerella invaria|10HIMXD-0172|United States.North Carolina
Ephemerella invaria|07GSM-5608|United States.North Carolina
Ephemerella invaria|07GSM-5618|United States.North Carolina
Ephemerella invaria|07GSM-5613|United States.North Carolina
Ephemerella invaria|07GSM-5609|United States.North Carolina
Ephemerella invaria|10LJMAY-010|United States.Tennessee
Ephemerella invaria|10LJMAY-006|United States.Tennessee
Ephemerella invaria|10LJMAY-005|United States.Tennessee
Ephemerella invaria|10HIMXD-0160|United States.North Carolina
Ephemerella invaria|08-SWRC-1637|United States.Pennsylvania
Ephemerella invaria|08-SWRC-1307|United States.Pennsylvania
Ephemerella|08-SWRC-1182|United States.Pennsylvania
Ephemerella invaria|08-SWRC-1172|United States.Pennsylvania
Ephemerella invaria|08-SWRC-1171|United States.Pennsylvania
Ephemerella invaria|08-SWRC-1170|United States.Pennsylvania
Ephemerella invaria|08-SWRC-0932|United States.Pennsylvania
Ephemerella invaria|08-SWRC-0930|United States.Pennsylvania
Ephemerella invaria|08-SWRC-0928|United States.Pennsylvania
Ephemerella invaria|08-SWRC-0926|United States.Pennsylvania
Ephemerella invaria|08-SWRC-0734|United States.Pennsylvania
Ephemerella invaria|08-SWRC-0730|United States.Pennsylvania
Ephemerella invaria|09LJ45|United States.Pennsylvania
Ephemerella invaria|09NBMAY-0699|Canada.New Brunswick
Ephemerella invaria|7A LAR ME|United States.Maine
Ephemerella invaria|22 BR ME|United States.Maine
Ephemerella invaria|09NBMAY-0660|Canada.New Brunswick
Ephemerella invaria|09NBMAY-0500|Canada.New Brunswick
Ephemerella invaria|09CPMAY-016|Canada.Nova Scotia
Ephemerella invaria|09NBMAY-0190|Canada.New Brunswick
Ephemerella invaria|09NBMAY-0183|Canada.New Brunswick
Ephemerella invaria|09LJ69|United States.Pennsylvania
Ephemerella invaria|09NBMAY-0114|Canada.New Brunswick
Ephemerella invaria|09NBMAY-0119|Canada.New Brunswick
Ephemerella invaria|09CPMAY-064|Canada.Nova Scotia
Ephemerella invaria|09NBMAY-0102|Canada.New Brunswick
Ephemerella invaria|09NBMAY-0108|Canada.New Brunswick
Ephemerella invaria|09NBMAY-0125|Canada.New Brunswick
Ephemerella invaria|09NBMAY-0150|Canada.New Brunswick
Ephemerella invaria|09NBMAY-0413|Canada.New Brunswick
Ephemerella invaria|09NBMAY-0416|Canada.New Brunswick
Ephemerella invaria|09NBMAY-0417|Canada.New Brunswick
Ephemerella invaria|09NBMAY-0419|Canada.New Brunswick
Ephemerella invaria|09NBMAY-0474|Canada.New Brunswick
Ephemerella invaria|09NBMAY-0501|Canada.New Brunswick
Ephemerella invaria|09NBMAY-0601|Canada.New Brunswick
Ephemerella invaria|09NBMAY-0612|Canada.New Brunswick
Ephemerella invaria|09NBMAY-0638|Canada.New Brunswick
Ephemerella invaria|LJNY-002|United States.New York
Ephemerella invaria|09LJ63|United States.Pennsylvania
Ephemerella invaria|09NBMAY-0696|Canada.New Brunswick
Ephemerella invaria|09NBMAY-0677|Canada.New Brunswick
Ephemerella invaria|09NBMAY-0653|Canada.New Brunswick
Ephemerella invaria|09NBMAY-0648|Canada.New Brunswick
Ephemerella invaria|09NBMAY-0647|Canada.New Brunswick
Ephemerella invaria|09NBMAY-0645|Canada.New Brunswick
Ephemerella invaria|08-SWRC-1309|United States.Pennsylvania
Ephemerella invaria|08-SWRC-0980|United States.Pennsylvania
Ephemerella|08-SWRC-0957|United States.Pennsylvania
Ephemerella invaria|08-SWRC-0935|United States.Pennsylvania
Ephemerella invaria|08-SWRC-0934|United States.Pennsylvania
Ephemerella invaria|08-SWRC-0925|United States.Pennsylvania
Ephemerella invaria|08-SWRC-0924|United States.Pennsylvania
Ephemerella invaria|08-SWRC-0738|United States.Pennsylvania
Ephemerella|08-SWRC-0732|United States.Pennsylvania
Ephemerella invaria|08-SWRC-0729|United States.Pennsylvania
Ephemerella invaria|08-SWRC-1310|United States.Pennsylvania
Ephemerella invaria|08-SWRC-0741|United States.Pennsylvania
Ephemerella invaria|09NBMAY-0103|Canada.New Brunswick
Ephemerella|08-SWRC-0743|United States.Pennsylvania
Ephemerella invaria|08-SWRC-0737|United States.Pennsylvania
Ephemerella invaria|09LJ66|United States.Pennsylvania
Ephemerella invaria|08-SWRC-1306|United States.Pennsylvania
Ephemerella|08-SWRC-0745|United States.Pennsylvania
Ephemerella invaria|08-SWRC-0929|United States.Pennsylvania
Ephemerella invaria|09LJ60|United States.Pennsylvania
Ephemerella invaria|09LJ68|United States.Pennsylvania
Ephemerella invaria|09LJ46|United States.Pennsylvania
Ephemerella dorothea
Ephemerella invaria

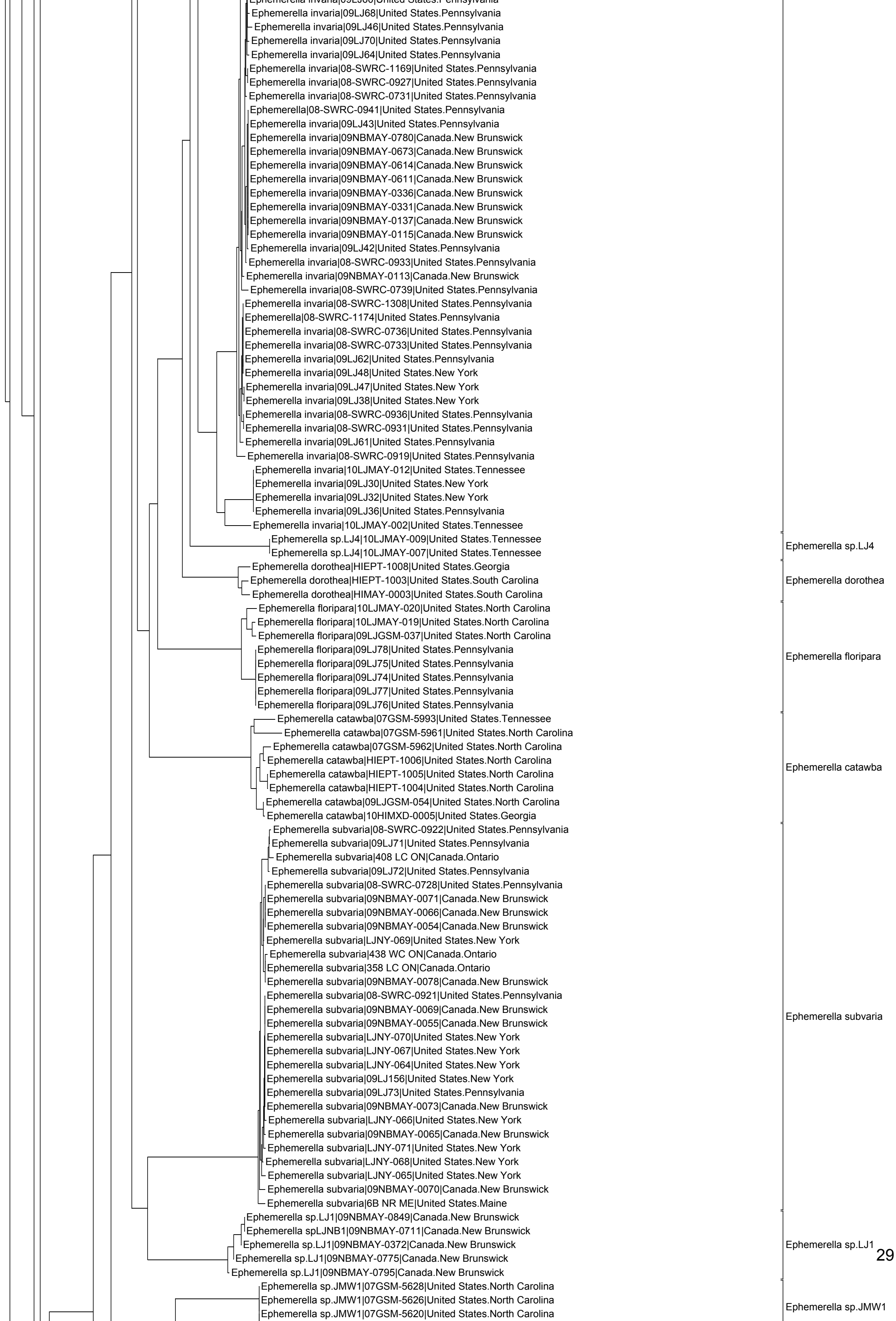

Ephemerella invaria|09LJ68|United States.Pennsylvania
Ephemerella invaria|09LJ46|United States.Pennsylvania
Ephemerella invaria|09LJ70|United States.Pennsylvania
Ephemerella invaria|09LJ64|United States.Pennsylvania
Ephemerella invaria|08-SWRC-1169|United States.Pennsylvania
Ephemerella invaria|08-SWRC-0927|United States.Pennsylvania
Ephemerella invaria|08-SWRC-0731|United States.Pennsylvania
Ephemerella|08-SWRC-0941|United States.Pennsylvania
Ephemerella invaria|09LJ43|United States.Pennsylvania
Ephemerella invaria|09NBMAY-0780|Canada.New Brunswick
Ephemerella invaria|09NBMAY-0673|Canada.New Brunswick
Ephemerella invaria|09NBMAY-0614|Canada.New Brunswick
Ephemerella invaria|09NBMAY-0611|Canada.New Brunswick
Ephemerella invaria|09NBMAY-0336|Canada.New Brunswick
Ephemerella invaria|09NBMAY-0331|Canada.New Brunswick
Ephemerella invaria|09NBMAY-0137|Canada.New Brunswick
Ephemerella invaria|09NBMAY-0115|Canada.New Brunswick
Ephemerella invaria|09LJ42|United States.Pennsylvania
Ephemerella invaria|08-SWRC-0933|United States.Pennsylvania
Ephemerella invaria|09NBMAY-0113|Canada.New Brunswick
Ephemerella invaria|08-SWRC-0739|United States.Pennsylvania
Ephemerella invaria|08-SWRC-1308|United States.Pennsylvania
Ephemerella|08-SWRC-1174|United States.Pennsylvania
Ephemerella invaria|08-SWRC-0736|United States.Pennsylvania
Ephemerella invaria|08-SWRC-0733|United States.Pennsylvania
Ephemerella invaria|09LJ62|United States.Pennsylvania
Ephemerella invaria|09LJ48|United States.New York
Ephemerella invaria|09LJ47|United States.New York
Ephemerella invaria|09LJ38|United States.New York
Ephemerella invaria|08-SWRC-0936|United States.Pennsylvania
Ephemerella invaria|08-SWRC-0931|United States.Pennsylvania
Ephemerella invaria|09LJ61|United States.Pennsylvania
Ephemerella invaria|08-SWRC-0919|United States.Pennsylvania
Ephemerella invaria|10LJMAY-012|United States.Tennessee
Ephemerella invaria|09LJ30|United States.New York
Ephemerella invaria|09LJ32|United States.New York
Ephemerella invaria|09LJ36|United States.Pennsylvania
Ephemerella invaria|10LJMAY-002|United States.Tennessee
Ephemerella sp.LJ4|10LJMAY-009|United States.Tennessee
Ephemerella sp.LJ4|10LJMAY-007|United States.Tennessee
Ephemerella sp.LJ4
Ephemerella dorothea|HIEPT-1008|United States.Georgia
Ephemerella dorothea|HIEPT-1003|United States.South Carolina
Ephemerella dorothea|HIMAY-0003|United States.South Carolina
Ephemerella dorothea
Ephemerella floripara|10LJMAY-020|United States.North Carolina
Ephemerella floripara|10LJMAY-019|United States.North Carolina
Ephemerella floripara|09LJGSM-037|United States.North Carolina
Ephemerella floripara|09LJ78|United States.Pennsylvania
Ephemerella floripara|09LJ75|United States.Pennsylvania
Ephemerella floripara|09LJ74|United States.Pennsylvania
Ephemerella floripara|09LJ77|United States.Pennsylvania
Ephemerella floripara|09LJ76|United States.Pennsylvania
Ephemerella floripara
Ephemerella catawba|07GSM-5993|United States.Tennessee
Ephemerella catawba|07GSM-5961|United States.North Carolina
Ephemerella catawba|07GSM-5962|United States.North Carolina
Ephemerella catawba|HIEPT-1006|United States.North Carolina
Ephemerella catawba|HIEPT-1005|United States.North Carolina
Ephemerella catawba|HIEPT-1004|United States.North Carolina
Ephemerella catawba|09LJGSM-054|United States.North Carolina
Ephemerella catawba|10HIMXD-0005|United States.Georgia
Ephemerella catawba
Ephemerella subvaria|08-SWRC-0922|United States.Pennsylvania
Ephemerella subvaria|09LJ71|United States.Pennsylvania
Ephemerella subvaria|408 LC ON|Canada.Ontario
Ephemerella subvaria|09LJ72|United States.Pennsylvania
Ephemerella subvaria|08-SWRC-0728|United States.Pennsylvania
Ephemerella subvaria|09NBMAY-0071|Canada.New Brunswick
Ephemerella subvaria|09NBMAY-0066|Canada.New Brunswick
Ephemerella subvaria|09NBMAY-0054|Canada.New Brunswick
Ephemerella subvaria|LJNY-069|United States.New York
Ephemerella subvaria|438 WC ON|Canada.Ontario
Ephemerella subvaria|358 LC ON|Canada.Ontario
Ephemerella subvaria|09NBMAY-0078|Canada.New Brunswick
Ephemerella subvaria|08-SWRC-0921|United States.Pennsylvania
Ephemerella subvaria|09NBMAY-0069|Canada.New Brunswick
Ephemerella subvaria|09NBMAY-0055|Canada.New Brunswick
Ephemerella subvaria|LJNY-070|United States.New York
Ephemerella subvaria|LJNY-067|United States.New York
Ephemerella subvaria|LJNY-064|United States.New York
Ephemerella subvaria|09LJ156|United States.New York
Ephemerella subvaria|09LJ73|United States.Pennsylvania
Ephemerella subvaria|09NBMAY-0073|Canada.New Brunswick
Ephemerella subvaria|LJNY-066|United States.New York
Ephemerella subvaria|09NBMAY-0065|Canada.New Brunswick
Ephemerella subvaria|LJNY-071|United States.New York
Ephemerella subvaria|LJNY-068|United States.New York
Ephemerella subvaria|LJNY-065|United States.New York
Ephemerella subvaria|09NBMAY-0070|Canada.New Brunswick
Ephemerella subvaria|6B NR ME|United States.Maine
Ephemerella subvaria
Ephemerella sp.LJ1|09NBMAY-0849|Canada.New Brunswick
Ephemerella spLJNB1|09NBMAY-0711|Canada.New Brunswick
Ephemerella sp.LJ1|09NBMAY-0372|Canada.New Brunswick
Ephemerella sp.LJ1|09NBMAY-0775|Canada.New Brunswick
Ephemerella sp.LJ1|09NBMAY-0795|Canada.New Brunswick
Ephemerella sp.LJ1
Ephemerella sp.JMW1|07GSM-5628|United States.North Carolina
Ephemerella sp.JMW1|07GSM-5626|United States.North Carolina
Ephemerella sp.JMW1|07GSM-5620|United States.North Carolina
Ephemerella sp.JMW1

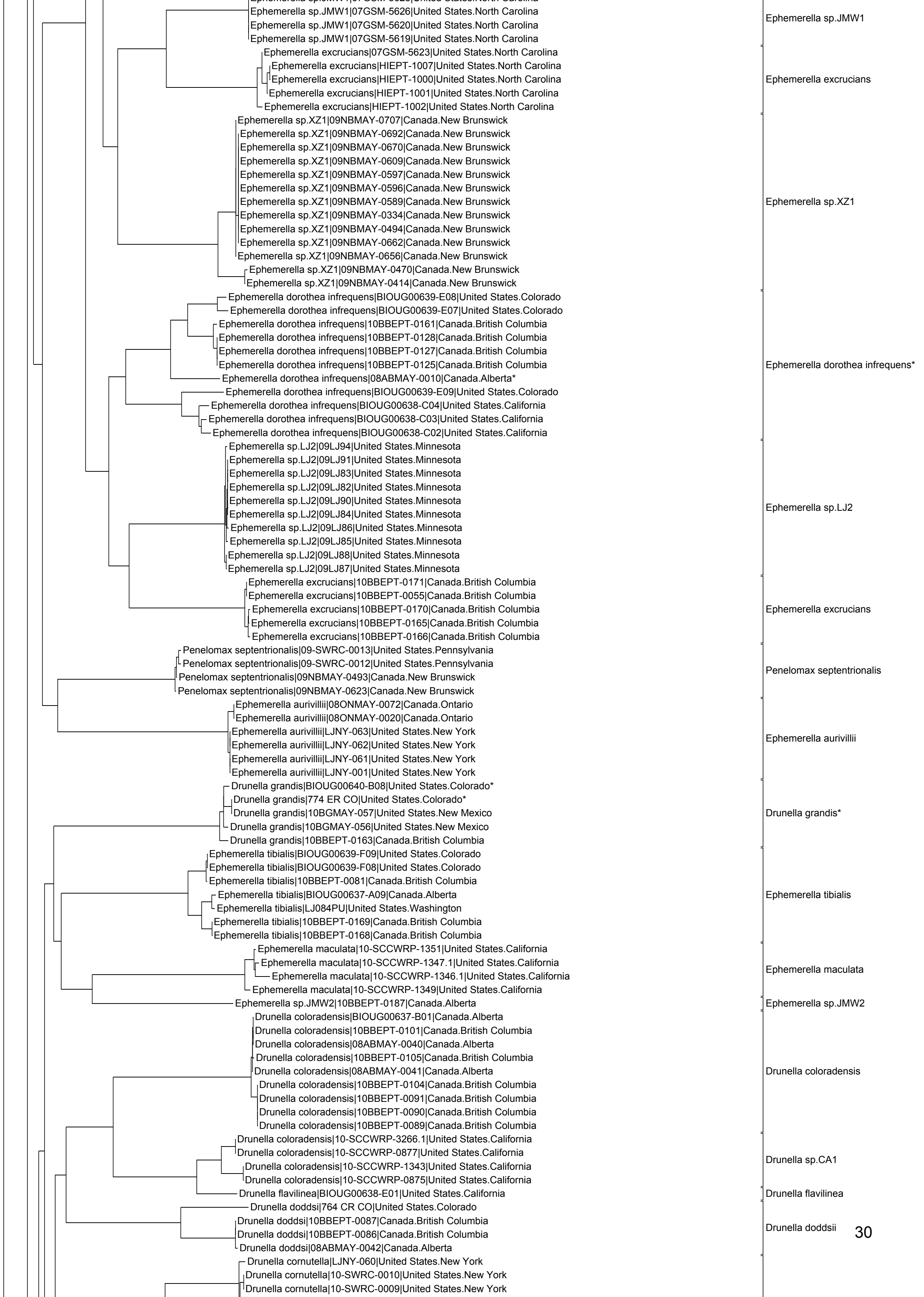
Ephemerella sp.JMW1|07GSM-5626|United States.North Carolina
Ephemerella sp.JMW1|07GSM-5620|United States.North Carolina
Ephemerella sp.JMW1|07GSM-5619|United States.North Carolina
Ephemerella excrucians|07GSM-5623|United States.North Carolina
Ephemerella excrucians|HIEPT-1007|United States.North Carolina
Ephemerella excrucians|HIEPT-1000|United States.North Carolina
Ephemerella excrucians|HIEPT-1001|United States.North Carolina
Ephemerella excrucians|HIEPT-1002|United States.North Carolina
Ephemerella sp.XZ1|09NBMAY-0707|Canada.New Brunswick
Ephemerella sp.XZ1|09NBMAY-0692|Canada.New Brunswick
Ephemerella sp.XZ1|09NBMAY-0670|Canada.New Brunswick
Ephemerella sp.XZ1|09NBMAY-0609|Canada.New Brunswick
Ephemerella sp.XZ1|09NBMAY-0597|Canada.New Brunswick
Ephemerella sp.XZ1|09NBMAY-0596|Canada.New Brunswick
Ephemerella sp.XZ1|09NBMAY-0589|Canada.New Brunswick
Ephemerella sp.XZ1|09NBMAY-0334|Canada.New Brunswick
Ephemerella sp.XZ1|09NBMAY-0494|Canada.New Brunswick
Ephemerella sp.XZ1|09NBMAY-0662|Canada.New Brunswick
Ephemerella sp.XZ1|09NBMAY-0656|Canada.New Brunswick
Ephemerella sp.XZ1|09NBMAY-0470|Canada.New Brunswick
Ephemerella sp.XZ1|09NBMAY-0414|Canada.New Brunswick
Ephemerella dorothea infrequens|BIOUG00639-E08|United States.Colorado
Ephemerella dorothea infrequens|BIOUG00639-E07|United States.Colorado
Ephemerella dorothea infrequens|10BBEPT-0161|Canada.British Columbia
Ephemerella dorothea infrequens|10BBEPT-0128|Canada.British Columbia
Ephemerella dorothea infrequens|10BBEPT-0127|Canada.British Columbia
Ephemerella dorothea infrequens|10BBEPT-0125|Canada.British Columbia
Ephemerella dorothea infrequens|08ABMAY-0010|Canada.Alberta*
Ephemerella dorothea infrequens|BIOUG00639-E09|United States.Colorado
Ephemerella dorothea infrequens|BIOUG00638-C04|United States.California
Ephemerella dorothea infrequens|BIOUG00638-C03|United States.California
Ephemerella dorothea infrequens|BIOUG00638-C02|United States.California
Ephemerella sp.LJ2|09LJ94|United States.Minnesota
Ephemerella sp.LJ2|09LJ91|United States.Minnesota
Ephemerella sp.LJ2|09LJ83|United States.Minnesota
Ephemerella sp.LJ2|09LJ82|United States.Minnesota
Ephemerella sp.LJ2|09LJ90|United States.Minnesota
Ephemerella sp.LJ2|09LJ84|United States.Minnesota
Ephemerella sp.LJ2|09LJ86|United States.Minnesota
Ephemerella sp.LJ2|09LJ85|United States.Minnesota
Ephemerella sp.LJ2|09LJ88|United States.Minnesota
Ephemerella sp.LJ2|09LJ87|United States.Minnesota
Ephemerella excrucians|10BBEPT-0171|Canada.British Columbia
Ephemerella excrucians|10BBEPT-0055|Canada.British Columbia
Ephemerella excrucians|10BBEPT-0170|Canada.British Columbia
Ephemerella excrucians|10BBEPT-0165|Canada.British Columbia
Ephemerella excrucians|10BBEPT-0166|Canada.British Columbia
Penelomax septentrionalis|09-SWRC-0013|United States.Pennsylvania
Penelomax septentrionalis|09-SWRC-0012|United States.Pennsylvania
Penelomax septentrionalis|09NBMAY-0493|Canada.New Brunswick
Penelomax septentrionalis|09NBMAY-0623|Canada.New Brunswick
Ephemerella aurivillii|08ONMAY-0072|Canada.Ontario
Ephemerella aurivillii|08ONMAY-0020|Canada.Ontario
Ephemerella aurivillii|LJNY-063|United States.New York
Ephemerella aurivillii|LJNY-062|United States.New York
Ephemerella aurivillii|LJNY-061|United States.New York
Ephemerella aurivillii|LJNY-001|United States.New York
Drunella grandis|BIOUG00640-B08|United States.Colorado*
Drunella grandis|774 ER CO|United States.Colorado*
Drunella grandis|10BGMAY-057|United States.New Mexico
Drunella grandis|10BGMAY-056|United States.New Mexico
Drunella grandis|10BBEPT-0163|Canada.British Columbia
Ephemerella tibialis|BIOUG00639-F09|United States.Colorado
Ephemerella tibialis|BIOUG00639-F08|United States.Colorado
Ephemerella tibialis|10BBEPT-0081|Canada.British Columbia
Ephemerella tibialis|BIOUG00637-A09|Canada.Alberta
Ephemerella tibialis|LJ084PU|United States.Washington
Ephemerella tibialis|10BBEPT-0169|Canada.British Columbia
Ephemerella tibialis|10BBEPT-0168|Canada.British Columbia
Ephemerella maculata|10-SCCWRP-1351|United States.California
Ephemerella maculata|10-SCCWRP-1347.1|United States.California
Ephemerella maculata|10-SCCWRP-1346.1|United States.California
Ephemerella maculata|10-SCCWRP-1349|United States.California
Ephemerella sp.JMW2|10BBEPT-0187|Canada.Alberta
Drunella coloradensis|BIOUG00637-B01|Canada.Alberta
Drunella coloradensis|10BBEPT-0101|Canada.British Columbia
Drunella coloradensis|08ABMAY-0040|Canada.Alberta
Drunella coloradensis|10BBEPT-0105|Canada.British Columbia
Drunella coloradensis|08ABMAY-0041|Canada.Alberta
Drunella coloradensis|10BBEPT-0104|Canada.British Columbia
Drunella coloradensis|10BBEPT-0091|Canada.British Columbia
Drunella coloradensis|10BBEPT-0090|Canada.British Columbia
Drunella coloradensis|10BBEPT-0089|Canada.British Columbia
Drunella coloradensis|10-SCCWRP-3266.1|United States.California
Drunella coloradensis|10-SCCWRP-0877|United States.California
Drunella coloradensis|10-SCCWRP-1343|United States.California
Drunella coloradensis|10-SCCWRP-0875|United States.California
Drunella flavilinea|BIOUG00638-E01|United States.California
Drunella doddsi|764 CR CO|United States.Colorado
Drunella doddsi|10BBEPT-0087|Canada.British Columbia
Drunella doddsi|10BBEPT-0086|Canada.British Columbia
Drunella doddsi|08ABMAY-0042|Canada.Alberta
Drunella cornutella|LJNY-060|United States.New York
Drunella cornutella|10-SWRC-0010|United States.New York
Drunella cornutella|10-SWRC-0009|United States.New York
Ephemerella sp.JMW1
Ephemerella excrucians
Ephemerella sp.XZ1
Ephemerella dorothea infrequens*
Ephemerella sp.LJ2
Ephemerella excrucians
Penelomax septentrionalis
Ephemerella aurivillii
Drunella grandis*
Ephemerella tibialis
Ephemerella maculata
Ephemerella sp.JMW2
Drunella coloradensis
Drunella sp.CA1
Drunella flavilinea
Drunella doddsii

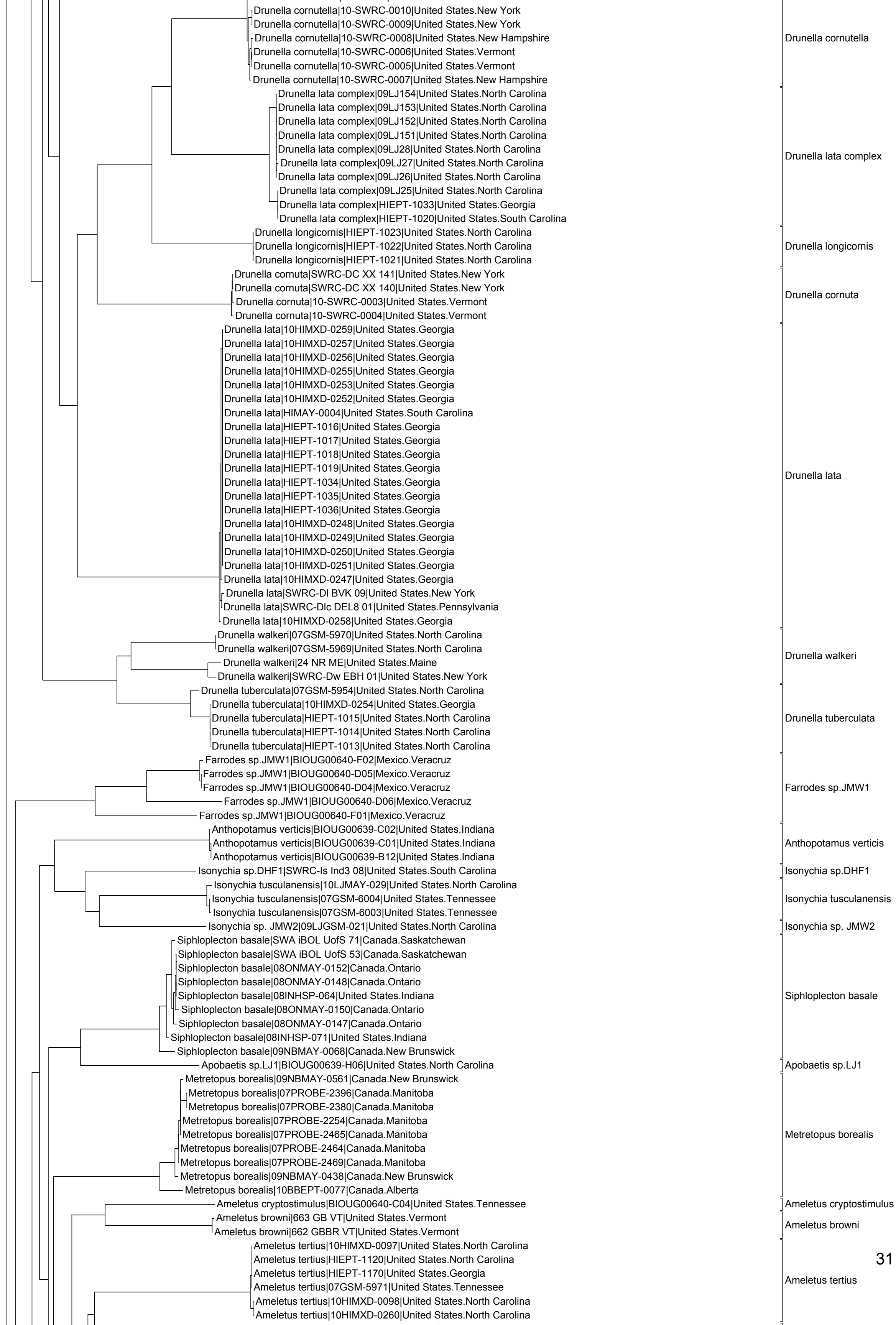
Drunella cornutella|10-SWRC-0010|United States.New York
Drunella cornutella|10-SWRC-0009|United States.New York
Drunella cornutella|10-SWRC-0008|United States.New Hampshire
Drunella cornutella|10-SWRC-0006|United States.Vermont
Drunella cornutella|10-SWRC-0005|United States.Vermont
Drunella cornutella|10-SWRC-0007|United States.New Hampshire
Drunella lata complex|09LJ154|United States.North Carolina
Drunella lata complex|09LJ153|United States.North Carolina
Drunella lata complex|09LJ152|United States.North Carolina
Drunella lata complex|09LJ151|United States.North Carolina
Drunella lata complex|09LJ28|United States.North Carolina
Drunella lata complex|09LJ27|United States.North Carolina
Drunella lata complex|09LJ26|United States.North Carolina
Drunella lata complex|09LJ25|United States.North Carolina
Drunella lata complex|HIEPT-1033|United States.Georgia
Drunella lata complex|HIEPT-1020|United States.South Carolina
Drunella longicornis|HIEPT-1023|United States.North Carolina
Drunella longicornis|HIEPT-1022|United States.North Carolina
Drunella longicornis|HIEPT-1021|United States.North Carolina
Drunella cornuta|SWRC-DC XX 141|United States.New York
Drunella cornuta|SWRC-DC XX 140|United States.New York
Drunella cornuta|10-SWRC-0003|United States.Vermont
Drunella cornuta|10-SWRC-0004|United States.Vermont
Drunella lata|10HIMXD-0259|United States.Georgia
Drunella lata|10HIMXD-0257|United States.Georgia
Drunella lata|10HIMXD-0256|United States.Georgia
Drunella lata|10HIMXD-0255|United States.Georgia
Drunella lata|10HIMXD-0253|United States.Georgia
Drunella lata|10HIMXD-0252|United States.Georgia
Drunella lata|HIMAY-0004|United States.South Carolina
Drunella lata|HIEPT-1016|United States.Georgia
Drunella lata|HIEPT-1017|United States.Georgia
Drunella lata|HIEPT-1018|United States.Georgia
Drunella lata|HIEPT-1019|United States.Georgia
Drunella lata|HIEPT-1034|United States.Georgia
Drunella lata|HIEPT-1035|United States.Georgia
Drunella lata|HIEPT-1036|United States.Georgia
Drunella lata|10HIMXD-0248|United States.Georgia
Drunella lata|10HIMXD-0249|United States.Georgia
Drunella lata|10HIMXD-0250|United States.Georgia
Drunella lata|10HIMXD-0251|United States.Georgia
Drunella lata|10HIMXD-0247|United States.Georgia
Drunella lata|SWRC-Dl BVK 09|United States.New York
Drunella lata|SWRC-Dlc DEL8 01|United States.Pennsylvania
Drunella lata|10HIMXD-0258|United States.Georgia
Drunella walkeri|07GSM-5970|United States.North Carolina
Drunella walkeri|07GSM-5969|United States.North Carolina
Drunella walkeri|24 NR ME|United States.Maine
Drunella walkeri|SWRC-Dw EBH 01|United States.New York
Drunella tuberculata|07GSM-5954|United States.North Carolina
Drunella tuberculata|10HIMXD-0254|United States.Georgia
Drunella tuberculata|HIEPT-1015|United States.North Carolina
Drunella tuberculata|HIEPT-1014|United States.North Carolina
Drunella tuberculata|HIEPT-1013|United States.North Carolina
Farrodes sp.JMW1|BIOUG00640-F02|Mexico.Veracruz
Farrodes sp.JMW1|BIOUG00640-D05|Mexico.Veracruz
Farrodes sp.JMW1|BIOUG00640-D04|Mexico.Veracruz
Farrodes sp.JMW1|BIOUG00640-D06|Mexico.Veracruz
Farrodes sp.JMW1|BIOUG00640-F01|Mexico.Veracruz
Anthopotamus verticis|BIOUG00639-C02|United States.Indiana
Anthopotamus verticis|BIOUG00639-C01|United States.Indiana
Anthopotamus verticis|BIOUG00639-B12|United States.Indiana
Isonychia sp.DHF1|SWRC-Is Ind3 08|United States.South Carolina
Isonychia tusculanensis|10LJMAY-029|United States.North Carolina
Isonychia tusculanensis|07GSM-6004|United States.Tennessee
Isonychia tusculanensis|07GSM-6003|United States.Tennessee
Isonychia sp. JMW2|09LJGSM-021|United States.North Carolina
Siphloplecton basale|SWA iBOL UofS 71|Canada.Saskatchewan
Siphloplecton basale|SWA iBOL UofS 53|Canada.Saskatchewan
Siphloplecton basale|08ONMAY-0152|Canada.Ontario
Siphloplecton basale|08ONMAY-0148|Canada.Ontario
Siphloplecton basale|08INHSP-064|United States.Indiana
Siphloplecton basale|08ONMAY-0150|Canada.Ontario
Siphloplecton basale|08ONMAY-0147|Canada.Ontario
Siphloplecton basale|08INHSP-071|United States.Indiana
Siphloplecton basale|09NBMAY-0068|Canada.New Brunswick
Apobaetis sp.LJ1|BIOUG00639-H06|United States.North Carolina
Metretopus borealis|09NBMAY-0561|Canada.New Brunswick
Metretopus borealis|07PROBE-2396|Canada.Manitoba
Metretopus borealis|07PROBE-2380|Canada.Manitoba
Metretopus borealis|07PROBE-2254|Canada.Manitoba
Metretopus borealis|07PROBE-2465|Canada.Manitoba
Metretopus borealis|07PROBE-2464|Canada.Manitoba
Metretopus borealis|07PROBE-2469|Canada.Manitoba
Metretopus borealis|09NBMAY-0438|Canada.New Brunswick
Metretopus borealis|10BBEPT-0077|Canada.Alberta
Ameletus cryptostimulus|BIOUG00640-C04|United States.Tennessee
Ameletus browni|663 GB VT|United States.Vermont
Ameletus browni|662 GBBR VT|United States.Vermont
Ameletus tertius|10HIMXD-0097|United States.North Carolina
Ameletus tertius|HIEPT-1120|United States.North Carolina
Ameletus tertius|HIEPT-1170|United States.Georgia
Ameletus tertius|07GSM-5971|United States.Tennessee
Ameletus tertius|10HIMXD-0098|United States.North Carolina
Ameletus tertius|10HIMXD-0260|United States.North Carolina
Drunella cornutella
Drunella lata complex
Drunella longicornis
Drunella cornuta
Drunella lata
Drunella walkeri
Drunella tuberculata
Farrodes sp.JMW1
Anthopotamus verticis
Isonychia sp.DHF1
Isonychia tusculanensis
Isonychia sp. JMW2
Siphloplecton basale
Apobaetis sp.LJ1
Metretopus borealis
Ameletus cryptostimulus
Ameletus browni
Ameletus tertius

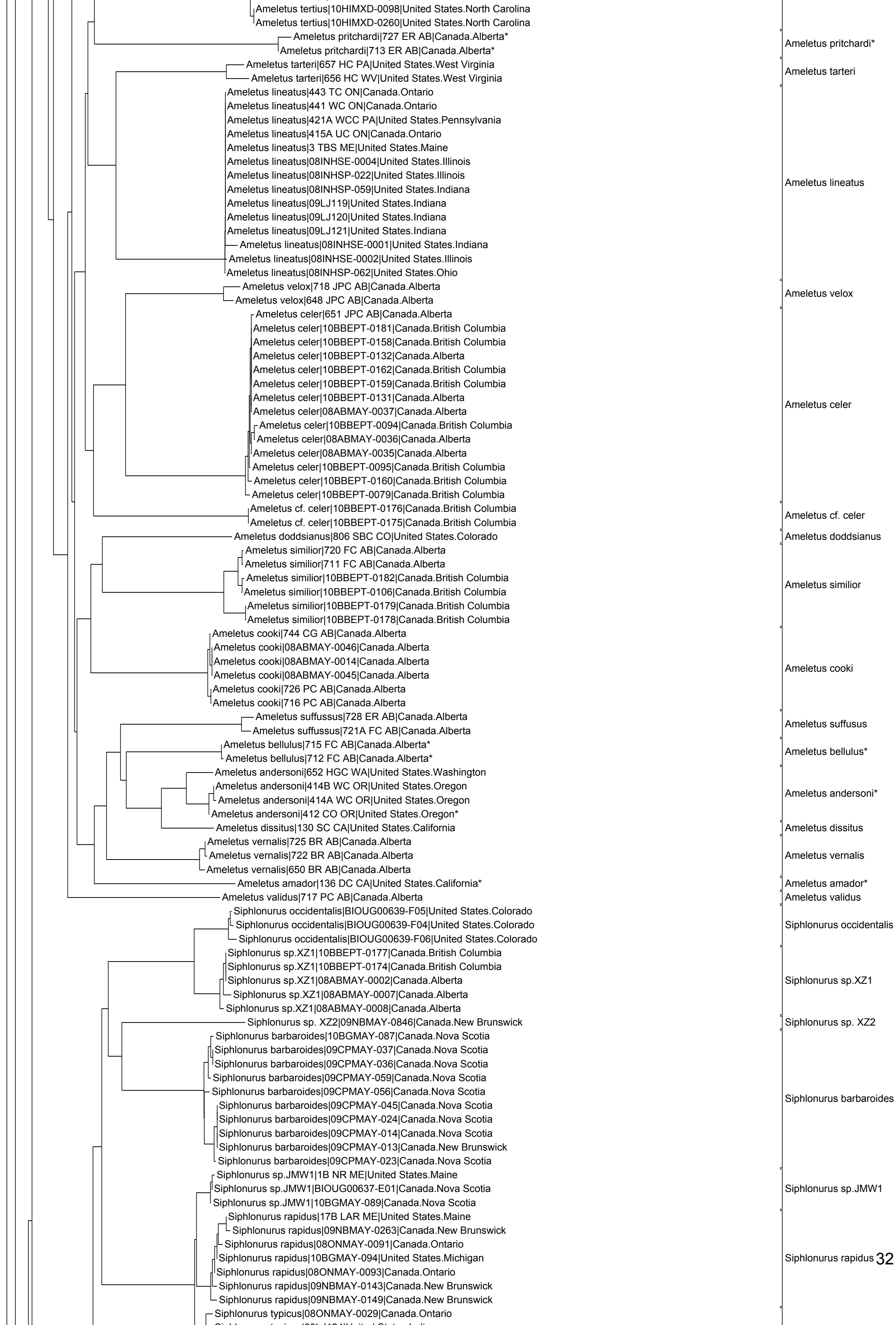
Ameletus tertius|10HIMXD-0098|United States.North Carolina
Ameletus tertius|10HIMXD-0260|United States.North Carolina
Ameletus pritchardi|727 ER AB|Canada.Alberta*
Ameletus pritchardi|713 ER AB|Canada.Alberta*
Ameletus tarteri|657 HC PA|United States.West Virginia
Ameletus tarteri|656 HC WV|United States.West Virginia
Ameletus lineatus|443 TC ON|Canada.Ontario
Ameletus lineatus|441 WC ON|Canada.Ontario
Ameletus lineatus|421A WCC PA|United States.Pennsylvania
Ameletus lineatus|415A UC ON|Canada.Ontario
Ameletus lineatus|3 TBS ME|United States.Maine
Ameletus lineatus|08INHSE-0004|United States.Illinois
Ameletus lineatus|08INHSP-022|United States.Illinois
Ameletus lineatus|08INHSP-059|United States.Indiana
Ameletus lineatus|09LJ119|United States.Indiana
Ameletus lineatus|09LJ120|United States.Indiana
Ameletus lineatus|09LJ121|United States.Indiana
Ameletus lineatus|08INHSE-0001|United States.Indiana
Ameletus lineatus|08INHSE-0002|United States.Illinois
Ameletus lineatus|08INHSP-062|United States.Ohio
Ameletus velox|718 JPC AB|Canada.Alberta
Ameletus velox|648 JPC AB|Canada.Alberta
Ameletus celer|651 JPC AB|Canada.Alberta
Ameletus celer|10BBEPT-0181|Canada.British Columbia
Ameletus celer|10BBEPT-0158|Canada.British Columbia
Ameletus celer|10BBEPT-0132|Canada.Alberta
Ameletus celer|10BBEPT-0162|Canada.British Columbia
Ameletus celer|10BBEPT-0159|Canada.British Columbia
Ameletus celer|10BBEPT-0131|Canada.Alberta
Ameletus celer|08ABMAY-0037|Canada.Alberta
Ameletus celer|10BBEPT-0094|Canada.British Columbia
Ameletus celer|08ABMAY-0036|Canada.Alberta
Ameletus celer|08ABMAY-0035|Canada.Alberta
Ameletus celer|10BBEPT-0095|Canada.British Columbia
Ameletus celer|10BBEPT-0160|Canada.British Columbia
Ameletus celer|10BBEPT-0079|Canada.British Columbia
Ameletus cf. celer|10BBEPT-0176|Canada.British Columbia
Ameletus cf. celer|10BBEPT-0175|Canada.British Columbia
Ameletus doddsianus|806 SBC CO|United States.Colorado
Ameletus similior|720 FC AB|Canada.Alberta
Ameletus similior|711 FC AB|Canada.Alberta
Ameletus similior|10BBEPT-0182|Canada.British Columbia
Ameletus similior|10BBEPT-0106|Canada.British Columbia
Ameletus similior|10BBEPT-0179|Canada.British Columbia
Ameletus similior|10BBEPT-0178|Canada.British Columbia
Ameletus cooki|744 CG AB|Canada.Alberta
Ameletus cooki|08ABMAY-0046|Canada.Alberta
Ameletus cooki|08ABMAY-0014|Canada.Alberta
Ameletus cooki|08ABMAY-0045|Canada.Alberta
Ameletus cooki|726 PC AB|Canada.Alberta
Ameletus cooki|716 PC AB|Canada.Alberta
Ameletus suffussus|728 ER AB|Canada.Alberta
Ameletus suffussus|721A FC AB|Canada.Alberta
Ameletus bellulus|715 FC AB|Canada.Alberta*
Ameletus bellulus|712 FC AB|Canada.Alberta*
Ameletus andersoni|652 HGC WA|United States.Washington
Ameletus andersoni|414B WC OR|United States.Oregon
Ameletus andersoni|414A WC OR|United States.Oregon
Ameletus andersoni|412 CO OR|United States.Oregon*
Ameletus dissitus|130 SC CA|United States.California
Ameletus vernalis|725 BR AB|Canada.Alberta
Ameletus vernalis|722 BR AB|Canada.Alberta
Ameletus vernalis|650 BR AB|Canada.Alberta
Ameletus amador|136 DC CA|United States.California*
Ameletus validus|717 PC AB|Canada.Alberta
Siphlonurus occidentalis|BIOUG00639-F05|United States.Colorado
Siphlonurus occidentalis|BIOUG00639-F04|United States.Colorado
Siphlonurus occidentalis|BIOUG00639-F06|United States.Colorado
Siphlonurus sp.XZ1|10BBEPT-0177|Canada.British Columbia
Siphlonurus sp.XZ1|10BBEPT-0174|Canada.British Columbia
Siphlonurus sp.XZ1|08ABMAY-0002|Canada.Alberta
Siphlonurus sp.XZ1|08ABMAY-0007|Canada.Alberta
Siphlonurus sp.XZ1|08ABMAY-0008|Canada.Alberta
Siphlonurus sp. XZ2|09NBMAY-0846|Canada.New Brunswick
Siphlonurus barbaroides|10BGMAY-087|Canada.Nova Scotia
Siphlonurus barbaroides|09CPMAY-037|Canada.Nova Scotia
Siphlonurus barbaroides|09CPMAY-036|Canada.Nova Scotia
Siphlonurus barbaroides|09CPMAY-059|Canada.Nova Scotia
Siphlonurus barbaroides|09CPMAY-056|Canada.Nova Scotia
Siphlonurus barbaroides|09CPMAY-045|Canada.Nova Scotia
Siphlonurus barbaroides|09CPMAY-024|Canada.Nova Scotia
Siphlonurus barbaroides|09CPMAY-014|Canada.Nova Scotia
Siphlonurus barbaroides|09CPMAY-013|Canada.New Brunswick
Siphlonurus barbaroides|09CPMAY-023|Canada.Nova Scotia
Siphlonurus sp.JMW1|1B NR ME|United States.Maine
Siphlonurus sp.JMW1|BIOUG00637-E01|Canada.Nova Scotia
Siphlonurus sp.JMW1|10BGMAY-089|Canada.Nova Scotia
Siphlonurus rapidus|17B LAR ME|United States.Maine
Siphlonurus rapidus|09NBMAY-0263|Canada.New Brunswick
Siphlonurus rapidus|08ONMAY-0091|Canada.Ontario
Siphlonurus rapidus|10BGMAY-094|United States.Michigan
Siphlonurus rapidus|08ONMAY-0093|Canada.Ontario
Siphlonurus rapidus|09NBMAY-0143|Canada.New Brunswick
Siphlonurus rapidus|09NBMAY-0149|Canada.New Brunswick
Siphlonurus typicus|08ONMAY-0029|Canada.Ontario
Ameletus pritchardi*
Ameletus tarteri
Ameletus lineatus
Ameletus velox
Ameletus celer
Ameletus cf. celer
Ameletus doddsianus
Ameletus similior
Ameletus cooki
Ameletus suffusus
Ameletus bellulus*
Ameletus andersoni*
Ameletus dissitus
Ameletus vernalis
Ameletus amador*
Ameletus validus
Siphlonurus occidentalis
Siphlonurus sp.XZ1
Siphlonurus sp. XZ2
Siphlonurus barbaroides
Siphlonurus sp.JMW1
Siphlonurus rapidus

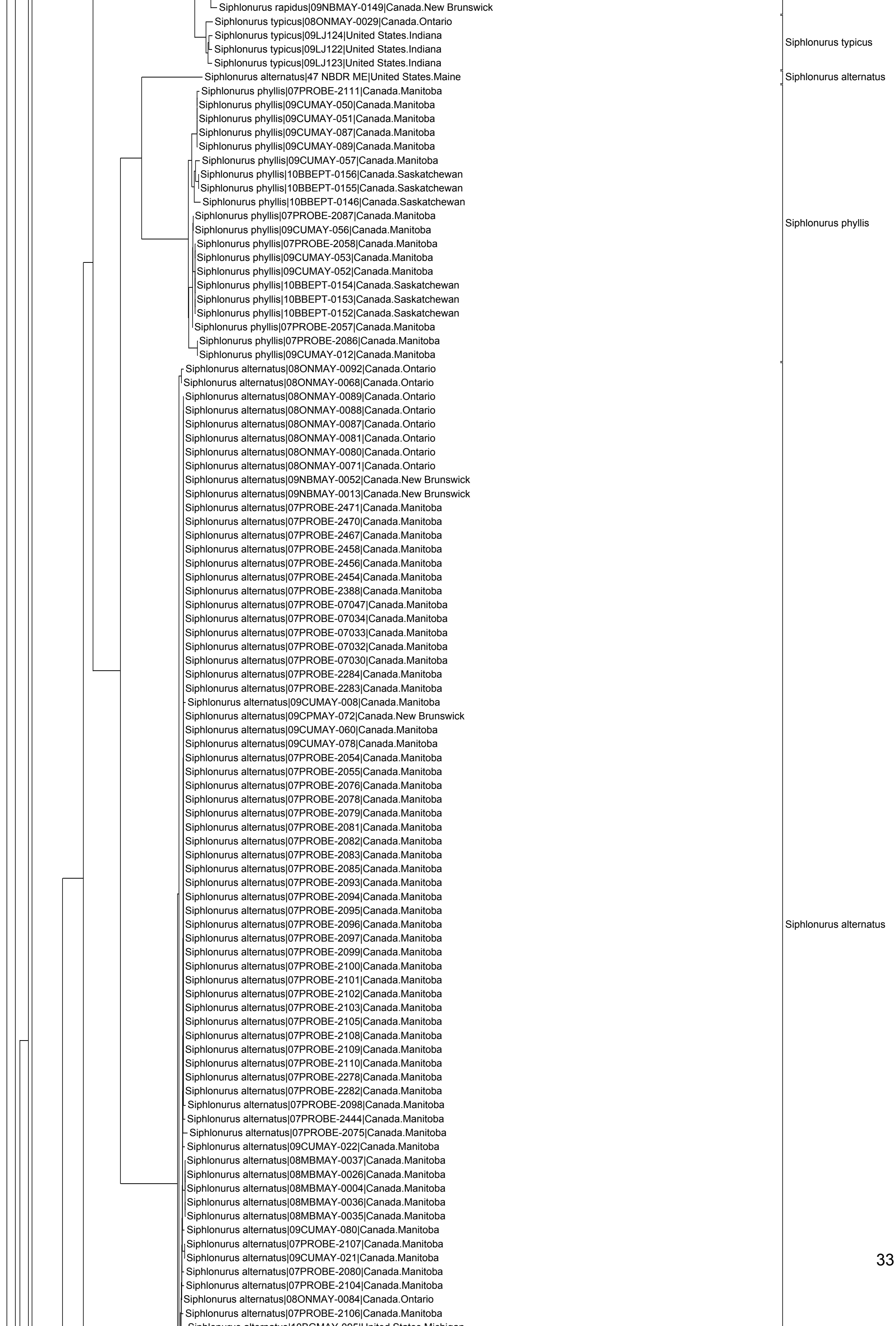
Siphlonurus rapidus|09NBMAY-0149|Canada.New Brunswick
Siphlonurus typicus|08ONMAY-0029|Canada.Ontario
Siphlonurus typicus|09LJ124|United States.Indiana
Siphlonurus typicus|09LJ122|United States.Indiana
Siphlonurus typicus|09LJ123|United States.Indiana
Siphlonurus alternatus|47 NBDR ME|United States.Maine
Siphlonurus phyllis|07PROBE-2111|Canada.Manitoba
Siphlonurus phyllis|09CUMAY-050|Canada.Manitoba
Siphlonurus phyllis|09CUMAY-051|Canada.Manitoba
Siphlonurus phyllis|09CUMAY-087|Canada.Manitoba
Siphlonurus phyllis|09CUMAY-089|Canada.Manitoba
Siphlonurus phyllis|09CUMAY-057|Canada.Manitoba
Siphlonurus phyllis|10BBEPT-0156|Canada.Saskatchewan
Siphlonurus phyllis|10BBEPT-0155|Canada.Saskatchewan
Siphlonurus phyllis|10BBEPT-0146|Canada.Saskatchewan
Siphlonurus phyllis|07PROBE-2087|Canada.Manitoba
Siphlonurus phyllis|09CUMAY-056|Canada.Manitoba
Siphlonurus phyllis|07PROBE-2058|Canada.Manitoba
Siphlonurus phyllis|09CUMAY-053|Canada.Manitoba
Siphlonurus phyllis|09CUMAY-052|Canada.Manitoba
Siphlonurus phyllis|10BBEPT-0154|Canada.Saskatchewan
Siphlonurus phyllis|10BBEPT-0153|Canada.Saskatchewan
Siphlonurus phyllis|10BBEPT-0152|Canada.Saskatchewan
Siphlonurus phyllis|07PROBE-2057|Canada.Manitoba
Siphlonurus phyllis|07PROBE-2086|Canada.Manitoba
Siphlonurus phyllis|09CUMAY-012|Canada.Manitoba
Siphlonurus alternatus|08ONMAY-0092|Canada.Ontario
Siphlonurus alternatus|08ONMAY-0068|Canada.Ontario
Siphlonurus alternatus|08ONMAY-0089|Canada.Ontario
Siphlonurus alternatus|08ONMAY-0088|Canada.Ontario
Siphlonurus alternatus|08ONMAY-0087|Canada.Ontario
Siphlonurus alternatus|08ONMAY-0081|Canada.Ontario
Siphlonurus alternatus|08ONMAY-0080|Canada.Ontario
Siphlonurus alternatus|08ONMAY-0071|Canada.Ontario
Siphlonurus alternatus|09NBMAY-0052|Canada.New Brunswick
Siphlonurus alternatus|09NBMAY-0013|Canada.New Brunswick
Siphlonurus alternatus|07PROBE-2471|Canada.Manitoba
Siphlonurus alternatus|07PROBE-2470|Canada.Manitoba
Siphlonurus alternatus|07PROBE-2467|Canada.Manitoba
Siphlonurus alternatus|07PROBE-2458|Canada.Manitoba
Siphlonurus alternatus|07PROBE-2456|Canada.Manitoba
Siphlonurus alternatus|07PROBE-2454|Canada.Manitoba
Siphlonurus alternatus|07PROBE-2388|Canada.Manitoba
Siphlonurus alternatus|07PROBE-07047|Canada.Manitoba
Siphlonurus alternatus|07PROBE-07034|Canada.Manitoba
Siphlonurus alternatus|07PROBE-07033|Canada.Manitoba
Siphlonurus alternatus|07PROBE-07032|Canada.Manitoba
Siphlonurus alternatus|07PROBE-07030|Canada.Manitoba
Siphlonurus alternatus|07PROBE-2284|Canada.Manitoba
Siphlonurus alternatus|07PROBE-2283|Canada.Manitoba
Siphlonurus alternatus|09CUMAY-008|Canada.Manitoba
Siphlonurus alternatus|09CPMAY-072|Canada.New Brunswick
Siphlonurus alternatus|09CUMAY-060|Canada.Manitoba
Siphlonurus alternatus|09CUMAY-078|Canada.Manitoba
Siphlonurus alternatus|07PROBE-2054|Canada.Manitoba
Siphlonurus alternatus|07PROBE-2055|Canada.Manitoba
Siphlonurus alternatus|07PROBE-2076|Canada.Manitoba
Siphlonurus alternatus|07PROBE-2078|Canada.Manitoba
Siphlonurus alternatus|07PROBE-2079|Canada.Manitoba
Siphlonurus alternatus|07PROBE-2081|Canada.Manitoba
Siphlonurus alternatus|07PROBE-2082|Canada.Manitoba
Siphlonurus alternatus|07PROBE-2083|Canada.Manitoba
Siphlonurus alternatus|07PROBE-2085|Canada.Manitoba
Siphlonurus alternatus|07PROBE-2093|Canada.Manitoba
Siphlonurus alternatus|07PROBE-2094|Canada.Manitoba
Siphlonurus alternatus|07PROBE-2095|Canada.Manitoba
Siphlonurus alternatus|07PROBE-2096|Canada.Manitoba
Siphlonurus alternatus|07PROBE-2097|Canada.Manitoba
Siphlonurus alternatus|07PROBE-2099|Canada.Manitoba
Siphlonurus alternatus|07PROBE-2100|Canada.Manitoba
Siphlonurus alternatus|07PROBE-2101|Canada.Manitoba
Siphlonurus alternatus|07PROBE-2102|Canada.Manitoba
Siphlonurus alternatus|07PROBE-2103|Canada.Manitoba
Siphlonurus alternatus|07PROBE-2105|Canada.Manitoba
Siphlonurus alternatus|07PROBE-2108|Canada.Manitoba
Siphlonurus alternatus|07PROBE-2109|Canada.Manitoba
Siphlonurus alternatus|07PROBE-2110|Canada.Manitoba
Siphlonurus alternatus|07PROBE-2278|Canada.Manitoba
Siphlonurus alternatus|07PROBE-2282|Canada.Manitoba
Siphlonurus alternatus|07PROBE-2098|Canada.Manitoba
Siphlonurus alternatus|07PROBE-2444|Canada.Manitoba
Siphlonurus alternatus|07PROBE-2075|Canada.Manitoba
Siphlonurus alternatus|09CUMAY-022|Canada.Manitoba
Siphlonurus alternatus|08MBMAY-0037|Canada.Manitoba
Siphlonurus alternatus|08MBMAY-0026|Canada.Manitoba
Siphlonurus alternatus|08MBMAY-0004|Canada.Manitoba
Siphlonurus alternatus|08MBMAY-0036|Canada.Manitoba
Siphlonurus alternatus|08MBMAY-0035|Canada.Manitoba
Siphlonurus alternatus|09CUMAY-080|Canada.Manitoba
Siphlonurus alternatus|07PROBE-2107|Canada.Manitoba
Siphlonurus alternatus|09CUMAY-021|Canada.Manitoba
Siphlonurus alternatus|07PROBE-2080|Canada.Manitoba
Siphlonurus alternatus|07PROBE-2104|Canada.Manitoba
Siphlonurus alternatus|08ONMAY-0084|Canada.Ontario
Siphlonurus alternatus|07PROBE-2106|Canada.Manitoba
Siphlonurus typicus
Siphlonurus alternatus
Siphlonurus phyllis
Siphlonurus alternatus

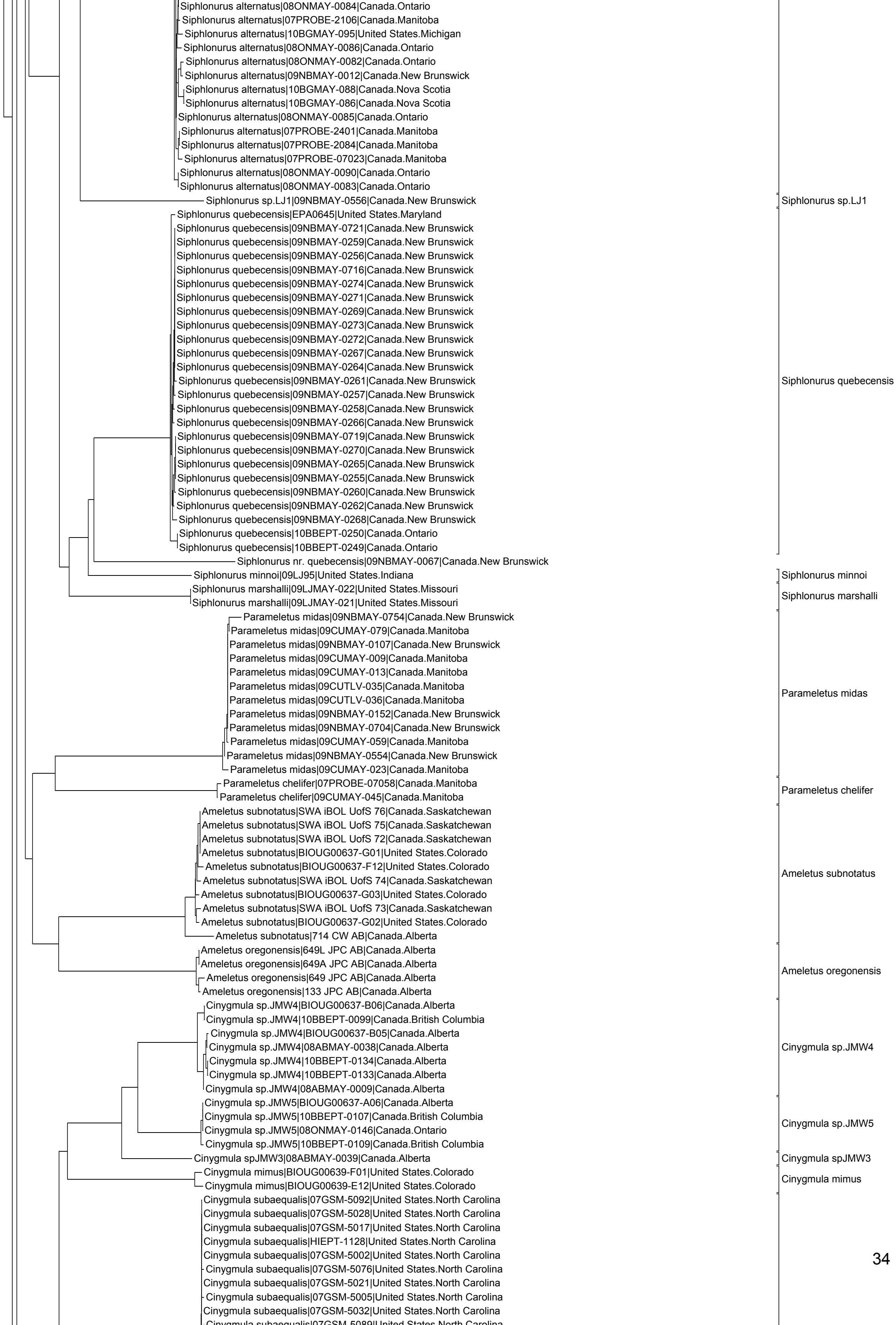

Siphlonurus alternatus|08ONMAY-0084|Canada.Ontario
Siphlonurus alternatus|07PROBE-2106|Canada.Manitoba
Siphlonurus alternatus|10BGMAY-095|United States.Michigan
Siphlonurus alternatus|08ONMAY-0086|Canada.Ontario
Siphlonurus alternatus|08ONMAY-0082|Canada.Ontario
Siphlonurus alternatus|09NBMAY-0012|Canada.New Brunswick
Siphlonurus alternatus|10BGMAY-088|Canada.Nova Scotia
Siphlonurus alternatus|10BGMAY-086|Canada.Nova Scotia
Siphlonurus alternatus|08ONMAY-0085|Canada.Ontario
Siphlonurus alternatus|07PROBE-2401|Canada.Manitoba
Siphlonurus alternatus|07PROBE-2084|Canada.Manitoba
Siphlonurus alternatus|07PROBE-07023|Canada.Manitoba
Siphlonurus alternatus|08ONMAY-0090|Canada.Ontario
Siphlonurus alternatus|08ONMAY-0083|Canada.Ontario
Siphlonurus sp.LJ1|09NBMAY-0556|Canada.New Brunswick
Siphlonurus sp.LJ1
Siphlonurus quebecensis|EPA0645|United States.Maryland
Siphlonurus quebecensis|09NBMAY-0721|Canada.New Brunswick
Siphlonurus quebecensis|09NBMAY-0259|Canada.New Brunswick
Siphlonurus quebecensis|09NBMAY-0256|Canada.New Brunswick
Siphlonurus quebecensis|09NBMAY-0716|Canada.New Brunswick
Siphlonurus quebecensis|09NBMAY-0274|Canada.New Brunswick
Siphlonurus quebecensis|09NBMAY-0271|Canada.New Brunswick
Siphlonurus quebecensis|09NBMAY-0269|Canada.New Brunswick
Siphlonurus quebecensis|09NBMAY-0273|Canada.New Brunswick
Siphlonurus quebecensis|09NBMAY-0272|Canada.New Brunswick
Siphlonurus quebecensis|09NBMAY-0267|Canada.New Brunswick
Siphlonurus quebecensis|09NBMAY-0264|Canada.New Brunswick
Siphlonurus quebecensis|09NBMAY-0261|Canada.New Brunswick
Siphlonurus quebecensis
Siphlonurus quebecensis|09NBMAY-0257|Canada.New Brunswick
Siphlonurus quebecensis|09NBMAY-0258|Canada.New Brunswick
Siphlonurus quebecensis|09NBMAY-0266|Canada.New Brunswick
Siphlonurus quebecensis|09NBMAY-0719|Canada.New Brunswick
Siphlonurus quebecensis|09NBMAY-0270|Canada.New Brunswick
Siphlonurus quebecensis|09NBMAY-0265|Canada.New Brunswick
Siphlonurus quebecensis|09NBMAY-0255|Canada.New Brunswick
Siphlonurus quebecensis|09NBMAY-0260|Canada.New Brunswick
Siphlonurus quebecensis|09NBMAY-0262|Canada.New Brunswick
Siphlonurus quebecensis|09NBMAY-0268|Canada.New Brunswick
Siphlonurus quebecensis|10BBEPT-0250|Canada.Ontario
Siphlonurus quebecensis|10BBEPT-0249|Canada.Ontario
Siphlonurus nr. quebecensis|09NBMAY-0067|Canada.New Brunswick
Siphlonurus minnoi|09LJ95|United States.Indiana
Siphlonurus minnoi
Siphlonurus marshalli|09LJMAY-022|United States.Missouri
Siphlonurus marshalli
Siphlonurus marshalli|09LJMAY-021|United States.Missouri
Parameletus midas|09NBMAY-0754|Canada.New Brunswick
Parameletus midas|09CUMAY-079|Canada.Manitoba
Parameletus midas|09NBMAY-0107|Canada.New Brunswick
Parameletus midas|09CUMAY-009|Canada.Manitoba
Parameletus midas|09CUMAY-013|Canada.Manitoba
Parameletus midas|09CUTLV-035|Canada.Manitoba
Parameletus midas
Parameletus midas|09CUTLV-036|Canada.Manitoba
Parameletus midas|09NBMAY-0152|Canada.New Brunswick
Parameletus midas|09NBMAY-0704|Canada.New Brunswick
Parameletus midas|09CUMAY-059|Canada.Manitoba
Parameletus midas|09NBMAY-0554|Canada.New Brunswick
Parameletus midas|09CUMAY-023|Canada.Manitoba
Parameletus chelifer|07PROBE-07058|Canada.Manitoba
Parameletus chelifer
Parameletus chelifer|09CUMAY-045|Canada.Manitoba
Ameletus subnotatus|SWA iBOL UofS 76|Canada.Saskatchewan
Ameletus subnotatus|SWA iBOL UofS 75|Canada.Saskatchewan
Ameletus subnotatus|SWA iBOL UofS 72|Canada.Saskatchewan
Ameletus subnotatus|BIOUG00637-G01|United States.Colorado
Ameletus subnotatus|BIOUG00637-F12|United States.Colorado
Ameletus subnotatus
Ameletus subnotatus|SWA iBOL UofS 74|Canada.Saskatchewan
Ameletus subnotatus|BIOUG00637-G03|United States.Colorado
Ameletus subnotatus|SWA iBOL UofS 73|Canada.Saskatchewan
Ameletus subnotatus|BIOUG00637-G02|United States.Colorado
Ameletus subnotatus|714 CW AB|Canada.Alberta
Ameletus oregonensis|649L JPC AB|Canada.Alberta
Ameletus oregonensis|649A JPC AB|Canada.Alberta
Ameletus oregonensis
Ameletus oregonensis|649 JPC AB|Canada.Alberta
Ameletus oregonensis|133 JPC AB|Canada.Alberta
Cinygmula sp.JMW4|BIOUG00637-B06|Canada.Alberta
Cinygmula sp.JMW4|10BBEPT-0099|Canada.British Columbia
Cinygmula sp.JMW4|BIOUG00637-B05|Canada.Alberta
Cinygmula sp.JMW4|08ABMAY-0038|Canada.Alberta
Cinygmula sp.JMW4
Cinygmula sp.JMW4|10BBEPT-0134|Canada.Alberta
Cinygmula sp.JMW4|10BBEPT-0133|Canada.Alberta
Cinygmula sp.JMW4|08ABMAY-0009|Canada.Alberta
Cinygmula sp.JMW5|BIOUG00637-A06|Canada.Alberta
Cinygmula sp.JMW5|10BBEPT-0107|Canada.British Columbia
Cinygmula sp.JMW5
Cinygmula sp.JMW5|08ONMAY-0146|Canada.Ontario
Cinygmula sp.JMW5|10BBEPT-0109|Canada.British Columbia
Cinygmula spJMW3|08ABMAY-0039|Canada.Alberta
Cinygmula spJMW3
Cinygmula mimus|BIOUG00639-F01|United States.Colorado
Cinygmula mimus
Cinygmula mimus|BIOUG00639-E12|United States.Colorado
Cinygmula subaequalis|07GSM-5092|United States.North Carolina
Cinygmula subaequalis|07GSM-5028|United States.North Carolina
Cinygmula subaequalis|07GSM-5017|United States.North Carolina
Cinygmula subaequalis|HIEPT-1128|United States.North Carolina
Cinygmula subaequalis|07GSM-5002|United States.North Carolina
Cinygmula subaequalis|07GSM-5076|United States.North Carolina
Cinygmula subaequalis|07GSM-5021|United States.North Carolina
Cinygmula subaequalis|07GSM-5005|United States.North Carolina
Cinygmula subaequalis|07GSM-5032|United States.North Carolina

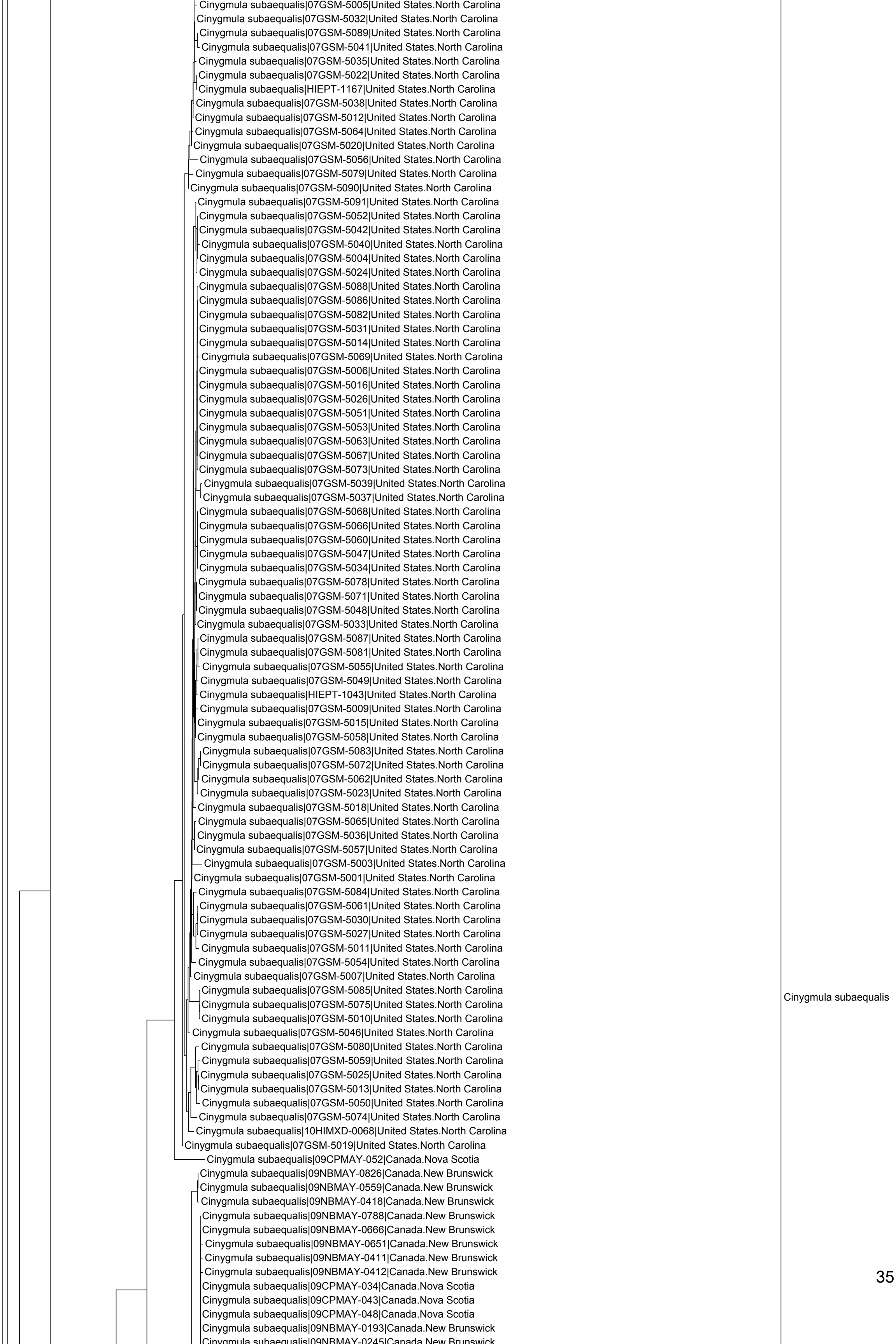
Cinygmula subaequalis|07GSM-5005|United States.North Carolina
Cinygmula subaequalis|07GSM-5032|United States.North Carolina
Cinygmula subaequalis|07GSM-5089|United States.North Carolina
Cinygmula subaequalis|07GSM-5041|United States.North Carolina
Cinygmula subaequalis|07GSM-5035|United States.North Carolina
Cinygmula subaequalis|07GSM-5022|United States.North Carolina
Cinygmula subaequalis|HIEPT-1167|United States.North Carolina
Cinygmula subaequalis|07GSM-5038|United States.North Carolina
Cinygmula subaequalis|07GSM-5012|United States.North Carolina
Cinygmula subaequalis|07GSM-5064|United States.North Carolina
Cinygmula subaequalis|07GSM-5020|United States.North Carolina
Cinygmula subaequalis|07GSM-5056|United States.North Carolina
Cinygmula subaequalis|07GSM-5079|United States.North Carolina
Cinygmula subaequalis|07GSM-5090|United States.North Carolina
Cinygmula subaequalis|07GSM-5091|United States.North Carolina
Cinygmula subaequalis|07GSM-5052|United States.North Carolina
Cinygmula subaequalis|07GSM-5042|United States.North Carolina
Cinygmula subaequalis|07GSM-5040|United States.North Carolina
Cinygmula subaequalis|07GSM-5004|United States.North Carolina
Cinygmula subaequalis|07GSM-5024|United States.North Carolina
Cinygmula subaequalis|07GSM-5088|United States.North Carolina
Cinygmula subaequalis|07GSM-5086|United States.North Carolina
Cinygmula subaequalis|07GSM-5082|United States.North Carolina
Cinygmula subaequalis|07GSM-5031|United States.North Carolina
Cinygmula subaequalis|07GSM-5014|United States.North Carolina
Cinygmula subaequalis|07GSM-5069|United States.North Carolina
Cinygmula subaequalis|07GSM-5006|United States.North Carolina
Cinygmula subaequalis|07GSM-5016|United States.North Carolina
Cinygmula subaequalis|07GSM-5026|United States.North Carolina
Cinygmula subaequalis|07GSM-5051|United States.North Carolina
Cinygmula subaequalis|07GSM-5053|United States.North Carolina
Cinygmula subaequalis|07GSM-5063|United States.North Carolina
Cinygmula subaequalis|07GSM-5067|United States.North Carolina
Cinygmula subaequalis|07GSM-5073|United States.North Carolina
Cinygmula subaequalis|07GSM-5039|United States.North Carolina
Cinygmula subaequalis|07GSM-5037|United States.North Carolina
Cinygmula subaequalis|07GSM-5068|United States.North Carolina
Cinygmula subaequalis|07GSM-5066|United States.North Carolina
Cinygmula subaequalis|07GSM-5060|United States.North Carolina
Cinygmula subaequalis|07GSM-5047|United States.North Carolina
Cinygmula subaequalis|07GSM-5034|United States.North Carolina
Cinygmula subaequalis|07GSM-5078|United States.North Carolina
Cinygmula subaequalis|07GSM-5071|United States.North Carolina
Cinygmula subaequalis|07GSM-5048|United States.North Carolina
Cinygmula subaequalis|07GSM-5033|United States.North Carolina
Cinygmula subaequalis|07GSM-5087|United States.North Carolina
Cinygmula subaequalis|07GSM-5081|United States.North Carolina
Cinygmula subaequalis|07GSM-5055|United States.North Carolina
Cinygmula subaequalis|07GSM-5049|United States.North Carolina
Cinygmula subaequalis|HIEPT-1043|United States.North Carolina
Cinygmula subaequalis|07GSM-5009|United States.North Carolina
Cinygmula subaequalis|07GSM-5015|United States.North Carolina
Cinygmula subaequalis|07GSM-5058|United States.North Carolina
Cinygmula subaequalis|07GSM-5083|United States.North Carolina
Cinygmula subaequalis|07GSM-5072|United States.North Carolina
Cinygmula subaequalis|07GSM-5062|United States.North Carolina
Cinygmula subaequalis|07GSM-5023|United States.North Carolina
Cinygmula subaequalis|07GSM-5018|United States.North Carolina
Cinygmula subaequalis|07GSM-5065|United States.North Carolina
Cinygmula subaequalis|07GSM-5036|United States.North Carolina
Cinygmula subaequalis|07GSM-5057|United States.North Carolina
Cinygmula subaequalis|07GSM-5003|United States.North Carolina
Cinygmula subaequalis|07GSM-5001|United States.North Carolina
Cinygmula subaequalis|07GSM-5084|United States.North Carolina
Cinygmula subaequalis|07GSM-5061|United States.North Carolina
Cinygmula subaequalis|07GSM-5030|United States.North Carolina
Cinygmula subaequalis|07GSM-5027|United States.North Carolina
Cinygmula subaequalis|07GSM-5011|United States.North Carolina
Cinygmula subaequalis|07GSM-5054|United States.North Carolina
Cinygmula subaequalis|07GSM-5007|United States.North Carolina
Cinygmula subaequalis|07GSM-5085|United States.North Carolina
Cinygmula subaequalis|07GSM-5075|United States.North Carolina
Cinygmula subaequalis|07GSM-5010|United States.North Carolina
Cinygmula subaequalis|07GSM-5046|United States.North Carolina
Cinygmula subaequalis|07GSM-5080|United States.North Carolina
Cinygmula subaequalis|07GSM-5059|United States.North Carolina
Cinygmula subaequalis|07GSM-5025|United States.North Carolina
Cinygmula subaequalis|07GSM-5013|United States.North Carolina
Cinygmula subaequalis|07GSM-5050|United States.North Carolina
Cinygmula subaequalis|07GSM-5074|United States.North Carolina
Cinygmula subaequalis|10HIMXD-0068|United States.North Carolina
Cinygmula subaequalis|07GSM-5019|United States.North Carolina
Cinygmula subaequalis|09CPMAY-052|Canada.Nova Scotia
Cinygmula subaequalis|09NBMAY-0826|Canada.New Brunswick
Cinygmula subaequalis|09NBMAY-0559|Canada.New Brunswick
Cinygmula subaequalis|09NBMAY-0418|Canada.New Brunswick
Cinygmula subaequalis|09NBMAY-0788|Canada.New Brunswick
Cinygmula subaequalis|09NBMAY-0666|Canada.New Brunswick
Cinygmula subaequalis|09NBMAY-0651|Canada.New Brunswick
Cinygmula subaequalis|09NBMAY-0411|Canada.New Brunswick
Cinygmula subaequalis|09NBMAY-0412|Canada.New Brunswick
Cinygmula subaequalis|09CPMAY-034|Canada.Nova Scotia
Cinygmula subaequalis|09CPMAY-043|Canada.Nova Scotia
Cinygmula subaequalis|09CPMAY-048|Canada.Nova Scotia
Cinygmula subaequalis|09NBMAY-0193|Canada.New Brunswick
Cinygmula subaequalis|09NBMAY-0245|Canada.New Brunswick
Cinygmula subaequalis

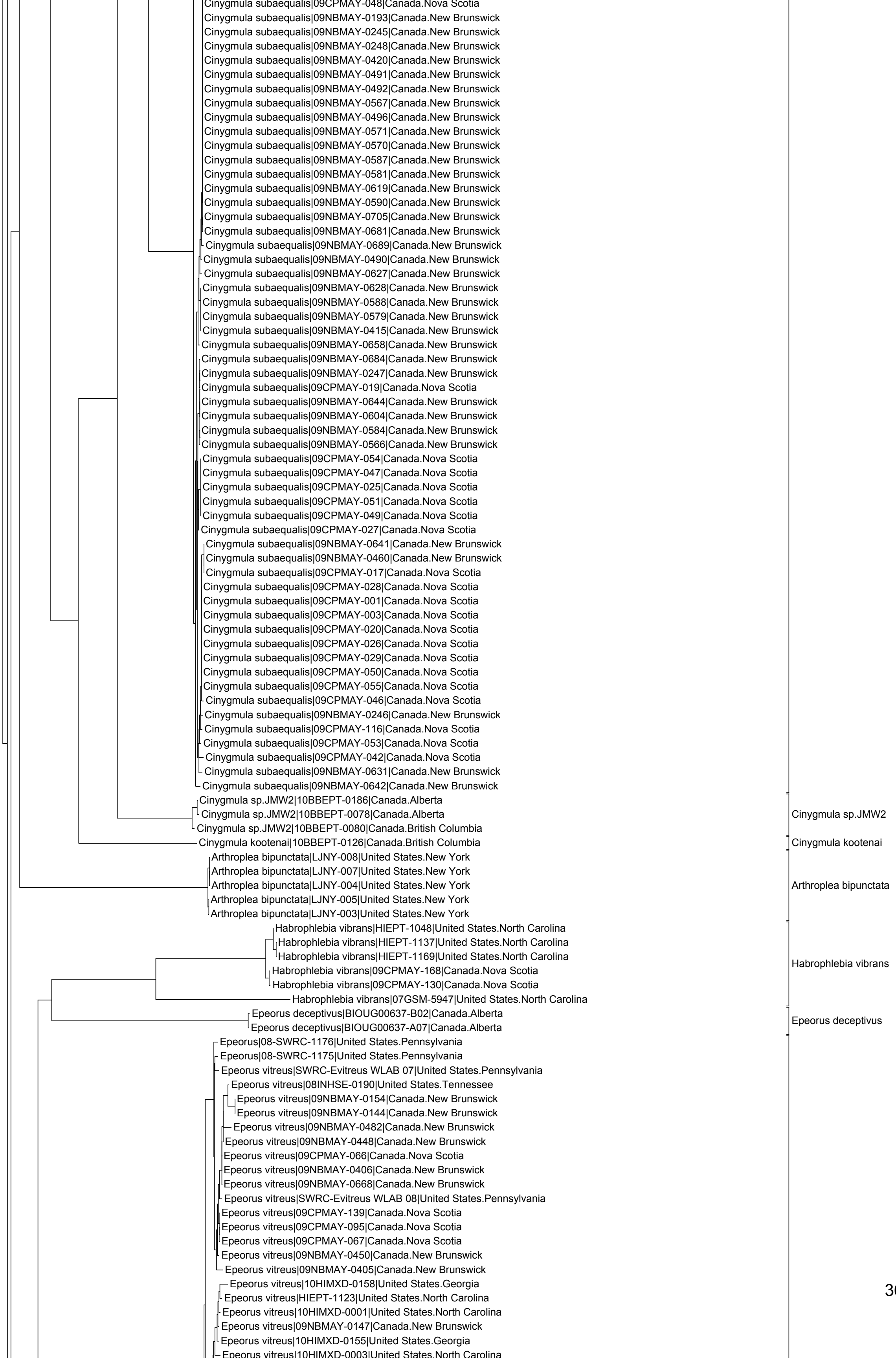
Cinygmula subaequalis|09CPMAY-048|Canada.Nova Scotia
Cinygmula subaequalis|09NBMAY-0193|Canada.New Brunswick
Cinygmula subaequalis|09NBMAY-0245|Canada.New Brunswick
Cinygmula subaequalis|09NBMAY-0248|Canada.New Brunswick
Cinygmula subaequalis|09NBMAY-0420|Canada.New Brunswick
Cinygmula subaequalis|09NBMAY-0491|Canada.New Brunswick
Cinygmula subaequalis|09NBMAY-0492|Canada.New Brunswick
Cinygmula subaequalis|09NBMAY-0567|Canada.New Brunswick
Cinygmula subaequalis|09NBMAY-0496|Canada.New Brunswick
Cinygmula subaequalis|09NBMAY-0571|Canada.New Brunswick
Cinygmula subaequalis|09NBMAY-0570|Canada.New Brunswick
Cinygmula subaequalis|09NBMAY-0587|Canada.New Brunswick
Cinygmula subaequalis|09NBMAY-0581|Canada.New Brunswick
Cinygmula subaequalis|09NBMAY-0619|Canada.New Brunswick
Cinygmula subaequalis|09NBMAY-0590|Canada.New Brunswick
Cinygmula subaequalis|09NBMAY-0705|Canada.New Brunswick
Cinygmula subaequalis|09NBMAY-0681|Canada.New Brunswick
Cinygmula subaequalis|09NBMAY-0689|Canada.New Brunswick
Cinygmula subaequalis|09NBMAY-0490|Canada.New Brunswick
Cinygmula subaequalis|09NBMAY-0627|Canada.New Brunswick
Cinygmula subaequalis|09NBMAY-0628|Canada.New Brunswick
Cinygmula subaequalis|09NBMAY-0588|Canada.New Brunswick
Cinygmula subaequalis|09NBMAY-0579|Canada.New Brunswick
Cinygmula subaequalis|09NBMAY-0415|Canada.New Brunswick
Cinygmula subaequalis|09NBMAY-0658|Canada.New Brunswick
Cinygmula subaequalis|09NBMAY-0684|Canada.New Brunswick
Cinygmula subaequalis|09NBMAY-0247|Canada.New Brunswick
Cinygmula subaequalis|09CPMAY-019|Canada.Nova Scotia
Cinygmula subaequalis|09NBMAY-0644|Canada.New Brunswick
Cinygmula subaequalis|09NBMAY-0604|Canada.New Brunswick
Cinygmula subaequalis|09NBMAY-0584|Canada.New Brunswick
Cinygmula subaequalis|09NBMAY-0566|Canada.New Brunswick
Cinygmula subaequalis|09CPMAY-054|Canada.Nova Scotia
Cinygmula subaequalis|09CPMAY-047|Canada.Nova Scotia
Cinygmula subaequalis|09CPMAY-025|Canada.Nova Scotia
Cinygmula subaequalis|09CPMAY-051|Canada.Nova Scotia
Cinygmula subaequalis|09CPMAY-049|Canada.Nova Scotia
Cinygmula subaequalis|09CPMAY-027|Canada.Nova Scotia
Cinygmula subaequalis|09NBMAY-0641|Canada.New Brunswick
Cinygmula subaequalis|09NBMAY-0460|Canada.New Brunswick
Cinygmula subaequalis|09CPMAY-017|Canada.Nova Scotia
Cinygmula subaequalis|09CPMAY-028|Canada.Nova Scotia
Cinygmula subaequalis|09CPMAY-001|Canada.Nova Scotia
Cinygmula subaequalis|09CPMAY-003|Canada.Nova Scotia
Cinygmula subaequalis|09CPMAY-020|Canada.Nova Scotia
Cinygmula subaequalis|09CPMAY-026|Canada.Nova Scotia
Cinygmula subaequalis|09CPMAY-029|Canada.Nova Scotia
Cinygmula subaequalis|09CPMAY-050|Canada.Nova Scotia
Cinygmula subaequalis|09CPMAY-055|Canada.Nova Scotia
Cinygmula subaequalis|09CPMAY-046|Canada.Nova Scotia
Cinygmula subaequalis|09NBMAY-0246|Canada.New Brunswick
Cinygmula subaequalis|09CPMAY-116|Canada.Nova Scotia
Cinygmula subaequalis|09CPMAY-053|Canada.Nova Scotia
Cinygmula subaequalis|09CPMAY-042|Canada.Nova Scotia
Cinygmula subaequalis|09NBMAY-0631|Canada.New Brunswick
Cinygmula subaequalis|09NBMAY-0642|Canada.New Brunswick
Cinygmula sp.JMW2|10BBEPT-0186|Canada.Alberta
Cinygmula sp.JMW2|10BBEPT-0078|Canada.Alberta
Cinygmula sp.JMW2|10BBEPT-0080|Canada.British Columbia
Cinygmula sp.JMW2
Cinygmula kootenai|10BBEPT-0126|Canada.British Columbia
Cinygmula kootenai
Arthroplea bipunctata|LJNY-008|United States.New York
Arthroplea bipunctata|LJNY-007|United States.New York
Arthroplea bipunctata|LJNY-004|United States.New York
Arthroplea bipunctata|LJNY-005|United States.New York
Arthroplea bipunctata|LJNY-003|United States.New York
Arthroplea bipunctata
Habrophlebia vibrans|HIEPT-1048|United States.North Carolina
Habrophlebia vibrans|HIEPT-1137|United States.North Carolina
Habrophlebia vibrans|HIEPT-1169|United States.North Carolina
Habrophlebia vibrans|09CPMAY-168|Canada.Nova Scotia
Habrophlebia vibrans|09CPMAY-130|Canada.Nova Scotia
Habrophlebia vibrans|07GSM-5947|United States.North Carolina
Habrophlebia vibrans
Epeorus deceptivus|BIOUG00637-B02|Canada.Alberta
Epeorus deceptivus|BIOUG00637-A07|Canada.Alberta
Epeorus deceptivus
Epeorus|08-SWRC-1176|United States.Pennsylvania
Epeorus|08-SWRC-1175|United States.Pennsylvania
Epeorus vitreus|SWRC-Evitreus WLAB 07|United States.Pennsylvania
Epeorus vitreus|08INHSE-0190|United States.Tennessee
Epeorus vitreus|09NBMAY-0154|Canada.New Brunswick
Epeorus vitreus|09NBMAY-0144|Canada.New Brunswick
Epeorus vitreus|09NBMAY-0482|Canada.New Brunswick
Epeorus vitreus|09NBMAY-0448|Canada.New Brunswick
Epeorus vitreus|09CPMAY-066|Canada.Nova Scotia
Epeorus vitreus|09NBMAY-0406|Canada.New Brunswick
Epeorus vitreus|09NBMAY-0668|Canada.New Brunswick
Epeorus vitreus|SWRC-Evitreus WLAB 08|United States.Pennsylvania
Epeorus vitreus|09CPMAY-139|Canada.Nova Scotia
Epeorus vitreus|09CPMAY-095|Canada.Nova Scotia
Epeorus vitreus|09CPMAY-067|Canada.Nova Scotia
Epeorus vitreus|09NBMAY-0450|Canada.New Brunswick
Epeorus vitreus|09NBMAY-0405|Canada.New Brunswick
Epeorus vitreus|10HIMXD-0158|United States.Georgia
Epeorus vitreus|HIEPT-1123|United States.North Carolina
Epeorus vitreus|10HIMXD-0001|United States.North Carolina
Epeorus vitreus|09NBMAY-0147|Canada.New Brunswick
Epeorus vitreus|10HIMXD-0155|United States.Georgia
Epeorus vitreus|10HIMXD-0003|United States.North Carolina

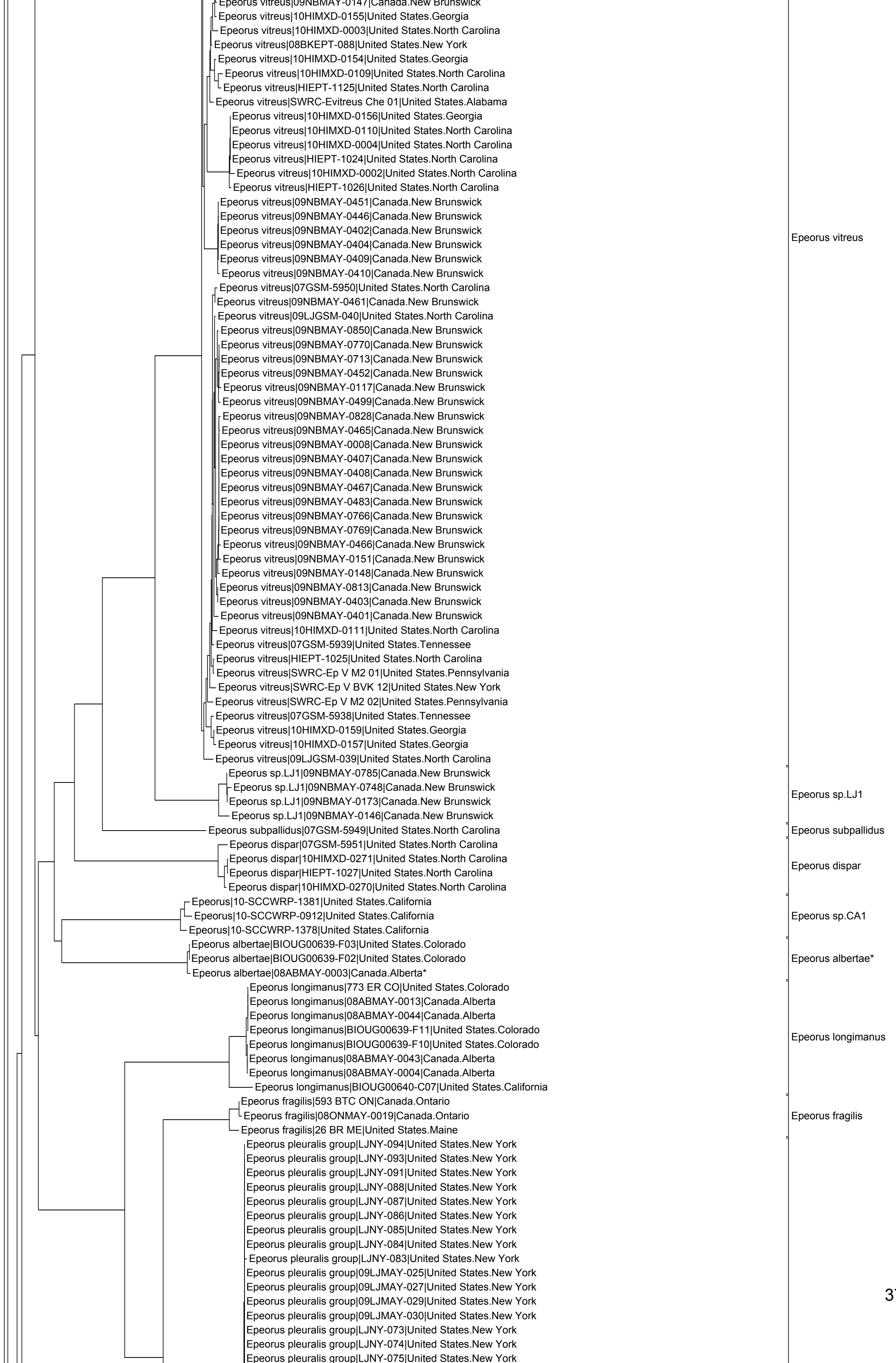

Epeorus vitreus|09NBMAY-0147|Canada.New Brunswick
Epeorus vitreus|10HIMXD-0155|United States.Georgia
Epeorus vitreus|10HIMXD-0003|United States.North Carolina
Epeorus vitreus|08BKEPT-088|United States.New York
Epeorus vitreus|10HIMXD-0154|United States.Georgia
Epeorus vitreus|10HIMXD-0109|United States.North Carolina
Epeorus vitreus|HIEPT-1125|United States.North Carolina
Epeorus vitreus|SWRC-Evitreus Che 01|United States.Alabama
Epeorus vitreus|10HIMXD-0156|United States.Georgia
Epeorus vitreus|10HIMXD-0110|United States.North Carolina
Epeorus vitreus|10HIMXD-0004|United States.North Carolina
Epeorus vitreus|HIEPT-1024|United States.North Carolina
Epeorus vitreus|10HIMXD-0002|United States.North Carolina
Epeorus vitreus|HIEPT-1026|United States.North Carolina
Epeorus vitreus|09NBMAY-0451|Canada.New Brunswick
Epeorus vitreus|09NBMAY-0446|Canada.New Brunswick
Epeorus vitreus|09NBMAY-0402|Canada.New Brunswick
Epeorus vitreus|09NBMAY-0404|Canada.New Brunswick
Epeorus vitreus|09NBMAY-0409|Canada.New Brunswick
Epeorus vitreus|09NBMAY-0410|Canada.New Brunswick
Epeorus vitreus|07GSM-5950|United States.North Carolina
Epeorus vitreus|09NBMAY-0461|Canada.New Brunswick
Epeorus vitreus|09LJGSM-040|United States.North Carolina
Epeorus vitreus|09NBMAY-0850|Canada.New Brunswick
Epeorus vitreus|09NBMAY-0770|Canada.New Brunswick
Epeorus vitreus|09NBMAY-0713|Canada.New Brunswick
Epeorus vitreus|09NBMAY-0452|Canada.New Brunswick
Epeorus vitreus|09NBMAY-0117|Canada.New Brunswick
Epeorus vitreus|09NBMAY-0499|Canada.New Brunswick
Epeorus vitreus|09NBMAY-0828|Canada.New Brunswick
Epeorus vitreus|09NBMAY-0465|Canada.New Brunswick
Epeorus vitreus|09NBMAY-0008|Canada.New Brunswick
Epeorus vitreus|09NBMAY-0407|Canada.New Brunswick
Epeorus vitreus|09NBMAY-0408|Canada.New Brunswick
Epeorus vitreus|09NBMAY-0467|Canada.New Brunswick
Epeorus vitreus|09NBMAY-0483|Canada.New Brunswick
Epeorus vitreus|09NBMAY-0766|Canada.New Brunswick
Epeorus vitreus|09NBMAY-0769|Canada.New Brunswick
Epeorus vitreus|09NBMAY-0466|Canada.New Brunswick
Epeorus vitreus|09NBMAY-0151|Canada.New Brunswick
Epeorus vitreus|09NBMAY-0148|Canada.New Brunswick
Epeorus vitreus|09NBMAY-0813|Canada.New Brunswick
Epeorus vitreus|09NBMAY-0403|Canada.New Brunswick
Epeorus vitreus|09NBMAY-0401|Canada.New Brunswick
Epeorus vitreus|10HIMXD-0111|United States.North Carolina
Epeorus vitreus|07GSM-5939|United States.Tennessee
Epeorus vitreus|HIEPT-1025|United States.North Carolina
Epeorus vitreus|SWRC-Ep V M2 01|United States.Pennsylvania
Epeorus vitreus|SWRC-Ep V BVK 12|United States.New York
Epeorus vitreus|SWRC-Ep V M2 02|United States.Pennsylvania
Epeorus vitreus|07GSM-5938|United States.Tennessee
Epeorus vitreus|10HIMXD-0159|United States.Georgia
Epeorus vitreus|10HIMXD-0157|United States.Georgia
Epeorus vitreus|09LJGSM-039|United States.North Carolina
Epeorus vitreus
Epeorus sp.LJ1|09NBMAY-0785|Canada.New Brunswick
Epeorus sp.LJ1|09NBMAY-0748|Canada.New Brunswick
Epeorus sp.LJ1|09NBMAY-0173|Canada.New Brunswick
Epeorus sp.LJ1|09NBMAY-0146|Canada.New Brunswick
Epeorus sp.LJ1
Epeorus subpallidus|07GSM-5949|United States.North Carolina
Epeorus subpallidus
Epeorus dispar|07GSM-5951|United States.North Carolina
Epeorus dispar|10HIMXD-0271|United States.North Carolina
Epeorus dispar|HIEPT-1027|United States.North Carolina
Epeorus dispar|10HIMXD-0270|United States.North Carolina
Epeorus dispar
Epeorus|10-SCCWRP-1381|United States.California
Epeorus|10-SCCWRP-0912|United States.California
Epeorus|10-SCCWRP-1378|United States.California
Epeorus sp.CA1
Epeorus albertae|BIOUG00639-F03|United States.Colorado
Epeorus albertae|BIOUG00639-F02|United States.Colorado
Epeorus albertae|08ABMAY-0003|Canada.Alberta*
Epeorus albertae*
Epeorus longimanus|773 ER CO|United States.Colorado
Epeorus longimanus|08ABMAY-0013|Canada.Alberta
Epeorus longimanus|08ABMAY-0044|Canada.Alberta
Epeorus longimanus|BIOUG00639-F11|United States.Colorado
Epeorus longimanus|BIOUG00639-F10|United States.Colorado
Epeorus longimanus|08ABMAY-0043|Canada.Alberta
Epeorus longimanus|08ABMAY-0004|Canada.Alberta
Epeorus longimanus|BIOUG00640-C07|United States.California
Epeorus longimanus
Epeorus fragilis|593 BTC ON|Canada.Ontario
Epeorus fragilis|08ONMAY-0019|Canada.Ontario
Epeorus fragilis|26 BR ME|United States.Maine
Epeorus fragilis
Epeorus pleuralis group|LJNY-094|United States.New York
Epeorus pleuralis group|LJNY-093|United States.New York
Epeorus pleuralis group|LJNY-091|United States.New York
Epeorus pleuralis group|LJNY-088|United States.New York
Epeorus pleuralis group|LJNY-087|United States.New York
Epeorus pleuralis group|LJNY-086|United States.New York
Epeorus pleuralis group|LJNY-085|United States.New York
Epeorus pleuralis group|LJNY-084|United States.New York
Epeorus pleuralis group|LJNY-083|United States.New York
Epeorus pleuralis group|09LJMAY-025|United States.New York
Epeorus pleuralis group|09LJMAY-027|United States.New York
Epeorus pleuralis group|09LJMAY-029|United States.New York
Epeorus pleuralis group|09LJMAY-030|United States.New York
Epeorus pleuralis group|LJNY-073|United States.New York
Epeorus pleuralis group|LJNY-074|United States.New York
Epeorus pleuralis group|LJNY-075|United States.New York

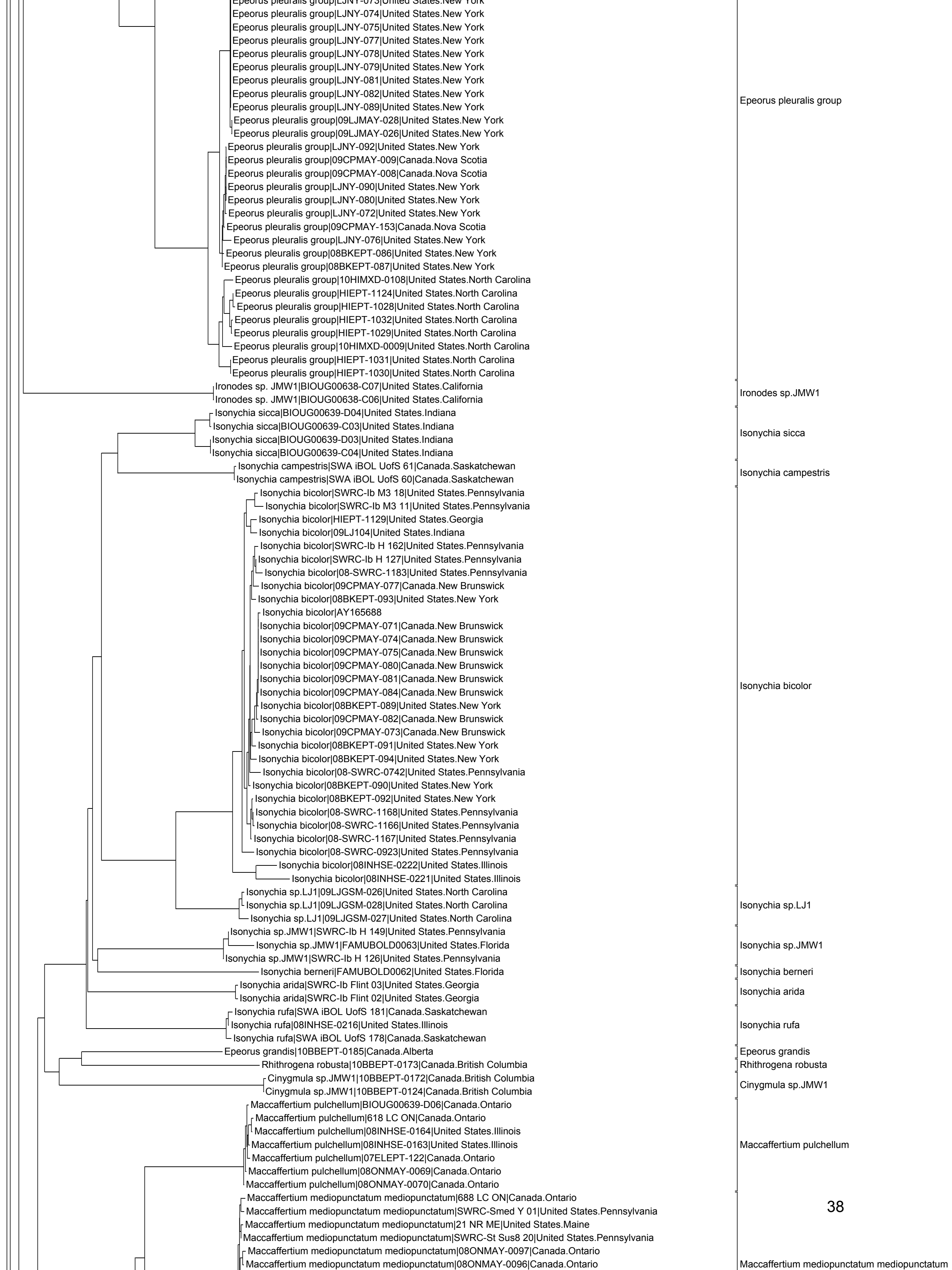
Epeorus pleuralis group|LJNY-074|United States.New York
Epeorus pleuralis group|LJNY-075|United States.New York
Epeorus pleuralis group|LJNY-077|United States.New York
Epeorus pleuralis group|LJNY-078|United States.New York
Epeorus pleuralis group|LJNY-079|United States.New York
Epeorus pleuralis group|LJNY-081|United States.New York
Epeorus pleuralis group|LJNY-082|United States.New York
Epeorus pleuralis group|LJNY-089|United States.New York
Epeorus pleuralis group|09LJMAY-028|United States.New York
Epeorus pleuralis group|09LJMAY-026|United States.New York
Epeorus pleuralis group|LJNY-092|United States.New York
Epeorus pleuralis group|09CPMAY-009|Canada.Nova Scotia
Epeorus pleuralis group|09CPMAY-008|Canada.Nova Scotia
Epeorus pleuralis group|LJNY-090|United States.New York
Epeorus pleuralis group|LJNY-080|United States.New York
Epeorus pleuralis group|LJNY-072|United States.New York
Epeorus pleuralis group|09CPMAY-153|Canada.Nova Scotia
Epeorus pleuralis group|LJNY-076|United States.New York
Epeorus pleuralis group|08BKEPT-086|United States.New York
Epeorus pleuralis group|08BKEPT-087|United States.New York
Epeorus pleuralis group|10HIMXD-0108|United States.North Carolina
Epeorus pleuralis group|HIEPT-1124|United States.North Carolina
Epeorus pleuralis group|HIEPT-1028|United States.North Carolina
Epeorus pleuralis group|HIEPT-1032|United States.North Carolina
Epeorus pleuralis group|HIEPT-1029|United States.North Carolina
Epeorus pleuralis group|10HIMXD-0009|United States.North Carolina
Epeorus pleuralis group|HIEPT-1031|United States.North Carolina
Epeorus pleuralis group|HIEPT-1030|United States.North Carolina
Ironodes sp. JMW1|BIOUG00638-C07|United States.California
Ironodes sp. JMW1|BIOUG00638-C06|United States.California
Isonychia sicca|BIOUG00639-D04|United States.Indiana
Isonychia sicca|BIOUG00639-C03|United States.Indiana
Isonychia sicca|BIOUG00639-D03|United States.Indiana
Isonychia sicca|BIOUG00639-C04|United States.Indiana
Isonychia campestris|SWA iBOL UofS 61|Canada.Saskatchewan
Isonychia campestris|SWA iBOL UofS 60|Canada.Saskatchewan
Isonychia bicolor|SWRC-Ib M3 18|United States.Pennsylvania
Isonychia bicolor|SWRC-Ib M3 11|United States.Pennsylvania
Isonychia bicolor|HIEPT-1129|United States.Georgia
Isonychia bicolor|09LJ104|United States.Indiana
Isonychia bicolor|SWRC-Ib H 162|United States.Pennsylvania
Isonychia bicolor|SWRC-Ib H 127|United States.Pennsylvania
Isonychia bicolor|08-SWRC-1183|United States.Pennsylvania
Isonychia bicolor|09CPMAY-077|Canada.New Brunswick
Isonychia bicolor|08BKEPT-093|United States.New York
Isonychia bicolor|AY165688
Isonychia bicolor|09CPMAY-071|Canada.New Brunswick
Isonychia bicolor|09CPMAY-074|Canada.New Brunswick
Isonychia bicolor|09CPMAY-075|Canada.New Brunswick
Isonychia bicolor|09CPMAY-080|Canada.New Brunswick
Isonychia bicolor|09CPMAY-081|Canada.New Brunswick
Isonychia bicolor|09CPMAY-084|Canada.New Brunswick
Isonychia bicolor|08BKEPT-089|United States.New York
Isonychia bicolor|09CPMAY-082|Canada.New Brunswick
Isonychia bicolor|09CPMAY-073|Canada.New Brunswick
Isonychia bicolor|08BKEPT-091|United States.New York
Isonychia bicolor|08BKEPT-094|United States.New York
Isonychia bicolor|08-SWRC-0742|United States.Pennsylvania
Isonychia bicolor|08BKEPT-090|United States.New York
Isonychia bicolor|08BKEPT-092|United States.New York
Isonychia bicolor|08-SWRC-1168|United States.Pennsylvania
Isonychia bicolor|08-SWRC-1166|United States.Pennsylvania
Isonychia bicolor|08-SWRC-1167|United States.Pennsylvania
Isonychia bicolor|08-SWRC-0923|United States.Pennsylvania
Isonychia bicolor|08INHSE-0222|United States.Illinois
Isonychia bicolor|08INHSE-0221|United States.Illinois
Isonychia sp.LJ1|09LJGSM-026|United States.North Carolina
Isonychia sp.LJ1|09LJGSM-028|United States.North Carolina
Isonychia sp.LJ1|09LJGSM-027|United States.North Carolina
Isonychia sp.JMW1|SWRC-Ib H 149|United States.Pennsylvania
Isonychia sp.JMW1|FAMUBOLD0063|United States.Florida
Isonychia sp.JMW1|SWRC-Ib H 126|United States.Pennsylvania
Isonychia berneri|FAMUBOLD0062|United States.Florida
Isonychia arida|SWRC-Ib Flint 03|United States.Georgia
Isonychia arida|SWRC-Ib Flint 02|United States.Georgia
Isonychia rufa|SWA iBOL UofS 181|Canada.Saskatchewan
Isonychia rufa|08INHSE-0216|United States.Illinois
Isonychia rufa|SWA iBOL UofS 178|Canada.Saskatchewan
Epeorus grandis|10BBEPT-0185|Canada.Alberta
Rhithrogena robusta|10BBEPT-0173|Canada.British Columbia
Cinygmula sp.JMW1|10BBEPT-0172|Canada.British Columbia
Cinygmula sp.JMW1|10BBEPT-0124|Canada.British Columbia
Maccaffertium pulchellum|BIOUG00639-D06|Canada.Ontario
Maccaffertium pulchellum|618 LC ON|Canada.Ontario
Maccaffertium pulchellum|08INHSE-0164|United States.Illinois
Maccaffertium pulchellum|08INHSE-0163|United States.Illinois
Maccaffertium pulchellum|07ELEPT-122|Canada.Ontario
Maccaffertium pulchellum|08ONMAY-0069|Canada.Ontario
Maccaffertium pulchellum|08ONMAY-0070|Canada.Ontario
Maccaffertium mediopunctatum mediopunctatum|688 LC ON|Canada.Ontario
Maccaffertium mediopunctatum mediopunctatum|SWRC-Smed Y 01|United States.Pennsylvania
Maccaffertium mediopunctatum mediopunctatum|21 NR ME|United States.Maine
Maccaffertium mediopunctatum mediopunctatum|SWRC-St Sus8 20|United States.Pennsylvania
Maccaffertium mediopunctatum mediopunctatum|08ONMAY-0097|Canada.Ontario
Maccaffertium mediopunctatum mediopunctatum|08ONMAY-0096|Canada.Ontario
Epeorus pleuralis group
Ironodes sp.JMW1
Isonychia sicca
Isonychia campestris
Isonychia bicolor
Isonychia sp.LJ1
Isonychia sp.JMW1
Isonychia berneri
Isonychia arida
Isonychia rufa
Epeorus grandis
Rhithrogena robusta
Cinygmula sp.JMW1
Maccaffertium pulchellum
Maccaffertium mediopunctatum mediopunctatum

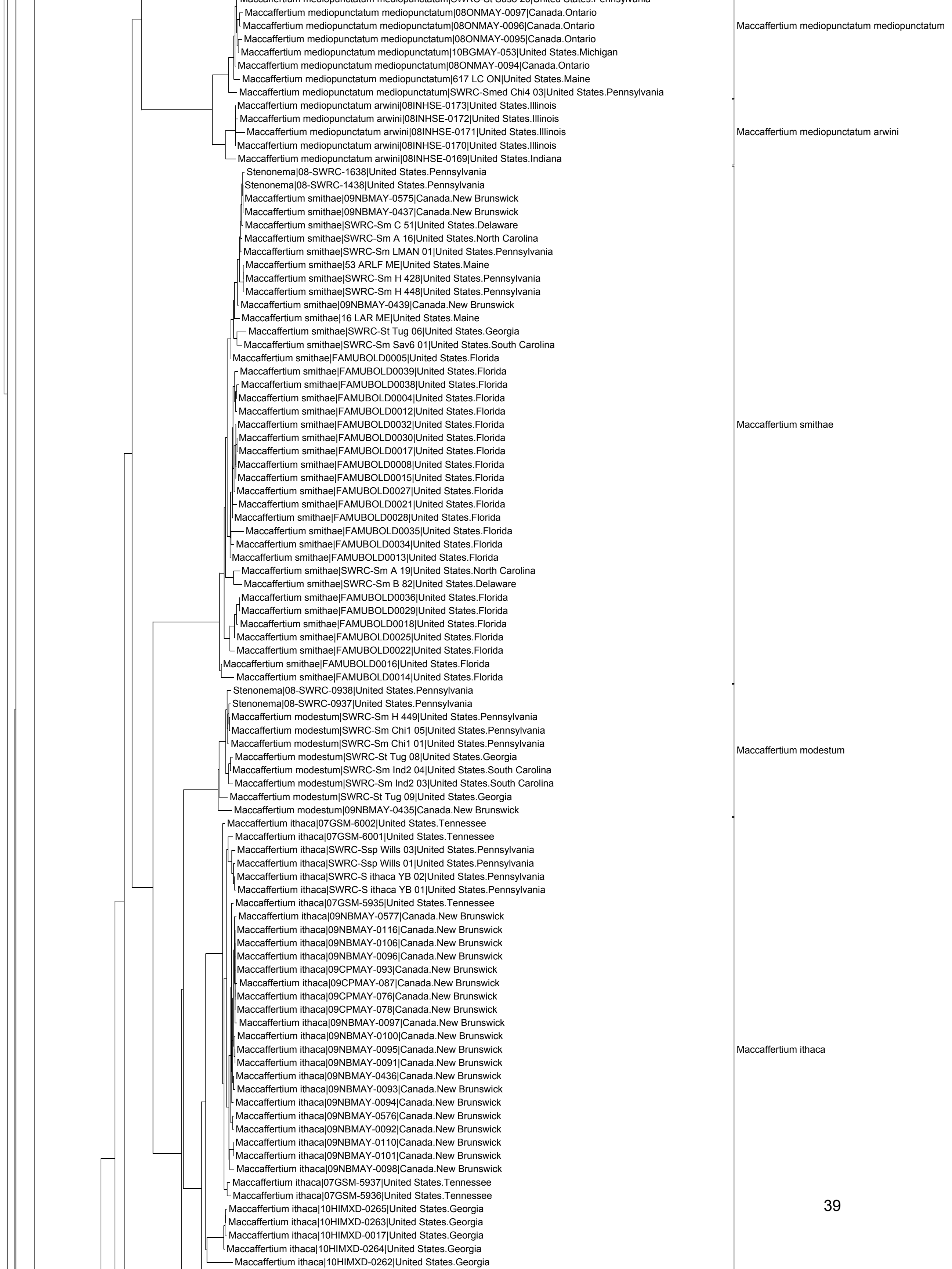
Maccaffertium mediopunctatum mediopunctatum|08ONMAY-0097|Canada.Ontario
Maccaffertium mediopunctatum mediopunctatum|08ONMAY-0096|Canada.Ontario
Maccaffertium mediopunctatum mediopunctatum|08ONMAY-0095|Canada.Ontario
Maccaffertium mediopunctatum mediopunctatum|10BGMAY-053|United States.Michigan
Maccaffertium mediopunctatum mediopunctatum|08ONMAY-0094|Canada.Ontario
Maccaffertium mediopunctatum mediopunctatum|617 LC ON|United States.Maine
Maccaffertium mediopunctatum mediopunctatum|SWRC-Smed Chi4 03|United States.Pennsylvania
Maccaffertium mediopunctatum arwini|08INHSE-0173|United States.Illinois
Maccaffertium mediopunctatum arwini|08INHSE-0172|United States.Illinois
Maccaffertium mediopunctatum arwini|08INHSE-0171|United States.Illinois
Maccaffertium mediopunctatum arwini|08INHSE-0170|United States.Illinois
Maccaffertium mediopunctatum arwini|08INHSE-0169|United States.Indiana
Stenonema|08-SWRC-1638|United States.Pennsylvania
Stenonema|08-SWRC-1438|United States.Pennsylvania
Maccaffertium smithae|09NBMAY-0575|Canada.New Brunswick
Maccaffertium smithae|09NBMAY-0437|Canada.New Brunswick
Maccaffertium smithae|SWRC-Sm C 51|United States.Delaware
Maccaffertium smithae|SWRC-Sm A 16|United States.North Carolina
Maccaffertium smithae|SWRC-Sm LMAN 01|United States.Pennsylvania
Maccaffertium smithae|53 ARLF ME|United States.Maine
Maccaffertium smithae|SWRC-Sm H 428|United States.Pennsylvania
Maccaffertium smithae|SWRC-Sm H 448|United States.Pennsylvania
Maccaffertium smithae|09NBMAY-0439|Canada.New Brunswick
Maccaffertium smithae|16 LAR ME|United States.Maine
Maccaffertium smithae|SWRC-St Tug 06|United States.Georgia
Maccaffertium smithae|SWRC-Sm Sav6 01|United States.South Carolina
Maccaffertium smithae|FAMUBOLD0005|United States.Florida
Maccaffertium smithae|FAMUBOLD0039|United States.Florida
Maccaffertium smithae|FAMUBOLD0038|United States.Florida
Maccaffertium smithae|FAMUBOLD0004|United States.Florida
Maccaffertium smithae|FAMUBOLD0012|United States.Florida
Maccaffertium smithae|FAMUBOLD0032|United States.Florida
Maccaffertium smithae|FAMUBOLD0030|United States.Florida
Maccaffertium smithae|FAMUBOLD0017|United States.Florida
Maccaffertium smithae|FAMUBOLD0008|United States.Florida
Maccaffertium smithae|FAMUBOLD0015|United States.Florida
Maccaffertium smithae|FAMUBOLD0027|United States.Florida
Maccaffertium smithae|FAMUBOLD0021|United States.Florida
Maccaffertium smithae|FAMUBOLD0028|United States.Florida
Maccaffertium smithae|FAMUBOLD0035|United States.Florida
Maccaffertium smithae|FAMUBOLD0034|United States.Florida
Maccaffertium smithae|FAMUBOLD0013|United States.Florida
Maccaffertium smithae|SWRC-Sm A 19|United States.North Carolina
Maccaffertium smithae|SWRC-Sm B 82|United States.Delaware
Maccaffertium smithae|FAMUBOLD0036|United States.Florida
Maccaffertium smithae|FAMUBOLD0029|United States.Florida
Maccaffertium smithae|FAMUBOLD0018|United States.Florida
Maccaffertium smithae|FAMUBOLD0025|United States.Florida
Maccaffertium smithae|FAMUBOLD0022|United States.Florida
Maccaffertium smithae|FAMUBOLD0016|United States.Florida
Maccaffertium smithae|FAMUBOLD0014|United States.Florida
Stenonema|08-SWRC-0938|United States.Pennsylvania
Stenonema|08-SWRC-0937|United States.Pennsylvania
Maccaffertium modestum|SWRC-Sm H 449|United States.Pennsylvania
Maccaffertium modestum|SWRC-Sm Chi1 05|United States.Pennsylvania
Maccaffertium modestum|SWRC-Sm Chi1 01|United States.Pennsylvania
Maccaffertium modestum|SWRC-St Tug 08|United States.Georgia
Maccaffertium modestum|SWRC-Sm Ind2 04|United States.South Carolina
Maccaffertium modestum|SWRC-Sm Ind2 03|United States.South Carolina
Maccaffertium modestum|SWRC-St Tug 09|United States.Georgia
Maccaffertium modestum|09NBMAY-0435|Canada.New Brunswick
Maccaffertium ithaca|07GSM-6002|United States.Tennessee
Maccaffertium ithaca|07GSM-6001|United States.Tennessee
Maccaffertium ithaca|SWRC-Ssp Wills 03|United States.Pennsylvania
Maccaffertium ithaca|SWRC-Ssp Wills 01|United States.Pennsylvania
Maccaffertium ithaca|SWRC-S ithaca YB 02|United States.Pennsylvania
Maccaffertium ithaca|SWRC-S ithaca YB 01|United States.Pennsylvania
Maccaffertium ithaca|07GSM-5935|United States.Tennessee
Maccaffertium ithaca|09NBMAY-0577|Canada.New Brunswick
Maccaffertium ithaca|09NBMAY-0116|Canada.New Brunswick
Maccaffertium ithaca|09NBMAY-0106|Canada.New Brunswick
Maccaffertium ithaca|09NBMAY-0096|Canada.New Brunswick
Maccaffertium ithaca|09CPMAY-093|Canada.New Brunswick
Maccaffertium ithaca|09CPMAY-087|Canada.New Brunswick
Maccaffertium ithaca|09CPMAY-076|Canada.New Brunswick
Maccaffertium ithaca|09CPMAY-078|Canada.New Brunswick
Maccaffertium ithaca|09NBMAY-0097|Canada.New Brunswick
Maccaffertium ithaca|09NBMAY-0100|Canada.New Brunswick
Maccaffertium ithaca|09NBMAY-0095|Canada.New Brunswick
Maccaffertium ithaca|09NBMAY-0091|Canada.New Brunswick
Maccaffertium ithaca|09NBMAY-0436|Canada.New Brunswick
Maccaffertium ithaca|09NBMAY-0093|Canada.New Brunswick
Maccaffertium ithaca|09NBMAY-0094|Canada.New Brunswick
Maccaffertium ithaca|09NBMAY-0576|Canada.New Brunswick
Maccaffertium ithaca|09NBMAY-0092|Canada.New Brunswick
Maccaffertium ithaca|09NBMAY-0110|Canada.New Brunswick
Maccaffertium ithaca|09NBMAY-0101|Canada.New Brunswick
Maccaffertium ithaca|09NBMAY-0098|Canada.New Brunswick
Maccaffertium ithaca|07GSM-5937|United States.Tennessee
Maccaffertium ithaca|07GSM-5936|United States.Tennessee
Maccaffertium ithaca|10HIMXD-0265|United States.Georgia
Maccaffertium ithaca|10HIMXD-0263|United States.Georgia
Maccaffertium ithaca|10HIMXD-0017|United States.Georgia
Maccaffertium ithaca|10HIMXD-0264|United States.Georgia
Maccaffertium ithaca|10HIMXD-0262|United States.Georgia
Maccaffertium mediopunctatum mediopunctatum
Maccaffertium mediopunctatum arwini
Maccaffertium smithae
Maccaffertium modestum
Maccaffertium ithaca

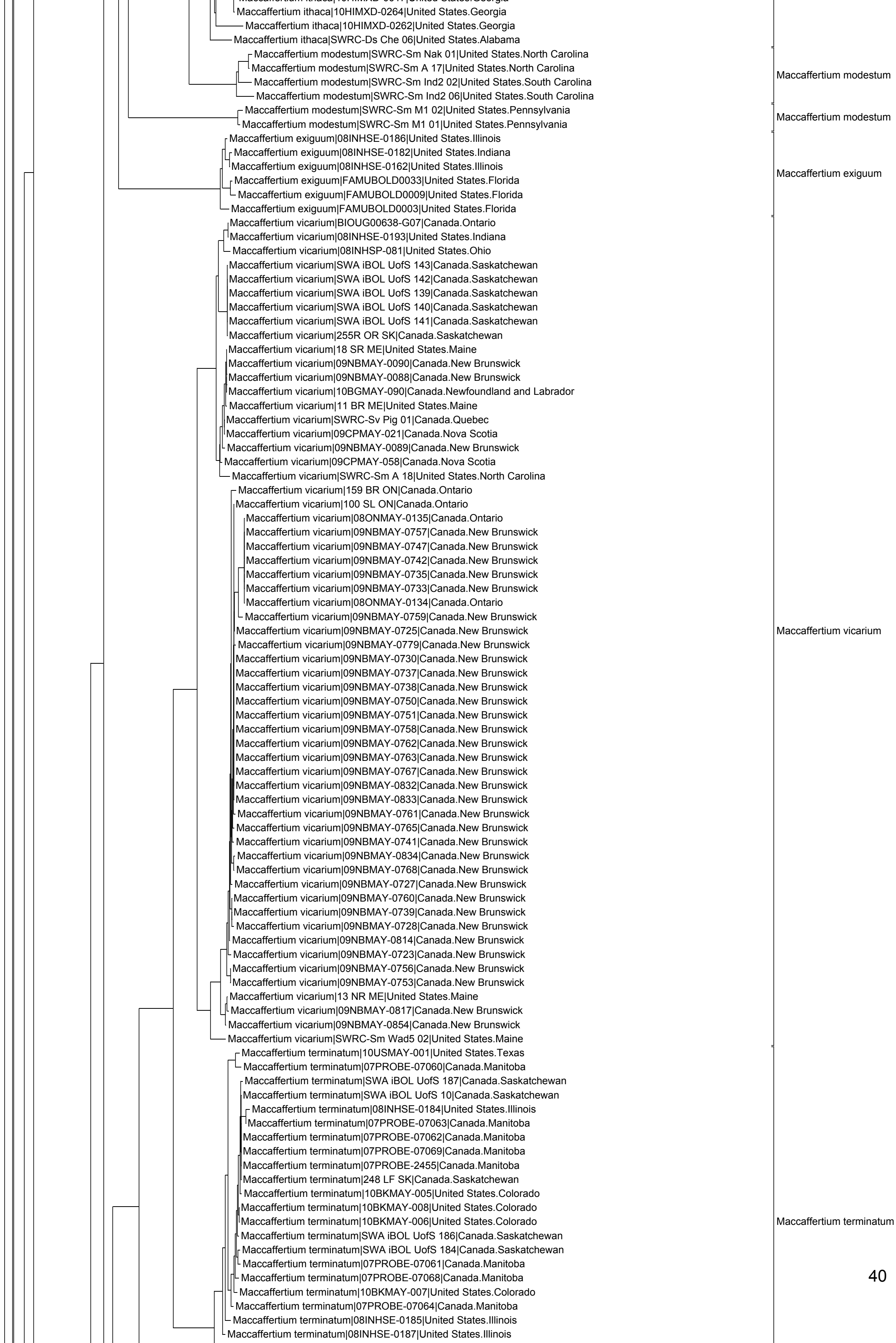
Maccaffertium ithaca|10HIMXD-0264|United States.Georgia
Maccaffertium ithaca|10HIMXD-0262|United States.Georgia
Maccaffertium ithaca|SWRC-Ds Che 06|United States.Alabama
Maccaffertium modestum|SWRC-Sm Nak 01|United States.North Carolina
Maccaffertium modestum|SWRC-Sm A 17|United States.North Carolina
Maccaffertium modestum|SWRC-Sm Ind2 02|United States.South Carolina
Maccaffertium modestum|SWRC-Sm Ind2 06|United States.South Carolina
Maccaffertium modestum|SWRC-Sm M1 02|United States.Pennsylvania
Maccaffertium modestum|SWRC-Sm M1 01|United States.Pennsylvania
Maccaffertium exiguum|08INHSE-0186|United States.Illinois
Maccaffertium exiguum|08INHSE-0182|United States.Indiana
Maccaffertium exiguum|08INHSE-0162|United States.Illinois
Maccaffertium exiguum|FAMUBOLD0033|United States.Florida
Maccaffertium exiguum|FAMUBOLD0009|United States.Florida
Maccaffertium exiguum|FAMUBOLD0003|United States.Florida
Maccaffertium vicarium|BIOUG00638-G07|Canada.Ontario
Maccaffertium vicarium|08INHSE-0193|United States.Indiana
Maccaffertium vicarium|08INHSP-081|United States.Ohio
Maccaffertium vicarium|SWA iBOL UofS 143|Canada.Saskatchewan
Maccaffertium vicarium|SWA iBOL UofS 142|Canada.Saskatchewan
Maccaffertium vicarium|SWA iBOL UofS 139|Canada.Saskatchewan
Maccaffertium vicarium|SWA iBOL UofS 140|Canada.Saskatchewan
Maccaffertium vicarium|SWA iBOL UofS 141|Canada.Saskatchewan
Maccaffertium vicarium|255R OR SK|Canada.Saskatchewan
Maccaffertium vicarium|18 SR ME|United States.Maine
Maccaffertium vicarium|09NBMAY-0090|Canada.New Brunswick
Maccaffertium vicarium|09NBMAY-0088|Canada.New Brunswick
Maccaffertium vicarium|10BGMAY-090|Canada.Newfoundland and Labrador
Maccaffertium vicarium|11 BR ME|United States.Maine
Maccaffertium vicarium|SWRC-Sv Pig 01|Canada.Quebec
Maccaffertium vicarium|09CPMAY-021|Canada.Nova Scotia
Maccaffertium vicarium|09NBMAY-0089|Canada.New Brunswick
Maccaffertium vicarium|09CPMAY-058|Canada.Nova Scotia
Maccaffertium vicarium|SWRC-Sm A 18|United States.North Carolina
Maccaffertium vicarium|159 BR ON|Canada.Ontario
Maccaffertium vicarium|100 SL ON|Canada.Ontario
Maccaffertium vicarium|08ONMAY-0135|Canada.Ontario
Maccaffertium vicarium|09NBMAY-0757|Canada.New Brunswick
Maccaffertium vicarium|09NBMAY-0747|Canada.New Brunswick
Maccaffertium vicarium|09NBMAY-0742|Canada.New Brunswick
Maccaffertium vicarium|09NBMAY-0735|Canada.New Brunswick
Maccaffertium vicarium|09NBMAY-0733|Canada.New Brunswick
Maccaffertium vicarium|08ONMAY-0134|Canada.Ontario
Maccaffertium vicarium|09NBMAY-0759|Canada.New Brunswick
Maccaffertium vicarium|09NBMAY-0725|Canada.New Brunswick
Maccaffertium vicarium|09NBMAY-0779|Canada.New Brunswick
Maccaffertium vicarium|09NBMAY-0730|Canada.New Brunswick
Maccaffertium vicarium|09NBMAY-0737|Canada.New Brunswick
Maccaffertium vicarium|09NBMAY-0738|Canada.New Brunswick
Maccaffertium vicarium|09NBMAY-0750|Canada.New Brunswick
Maccaffertium vicarium|09NBMAY-0751|Canada.New Brunswick
Maccaffertium vicarium|09NBMAY-0758|Canada.New Brunswick
Maccaffertium vicarium|09NBMAY-0762|Canada.New Brunswick
Maccaffertium vicarium|09NBMAY-0763|Canada.New Brunswick
Maccaffertium vicarium|09NBMAY-0767|Canada.New Brunswick
Maccaffertium vicarium|09NBMAY-0832|Canada.New Brunswick
Maccaffertium vicarium|09NBMAY-0833|Canada.New Brunswick
Maccaffertium vicarium|09NBMAY-0761|Canada.New Brunswick
Maccaffertium vicarium|09NBMAY-0765|Canada.New Brunswick
Maccaffertium vicarium|09NBMAY-0741|Canada.New Brunswick
Maccaffertium vicarium|09NBMAY-0834|Canada.New Brunswick
Maccaffertium vicarium|09NBMAY-0768|Canada.New Brunswick
Maccaffertium vicarium|09NBMAY-0727|Canada.New Brunswick
Maccaffertium vicarium|09NBMAY-0760|Canada.New Brunswick
Maccaffertium vicarium|09NBMAY-0739|Canada.New Brunswick
Maccaffertium vicarium|09NBMAY-0728|Canada.New Brunswick
Maccaffertium vicarium|09NBMAY-0814|Canada.New Brunswick
Maccaffertium vicarium|09NBMAY-0723|Canada.New Brunswick
Maccaffertium vicarium|09NBMAY-0756|Canada.New Brunswick
Maccaffertium vicarium|09NBMAY-0753|Canada.New Brunswick
Maccaffertium vicarium|13 NR ME|United States.Maine
Maccaffertium vicarium|09NBMAY-0817|Canada.New Brunswick
Maccaffertium vicarium|09NBMAY-0854|Canada.New Brunswick
Maccaffertium vicarium|SWRC-Sm Wad5 02|United States.Maine
Maccaffertium terminatum|10USMAY-001|United States.Texas
Maccaffertium terminatum|07PROBE-07060|Canada.Manitoba
Maccaffertium terminatum|SWA iBOL UofS 187|Canada.Saskatchewan
Maccaffertium terminatum|SWA iBOL UofS 10|Canada.Saskatchewan
Maccaffertium terminatum|08INHSE-0184|United States.Illinois
Maccaffertium terminatum|07PROBE-07063|Canada.Manitoba
Maccaffertium terminatum|07PROBE-07062|Canada.Manitoba
Maccaffertium terminatum|07PROBE-07069|Canada.Manitoba
Maccaffertium terminatum|07PROBE-2455|Canada.Manitoba
Maccaffertium terminatum|248 LF SK|Canada.Saskatchewan
Maccaffertium terminatum|10BKMAY-005|United States.Colorado
Maccaffertium terminatum|10BKMAY-008|United States.Colorado
Maccaffertium terminatum|10BKMAY-006|United States.Colorado
Maccaffertium terminatum|SWA iBOL UofS 186|Canada.Saskatchewan
Maccaffertium terminatum|SWA iBOL UofS 184|Canada.Saskatchewan
Maccaffertium terminatum|07PROBE-07061|Canada.Manitoba
Maccaffertium terminatum|07PROBE-07068|Canada.Manitoba
Maccaffertium terminatum|10BKMAY-007|United States.Colorado
Maccaffertium terminatum|07PROBE-07064|Canada.Manitoba
Maccaffertium terminatum|08INHSE-0185|United States.Illinois
Maccaffertium terminatum|08INHSE-0187|United States.Illinois
Maccaffertium modestum
Maccaffertium modestum
Maccaffertium exiguum
Maccaffertium vicarium
Maccaffertium terminatum

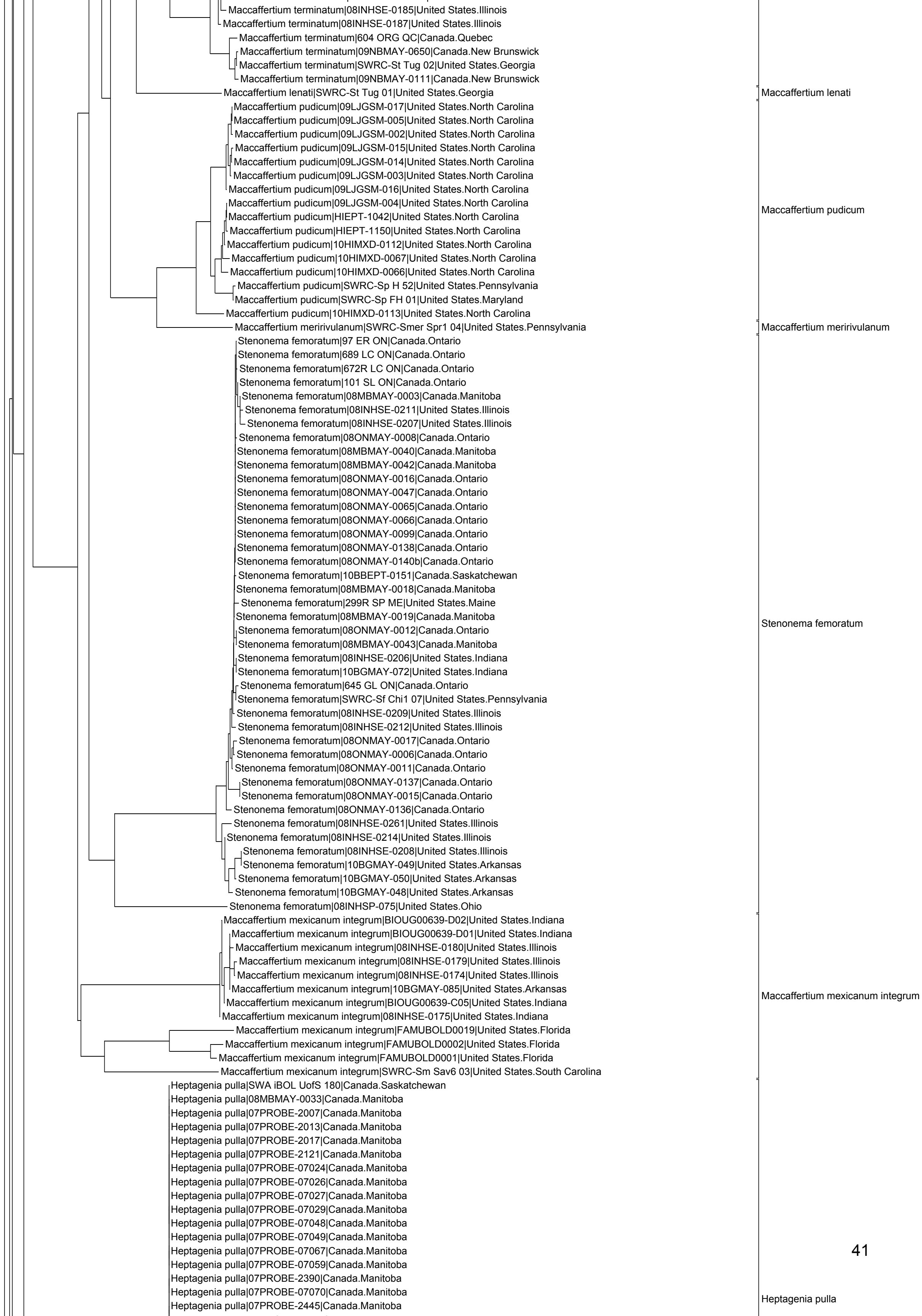

Maccaffertium terminatum|08INHSE-0185|United States.Illinois
Maccaffertium terminatum|08INHSE-0187|United States.Illinois
Maccaffertium terminatum|604 ORG QC|Canada.Quebec
Maccaffertium terminatum|09NBMAY-0650|Canada.New Brunswick
Maccaffertium terminatum|SWRC-St Tug 02|United States.Georgia
Maccaffertium terminatum|09NBMAY-0111|Canada.New Brunswick
Maccaffertium lenati|SWRC-St Tug 01|United States.Georgia
Maccaffertium lenati
Maccaffertium pudicum|09LJGSM-017|United States.North Carolina
Maccaffertium pudicum|09LJGSM-005|United States.North Carolina
Maccaffertium pudicum|09LJGSM-002|United States.North Carolina
Maccaffertium pudicum|09LJGSM-015|United States.North Carolina
Maccaffertium pudicum|09LJGSM-014|United States.North Carolina
Maccaffertium pudicum|09LJGSM-003|United States.North Carolina
Maccaffertium pudicum|09LJGSM-016|United States.North Carolina
Maccaffertium pudicum|09LJGSM-004|United States.North Carolina
Maccaffertium pudicum|HIEPT-1042|United States.North Carolina
Maccaffertium pudicum|HIEPT-1150|United States.North Carolina
Maccaffertium pudicum|10HIMXD-0112|United States.North Carolina
Maccaffertium pudicum|10HIMXD-0067|United States.North Carolina
Maccaffertium pudicum|10HIMXD-0066|United States.North Carolina
Maccaffertium pudicum|SWRC-Sp H 52|United States.Pennsylvania
Maccaffertium pudicum|SWRC-Sp FH 01|United States.Maryland
Maccaffertium pudicum|10HIMXD-0113|United States.North Carolina
Maccaffertium pudicum
Maccaffertium meririvulanum|SWRC-Smer Spr1 04|United States.Pennsylvania
Maccaffertium meririvulanum
Stenonema femoratum|97 ER ON|Canada.Ontario
Stenonema femoratum|689 LC ON|Canada.Ontario
Stenonema femoratum|672R LC ON|Canada.Ontario
Stenonema femoratum|101 SL ON|Canada.Ontario
Stenonema femoratum|08MBMAY-0003|Canada.Manitoba
Stenonema femoratum|08INHSE-0211|United States.Illinois
Stenonema femoratum|08INHSE-0207|United States.Illinois
Stenonema femoratum|08ONMAY-0008|Canada.Ontario
Stenonema femoratum|08MBMAY-0040|Canada.Manitoba
Stenonema femoratum|08MBMAY-0042|Canada.Manitoba
Stenonema femoratum|08ONMAY-0016|Canada.Ontario
Stenonema femoratum|08ONMAY-0047|Canada.Ontario
Stenonema femoratum|08ONMAY-0065|Canada.Ontario
Stenonema femoratum|08ONMAY-0066|Canada.Ontario
Stenonema femoratum|08ONMAY-0099|Canada.Ontario
Stenonema femoratum|08ONMAY-0138|Canada.Ontario
Stenonema femoratum|08ONMAY-0140b|Canada.Ontario
Stenonema femoratum|10BBEPT-0151|Canada.Saskatchewan
Stenonema femoratum|08MBMAY-0018|Canada.Manitoba
Stenonema femoratum|299R SP ME|United States.Maine
Stenonema femoratum|08MBMAY-0019|Canada.Manitoba
Stenonema femoratum|08ONMAY-0012|Canada.Ontario
Stenonema femoratum|08MBMAY-0043|Canada.Manitoba
Stenonema femoratum|08INHSE-0206|United States.Indiana
Stenonema femoratum|10BGMAY-072|United States.Indiana
Stenonema femoratum|645 GL ON|Canada.Ontario
Stenonema femoratum|SWRC-Sf Chi1 07|United States.Pennsylvania
Stenonema femoratum|08INHSE-0209|United States.Illinois
Stenonema femoratum|08INHSE-0212|United States.Illinois
Stenonema femoratum|08ONMAY-0017|Canada.Ontario
Stenonema femoratum|08ONMAY-0006|Canada.Ontario
Stenonema femoratum|08ONMAY-0011|Canada.Ontario
Stenonema femoratum|08ONMAY-0137|Canada.Ontario
Stenonema femoratum|08ONMAY-0015|Canada.Ontario
Stenonema femoratum|08ONMAY-0136|Canada.Ontario
Stenonema femoratum|08INHSE-0261|United States.Illinois
Stenonema femoratum|08INHSE-0214|United States.Illinois
Stenonema femoratum|08INHSE-0208|United States.Illinois
Stenonema femoratum|10BGMAY-049|United States.Arkansas
Stenonema femoratum|10BGMAY-050|United States.Arkansas
Stenonema femoratum|10BGMAY-048|United States.Arkansas
Stenonema femoratum|08INHSP-075|United States.Ohio
Stenonema femoratum
Maccaffertium mexicanum integrum|BIOUG00639-D02|United States.Indiana
Maccaffertium mexicanum integrum|BIOUG00639-D01|United States.Indiana
Maccaffertium mexicanum integrum|08INHSE-0180|United States.Illinois
Maccaffertium mexicanum integrum|08INHSE-0179|United States.Illinois
Maccaffertium mexicanum integrum|08INHSE-0174|United States.Illinois
Maccaffertium mexicanum integrum|10BGMAY-085|United States.Arkansas
Maccaffertium mexicanum integrum|BIOUG00639-C05|United States.Indiana
Maccaffertium mexicanum integrum|08INHSE-0175|United States.Indiana
Maccaffertium mexicanum integrum|FAMUBOLD0019|United States.Florida
Maccaffertium mexicanum integrum|FAMUBOLD0002|United States.Florida
Maccaffertium mexicanum integrum|FAMUBOLD0001|United States.Florida
Maccaffertium mexicanum integrum|SWRC-Sm Sav6 03|United States.South Carolina
Maccaffertium mexicanum integrum
Heptagenia pulla|SWA iBOL UofS 180|Canada.Saskatchewan
Heptagenia pulla|08MBMAY-0033|Canada.Manitoba
Heptagenia pulla|07PROBE-2007|Canada.Manitoba
Heptagenia pulla|07PROBE-2013|Canada.Manitoba
Heptagenia pulla|07PROBE-2017|Canada.Manitoba
Heptagenia pulla|07PROBE-2121|Canada.Manitoba
Heptagenia pulla|07PROBE-07024|Canada.Manitoba
Heptagenia pulla|07PROBE-07026|Canada.Manitoba
Heptagenia pulla|07PROBE-07027|Canada.Manitoba
Heptagenia pulla|07PROBE-07029|Canada.Manitoba
Heptagenia pulla|07PROBE-07048|Canada.Manitoba
Heptagenia pulla|07PROBE-07049|Canada.Manitoba
Heptagenia pulla|07PROBE-07067|Canada.Manitoba
Heptagenia pulla|07PROBE-07059|Canada.Manitoba
Heptagenia pulla|07PROBE-2390|Canada.Manitoba
Heptagenia pulla|07PROBE-07070|Canada.Manitoba
Heptagenia pulla|07PROBE-2445|Canada.Manitoba
Heptagenia pulla

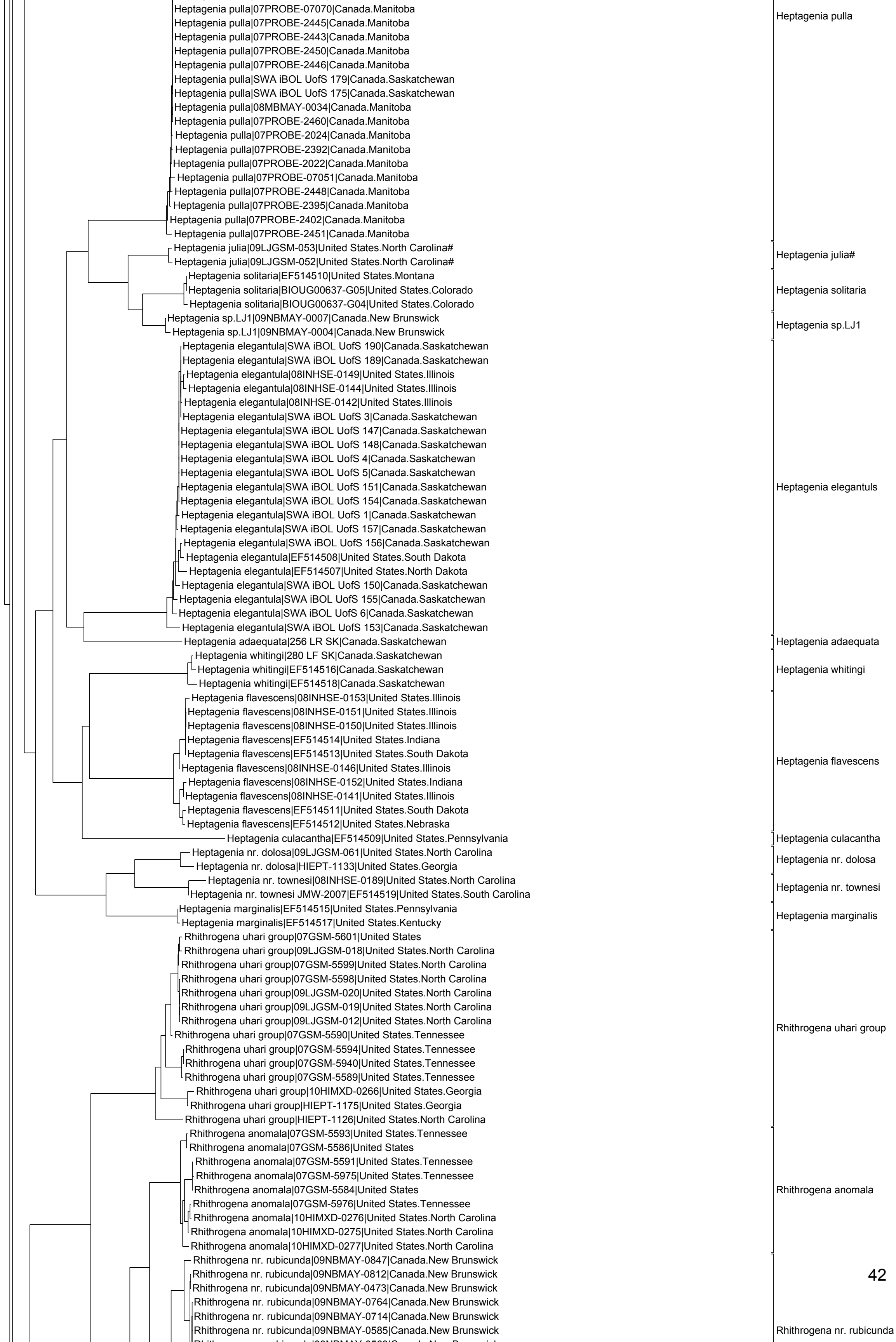

Heptagenia pulla|07PROBE-07070|Canada.Manitoba
Heptagenia pulla|07PROBE-2445|Canada.Manitoba
Heptagenia pulla|07PROBE-2443|Canada.Manitoba
Heptagenia pulla|07PROBE-2450|Canada.Manitoba
Heptagenia pulla|07PROBE-2446|Canada.Manitoba
Heptagenia pulla|SWA iBOL UofS 179|Canada.Saskatchewan
Heptagenia pulla|SWA iBOL UofS 175|Canada.Saskatchewan
Heptagenia pulla|08MBMAY-0034|Canada.Manitoba
Heptagenia pulla|07PROBE-2460|Canada.Manitoba
Heptagenia pulla|07PROBE-2024|Canada.Manitoba
Heptagenia pulla|07PROBE-2392|Canada.Manitoba
Heptagenia pulla|07PROBE-2022|Canada.Manitoba
Heptagenia pulla|07PROBE-07051|Canada.Manitoba
Heptagenia pulla|07PROBE-2448|Canada.Manitoba
Heptagenia pulla|07PROBE-2395|Canada.Manitoba
Heptagenia pulla|07PROBE-2402|Canada.Manitoba
Heptagenia pulla|07PROBE-2451|Canada.Manitoba
Heptagenia julia|09LJGSM-053|United States.North Carolina#
Heptagenia julia|09LJGSM-052|United States.North Carolina#
Heptagenia solitaria|EF514510|United States.Montana
Heptagenia solitaria|BIOUG00637-G05|United States.Colorado
Heptagenia solitaria|BIOUG00637-G04|United States.Colorado
Heptagenia sp.LJ1|09NBMAY-0007|Canada.New Brunswick
Heptagenia sp.LJ1|09NBMAY-0004|Canada.New Brunswick
Heptagenia elegantula|SWA iBOL UofS 190|Canada.Saskatchewan
Heptagenia elegantula|SWA iBOL UofS 189|Canada.Saskatchewan
Heptagenia elegantula|08INHSE-0149|United States.Illinois
Heptagenia elegantula|08INHSE-0144|United States.Illinois
Heptagenia elegantula|08INHSE-0142|United States.Illinois
Heptagenia elegantula|SWA iBOL UofS 3|Canada.Saskatchewan
Heptagenia elegantula|SWA iBOL UofS 147|Canada.Saskatchewan
Heptagenia elegantula|SWA iBOL UofS 148|Canada.Saskatchewan
Heptagenia elegantula|SWA iBOL UofS 4|Canada.Saskatchewan
Heptagenia elegantula|SWA iBOL UofS 5|Canada.Saskatchewan
Heptagenia elegantula|SWA iBOL UofS 151|Canada.Saskatchewan
Heptagenia elegantula|SWA iBOL UofS 154|Canada.Saskatchewan
Heptagenia elegantula|SWA iBOL UofS 1|Canada.Saskatchewan
Heptagenia elegantula|SWA iBOL UofS 157|Canada.Saskatchewan
Heptagenia elegantula|SWA iBOL UofS 156|Canada.Saskatchewan
Heptagenia elegantula|EF514508|United States.South Dakota
Heptagenia elegantula|EF514507|United States.North Dakota
Heptagenia elegantula|SWA iBOL UofS 150|Canada.Saskatchewan
Heptagenia elegantula|SWA iBOL UofS 155|Canada.Saskatchewan
Heptagenia elegantula|SWA iBOL UofS 6|Canada.Saskatchewan
Heptagenia elegantula|SWA iBOL UofS 153|Canada.Saskatchewan
Heptagenia adaequata|256 LR SK|Canada.Saskatchewan
Heptagenia whitingi|280 LF SK|Canada.Saskatchewan
Heptagenia whitingi|EF514516|Canada.Saskatchewan
Heptagenia whitingi|EF514518|Canada.Saskatchewan
Heptagenia flavescens|08INHSE-0153|United States.Illinois
Heptagenia flavescens|08INHSE-0151|United States.Illinois
Heptagenia flavescens|08INHSE-0150|United States.Illinois
Heptagenia flavescens|EF514514|United States.Indiana
Heptagenia flavescens|EF514513|United States.South Dakota
Heptagenia flavescens|08INHSE-0146|United States.Illinois
Heptagenia flavescens|08INHSE-0152|United States.Indiana
Heptagenia flavescens|08INHSE-0141|United States.Illinois
Heptagenia flavescens|EF514511|United States.South Dakota
Heptagenia flavescens|EF514512|United States.Nebraska
Heptagenia culacantha|EF514509|United States.Pennsylvania
Heptagenia nr. dolosa|09LJGSM-061|United States.North Carolina
Heptagenia nr. dolosa|HIEPT-1133|United States.Georgia
Heptagenia nr. townesi|08INHSE-0189|United States.North Carolina
Heptagenia nr. townesi JMW-2007|EF514519|United States.South Carolina
Heptagenia marginalis|EF514515|United States.Pennsylvania
Heptagenia marginalis|EF514517|United States.Kentucky
Rhithrogena uhari group|07GSM-5601|United States
Rhithrogena uhari group|09LJGSM-018|United States.North Carolina
Rhithrogena uhari group|07GSM-5599|United States.North Carolina
Rhithrogena uhari group|07GSM-5598|United States.North Carolina
Rhithrogena uhari group|09LJGSM-020|United States.North Carolina
Rhithrogena uhari group|09LJGSM-019|United States.North Carolina
Rhithrogena uhari group|09LJGSM-012|United States.North Carolina
Rhithrogena uhari group|07GSM-5590|United States.Tennessee
Rhithrogena uhari group|07GSM-5594|United States.Tennessee
Rhithrogena uhari group|07GSM-5940|United States.Tennessee
Rhithrogena uhari group|07GSM-5589|United States.Tennessee
Rhithrogena uhari group|10HIMXD-0266|United States.Georgia
Rhithrogena uhari group|HIEPT-1175|United States.Georgia
Rhithrogena uhari group|HIEPT-1126|United States.North Carolina
Rhithrogena anomala|07GSM-5593|United States.Tennessee
Rhithrogena anomala|07GSM-5586|United States
Rhithrogena anomala|07GSM-5591|United States.Tennessee
Rhithrogena anomala|07GSM-5975|United States.Tennessee
Rhithrogena anomala|07GSM-5584|United States
Rhithrogena anomala|07GSM-5976|United States.Tennessee
Rhithrogena anomala|10HIMXD-0276|United States.North Carolina
Rhithrogena anomala|10HIMXD-0275|United States.North Carolina
Rhithrogena anomala|10HIMXD-0277|United States.North Carolina
Rhithrogena nr. rubicunda|09NBMAY-0847|Canada.New Brunswick
Rhithrogena nr. rubicunda|09NBMAY-0812|Canada.New Brunswick
Rhithrogena nr. rubicunda|09NBMAY-0473|Canada.New Brunswick
Rhithrogena nr. rubicunda|09NBMAY-0764|Canada.New Brunswick
Rhithrogena nr. rubicunda|09NBMAY-0714|Canada.New Brunswick
Rhithrogena nr. rubicunda|09NBMAY-0585|Canada.New Brunswick
Heptagenia pulla
Heptagenia julia#
Heptagenia solitaria
Heptagenia sp.LJ1
Heptagenia elegantuls
Heptagenia adaequata
Heptagenia whitingi
Heptagenia flavescens
Heptagenia culacantha
Heptagenia nr. dolosa
Heptagenia nr. townesi
Heptagenia marginalis
Rhithrogena uhari group
Rhithrogena anomala
Rhithrogena nr. rubicunda

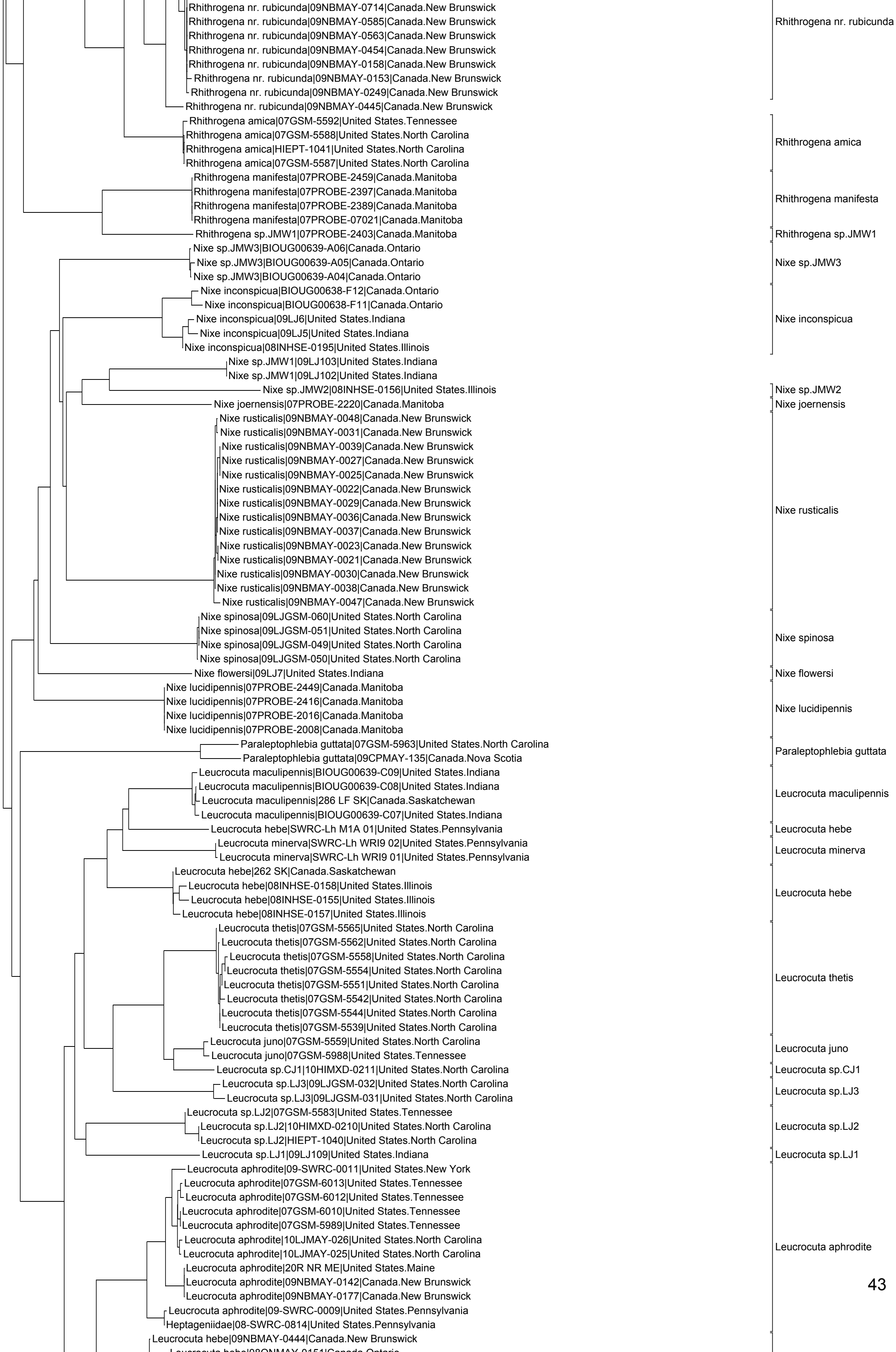
Rhithrogena nr. rubicunda|09NBMAY-0714|Canada.New Brunswick
Rhithrogena nr. rubicunda|09NBMAY-0585|Canada.New Brunswick
Rhithrogena nr. rubicunda|09NBMAY-0563|Canada.New Brunswick
Rhithrogena nr. rubicunda|09NBMAY-0454|Canada.New Brunswick
Rhithrogena nr. rubicunda|09NBMAY-0158|Canada.New Brunswick
Rhithrogena nr. rubicunda|09NBMAY-0153|Canada.New Brunswick
Rhithrogena nr. rubicunda|09NBMAY-0249|Canada.New Brunswick
Rhithrogena nr. rubicunda|09NBMAY-0445|Canada.New Brunswick
Rhithrogena amica|07GSM-5592|United States.Tennessee
Rhithrogena amica|07GSM-5588|United States.North Carolina
Rhithrogena amica|HIEPT-1041|United States.North Carolina
Rhithrogena amica|07GSM-5587|United States.North Carolina
Rhithrogena manifesta|07PROBE-2459|Canada.Manitoba
Rhithrogena manifesta|07PROBE-2397|Canada.Manitoba
Rhithrogena manifesta|07PROBE-2389|Canada.Manitoba
Rhithrogena manifesta|07PROBE-07021|Canada.Manitoba
Rhithrogena sp.JMW1|07PROBE-2403|Canada.Manitoba
Nixe sp.JMW3|BIOUG00639-A06|Canada.Ontario
Nixe sp.JMW3|BIOUG00639-A05|Canada.Ontario
Nixe sp.JMW3|BIOUG00639-A04|Canada.Ontario
Nixe inconspicua|BIOUG00638-F12|Canada.Ontario
Nixe inconspicua|BIOUG00638-F11|Canada.Ontario
Nixe inconspicua|09LJ6|United States.Indiana
Nixe inconspicua|09LJ5|United States.Indiana
Nixe inconspicua|08INHSE-0195|United States.Illinois
Nixe sp.JMW1|09LJ103|United States.Indiana
Nixe sp.JMW1|09LJ102|United States.Indiana
Nixe sp.JMW2|08INHSE-0156|United States.Illinois
Nixe joernensis|07PROBE-2220|Canada.Manitoba
Nixe rusticalis|09NBMAY-0048|Canada.New Brunswick
Nixe rusticalis|09NBMAY-0031|Canada.New Brunswick
Nixe rusticalis|09NBMAY-0039|Canada.New Brunswick
Nixe rusticalis|09NBMAY-0027|Canada.New Brunswick
Nixe rusticalis|09NBMAY-0025|Canada.New Brunswick
Nixe rusticalis|09NBMAY-0022|Canada.New Brunswick
Nixe rusticalis|09NBMAY-0029|Canada.New Brunswick
Nixe rusticalis|09NBMAY-0036|Canada.New Brunswick
Nixe rusticalis|09NBMAY-0037|Canada.New Brunswick
Nixe rusticalis|09NBMAY-0023|Canada.New Brunswick
Nixe rusticalis|09NBMAY-0021|Canada.New Brunswick
Nixe rusticalis|09NBMAY-0030|Canada.New Brunswick
Nixe rusticalis|09NBMAY-0038|Canada.New Brunswick
Nixe rusticalis|09NBMAY-0047|Canada.New Brunswick
Nixe spinosa|09LJGSM-060|United States.North Carolina
Nixe spinosa|09LJGSM-051|United States.North Carolina
Nixe spinosa|09LJGSM-049|United States.North Carolina
Nixe spinosa|09LJGSM-050|United States.North Carolina
Nixe flowersi|09LJ7|United States.Indiana
Nixe lucidipennis|07PROBE-2449|Canada.Manitoba
Nixe lucidipennis|07PROBE-2416|Canada.Manitoba
Nixe lucidipennis|07PROBE-2016|Canada.Manitoba
Nixe lucidipennis|07PROBE-2008|Canada.Manitoba
Paraleptophlebia guttata|07GSM-5963|United States.North Carolina
Paraleptophlebia guttata|09CPMAY-135|Canada.Nova Scotia
Leucrocuta maculipennis|BIOUG00639-C09|United States.Indiana
Leucrocuta maculipennis|BIOUG00639-C08|United States.Indiana
Leucrocuta maculipennis|286 LF SK|Canada.Saskatchewan
Leucrocuta maculipennis|BIOUG00639-C07|United States.Indiana
Leucrocuta hebe|SWRC-Lh M1A 01|United States.Pennsylvania
Leucrocuta minerva|SWRC-Lh WRI9 02|United States.Pennsylvania
Leucrocuta minerva|SWRC-Lh WRI9 01|United States.Pennsylvania
Leucrocuta hebe|262 SK|Canada.Saskatchewan
Leucrocuta hebe|08INHSE-0158|United States.Illinois
Leucrocuta hebe|08INHSE-0155|United States.Illinois
Leucrocuta hebe|08INHSE-0157|United States.Illinois
Leucrocuta thetis|07GSM-5565|United States.North Carolina
Leucrocuta thetis|07GSM-5562|United States.North Carolina
Leucrocuta thetis|07GSM-5558|United States.North Carolina
Leucrocuta thetis|07GSM-5554|United States.North Carolina
Leucrocuta thetis|07GSM-5551|United States.North Carolina
Leucrocuta thetis|07GSM-5542|United States.North Carolina
Leucrocuta thetis|07GSM-5544|United States.North Carolina
Leucrocuta thetis|07GSM-5539|United States.North Carolina
Leucrocuta juno|07GSM-5559|United States.North Carolina
Leucrocuta juno|07GSM-5988|United States.Tennessee
Leucrocuta sp.CJ1|10HIMXD-0211|United States.North Carolina
Leucrocuta sp.LJ3|09LJGSM-032|United States.North Carolina
Leucrocuta sp.LJ3|09LJGSM-031|United States.North Carolina
Leucrocuta sp.LJ2|07GSM-5583|United States.Tennessee
Leucrocuta sp.LJ2|10HIMXD-0210|United States.North Carolina
Leucrocuta sp.LJ2|HIEPT-1040|United States.North Carolina
Leucrocuta sp.LJ1|09LJ109|United States.Indiana
Leucrocuta aphrodite|09-SWRC-0011|United States.New York
Leucrocuta aphrodite|07GSM-6013|United States.Tennessee
Leucrocuta aphrodite|07GSM-6012|United States.Tennessee
Leucrocuta aphrodite|07GSM-6010|United States.Tennessee
Leucrocuta aphrodite|07GSM-5989|United States.Tennessee
Leucrocuta aphrodite|10LJMAY-026|United States.North Carolina
Leucrocuta aphrodite|10LJMAY-025|United States.North Carolina
Leucrocuta aphrodite|20R NR ME|United States.Maine
Leucrocuta aphrodite|09NBMAY-0142|Canada.New Brunswick
Leucrocuta aphrodite|09NBMAY-0177|Canada.New Brunswick
Leucrocuta aphrodite|09-SWRC-0009|United States.Pennsylvania
Heptageniidae|08-SWRC-0814|United States.Pennsylvania
Leucrocuta hebe|09NBMAY-0444|Canada.New Brunswick
Rhithrogena nr. rubicunda
Rhithrogena amica
Rhithrogena manifesta
Rhithrogena sp.JMW1
Nixe sp.JMW3
Nixe inconspicua
Nixe sp.JMW2
Nixe joernensis
Nixe rusticalis
Nixe spinosa
Nixe flowersi
Nixe lucidipennis
Paraleptophlebia guttata
Leucrocuta maculipennis
Leucrocuta hebe
Leucrocuta minerva
Leucrocuta hebe
Leucrocuta thetis
Leucrocuta juno
Leucrocuta sp.CJ1
Leucrocuta sp.LJ3
Leucrocuta sp.LJ2
Leucrocuta sp.LJ1
Leucrocuta aphrodite

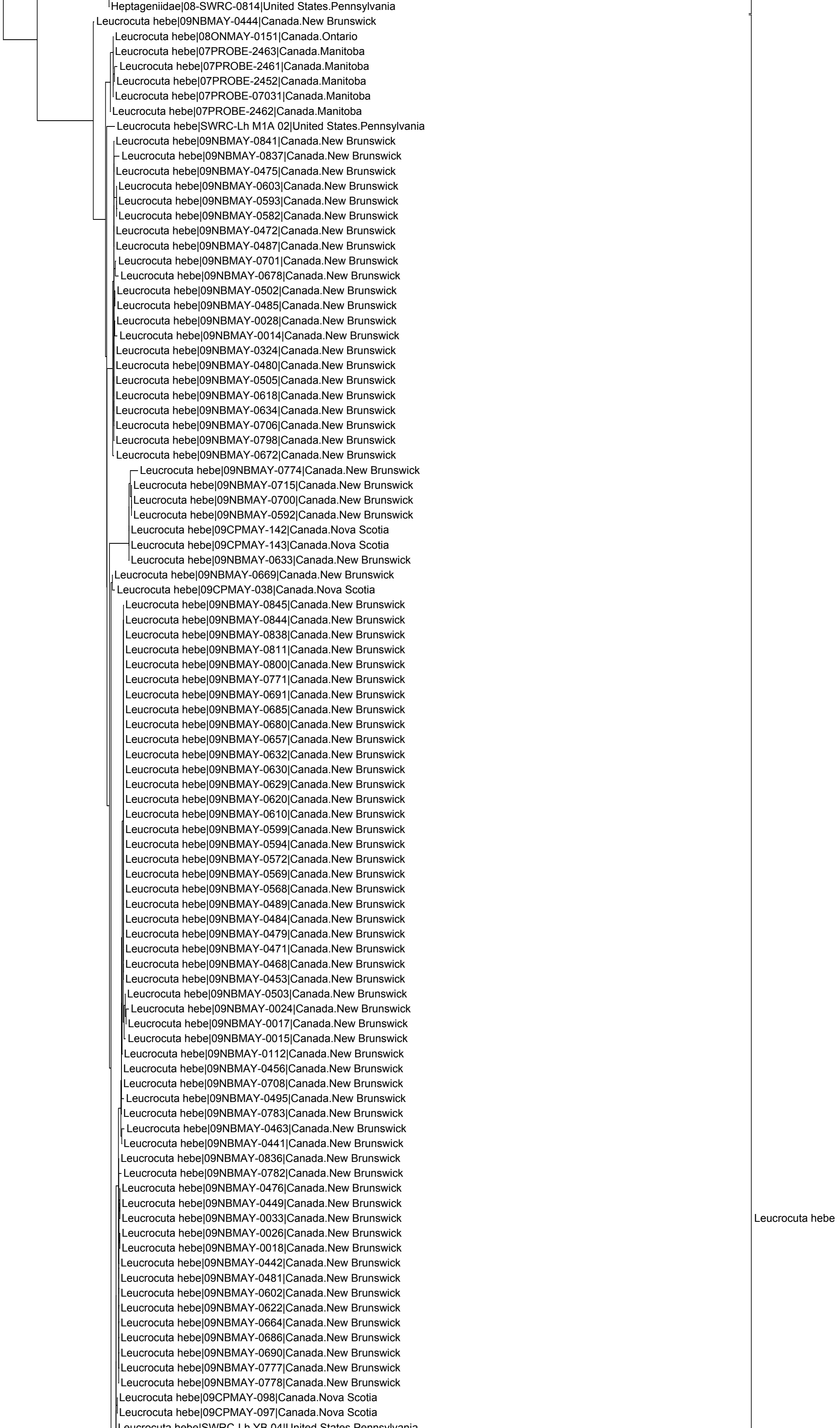
Heptageniidae|08-SWRC-0814|United States.Pennsylvania
Leucrocuta hebe|09NBMAY-0444|Canada.New Brunswick
Leucrocuta hebe|08ONMAY-0151|Canada.Ontario
Leucrocuta hebe|07PROBE-2463|Canada.Manitoba
Leucrocuta hebe|07PROBE-2461|Canada.Manitoba
Leucrocuta hebe|07PROBE-2452|Canada.Manitoba
Leucrocuta hebe|07PROBE-07031|Canada.Manitoba
Leucrocuta hebe|07PROBE-2462|Canada.Manitoba
Leucrocuta hebe|SWRC-Lh M1A 02|United States.Pennsylvania
Leucrocuta hebe|09NBMAY-0841|Canada.New Brunswick
Leucrocuta hebe|09NBMAY-0837|Canada.New Brunswick
Leucrocuta hebe|09NBMAY-0475|Canada.New Brunswick
Leucrocuta hebe|09NBMAY-0603|Canada.New Brunswick
Leucrocuta hebe|09NBMAY-0593|Canada.New Brunswick
Leucrocuta hebe|09NBMAY-0582|Canada.New Brunswick
Leucrocuta hebe|09NBMAY-0472|Canada.New Brunswick
Leucrocuta hebe|09NBMAY-0487|Canada.New Brunswick
Leucrocuta hebe|09NBMAY-0701|Canada.New Brunswick
Leucrocuta hebe|09NBMAY-0678|Canada.New Brunswick
Leucrocuta hebe|09NBMAY-0502|Canada.New Brunswick
Leucrocuta hebe|09NBMAY-0485|Canada.New Brunswick
Leucrocuta hebe|09NBMAY-0028|Canada.New Brunswick
Leucrocuta hebe|09NBMAY-0014|Canada.New Brunswick
Leucrocuta hebe|09NBMAY-0324|Canada.New Brunswick
Leucrocuta hebe|09NBMAY-0480|Canada.New Brunswick
Leucrocuta hebe|09NBMAY-0505|Canada.New Brunswick
Leucrocuta hebe|09NBMAY-0618|Canada.New Brunswick
Leucrocuta hebe|09NBMAY-0634|Canada.New Brunswick
Leucrocuta hebe|09NBMAY-0706|Canada.New Brunswick
Leucrocuta hebe|09NBMAY-0798|Canada.New Brunswick
Leucrocuta hebe|09NBMAY-0672|Canada.New Brunswick
Leucrocuta hebe|09NBMAY-0774|Canada.New Brunswick
Leucrocuta hebe|09NBMAY-0715|Canada.New Brunswick
Leucrocuta hebe|09NBMAY-0700|Canada.New Brunswick
Leucrocuta hebe|09NBMAY-0592|Canada.New Brunswick
Leucrocuta hebe|09CPMAY-142|Canada.Nova Scotia
Leucrocuta hebe|09CPMAY-143|Canada.Nova Scotia
Leucrocuta hebe|09NBMAY-0633|Canada.New Brunswick
Leucrocuta hebe|09NBMAY-0669|Canada.New Brunswick
Leucrocuta hebe|09CPMAY-038|Canada.Nova Scotia
Leucrocuta hebe|09NBMAY-0845|Canada.New Brunswick
Leucrocuta hebe|09NBMAY-0844|Canada.New Brunswick
Leucrocuta hebe|09NBMAY-0838|Canada.New Brunswick
Leucrocuta hebe|09NBMAY-0811|Canada.New Brunswick
Leucrocuta hebe|09NBMAY-0800|Canada.New Brunswick
Leucrocuta hebe|09NBMAY-0771|Canada.New Brunswick
Leucrocuta hebe|09NBMAY-0691|Canada.New Brunswick
Leucrocuta hebe|09NBMAY-0685|Canada.New Brunswick
Leucrocuta hebe|09NBMAY-0680|Canada.New Brunswick
Leucrocuta hebe|09NBMAY-0657|Canada.New Brunswick
Leucrocuta hebe|09NBMAY-0632|Canada.New Brunswick
Leucrocuta hebe|09NBMAY-0630|Canada.New Brunswick
Leucrocuta hebe|09NBMAY-0629|Canada.New Brunswick
Leucrocuta hebe|09NBMAY-0620|Canada.New Brunswick
Leucrocuta hebe|09NBMAY-0610|Canada.New Brunswick
Leucrocuta hebe|09NBMAY-0599|Canada.New Brunswick
Leucrocuta hebe|09NBMAY-0594|Canada.New Brunswick
Leucrocuta hebe|09NBMAY-0572|Canada.New Brunswick
Leucrocuta hebe|09NBMAY-0569|Canada.New Brunswick
Leucrocuta hebe|09NBMAY-0568|Canada.New Brunswick
Leucrocuta hebe|09NBMAY-0489|Canada.New Brunswick
Leucrocuta hebe|09NBMAY-0484|Canada.New Brunswick
Leucrocuta hebe|09NBMAY-0479|Canada.New Brunswick
Leucrocuta hebe|09NBMAY-0471|Canada.New Brunswick
Leucrocuta hebe|09NBMAY-0468|Canada.New Brunswick
Leucrocuta hebe|09NBMAY-0453|Canada.New Brunswick
Leucrocuta hebe|09NBMAY-0503|Canada.New Brunswick
Leucrocuta hebe|09NBMAY-0024|Canada.New Brunswick
Leucrocuta hebe|09NBMAY-0017|Canada.New Brunswick
Leucrocuta hebe|09NBMAY-0015|Canada.New Brunswick
Leucrocuta hebe|09NBMAY-0112|Canada.New Brunswick
Leucrocuta hebe|09NBMAY-0456|Canada.New Brunswick
Leucrocuta hebe|09NBMAY-0708|Canada.New Brunswick
Leucrocuta hebe|09NBMAY-0495|Canada.New Brunswick
Leucrocuta hebe|09NBMAY-0783|Canada.New Brunswick
Leucrocuta hebe|09NBMAY-0463|Canada.New Brunswick
Leucrocuta hebe|09NBMAY-0441|Canada.New Brunswick
Leucrocuta hebe|09NBMAY-0836|Canada.New Brunswick
Leucrocuta hebe|09NBMAY-0782|Canada.New Brunswick
Leucrocuta hebe|09NBMAY-0476|Canada.New Brunswick
Leucrocuta hebe|09NBMAY-0449|Canada.New Brunswick
Leucrocuta hebe|09NBMAY-0033|Canada.New Brunswick
Leucrocuta hebe|09NBMAY-0026|Canada.New Brunswick
Leucrocuta hebe|09NBMAY-0018|Canada.New Brunswick
Leucrocuta hebe|09NBMAY-0442|Canada.New Brunswick
Leucrocuta hebe|09NBMAY-0481|Canada.New Brunswick
Leucrocuta hebe|09NBMAY-0602|Canada.New Brunswick
Leucrocuta hebe|09NBMAY-0622|Canada.New Brunswick
Leucrocuta hebe|09NBMAY-0664|Canada.New Brunswick
Leucrocuta hebe|09NBMAY-0686|Canada.New Brunswick
Leucrocuta hebe|09NBMAY-0690|Canada.New Brunswick
Leucrocuta hebe|09NBMAY-0777|Canada.New Brunswick
Leucrocuta hebe|09NBMAY-0778|Canada.New Brunswick
Leucrocuta hebe|09CPMAY-098|Canada.Nova Scotia
Leucrocuta hebe|09CPMAY-097|Canada.Nova Scotia
Leucrocuta hebe

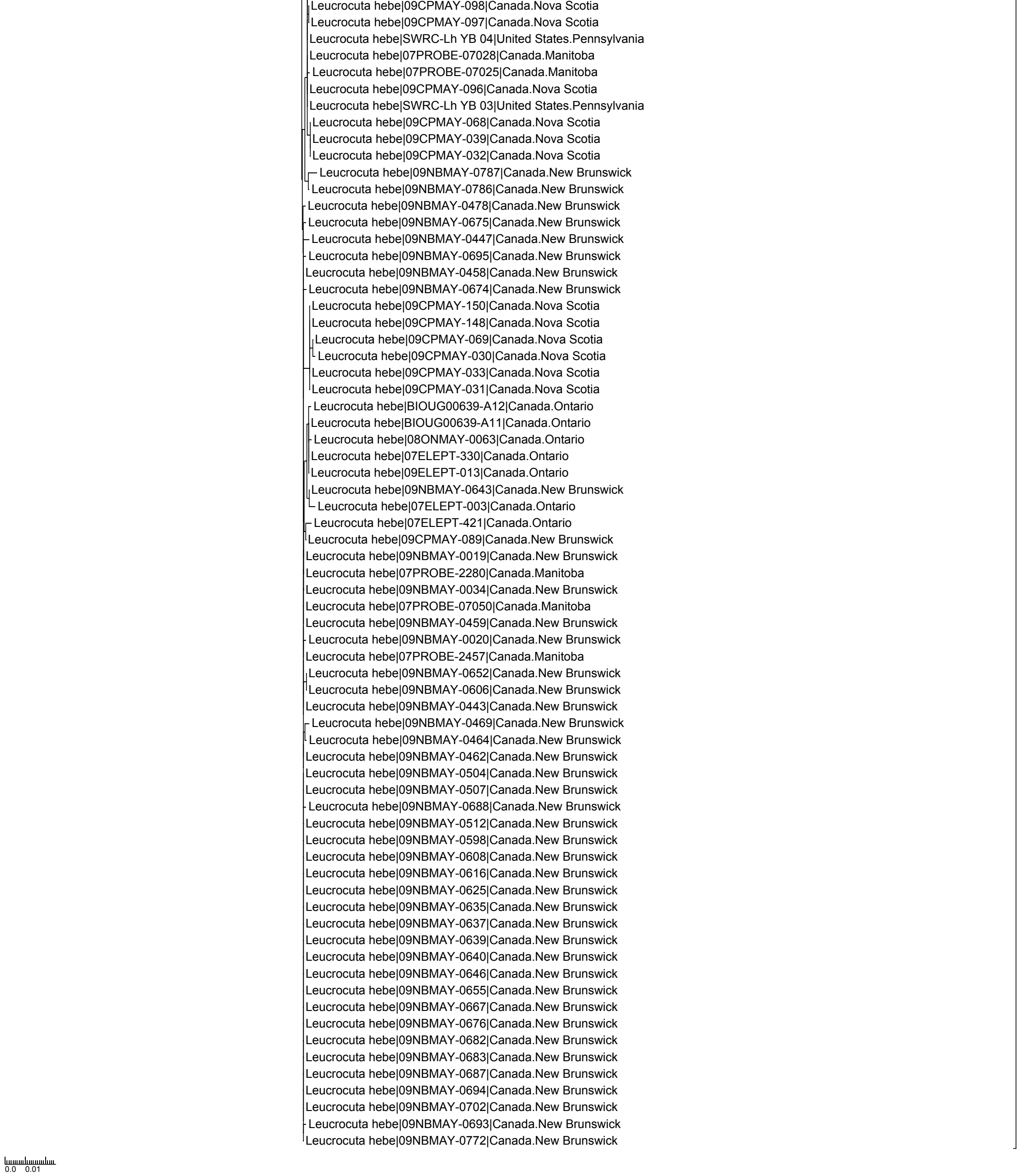
Leucrocuta hebe|09CPMAY-098|Canada.Nova Scotia
Leucrocuta hebe|09CPMAY-097|Canada.Nova Scotia
Leucrocuta hebe|SWRC-Lh YB 04|United States.Pennsylvania
Leucrocuta hebe|07PROBE-07028|Canada.Manitoba
Leucrocuta hebe|07PROBE-07025|Canada.Manitoba
Leucrocuta hebe|09CPMAY-096|Canada.Nova Scotia
Leucrocuta hebe|SWRC-Lh YB 03|United States.Pennsylvania
Leucrocuta hebe|09CPMAY-068|Canada.Nova Scotia
Leucrocuta hebe|09CPMAY-039|Canada.Nova Scotia
Leucrocuta hebe|09CPMAY-032|Canada.Nova Scotia
Leucrocuta hebe|09NBMAY-0787|Canada.New Brunswick
Leucrocuta hebe|09NBMAY-0786|Canada.New Brunswick
Leucrocuta hebe|09NBMAY-0478|Canada.New Brunswick
Leucrocuta hebe|09NBMAY-0675|Canada.New Brunswick
Leucrocuta hebe|09NBMAY-0447|Canada.New Brunswick
Leucrocuta hebe|09NBMAY-0695|Canada.New Brunswick
Leucrocuta hebe|09NBMAY-0458|Canada.New Brunswick
Leucrocuta hebe|09NBMAY-0674|Canada.New Brunswick
Leucrocuta hebe|09CPMAY-150|Canada.Nova Scotia
Leucrocuta hebe|09CPMAY-148|Canada.Nova Scotia
Leucrocuta hebe|09CPMAY-069|Canada.Nova Scotia
Leucrocuta hebe|09CPMAY-030|Canada.Nova Scotia
Leucrocuta hebe|09CPMAY-033|Canada.Nova Scotia
Leucrocuta hebe|09CPMAY-031|Canada.Nova Scotia
Leucrocuta hebe|BIOUG00639-A12|Canada.Ontario
Leucrocuta hebe|BIOUG00639-A11|Canada.Ontario
Leucrocuta hebe|08ONMAY-0063|Canada.Ontario
Leucrocuta hebe|07ELEPT-330|Canada.Ontario
Leucrocuta hebe|09ELEPT-013|Canada.Ontario
Leucrocuta hebe|09NBMAY-0643|Canada.New Brunswick
Leucrocuta hebe|07ELEPT-003|Canada.Ontario
Leucrocuta hebe|07ELEPT-421|Canada.Ontario
Leucrocuta hebe|09CPMAY-089|Canada.New Brunswick
Leucrocuta hebe|09NBMAY-0019|Canada.New Brunswick
Leucrocuta hebe|07PROBE-2280|Canada.Manitoba
Leucrocuta hebe|09NBMAY-0034|Canada.New Brunswick
Leucrocuta hebe|07PROBE-07050|Canada.Manitoba
Leucrocuta hebe|09NBMAY-0459|Canada.New Brunswick
Leucrocuta hebe|09NBMAY-0020|Canada.New Brunswick
Leucrocuta hebe|07PROBE-2457|Canada.Manitoba
Leucrocuta hebe|09NBMAY-0652|Canada.New Brunswick
Leucrocuta hebe|09NBMAY-0606|Canada.New Brunswick
Leucrocuta hebe|09NBMAY-0443|Canada.New Brunswick
Leucrocuta hebe|09NBMAY-0469|Canada.New Brunswick
Leucrocuta hebe|09NBMAY-0464|Canada.New Brunswick
Leucrocuta hebe|09NBMAY-0462|Canada.New Brunswick
Leucrocuta hebe|09NBMAY-0504|Canada.New Brunswick
Leucrocuta hebe|09NBMAY-0507|Canada.New Brunswick
Leucrocuta hebe|09NBMAY-0688|Canada.New Brunswick
Leucrocuta hebe|09NBMAY-0512|Canada.New Brunswick
Leucrocuta hebe|09NBMAY-0598|Canada.New Brunswick
Leucrocuta hebe|09NBMAY-0608|Canada.New Brunswick
Leucrocuta hebe|09NBMAY-0616|Canada.New Brunswick
Leucrocuta hebe|09NBMAY-0625|Canada.New Brunswick
Leucrocuta hebe|09NBMAY-0635|Canada.New Brunswick
Leucrocuta hebe|09NBMAY-0637|Canada.New Brunswick
Leucrocuta hebe|09NBMAY-0639|Canada.New Brunswick
Leucrocuta hebe|09NBMAY-0640|Canada.New Brunswick
Leucrocuta hebe|09NBMAY-0646|Canada.New Brunswick
Leucrocuta hebe|09NBMAY-0655|Canada.New Brunswick
Leucrocuta hebe|09NBMAY-0667|Canada.New Brunswick
Leucrocuta hebe|09NBMAY-0676|Canada.New Brunswick
Leucrocuta hebe|09NBMAY-0682|Canada.New Brunswick
Leucrocuta hebe|09NBMAY-0683|Canada.New Brunswick
Leucrocuta hebe|09NBMAY-0687|Canada.New Brunswick
Leucrocuta hebe|09NBMAY-0694|Canada.New Brunswick
Leucrocuta hebe|09NBMAY-0702|Canada.New Brunswick
Leucrocuta hebe|09NBMAY-0693|Canada.New Brunswick
Leucrocuta hebe|09NBMAY-0772|Canada.New Brunswick
0.0 0.01
