# Supplementary material for: A DNA Barcode Library for North American Ephemeroptera: Progress and Prospects
Source: PLoS One. 2012 May 30;7(5):e38063. doi: 10.1371/journal.pone.0038063 (PMC3364165; doi:10.1371/journal.pone.0038063)
Supplement: Table S1 — Species-level summary of K2P distance, sample distribution, and sample size for North American Ephemeroptera. All distributional records use standard 2-letter (or 3-letter, for Mexico) postal abbreviations. MNID = minimum interspecific K2P distance, MXID = maximum intraspecific K2P distance, species with maximum intraspecific sequence divergence ≥5.0% are indicated with ‘*’. (DOC) [file pone.0038063.s002.doc]

**Table S1 Species-level summary of K2P distance, sample distribution, and sample size for North American Ephemeroptera**

| **Family** | **Species** | **Distribution** | **N** | **MXID (%)** | **MNID (%)** |
| --- | --- | --- | --- | --- | --- |
| **Ameletidae** | | | | | |
|  | *Ameletus amador* | CA | 1 | – | 14.43 |
|  | *Ameletus andersoni* | OR, WA | 4 | 3.26 | 5.82 |
|  | *Ameletus bellulus* | AB | 2 | 0.16 | 10.47 |
|  | *Ameletus browni* | VT | 2 | 0.16 | 12.87 |
|  | *Ameletus celer* | AB, BC | 14 | 0.77 | 13.95 |
|  | *Ameletus cf celer* | BC | 2 | 0 | 17.38 |
|  | *Ameletus cooki* | AB | 6 | 0.48 | 14.33 |
|  | *Ameletus cryptostimulus* | TN | 1 | – | 12.87 |
|  | *Ameletus dissitus* | CA | 1 | – | 5.82 |
|  | *Ameletus doddsianus* | CO | 1 | – | 14.42 |
|  | *Ameletus lineatus* | IL, IN, ME, OH, ON, PA | 14 | 0.76 | 14.17 |
|  | *Ameletus oregonensis* | AB | 4 | 0.64 | 15.72 |
|  | *Ameletus pritchardi* | AB | 2 | 0.8 | 17.03 |
|  | *Ameletus similior* | AB, BC | 6 | 2.8 | 15.74 |
|  | *Ameletus subnotatus* | AB, CO, SK | 10 | 2.75 | 15.72 |
|  | *Ameletus suffusus* | AB | 2 | 1.29 | 11.62 |
|  | *Ameletus tarteri* | WV | 2 | 2.44 | 14.17 |
|  | *Ameletus tertius* | GA, NC, TN | 6 | 0.31 | 17.79 |
|  | *Ameletus validus* | AB | 1 | – | 15.95 |
|  | *Ameletus velox* | AB | 2 | 1.61 | 13.95 |
|  | *Ameletus vernalis* | AB | 3 | 0.8 | 11.02 |
| **Ametropodidae** | | | | | |
|  | *Ametropus neavei* | SK | 4 | 0.2 | 18.5 |
| **Baetidae** | | | | | |
|  | *Acentrella barbarae* | NC | 5 | 3.6 | 17.4 |
| ***** | *Acentrella insignificans* | AZ, | 3 | 22.9 | 17.7 |
|  | *Acentrella lapponica* | **AB** | 4 | 0.3 | 16.7 |
|  | *Acentrella nadineae* | **TN** | 4 | 0 | 17.8 |
| ***** | *Acentrella parvula* | NB, NY, ON, SK | 20 | 23.2 | 15 |
|  | *Acerpenna sp.JMW1* | MB, ON | 13 | 1.2 | 2.8 |
|  | *Acentrella sp.LJ1* | IN | 1 | – | 15.1 |
| ***** | *Acentrella turbida* | GA, MB, NY, NC, ON, SC, TN | 32 | 21.8 | 15.1 |
|  | *Acerpenna macdunnoughi* | IN, NY, NC, PA, TN | 7 | 3.1 | 17.9 |
| ***** | *Acerpenna pygmaea* | AL, FL, IL, MB, NB, ON | 17 | 26.2 | 13.5 |
|  | *Acerpenna sp. CHU1* | MB | 10 | 0 | 2.8 |
|  | *Americabaetis cf. pleturus* | VER | 2 | 0 | 18.6 |
|  | *Apobaetis sp.LJ1* | NC | 1 | – | 21.6 |
| ***** | *Baetis adonis* | CA | 8 | 18.9 | 0.9 |
| ***** | *Baetis bicaudatus* | AB, BC | 10 | 7.2 | 18.8 |
|  | *Baetis brunneicolor* | IL, MB, MN, SK | 65 | 1.9 | 13 |
|  | *Baetis bundyae* | AK, MB | 5 | 1.4 | 4.1 |
| ***** | *Baetis flavistriga* | NY, NC, TN | 11 | 9.4 | 3.6 |
|  | *Baetis foemina* | **NL** | 7 | 0.2 | 18.5 |
|  | *Baetis hudsonicus* | MB | 3 | 0.5 | 4.1 |
| ***** | *Baetis intercalaris* | FL, IN, NY, NC, NS, ON, TN | 16 | 22.2 | 18.2 |
| ***** | *Baetis magnus* | CO, VER | 5 | 20.6 | 17.5 |
|  | *Baetis notos* | AZ | 8 | 2.3 | 19.5 |
|  | *Baetis persecutor* | AB | 4 | 0 | 15.7 |
|  | *Baetis phoebus* | CO, IL, MB, MN, NY, NS, SK | 216 | 3.4 | 18.1 |
|  | *Baetis pluto* | **FL**, NC, TN | 11 | 3.9 | 19 |
|  | *Baetis rusticans* | NY, ON | 4 | 1.2 | 3.6 |
|  | *Baetis spCA1* | CA | 3 | 0.5 | 15.7 |
|  | *Baetis sp.JMW1* | NS | 1 | – | 19 |
| ***** | *Baetis tricaudatus* | AB, AZ, BC, CA, CO, IL, MN, NM, NY, NC, NS, ON, SK, TN, UT | 151 | 22.6 | 0.9 |
|  | *Baetodes caritus* | VER | 3 | 0.2 | 19.8 |
|  | *Baetodes sp.JMW1* | VER | 3 | 1.1 | 18.4 |
|  | *Baetodes sp.JMW2* | VER | 1 | – | 21.2 |
|  | *Baetodes tritus* | VER | 3 | 0.2 | 19 |
|  | *Barbaetis benfieldi* | NC | 1 | – | 23.4 |
| ***** | *Callibaetis ferrugineus* | AB, BC, IL, MB, NB, NY, ON, PA | 36 | 16.4 | 14.1 |
|  | *Callibaetis floridanus* | FL | 1 | – | 10.7 |
|  | *Callibaetis fluctuans* | IL, PA | 7 | 1.1 | 16.5 |
|  | *Callibaetis pictus* | AZ | 4 | 0.3 | 17.1 |
|  | *Callibaetis pretiosus* | NC | 1 | – | 14.1 |
|  | *Callibaetis sp.CA1* | CA | 1 | – | 17.5 |
|  | *Callibaetis sp.DHF1* | MD | 1 | – | 10.7 |
| ***** | *Camelobaetidius cf. kondratieffi* | VER | 3 | 6.6 | 18.4 |
|  | *Camelobaetidius mexicanus* | VER | 3 | 0.9 | 17.6 |
|  | *Camelobaetidius nr. warreni* | AZ | 1 | – | 17.6 |
| ***** | *Centroptilum alamance* | NC, PA | 2 | 9.4 | 15.2 |
|  | *Centroptilum bifurcatum* | SK | 15 | 0.8 | 21.8 |
| ***** | *Centroptilum minor* | NY, PA, SC | 16 | 20 | 16.3 |
|  | *Centroptilum sp.CA1* | CA | 2 | 0.2 | 18.8 |
|  | *Centroptilum sp.JMW1* | NB | 1 | – | 20.6 |
| ***** | *Centroptilum triangulifer* | IN, ME, NC, ON, PA, SC | 10 | 23.1 | 15.2 |
|  | *Centroptilum victoriae* | IN, NB, ON | 8 | 2.4 | 15.9 |
|  | *Cloeon cognatum* | MI, PA | 4 | 0.2 | 14.2 |
| ***** | *Diphetor hageni* | CA, FL, IN, PA, SK | 15 | 20.6 | 15.5 |
| ***** | *Fallceon quilleri* | AZ, CA, CO, NM | 10 | 26.7 | 16.9 |
|  | *Fallceon sp.JMW1* | VER | 4 | 2.3 | 17.9 |
|  | *Heterocloeon amplum* | NY | 2 | 0.8 | 16.9 |
|  | *Heterocloeon curiosum* | GA, NY, NC, SC | 5 | 1.7 | 16.9 |
|  | *Iswaeon anoka* | MB, NB, NC, ON | 46 | 4.4 | 16.8 |
|  | *Moribaetis macaferti* | VER | 3 | 0.2 | 19.5 |
|  | *Plauditus cingulatus* | NB, NY | 9 | 0.5 | 18.1 |
|  | *Plauditus dubius* | NB, NY, TN | 71 | 2.2 | 16.3 |
|  | *Plauditus sp.CHU1* | MB | 2 | 0 | 5.2 |
|  | *Plauditus sp.CHU2* | MB, ON | 12 | 1.1 | 17.3 |
|  | *Plauditus sp.JMW1* | IN | 1 | – | 16.4 |
|  | *Plauditus sp.JMW2* | NC | 1 | – | 16.3 |
|  | *Plauditus sp.LJ1* | NB, NY | 11 | 1.5 | 16 |
|  | *Plauditus sp.LJ2* | NB | 6 | 0.3 | 5.2 |
|  | *Plauditus virilis* | IN, ON | 24 | 0.8 | 17.1 |
|  | *Procloeon fragile* | ON, PA | 5 | 2.6 | 15.8 |
|  | *Procloeon intermediale* | NY | 1 | – | 15.5 |
|  | *Procloeon mendax* | MB | 11 | 0.3 | 15.4 |
|  | *Procloeon pennulatum* | MB, SK | 9 | 0.3 | 16.1 |
|  | *Procloeon rivulare* | GA, NY, PA, TN | 9 | 3.1 | 15.4 |
|  | *Procloeon rubropictum* | SK | 1 | – | 10.7 |
|  | *Procloeon rufostrigatum* | PA | 1 | – | 15.5 |
|  | *Procloeon sp.JMW1* | NB | 1 | – | 17.8 |
|  | *Procloeon sp.JMW2* | IN | 1 | – | 10.7 |
|  | *Procloeon sp.JMW3* | IN, ON | 3 | 2.6 | 15.4 |
|  | *Procloeon sp.JMW4* | AB, SK | 6 | 0.6 | 14.2 |
|  | *Procloeon sp.LJ1* | NC | 3 | 3.1 | 17.1 |
| ***** | *Procloeon viridoculare* | ME, SC | 2 | 18.1 | 15.6 |
|  | *Pseudocloeon ephippiatum* | IL | 1 | – | 17.2 |
|  | *Pseudocloeon frondale* | IN | 1 | – | 8.4 |
|  | *Pseudocloeon propinquum* | IL, IN, NY, SK | 6 | 2.5 | 8.4 |
| **Baetiscidae** | | | | | |
|  | *Baetisca carolina* | GA, NC, TN | 10 | 4.1 | 14.1 |
|  | *Baetisca lacustris* | IL, SK | 7 | 2.4 | 2.9 |
|  | *Baetisca laurentina* | ME, MB, ON, SK | 26 | 1.8 | 2.9 |
|  | *Baetisca obesa* | IL | 1 | – | 17.1 |
| **Behningiidae** | | | | | |
| ***** | *Dolania americana* | AL, FL | 5 | 7.9 |  |
| **Caenidae** | | | | | |
|  | *Brachycercus harrisella* | SK | 1 | – | 17.4 |
| ***** | *Caenis amica* | AR, FL, IL, NB, ON, SK | 12 | 21.9 | 0.5 |
|  | *Caenis anceps* | **ON** | 7 | 0.6 | 19.5 |
|  | *Caenis bajaensis* | CA, CO | 5 | 4.3 | 19.9 |
| ***** | *Caenis diminuta* | FL, NB, ON | 36 | 21 | 13.7 |
|  | *Caenis eglinensis* | FL | 4 | 0.6 | 11.6 |
|  | *Caenis hilaris* | IN | 2 | 2 | 19.7 |
| ***** | *Caenis latipennis* | AB, AZ, NB, OK, ON, SK | 43 | 12.4 | 9.2 |
| ***** | *Caenis punctata* | AZ, AR, IL, MO, NB, OK | 10 | 20.4 | 0.3 |
|  | *Caenis sp.XZ1* | ON | 1 | – | 9.6 |
|  | *Caenis youngi* | AB, MB, NB, ON, SK | 47 | 3.7 | 0.3 |
|  | *Cercobrachys cree* | SK | 2 | 0.8 | 14.5 |
|  | *Susperatus prudens* | SK | 2 | 0.3 | 14.5 |
| **Ephemerellidae** | | | | | |
| ***** | *Attenella attenuata* | DE, NS, PA, SC | 4 | 5.4 | 15.4 |
| ***** | *Attenella margarita* | CO, WA | 3 | 13.8 | 15.4 |
| ***** | *Dannella lita* | MB, ON | 4 | 9.8 | 4.8 |
|  | *Dannella provonshai* | NC | 1 | – | 4.8 |
| ***** | *Dannella simplex* | AL, GA, MB, NC, PA | 10 | 14.2 | 5.6 |
|  | *Dentatella coxalis* | NH | 2 | 0.3 | 12.9 |
|  | *Drunella coloradensis* | AB, BC | 9 | 0.7 | 15.9 |
|  | *Drunella cornuta* | NY, VT | 4 | 0.3 | 16.5 |
|  | *Drunella cornutella* | NH, NY, VT | 7 | 0.8 | 10.3 |
| ***** | *Drunella doddsii* | AB, BC, CO | 4 | 7.6 | 19.4 |
|  | *Drunella flavilinea* | CA | 1 | – | 4.9 |
|  | *Drunella grandis* | BC, CO, NM | 5 | 1.2 | 19.9 |
|  | *Drunella lata* | GA, NY, PA, SC | 22 | 0.5 | 15.9 |
|  | *Drunella lata complex* | GA, NC, SC | 10 | 1.1 | 10.3 |
|  | *Drunella longicornis* | NC | 3 | 0 | 12.1 |
|  | *Drunella sp.CA1* | CA | 4 | 2.2 | 4.9 |
|  | *Drunella tuberculata* | GA, NC | 5 | 1.7 | 10.8 |
| ***** | *Drunella walkeri* | ME, NY, NC | 4 | 10.6 | 10.8 |
|  | *Ephemerella aurivillii* | NY, ON | 6 | 0.5 | 17.5 |
|  | *Ephemerella catawba* | GA, NC, TN | 8 | 3.1 | 10.6 |
| ***** | *Ephemerella dorothea* | GA, NY, NC, NS, PA, SC, TN, WV | 236 | 17 | 4.6 |
| ***** | *Ephemerella dorothea infrequens* | AB, BC, CA, CO | 11 | 12.5 | 11.9 |
| ***** | *Ephemerella excrucians* | BC, GA, NC | 19 | 20.6 | 5.7 |
|  | *Ephemerella floripara* | NC, PA | 8 | 1.9 | 9.3 |
| ***** | *Ephemerella invaria* | ME, NB, NY, NC, NS, PA, TN, | 129 | 8.2 | 7.1 |
|  |  |  |  |  |  |
|  | *Ephemerella maculata* | CA | 4 | 1.3 | 18.3 |
|  | *Ephemerella needhami* | MB | 16 | 0.3 | 17.8 |
|  | *Ephemerella sp.JMW1* | NC | 4 | 0 | 10.3 |
|  | *Ephemerella sp.JMW2* | AB | 1 | – | 18.3 |
|  | *Ephemerella sp.JMW3* | PA | 1 | – | 4.6 |
|  | *Ephemerella sp.LJ1* | NB | 5 | 0.9 | 11.2 |
|  | *Ephemerella sp.LJ2* | MN | 10 | 0.3 | 12.7 |
|  | *Ephemerella sp.LJ3* | NC | 4 | 0 | 4.6 |
|  | *Ephemerella sp.LJ4* | TN | 2 | 0 | 7.1 |
|  | *Ephemerella sp.XZ1* | NB | 13 | 4.4 | 12.8 |
|  | *Ephemerella subvaria* | ME, NB, NY, ON, PA | 28 | 1.4 | 11.3 |
|  | *Ephemerella tibialis* | AB, BC, CO, WA | 7 | 3 | 17.7 |
|  | *Eurylophella aestiva* | **FL**, GA, NY, PA, VA | 16 | 1.4 | 14.9 |
| ***** | *Eurylophella bicolor* | AL, ME, MB, NB, PA, TN, VT | 23 | 8.8 | 13.4 |
|  | *Eurylophella bicoloroides* | NY, PA | 6 | 0.8 | 16.1 |
|  | *Eurylophella doris* | DE, GA, IN, MD, NC, SC | 14 | 0.9 | 4.4 |
|  | *Eurylophella enoensis* | NC | 3 | 0 | 15.6 |
| ***** | *Eurylophella funeralis* | GA, NY, NC, NS, PA, QC, VT, VA, WV | 27 | 16.3 | 15.1 |
|  | *Eurylophella lutulenta* | VT | 3 | 0.3 | 12.9 |
| ***** | *Eurylophella macdunnoughi* | NB, NY, PA, VT, VA, WV | 14 | 20.4 | 10.7 |
|  | *Eurylophella minimella* | ME, PA | 3 | 0.9 | 13.4 |
|  | *Eurylophella oviruptis* | NC | 5 | 2 | 18.7 |
|  | *Eurylophella poconoensis* | ME, NC, PA | 8 | 2 | 4.4 |
|  | *Eurylophella prudentalis* | DE, ME, NB, NL, PA, VT, VA | 11 | 1.2 | 14.9 |
|  | *Eurylophella sp.DHF1* | SC | 2 | 0.8 | 13.9 |
|  | *Eurylophella temporalis* | ME, MB, NB, ON | 22 | 1.3 | 6.8 |
|  | *Eurylophella temporalis* group | SC | 1 | – | 14.4 |
|  | *Eurylophella verisimilis* | DE, GA, ME, NB, NY, NC, PA, QC, TN, VT, VA | 30 | 4.3 | 10.7 |
|  | *Penelomax septentrionalis* | NB, PA | 4 | 0.5 | 16.8 |
|  | *Serratella frisoni* | MO | 2 | 0 | 13.4 |
| ***** | *Serratella micheneri* | CA, CO | 8 | 15.8 | 11.9 |
| ***** | *Serratella serrata* | NY, NC, PA, TN | 7 | 16 | 5.9 |
|  | *Serratella serratoides* | NY, SC | 2 | 2 | 4 |
|  | *Serratella sp.JMW1* | NC | 2 | 0.6 | 4 |
|  | *Serratella sp.JMW2* | NB | 1 | – | 14.3 |
| ***** | *Teloganopsis deficiens* | FL, GA, NC, PA | 125 | 19.4 | 18.7 |
| **Ephemeridae** | | | | | |
|  | *Ephemera blanda* | NC | 7 | 1.7 | 7.9 |
|  | *Ephemera guttulata* | NC | 2 | 1.2 | 18.3 |
|  | *Ephemera simulans* | AB, CO, IN, MB, MI, NB, OH, ON | 50 | 2.7 | 9 |
| ***** | *Ephemera varia* | NB, NC, ON, PA | 36 | 8.9 | 7.9 |
|  | *Hexagenia atrocaudata* | ON | 3 | 0.2 | 10.1 |
|  | *Hexagenia bilineata* | IL, OK | 5 | 1 | 7.7 |
| ***** | *Hexagenia limbata* | FL, IL, IN, MB, NB, OK, ON, SK, SD | 91 | 10.1 | 3.3 |
|  | *Hexagenia orlando* | FL | 1 | – | 3.3 |
|  | *Hexagenia rigida* | ON | 7 | 0.6 | 6.5 |
| **Heptageniidae** | | | | | |
|  | *Arthroplea bipunctata* | NY | 5 | 0.2 | 18.4 |
|  | *Cinygmula kootenai* | BC | 1 | – | 12.1 |
|  | *Cinygmula mimus* | CO | 2 | 0.9 | 13.3 |
|  | *Cinygmula sp.JMW1* | BC | 2 | 0.2 | 20.3 |
|  | *Cinygmula sp.JMW2* | AB, BC | 3 | 0.6 | 8.9 |
|  | *Cinygmula sp.JMW3* | AB | 1 | – | 8.2 |
|  | *Cinygmula sp.JMW4* | AB, BC | 7 | 1.1 | 7.6 |
|  | *Cinygmula sp.JMW5* | AB, BC, ON | 4 | 0.3 | 7.6 |
| ***** | *Cinygmula subaequalis* | NB, NC, NS | 156 | 7 | 8.9 |
|  | *Ecdyonurus criddlei* | CA | 5 | 0.5 | 19 |
|  | *Epeorus albertae* | AB, CO | 3 | 0.3 | 14.3 |
|  | *Epeorus deceptivus* | AB | 2 | 0.2 | 19.5 |
|  | *Epeorus dispar* | NC | 4 | 1.2 | 15.5 |
|  | *Epeorus fragilis* | ME, ON | 3 | 1.3 | 8 |
|  | *Epeorus pleuralis* group | NY, NC, NS | 42 | 3.8 | 8 |
|  | *Epeorus grandis* | AB | 1 | – | 17.2 |
|  | *Epeorus longimanus* | AB, CA, CO | 8 | 2.7 | 12.6 |
|  | *Epeorus nr. dulciana* | CA | 3 | 1.1 | 14.3 |
|  | *Epeorus sp.LJ1* | NB | 4 | 1.2 | 3.6 |
|  | *Epeorus subpallidus* | NC | 1 | – | 12 |
|  | *Epeorus vitreus* | AL, GA, NB, NY, NC, NS, PA, TN | 74 | 2.8 | 3.6 |
|  | *Heptagenia adaequata* | SK | 1 | – | 10.8 |
|  | *Heptagenia culacantha* | PA | 1 | – | 13.3 |
|  | *Heptagenia elegantula* | IL, SK, ND, SD | 21 | 1.6 | 9.9 |
|  | *Heptagenia flavescens* | IL, IN, SD, NE | 10 | 1.9 | 11 |
|  | *Heptagenia julia* | NC | 2 | 0.5 | 5.1 |
|  | *Heptagenia marginalis* | KY, PA | 2 | 0.2 | 7.6 |
|  | *Heptagenia nr. townesi* | NC, SC | 2 | 1 | 6.7 |
|  | *Heptagenia nr. dolosa* | GA, NC | 2 | 1.4 | 6.7 |
|  | *Heptagenia pulla* | MB, SK | 32 | 0.8 | 8.7 |
|  | *Heptagenia solitaria* | CO, MT | 3 | 0.5 | 3.8 |
|  | *Heptagenia sp.LJ1* | NB | 2 | 0.3 | 3.8 |
|  | *Heptagenia whitingi* | SK, | 3 | 1.1 | 11 |
|  | *Ironodes sp.JMW1* | CA | 2 | 0 | 19.3 |
|  | *Leucrocuta aphrodite* | ME, NB, NY, NC, PA, TN | 12 | 3.3 | 5.9 |
| ***** | *Leucrocuta hebe* | IL, MB, NB, NS, ON, PA, SK | 166 | 15.6 | 5.9 |
|  | *Leucrocuta juno* | NC, TN | 2 | 0.6 | 4.3 |
|  | *Leucrocuta maculipennis* | IN, SK | 4 | 0.8 | 8.6 |
|  | *Leucrocuta minerva* | PA | 2 | 0.2 | 9.2 |
|  | *Leucrocuta sp.CJ1* | NC | 1 | – | 4.3 |
|  | *Leucrocuta sp.LJ1* | IN | 1 | – | 12.5 |
|  | *Leucrocuta sp.LJ2* | NC, TN | 3 | 0.8 | 11.2 |
|  | *Leucrocuta sp.LJ3* | NC | 2 | 1.1 | 11.1 |
|  | *Leucrocuta thetis* | NC | 8 | 1 | 5.5 |
|  | *Maccaffertium exiguum* | FL, IL, IN | 6 | 1.4 | 10.7 |
|  | *Maccaffertium ithaca* | AL, GA, NB, PA, TN | 35 | 3.7 | 4.5 |
|  | *Maccaffertium lenati* | GA | 1 | – | 7.9 |
|  | *Maccaffertium mediopunctatum arwini* | IL, IN | 5 | 2 | 2 |
|  | *Maccaffertium mediopunctatum mediopunctatum* | ME, MI, ON, PA | 11 | 1.4 | 2 |
|  | *Maccaffertium meririvulanum* | PA | 1 | – | 8.4 |
| ***** | *Maccaffertium mexicanum integrum* | AR, FL, IL, IN, SC | 12 | 19.7 | 14.3 |
| ***** | *Maccaffertium modestum* | GA, NB, NC, PA, SC, | 16 | 14.3 | 4.5 |
|  | *Maccaffertium pudicum* | MD, NC, PA | 16 | 4.6 | 8.4 |
|  | *Maccaffertium pulchellum* | IL,ON | 7 | 3.2 | 8.4 |
|  | *Maccaffertium smithae* | **DE**, FL, GA, **ME**, **NB**, NC, PA, SC | 39 | 3.7 | 7.5 |
|  | *Maccaffertium terminatum* | CO, GA, IL, MB, NB, PA, QC, SK, TX | 25 | 3.6 | 5.2 |
|  | *Maccaffertium vicarium* | IN, ME, NB, NL, NC, NS, OH, ON, QC, SK | 59 | 4.7 | 5.1 |
|  | *Nixe flowersi* | IN | 1 | – | 15.6 |
|  | *Nixe inconspicua* | IL, IN, ON | 5 | 4.1 | 14 |
|  | *Nixe joernensis* | **MB** | 1 | – | 14.9 |
|  | *Nixe lucidipennis* | MB | 4 | 0 | 15.7 |
|  | *Nixe rusticalis* | NB | 14 | 0.8 | 16.4 |
|  | *Nixe sp.JMW1* | IN | 2 | 0 | 15.6 |
|  | *Nixe sp.JMW2* | IL | 1 | – | 15.8 |
|  | *Nixe sp.JMW3* | ON | 3 | 0.5 | 14 |
|  | *Nixe spinosa* | NC | 4 | 0.2 | 16.1 |
|  | *Pseudiron centralis* | IL | 1 | – | 18 |
|  | *Rhithrogena amica* | NC, TN | 4 | 0.3 | 6.5 |
|  | *Rhithrogena anomala* | NC, TN, | 9 | 1.6 | 3.8 |
|  | *Rhithrogena manifesta* | MB | 4 | 0 | 10.5 |
|  | *Rhithrogena nr. rubicunda* | NB | 12 | 2.5 | 3.8 |
|  | *Rhithrogena robusta* | BC | 1 | – | 19.4 |
|  | *Rhithrogena sp.JMW1* | MB | 1 | – | 10.5 |
|  | *Rhithrogena uhari* group | GA, NC, TN, | 14 | 3.9 | 10 |
|  | *Stenacron carolina* | SC | 1 | – | 8.5 |
| ***** | *Stenacron interpunctatum* | AB, AR, GA, IL, IN, ME, MB, MI, MN, MO, NB, NC, ON, PA, QC, SK, SC, TN | 62 | 19.7 | 8.5 |
| ***** | *Stenonema femoratum* | AR, IL, IN, ME, MB, OH, ON, PA, SK | 42 | 14.5 | 13.1 |
| **Isonychiidae** | | | | | |
|  | *Isonychia arida* | GA | 2 | 0.3 | 15.8 |
|  | *Isonychia berneri* | FL | 1 | – | 16.3 |
| ***** | *Isonychia bicolor* | GA, IL, IN, NB, NY, PA, | 30 | 5.5 | 9.5 |
|  | *Isonychia campestris* | SK | 2 | 0.2 | 12.4 |
|  | *Isonychia rufa* | IL, **SK** | 3 | 0.8 | 16.4 |
|  | *Isonychia sicca* | IN | 4 | 2.2 | 12.4 |
|  | *Isonychia sp.DHF1* | SC | 1 | – | 13.1 |
|  | *Isonychia sp.JMW1* | FL, PA | 3 | 1.6 | 14.9 |
|  | *Isonychia sp.JMW2* | NC | 1 | – | 12.9 |
|  | *Isonychia sp.LJ1* | NC | 3 | 1.1 | 9.5 |
|  | *Isonychia tusculanensis* | NC, TN | 3 | 0.6 | 12.9 |
| **Leptohyphidae** | | | | | |
|  | *Leptohyphes apache* | VER | 2 | 0 | 4.7 |
|  | *Leptohyphes musseri* | VER | 3 | 0.2 | 4.7 |
|  | *Leptohyphes zalope* | VER | 3 | 3.3 | 20 |
| ***** | *Tricorythodes explicatus* | NM, TX | 2 | 19.8 | 6.6 |
|  | *Tricorythodes fictus* | TX | 1 | – | 23.2 |
|  | *Tricorythodes mosegus* | MB, ON | 3 | 0.3 | 6.6 |
|  | *Tricorythodes sp.CA1* | CA | 3 | 0.2 | 4.9 |
|  | *Tricorythodes sp.CA2* | CA | 2 | 0.3 | 4.9 |
|  | *Tricorythodes sp.CA3* | CA | 3 | 0.2 | 18.6 |
|  | *Tricorythodes sp.CA4* | CA | 4 | 0.7 | 21.2 |
|  | *Tricorythodes sp.JMW1* | ON | 1 | – | 21.1 |
|  | *Tricorythodes sp.JMW2* | ON | 2 | 0.3 | 21.4 |
|  | *Tricorythodes sp.JMW3* | ON | 3 | 0.3 | 18.9 |
|  | *Tricorythodes sp.JMW4* | VER | 3 | 0.5 | 20.2 |
| **Leptophlebiidae** | | | | | |
|  | *Choroterpes basalis* | IL | 2 | 1 | 17 |
| ***** | *Farrodes sp.JMW1* | VER | 5 | 13.6 | 17.9 |
| ***** | *Habrophlebia vibrans* | NC, NS | 6 | 15.2 | 24.7 |
|  | *Habrophlebiodes americana* | NC, TN | 4 | 3.1 | 17.9 |
|  | *Hydrosmilodon primanus* | VER | 2 | 0 | 21.2 |
| ***** | *Leptophlebia cupida* | IL, IN, MB, NB, SK | 24 | 14.3 | 8.2 |
|  | *Leptophlebia intermedia* | IL, ME, ON, VA | 6 | 3.5 | 16.7 |
|  | *Leptophlebia nebulosa* | AB, ME, MB, NB, ON | 22 | 4.5 | 8.2 |
|  | *Leptophlebia sp.XZ1* | ON | 1 | – | 8.4 |
|  | *Neochoroterpes oklahoma* | NM | 3 | 0.6 | 17 |
|  | *Paraleptophlebia jeanae* | IN | 6 | 1.9 | 7.3 |
|  | *Paraleptophlebia adoptiva* | ON | 1 | – | 13.6 |
|  | *Paraleptophlebia cf. debilis* | NB, NS | 4 | 1.7 | 9.2 |
|  | *Paraleptophlebia debilis* | BC, MB, SK | 6 | 1.4 | 5.8 |
|  | *Paraleptophlebia gregalis* | CA | 1 | – | 14.7 |
|  | *Paraleptophlebia guttata* | NC, NS | 2 | 4.6 | 20.8 |
|  | *Paraleptophlebia heteronea* | CO, WA | 5 | 3.1 | 15.6 |
|  | *Paraleptophlebia kirchneri* | TN | 2 | 0 | 20.2 |
|  | *Paraleptophlebia memorialis* | WA | 2 | 0.3 | 8.4 |
| ***** | *Paraleptophlebia mollis* | MB, NB, NS, ON | 145 | 7.7 | 11.5 |
|  | *Paraleptophlebia ontario* | IL | 1 | – | 7.3 |
|  | *Paraleptophlebia praepedita* | MB | 11 | 1 | 17.1 |
|  | *Paraleptophlebia sp. CHU1* | MB | 4 | 0 | 5.8 |
|  | *Paraleptophlebia sp.JMW1* | CA | 2 | 0.6 | 8.4 |
|  | *Paraleptophlebia sp.JMW2* | CA | 1 | – | 14.7 |
|  | *Paraleptophlebia sp.JMW3* | NC | 1 | – | 15.7 |
| ***** | *Paraleptophlebia sp.JMW4* | NC, SC | 3 | 5.1 | 14.8 |
|  | *Paraleptophlebia sp.XZ1* | NB | 3 | 0.3 | 11.5 |
|  | *Paraleptophlebia sticta* | IN | 3 | 0.5 | 20.5 |
|  | *Paraleptophlebia vaciva* | BC | 1 | – | 19 |
|  | *Paraleptophlebia volitans* | FL, NB | 8 | 2 | 17.1 |
|  | *Thraulodes pacaya* | VER | 2 | 0 | 19 |
|  | *Thraulodes sp.JMW1* | VER | 2 | 0 | 16.7 |
|  | *Thraulodes speciosus* | AZ | 2 | 0.2 | 16.7 |
| **Metretopodidae** | | | | | |
|  | *Metretopus borealis* | AB, MB, NB | 9 | 3.3 | 14.1 |
|  | *Siphloplecton basale* | IN, NB, ON, SK | 9 | 2 | 14.4 |
| **Neoephemeridae** | | | | | |
|  | *Neoephemera purpurea* | TN | 3 | 0.8 | 18.5 |
| **Palingeniidae** | | | | | |
|  | *Pentagenia vittigera* | IL, IN | 3 | 1.6 | 12.7 |
| **Polymitarcyidae** | | | | | |
| ***** | *Ephoron album* | SK | 17 | 9 | 6.3 |
|  | *Ephoron leukon* | ON | 4 | 0.6 | 6.3 |
| **Potamanthidae** | | | | | |
|  | *Anthopotamus verticis* | IN | 3 | 0.2 | 17.2 |
| **Siphlonuridae** | | | | | |
|  | *Parameletus chelifer* | MB | 2 | 0.3 | 19.4 |
|  | *Parameletus midas* | MB,NB | 12 | 1.1 | 18.4 |
| ***** | *Siphlonurus alternatus* | ME, MB, MI, NB, NS, ON | 82 | 9.4 | 6.3 |
|  | *Siphlonurus barbaroides* | NB, NS | 10 | 1.6 | 12 |
|  | *Siphlonurus marshalli* | MO | 2 | 0 | 13.2 |
|  | *Siphlonurus minnoi* | IN | 1 | – | 12.1 |
|  | *Siphlonurus nr. quebecensis* | NB | 1 | – | 13 |
|  | *Siphlonurus occidentalis* | CO | 3 | 0.9 | 3.8 |
|  | *Siphlonurus phyllis* | MB, SK | 20 | 1.4 | 6.3 |
|  | *Siphlonurus quebecensis* | MD, NB, ON | 25 | 0.8 | 12.1 |
|  | *Siphlonurus rapidus* | ME, MI, NB, ON | 7 | 1.5 | 1.3 |
|  | *Siphlonurus sp.JMW1* | ME, NS | 3 | 0.3 | 1.6 |
|  | *Siphlonurus sp.LJ1* | NB | 1 | – | 12.8 |
|  | *Siphlonurus sp.XZ2* | NB | 1 | – | 12.5 |
|  | *Siphlonurus sp.XZ1* | AB, BC | 5 | 1.1 | 3.8 |
|  | *Siphlonurus typicus* | IN, ON | 4 | 0.9 | 1.3 |

All distributional records use standard 2-letter (or 3-letter, for Mexico) postal abbreviations. MNID = minimum interspecific K2P distance, MXID = maximum intraspecific K2P distance. Species marked with a ‘*’ have ≥ 5.0% maximum intraspecific sequence divergence. New state/provincial distribution records are in bold font.
