# Supplementary material for: A DNA Barcode Library for North American Ephemeroptera: Progress and Prospects
Source: PLoS One. 2012 May 30;7(5):e38063. doi: 10.1371/journal.pone.0038063 (PMC3364165; doi:10.1371/journal.pone.0038063)
Supplement: Table S2 — Updated identifications for Ephemeroptera specimens with published barcode records. (DOC) [file pone.0038063.s003.doc]

**Table S2** Updated identifications for Ephemeroptera specimens with published barcode records

| **Published Species** | **Updated Identification** | **Publication** | **BOLD Specimen IDs** | **Notes** |
| --- | --- | --- | --- | --- |
| *Ecdyonurus criddlei* | *Nixe lucidipennis* | [1] | 07PROBE-2008 07PROBE-2016 07PROBE-2416 | These specimens shared a haplotype with an adult male *N. lucidipennis* from Indiana (not included in this analysis) and eggs dissected from females and larvae were of the *Nixe* type, with well-defined mesh-like ridges surrounding knob-terminated coiled threads. |
| *Paraleptophlebia aquilina* | *Paraleptophlebia mollis* | [1] | 07PROBE-07102 07PROBE-2286 07PROBE-2323 | Specimens were reexamined by both LMJ and JMW and found to match *P. mollis* rather than the initial identification of *P. aquilina.* |
| *Heptageniid* XZ sp. CHU1 | *Nixe joernensis* | [1] | 07PROBE-2220 | Additional barcoded larvae (not included in this analysis) closely match the description of *N. joernensis.* This species has not been reported from North America previously. |
| *Heptageniid* XZ sp. CHU2 | *Rhithrogena* sp.JMW1 | [1] | 07PROBE-2403 | The form of the wings, thoracic sutures, and subanal plate fit *Rhithrogena* well, but a species name cannot be confidently applied to females. |
| *Procloeon fragile* | *Procloeon pennulatum* | [1] | 07PROBE-2277 07PROBE-2276 07PROBE-2279 07PROBE-2113 07PROBE-2143 | Both larvae and adults fit the North American concept of *P. pennulatum.* |
| *Leucrocuta jewetti* | *Leucrocuta hebe* | [1] | 07PROBE-2461 07PROBE-07050 07PROBE-07025 07PROBE-2457 07PROBE-2280 07PROBE-07028 07PROBE-2462 07PROBE-07031 07PROBE-2452 07PROBE-2463 | *Leucrocuta jewetti* and *L. hebe* are difficult to separate; *L. jewetti* is primarily known from the northwestern United States and further sampling of *Leucrocuta* from eastern North America shows all of them to be *L. hebe.* |
| *Plauditus cf. dubius* | *Iswaeon anoka* | [1] | 07PROBE-2433 07PROBE-2413 07PROBE-2434 07PROBE-2409 07PROBE-2406 07PROBE-2415 07PROBE-2428 07PROBE-2400 07PROBE-2398 07PROBE-2435 07PROBE-2417 07PROBE-07037 07PROBE-07073 07PROBE-2427 07PROBE-07075 07PROBE-2426 07PROBE-2419 07PROBE-2421 07PROBE-2422 07PROBE-2424 07PROBE-2432 07PROBE-2436 07PROBE-2429 07PROBE-07104 07PROBE-2425 | The original identification was based on the difficult to identify adults. Sequencing of larvae and additional adults clearly show this to be *I. anoka*. |
| *Epeorus deceptivus* | *Epeorus longimanus* | [2] | 773_ER_CO | We were unable to reexamine the original specimen identified as *E. deceptivus*, but the COI sequence groups within *E. longimanus* and not with other specimens confirmed as *E. deceptivus* |
| *Siphlonurus quebecensis* (in part) | *Siphlonurus* spJMW1 | [2] | 1B_NR_ME | *Siphlonurus quebecensis* has a distinct color pattern and unique genitalia, but the most comprehensive and widely used key for the adults is difficult to interpret and can lead to many species being identified as *S. quebenensis*. One previously published barcode groups together with *Siphlonurus* spJMW1, a species that does not appear to match any named species, but has distinct genitalia. |
| *Siphlonurus quebecensis* (in part) | *Siphlonurus rapidus* | [2] | 17B_LAR_ME | This specimen was sister to other *S. rapidus* in our analysis and highly divergent from other *S. quebecensis*. |
| *Baetis flavistriga* | *Baetis phoebus* | [2] | 830_HC_SK | This specimen fits the concept of *B. phoebus*, which was recently removed from synonymy with *B. flavistriga* [3]. |
| *Maccaffertium modestum* (in part) | *Maccaffertium smithae* | [2] | 16_LAR_ME 53_ARLF_ME | *M. smithae* was previously only known from the southeastern United States and was considered to be closely related, and possibly synonymous with, *M. modestum* [4]. Our results show that *M. smithae* is more widespread throughout eastern North America than previously thought and that many specimens identified as *M. modestum* are actually *M. smithae*. |
| *Maccaffertium modestum* (in part) | *Maccaffertium pulchellum* | [2] | 618_LC_ON | These two species are difficult to separate, especially as adults. Barcodes from larvae and additional adults identified as *M. pulchellum* show a previously barcoded specimen was misidentified. |

**References**

1. Zhou X, Adamowicz SJ, Jacobus LM, DeWalt RE, Hebert PDN (2009) Towards a comprehensive barcode library for arctic life - Ephemeroptera, Plecoptera, and Trichoptera of Churchill, Manitoba, Canada. Frontiers in Zoology 6: 30. doi:10.1186/1742-9994-6-30.

2. Ball SL, Hebert PDN, Burian SK, Webb JM (2005) Biological identifications of mayflies (Ephemeroptera) using DNA barcodes. Journal of the North American Benthological Society 24: 508–524.

3. Zhou X, Jacobus LM, DeWalt RE, Adamowicz SJ, Hebert PDN (2010) Ephemeroptera, Plecoptera, and Trichoptera fauna of Churchill (Manitoba, Canada): insights into biodiversity patterns from DNA barcoding. Journal of the North American Benthological Society 29: 814–837. doi:10.1899/09-121.1.

4. Bednarik AF, McCafferty WP (1979) Biosystematic revision of the genus *Stenonema* (Ephemeroptera: Heptageniidae). Canadian Bulletin of Fisheries and Aquatic Sciences 201: 1–73.
