# Supplementary material for: A DNA Barcode Library for North American Ephemeroptera: Progress and Prospects
Source: PLoS One. 2012 May 30;7(5):e38063. doi: 10.1371/journal.pone.0038063 (PMC3364165; doi:10.1371/journal.pone.0038063)
Supplement: Text S1 — Taxonomic notes on selected North American Ephemeroptera species with barcode records. (DOC) [file pone.0038063.s005.doc]

**Text S1 Taxonomic notes on selected North American Ephemeroptera species with barcode records**

1. *Acentrella turbida* (McDunnough): Four haplotype groups were present. LMJ (unpublished) has noted differences in color pattern and leg setation. All specimens are from eastern North America, but the type locality of *A. turbida* is in western Alberta, Canada and we anticipate western specimens are likely to form an additional barcode cluster.
2. *Acentrella parvula* (McDunnough): Three haplotype groups were present. One contains a topotypical specimen (Tillsonburg, Ontario) as well as specimens from Saskatchewan and New Brunswick. It is possible one of the barcode clusters represents the recently described *A. rallatoma* Burian & Myers [1].
3. *Acentrella insignificans* (McDunnough): Two clusters were present. One consisted of specimens from Arizona, while the other is from ‘Klamath’ (likely in Oregon or California).
4. *Acerpenna pygmaea* (Say): The different clusters of *A. pygmaea* are not entirely allopatric as one cluster consists of specimens from Manitoba, New Brunswick, and Florida. One cluster of *A. pygmaea* is represented by a single specimen from Illinois and may represent *A. harti* (McDunnough), which was originally described from that state and was synonymized with *A. pygmaea* [2] . The clade of *A. pygmaea* from Alabama and Florida may represent *A. spiethi* (Berner), which has been collected from the Florida localities; *A. spiethi* was synonymized with *A. pygmaea* [3].
5. *Baetis adonis* Traver: Two clusters were present, both composed of specimens from near the type locality in southern California (“San Gabriel Mountains”) and both separated from clusters of *B. tricaudatus* by relatively low divergences. All specimens were reexamined to confirm their identification by members of the Aquatic Bioassessment Lab, California State University, Chico, California.
6. *Baetis flavistriga* McDunnough: This species will be treated in further detail in a forthcoming project on the *Baetis* of eastern North America.
7. *Baetis tricaudatus* Dodds: This species has 6 synonyms, is transcontinental in distribution, and is known for having extensive phenotypic variation. Five barcode clusters were present and one of them includes several divergent haplotype groups. Differences in the color pattern of adult males were noted among the haplotype clusters, as can be seen on BOLD images for BIOUG00637-D07, BIOUG00640-C05, BIOUG00637-G12, and 09CPMAY-129. BIOUG00637-D07 matches the description of *B. piscatoris* Traver fairly well, but larvae from the same locality fit the concept of *B. tricaudatus* rather than *B. piscatoris*, which was only recently discovered [4] .
8. *Baetis intercalaris* McDunnough: Three haplotype groups were present. Specimens from Florida showed the normal colour pattern for the species and formed a group sister to specimens from Indiana. The Indiana specimens are larvae with much more contrastingly colored abdominal terga than is normally observed in *B. intercalaris*. The third haplotype group consisted of 'typical' *B. intercalaris*.
9. *Callibaetis ferrugineus* (Walsh): This species has multiple synonyms and extensive variation. Two males (10BBEPT-0115, 10BBEPT-0130) from a single locality were significantly different based on the presence or absence of brown staining in the costal area, but they shared barcodes.
10. *Centroptilum triangulifer* (McDunnough): Two widely divergent haplotype groups were observed in this obligate parthenogen. One appears as a sister group to the sexual *C. alamance*. This clade corresponds to *C. triangulifer* and *C. alamance* sensu Funk et al.[5]. The second group includes specimens from Pennsylvania and South Carolina that, although morphologically indistinguishable from the other *C. triangulifer,* probably represent a distinct species. Allozymes confirm the deep divergence between this group and the *C. triangulifer* + *C. alamance* clade, with unique alleles at four loci (DHF, unpublished). In addition to the genetic differences, this group also exhibits several biological differences including morning (rather than afternoon/evening) emergence of subimagos and a winter egg diapause (DHF, unpublished).
11. *Diphetor hageni* (Eaton): There are three synonyms of this species and we observed three monophyletic clusters, one from California, one from Saskatchewan, and one with specimens from Florida, Indiana, and Pennsylvania. The latter cluster had divergences as high as 6.3%. California specimens may represent *D. devinctus* (Traver), recently synonymized with *D. hageni* [6]
12. *Fallceon quilleri* (Dodds): Six species are currently synonymized with *F. quilleri*, which is now considered to have a nearly transcontinental distribution. One cluster consisted of specimens from Colorado, the state of the type locality and fit the species concept well. Specimens collected in New Mexico and Arizona better fit the concept of *F. endymion* (Traver), a synonym of *F. quilleri*.
13. *Baetis magnus* McCafferty & Waltz: Two paraphyletic haplotype groups were present. One cluster comes from an isolated mountain range in Veracruz, Mexico and the other haplotype group consists of specimens from Colorado.
14. *Centroptilum minor* (McDunnough): Two haplotype groups were present. One included specimens from Pennsylvania and South Carolina that were from obligate parthenogenetic lines (DHF, unpublished)
15. *Procloeon viridoculare* (Berner): Two deeply divergent haplotype groups were observed. This species has one synonym, *Procloeon irrubrum* Lowen & Flannagan that may prove to be valid.
16. *Dolania americana* Edmunds & Traver: Previous allozyme work indicated populations from the Yellow River in southeastern Alabama were reproductively isolated from populations in the Blackwater River in northwest Florida [7] Our DNA barcoding data supports this observation as two divergent clusters occurred in our analysis. One cluster was represented by a single specimen from the Blackwater River in Florida and the other cluster had specimens from the Chickasaw River, Alabama, and the Yellow River, Florida.
17. *Caenis amica* Hagen: Three haplotype groups were present. One Florida specimen clustered together with a single individual of *C. punctata* from Missouri; this may be due to misidentification of either specimen.
18. *Caenis diminuta* Walker: There were two clusters of this species, one of which was composed of two divergent haplotype groups separated by a distance of ~10%. Specimens from Florida, where *C. diminuta* was originally described, occurred in both of the clusters.
19. *Caenis latipennis* Banks: There were two paraphyletic clusters due to the presence of *Caenis* sp.XZ1. One of the *C. latipennis* clusters was composed of individuals collected approximately 30km from the type locality of *C. delicata* Traver, currently considered a synonym of *C. latipennis*. One previously barcoded individual of *C. latipennis* [8] clustered with *C. amica*, but it likely reflects a misidentification.
20. *Dannella lita (Burks):* Two haplotype groups are present. The holotype of *Ephemerella* *unicornis* Needham was recently rediscovered, and it closely resembles *D. lita*. Reared larvae from near its type locale also resemble *D. lita* and may have been the basis for associating the current larval and adult concepts of *D. lita*. The two may be separate species. Alternatively, one cluster could be a variant of *D. provonshai*.
21. *Dannella provonshai* (McCafferty): The North Carolina specimen might be a misidentification of a new species. Specimens from NC/TN differ from the type concept (from Arkansas) with regards to color pattern and possibly size. The “true” *D. provonshai* might be represented by one of the clusters identified as *D. lita* or *D. simplex*.
22. *Dannella simplex* (McDunnough): Two haplotype clusters are present. One might represent a new species, variant or misidentification of *D. provonshai*.
23. *Drunella walkeri* (Eaton): Specimens from North Carolina fit the concept of the synonym *D. wayah* (Traver) and were deeply divergent from specimens from New York and Maine.
24. *Ephemerella dorothea* Needham: This “species” probably represents a complex of numerous cryptic species whose color and morphological variants overlap. Detailed ecological comparisons should be made, because they have been collected from a wide variety of stream habitats in areas with montane geological history. See also notes under *E*. *excrucians*.
25. *Ephemerella dorothea infrequens (McDunnough):* See notes for *E. dorothea.*
26. *Ephemerella excrucians* Walsh: Several polyphyletic haplotype clusters were observed. This species has recently had many species synonymized under it. Some of the clusters in our analysis fit these junior names, such as *E. crenula* Allen & Edmunds and *E. rossi* Allen & Edmunds. Some of the variants currently identified as excrucians complex probably belong instead to the dorothea complex, based on reared adults. The shape of tarsal claws and the armature of abdominal terga do not seem to be reliable for separating species groups. Morphometric analysis of the lateral margins of abdominal terga, as well as color patterns, should be explored.
27. *Ephemerella invaria* (Walker): This species formed a single cluster with high intraspecific divergence; *E. invaria* was previously found to be composed of several divergent groupings [9], some of which were subsequently removed from synonymy (i.e. *E. floripara* McCafferty). Hybridization may be occurring within this monophyletic, but divergent, grouping.
28. *Eurylophella bicolor* (Clemens): Fairly large intraspecific differences were found among geographically disparate populations of this widespread species, but they appear monophyletic.
29. *Eurylophella funeralis* (McDunnough): Two geographically distinct populations (one from an isolated cirque-like formation in SW Virginia known as Burkes Garden, the other from western West Virginia) were quite distinct from the large main group of populations, all which came from the east slope of the Appalachians from Quebec to Georgia. Despite the differences, *E. funeralis* formed a monophyletic group.
30. *Eurylophella macdunnoughi* Funk: COI data indicate this species, as presently recognized, is paraphyletic. The cluster that appears as a sister to *E. verisimilis,* from the Northeast, includes topotypic material. The second cluster includes material from West Virginia and the New River (Ohio) drainage of Virginia. Allozymes also showed these populations to be distinct from the Northeastern group [10]. However, due to allopatry and the absence of reliable morphological differences they were conservatively treated as geographic variants of *E. macdunnoughi* by Funk and Sweeney [11].
31. *Serratella micheneri* (Traver): Two polyphyletic haplotype clusters were present and there is one synonym of this species. One of the clusters is from the approximate type locality in the San Gabriel Mountains in southern California. One of the *S. micheneri* clusters might represent misidentification of a variant of *S. levis* (Day) but more study is needed to determine the bounds of morphological variation within each of these species. Alternatively, one cluster might represent *Ephemerella altana* Allen, currently a junior synonym of *S. micheneri*.
32. *Serratella serrata* (Morgan)*:* This species has recently had many synonyms assigned to it [12]. Some of the clusters in our analysis may be applicable to these junior names. *Serratella serratoides* [synonymized for awhile under *E. molita* (which may in fact be synonymous with *E. needhami*)] may eventually be found to be part of this group, because the type was collected together with otherwise typical *S. serrata* [13], and some putative *S. serrata* variants (not barcoded) have faint maculation on abdominal sterna, similar to *S. serratoides*.
33. *Serratella serratoides* (McDunnough): Current concepts of *S. serratoides* may include an entirely different and new species complex [14], especially if the type concept of *S. serratoides* is found to be part of the *S. serrata* complex (see remarks above). Notably, McDunnough [15] initially misidentified *S. serratoides* as *S. serrata.*
34. *Teloganopsis deficiens* (Morgan): There were three deeply divergent haplotype clusters. Two of these clades may have ecological differences as one only occurred in an impacted reach of White Clay Creek, Pennsylvania and the other only occurred in unimpacted areas [16].
35. *Ephemera varia* Eaton: This is a morphologically distinct species that consisted of two deeply divergent clades in our analysis. One of the clades consisted only of larvae from North Carolina, while the other had specimens from Ontario, New Brunswick, and Pennsylvania. The high divergence could be due to the geographic separation or cryptic diversity, but there is a remote possibility the North Carolina clade could also represent the undescribed larva of *E. traverae* Spieth, which is only known from Missouri and Oklahoma [17].
36. *Hexagenia limbata* (Serville): *Hexagenia limbata* is extremely variable morphologically and currently has 15 synonyms; Spieth [18] treated some of these as subspecies of either *H. limbata* or *H. munda* Eaton (which was later synonymized with *H. limbata* [19]). Three clades were observed in *H. limbata*, one of which fits the concept of *H. venusta* Eaton well, one of the synonyms of *H. limbata*. Most specimens from Canada fit the concept of *H. occulta* (Walker). However, Spieth (1941) notes that all of the subspecies he treated intergrade in areas of sympatry, so examination of additional specimens will be required to determine the status of current synonyms of *H. limbata*.
37. *Leucrocuta hebe* (McDunnough): Three barcode clusters were observed. Specimens from Saskatchewan have only a single pair of spines on the ventral surface of the penes, rather than the two normally observed and may represent a new species.
38. *Maccaffertium mexicanum integrum* (McDunnough): There are currently three synonyms, and our analysis showed a monophyletic cluster with three highly divergent clusters within it. Preliminary observations show that differences in the dark markings of the abdominal terga of adult males are correlated with the clusters, but larvae have not yet been examined in detail.
39. *Maccaffertium modestum* (Banks): This species is known to be highly variable morphologically, as indicated by the fact that it keys out in seven different places in the male adult key [20]. This is undoubtedly a species complex in need of revision.
40. *Stenacron interpunctatum* (Say): Previous barcoding results indicated *S. interpunctatum* may be a species complex [8]. None of the available keys or descriptions for the genus adequately encompass the amount of variation observed in specimens, rendering species level identifications of *Stenacron* tenuous at best. Our results also show multiple clusters within this species, with similarly colored specimens generally clustering together.
41. *Stenonema femoratum* (Say): This species was monophlyetic and all but one specimen formed a single haplotype cluster with little divergence. The remaining specimen was highly divergent from the others.
42. *Tricorythodes explicatus* (Eaton): One specimen of *T. explicatus* was located in its own clade distant from the other *T. explicatus*. This specimen was originally identified as *T. minutus* Traver[8], a species recently synonymized with *T. explicatus* [21]. Given that identification of *Tricorythodes* is difficult and that many species are only known in one lifestage, it is possible *T. minutus* is a distinct species from *T. explicatus*. The synonymy should be reexamined. Anecdotal evidence indicates there may be ecological and swarming behavioural differences in sympatric cryptic species of *Tricorythodes*.
43. *Farrodes* sp. JW1: Few larvae of this genus are described in North America and little is known about the morphological variation within species.
44. *Habrophlebiodes americana* (Banks): Two clusters were observed, and the species currently encompasses the concepts of two nominal species, *H. americana* and *H. betteni* (Needham). Two variants have been observed among larvae morphologically identifiable as *H. americana* (not barcoded) that differ in the pattern of spines on the posterior margins of the abdominal terga. More research is needed to apply names to these variants. Additionally, the possible misidentification of some of the more obscure southeastern United States species should be explored.
45. *Leptophlebia cupida* (Say): There were two clusters in our analysis. Extensive morphological variation has previously been observed [22] and the species has many synonyms.
46. *Ephoron album* (Say): Up to 9% divergence was observed in specimens from the North and South Saskatchewan Rivers, but the divergence between specimens was not correlated with collecting locale.
47. *Siphlonurus alternatus* (Say): The high divergence in *S. alternatus* was due to a single specimen from Maine [8]. It is possible this specimen was misidentified, but *S. alternatus* is usually distinct morphologically so we choose to retain the identification as is.

**References**

1. Burian SK, Myers L (2011) A new species of *Acentrella* Bengtsson (Ephemeroptera: Baetidae) from New York and New England (USA), redescription of the nymph of *A. parvula* (McDunnough), and key to known adult males of Nearctic *Acentrella*. Aquatic Insects 33: 305–334.

2. Waltz RD, Baumgardner DE, Kennedy JH (1998) Character variability and a new synonym of *Acerpenna pygmaea* (Ephemeroptera: Baetidae). Entomological News 109: 257–260.

3. Morihara DK, McCafferty WP (1979) The *Baetis* larvae of North America (Ephemeroptera: Baetidae). Transactions of the American Entomological Society 105: 139–221.

4. McCafferty WP, Meyer MD, Randolph RP, Webb JM (2008) Evaluation of mayfly species originally described as *Baetis* Leach (Ephemeroptera: Baetidae) from California. Proceedings of the Entomological Society of Washington 110: 577–591.

5. Funk DH, Jackson JK, Sweeney BW (2006) Taxonomy and genetics of the parthenogenetic mayfly *Centroptilum triangulifer* and its sexual sister *Centroptilum alamance* (Ephemeroptera:Baetidae). Journal of the North American Benthological Society 25: 417–429. doi:10.1899/0887-3593(2006)25[417:TAGOTP]2.0.CO;2.

6. Meyer MD, McCafferty WP (2001) Hagen’s small minnow mayfly (Ephemeroptera: Baetidae) in North America. Entomological News 112: 255–263.

7. Sweeney BW, Funk DH (1991) Population genetics of the burrowing mayfly *Dolania americana*: Geographic variation and the presence of a cryptic species. Aquatic Insects 13: 17–27.

8. Ball SL, Hebert PDN, Burian SK, Webb JM (2005) Biological identifications of mayflies (Ephemeroptera) using DNA barcodes. Journal of the North American Benthological Society 24: 508–524.

9. Alexander LC, Delion M, Hawthorne DJ, Lamp WO, Funk DH (2009) Mitochondrial lineages and DNA barcoding of closely related species in the mayfly genus *Ephemerella* (Ephemeroptera:Ephemerellidae). Journal of the North American Benthological Society 28: 584–595. doi:10.1899/08-150.1.

10. Sweeney BW, Funk DH, Vannote RL (1988) Electrophoretic study of eastern North American *Eurylophella* (Ephemeroptera: Ephemerellidae) with the discovery of morphologically cryptic species. Annals of the Entomological Society of America (USA).

11. Funk DH, Sweeney BW (1994) The larvae of the eastern North American *Eurylophella* Tiensuu (Ephemeroptera: Ephemerellidae). Transactions of the American Entomological Society 120: 209–286.

12. Jacobus LM, McCafferty WP (2003) Revisionary contributions to North American *Ephemerella* and *Serratella* (Ephemeroptera: Ephemerellidae). Journal of the New York Entomological Society 111: 174–193. doi:10.1664/0028-7199(2003)111[0174:RCTNAE]2.0.CO;2.

13. McDunnough J (1931) New species of North American Ephemeroptera. Canadian Entomologist 63: 82–93.

14. McDunnough J (1931) The eastern North American species of the genus Ephemerella and their nymphs (Ephemeroptera). Canadian Entomologist 63: 187–197,201–216, pl 11–14.

15. McDunnough J (1930) The Ephemeroptera of the north shore of the Gulf of St. Lawrence. Canadian Entomologist 62: 54–62.

16. Sweeney BW, Battle JM, Jackson JK, Dapkey T (2011) Can DNA barcodes of stream macroinvertebrates improve descriptions of community structure and water quality? Journal of the North American Benthological Society 30: 195–216. doi:10.1899/10-016.1.

17. McCafferty WP (1994) Distributional and classificatory supplement to the burrowing mayflies (Ephemeroptera: Ephemeroidea) of the United States. Entomological News 105: 1–13.

18. Spieth HT (1941) Taxonomic studies on the Ephemeroptera. II. The genus *Hexagenia*. American Midland Naturalist 26: 233–280.

19. McCafferty WP (1984) A new synonym in *Hexagenia* (Ephemeroptera: Ephemeridae). Proceedings of the Entomological of Washington 86: 789.

20. Bednarik AF, McCafferty WP (1979) Biosystematic revision of the genus *Stenonema* (Ephemeroptera: Heptageniidae). Canadian Bulletin of Fisheries and Aquatic Sciences 201: 1–73.

21. Baumgardner DE (2009) *Tricorythodes minutus* Traver, a new synonym of *Tricorythodes explicatus* Eaton (Ephemeroptera: Leptohyphidae). Proceedings of the Entomological Society of Washington 111: 57–67.

22. Burian SK (2001) A revision of the genus *Leptophlebia* Westwood in North America (Ephemeroptera: Leptophlebiidae: Leptophlebiinae). Ohio Biological Survey Bulletin New Series 13: 1–80.
